# Leveraging the Immune Response from LIFE Biomaterial and Photon-Flash in Pre-Clinical Pancreatic Cancer Treatment

**DOI:** 10.3390/pharmaceutics17101273

**Published:** 2025-09-29

**Authors:** Michele Moreau, Katelyn Kelly, Serena Mao, Debarghya China, Girmachew Wasihun, Aditya Pandya, MohammadAli Tajik-Mansoury, Daniel Sforza, Devin Miles, Amol K. Narang, Mohammad Rezaee, Wilfred Ngwa, Kai Ding

**Affiliations:** 1 Department of Radiation Oncology and Molecular Radiation Sciences, Johns Hopkins University, Baltimore, MD 21287, USA; kkelly80@jhmi.edu (K.K.); serenachiangmao@gmail.com (S.M.); gwasihu1@jh.edu (G.W.); mtajikm1@jhu.edu (M.T.-M.); dsforza1@jhmi.edu (D.S.); dmiles18@jhmi.edu (D.M.); anarang2@jhmi.edu (A.K.N.); mrezaee1@jhmi.edu (M.R.); wngwa1@jhmi.edu (W.N.); 2 Department of Biomedical Engineering, Johns Hopkins University, Baltimore, MD 21287, USA; dchina1@jhmi.edu; 3 Center for Molecular and Cellular Oncology, Yale School of Medicine, New Haven, CT 06511, USA; aditya.pandya@yale.edu

**Keywords:** ultra-high dose rate (≥40 Gy/s) Flash, LIFE Biomaterial, radiotherapy, immune response, immunohistochemistry

## Abstract

Pre-clinical animal studies evaluating the ‘flash effect’ caused by ultra-high dose rate (≥40 Gy/s) favorably spares normal tissue from radiation-caused toxicity while maintaining anti-tumor effects like conventional (CONV) radiation. The goal of this study is to leverage an immune response resulting from the treatment combination of flash radiotherapy (Flash-RT) and LIFE (liquid immunogenic fiducial eluter) biomaterial incorporating an anti-mouse CD40 monoclonal antibody to enhance the therapeutic ratio in pancreatic cancer. **Methods**: A small animal FLASH radiation research platform (FLASH-SARRP) was utilized to deliver both ultra-high and CONV dose-rate irradiation to treat syngeneic subcutaneous pancreatic tumors generated in 8–10-week-old male and female C57BL6 mice. The efficacy of FLASH versus CONV radiotherapy (RT) at varying doses of 5, 8, 10, and 15 Gy delivered in a single fraction was evaluated by assessing tumor growth and mice survival over time or comparing tumor weight at 10 days post-treatment. **Results**: Similar tumor control capability was observed by the high-dose rate and conventional RT related to the control group. Nevertheless, longer survival was observed for the FLASH group at 5 Gy compared to CONV and control at either 5 Gy, 10 Gy, or 15 Gy doses. Multiplex immunofluorescence and immunohistochemistry results showed higher T-cell infiltration within the combination of RT (either FLASH or CONV) and LIFE biomaterial-treated tumors compared to the control cohort. **Conclusions**: This animal study serves as an impetus for future studies leveraging the immune response using the combination of FLASH and LIFE Biomaterial to enhance the efficacy of pancreatic cancer treatment.

## 1. Introduction

Radiotherapy techniques are employed to mitigate or eliminate abnormal and malignant tissues by depositing the energy of ionizing radiation to disrupt cellular function and inhibit their uncontrolled growth. As a treatment modality, RT has long emphasized optimizing beam delivery to maximize damage to cancer tissue while minimizing damage to nearby normal tissues. However, RT can cause acute and late damage to healthy tissues, leading to toxicities associated with radiation [1,2,3]. Previous studies observed that using ultra-high dose rate (≥40 Gy/s) can create a ‘FLASH effect’ where healthy tissue can be spared while maintaining similar anti-tumor effects as conventional radiation [4,5,6,7,8]. FLASH RT is believed to be a paradigm shift in radiation medicine, as its ultrafast (<1 s) dose delivery eliminates the need for organ motion management and enhances the therapeutic ratio by sparing healthy tissues while effectively targeting tumors.

Pre-clinical studies of Flash-RT, characterizing the FLASH effect, have primarily focused on two domains: sparing normal tissues and improving tumor control. Previous studies have reported significantly reduced injuries to various healthy organs, such as skin, brain, lung, and eye, in FLASH-irradiated mice compared to their CONV counterparts [9,10,11,12,13]. Other studies have reported that FLASH-RT can render the tumor microenvironment more immunogenic; consequently, combination treatment with immunotherapy would have a greater impact on managing tumor growth [12,14,15,16].

Immunotherapy has been at the forefront of cancer combination therapy, along with other standard treatment modalities such as radiotherapy, chemotherapy, and surgery. The immune system, composed of white blood cells and lymphoid tissues [17], serves as the body’s main defense against invading microbes and pathogens. Nonetheless, cancer cells can evade immune system detection by disguising themselves as normal cells, thereby avoiding recognition and elimination by antigen-presenting cells. Immunotherapy is an emerging modality in cancer treatment that improves the immune system’s ability to recognize and eliminate cancer cells. By modulating immune checkpoints, stimulating antigen presentation, or employing engineered immune cells such as CAR-T cells, immunotherapy aims to overcome tumor-mediated immune evasion and improve therapeutic outcomes [18,19,20]. Agonistic CD40 monoclonal antibodies have been used to stimulate antigen-presenting cells (APCs) and encourage anti-tumor T cell replications [21]. However, due to the toxicities associated with existing immunotherapeutic drugs, developing newer and safer targeted therapies that combine immunotherapy with radiotherapy has been growing rapidly [22].

This study utilizes a drug delivery system and a fiducial marker, the LIFE biomaterial, to provide sustained local delivery of a monoclonal antibody (anti-CD40) directly at the tumor site throughout the treatment period. Previous studies have reported that the LIFE Biomaterial can serve as a fiducial marker, effectively identifying the region of interest during RT [23,24]. This study compares the effects of combining FLASH-RT or CONV-RT, each with or without LIFE Biomaterial loaded with an anti-CD40 antibody, on the growth of syngeneic subcutaneous pancreatic tumors in wild-type C57BL/6 mice. Overall survival is evaluated relative to non-treated cohorts and single treatments with either Flash or conventional RT alone. Assessment of immune cell infiltrations within the tumor microenvironment was observed in both non-treated and treated cohorts.

## 2. Materials and Methods

### 2.1. Materials

Titanium Oxide nanopowder (TiO_2_, Anatase, 99.5%, 5 nm), Stock#: US3838, TiO_2_ CAS#: 13463-67-7, Net weight: 100 g) was acquired from Sigma-Aldrich (St. Louis, MO, USA). Omniscan gadodiamide gadolinium-based nanoparticles were obtained from GE Healthcare (Silver Spring, MD, USA). Dimethyl sulfoxide (DMSO), Dulbecco’s modified eagle medium (DMEM), Roswell Park Memorial Institute (RPMI) medium, Trypsin-EDTA (0.25%), Fetal Bovine Serum (FBS, Cat#: 30-2020), Sodium Pyruvate (100 mM, Cat#: 11360070), Penicillin-Streptomycin (10,000 U/mL, Cat#: 15140122), and phosphate-buffered saline pH 7.4) were purchased from either the American Type Culture Collection (Manassas, VA, USA), ThermoFisher Scientific (Waltham, MA, USA), or Life Technologies (Frederick, MD, USA). The monoclonal antibody anti-mouse CD40 (FGK4.5/FGK45) was bought from BioXcell (Lebanon, NH, USA).

### 2.2. Liquid Immunogenic Fiducial Eluter (LIFE) Biomaterial Assembly

LIFE Biomaterials were fabricated following a previously published protocol [23,25]. In this study, LIFE biomaterials consisted of a mixture of two natural polymers: 4% (*w*/*v*) sodium alginate and 2% (*w*/*v*) chitosan, mixed in a 1:1 volume ratio to create a polymer blend. A CT contrast nanoparticle, titanium dioxide (TiO_2_), combined with an MRI contrast gadolinium-based nanoparticle (Omniscan) in a 1:1 volume ratio, was added to the polymer blend mixture to formulate the LIFE Biomaterial. On treatment day, 100 uL of the LIFE biomaterial mixed with 100 µg of an anti-mouse CD40 monoclonal antibody was dispensed intratumorally within the tumor.

### 2.3. Cell Culture Preparations for Mice Inoculation

The pancreatic cancer (KPC) cell line was derived from an LS-Kras; p53+/floxed, Pdx-cre mouse. KPC (C57BL6 genetic background) cells were cultured in DMEM supplemented with 10% FBS and 1% penicillin-streptomycin. Cells were maintained at 37 °C in a dampened incubator with 5% CO_2_. Non-immunodeficient untamed C57BL/6 strain male and female mice (with average body weight = 22 g) were obtained from Taconic Biosciences, Inc. (Germantown, NY, USA), or Charles River (Rockville, MD, USA) at 56–62 days old. They were injected subcutaneously with 1.5 × 10^5^ KPC cells procured in a 100 μL volume of cells apiece into the flanks. Following cancer cell injection, the tumors were allowed to grow to at least 3.0 mm or greater in diameter before initiating treatment. The mice’s body weights were not measured throughout the duration of the studies. Animal experiments were performed according to the procedures and protocols developed by the Johns Hopkins University Animal Care and Use Committee (ACUC) under protocol# MO24M298. Mice maintenance in the Johns Hopkins University animal facility was according to the Institutional Animal Care and Use Committee-approved strategies, consisting of stable environmental conditions, including a 12–14-h light/dark cycle and temperatures between 65 and 75 °F (18–23 °C) with 40–60% humidity. The mice’s tumor growth was calculated as V = 0.5 × length × (width^2^). A precise ruler was used to measure the longitudinal and latitudinal outliers of the tumor mass.

### 2.4. Data Collection and Image Analysis

To evaluate the possibility of LIFE Biomaterial serving as a fiducial marker helping to pinpoint the tumor location during radiotherapy, a human cadaver was utilized to perform CT and MR imaging. The human cadaver study was conducted in accordance with the Johns Hopkins Institutional Review Board (IRB), and the protocol was approved by the Institutional Review Board with Approval Code: IRB-X on 15 January 2018. Frozen individual cadaveric prototypes were thawed and inoculated with 3 mL of LIFE Biomaterial. A CT simulation (TOSHIBA Helical CT (Tampa, FL, USA) image with 2 mm slice depth, 120 kVp, and 100 mA of X-ray tube flow of charge) of the cadaveric model was taken in the recumbent site. A repeated scan of the specimens was taken. A Philips Achieva (Fort Myers, FL, USA) 3.0 T MRI structure with BODY magnetic loop was utilized to acquire magnetic resonance imaging, with a cyclic time of 5.31 ms, a 100 angle of tilt, a percent phase field of view of 70.833, and a slice width of 0.9 mm.

### 2.5. Small Animal FLASH Radiation Research Platform to Deliver Conventional and Flash Radiotherapy

#### 2.5.1. FLASH-SARRP

The FLASH-SARRP system was employed to deliver both CONV and ultra-high dose-rate irradiation using kilovoltage (kV) X-rays. Details of the system’s components and dosimetry performance have been described previously [26]. A brief overview is provided here: The system incorporates two high-capacity rotating-anode X-ray tubes (RAD44, Varex Imaging Inc., Salt Lake City, UT, USA), each powered by a 100 kW generator (CPI International Inc., Palo Alto, CA, USA), to deliver ultra-high dose rate X-rays at short source-to-surface distances (SSDs). Each X-ray source can operate at tube potentials up to 150 kVp, with a broad range of tube currents (5–630 mA) and exposure times (1 to 6300 ms), allowing for precise selection of radiation-dosage in the range of below 1 to over 100 Gray per second, depending on the field size and SSD. The system is integrated with a robotic animal stage capable of high-precision motion in the x, y, and z directions, allowing accurate and reproducible positioning of the mouse bed for the tumor irradiation.

#### 2.5.2. Animal Setup and Irradiation

**Figure 1:**
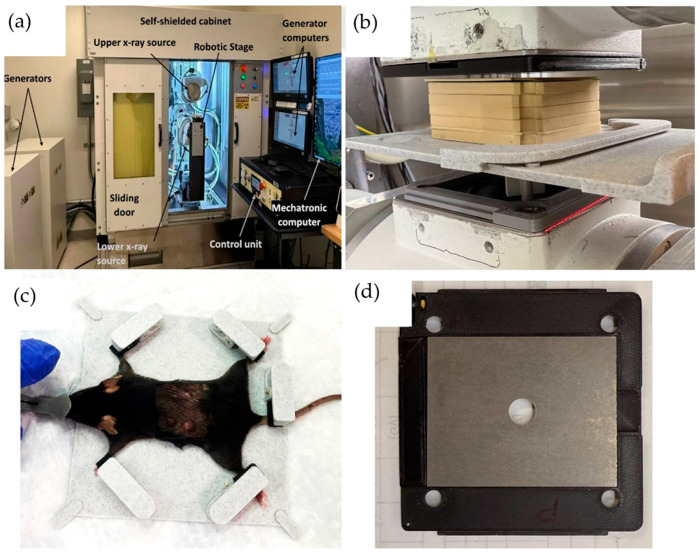
(**a**) FLASH-SARRP system includes robotic stage, (**b**) Dosimetry setup using Gafchromic film and solid slab water, (**c**) mouse bed setup, (**d**) 10 mm diameter collimator inside collimator holder.

A 2.1 mm thick tungsten collimator with a 10 mm diameter circular aperture was used to shape the radiation field. The collimator was mounted to the X-ray tube flange using a custom 3D-printed holder. To ensure high-precision and reproducible mouse positioning, a custom immobilization and docking device was developed and referenced to the mounting clamps on the robotic stage. The mouse was anesthetized using a mobile anesthesia system (Fisher anesthesia machine, Marsing, ID, USA) delivering 2% isoflurane in the medical air via housing connected to the nose cone. The mouse’s front and hind legs were immobilized in fixed locations on the bed, using specially designed clamps, to maintain consistent positioning. The mice were positioned at an SSD of 63 mm. The tumor was aligned under the 10 mm collimator aperture visually and using positioning lasers. Tumor positioning at the center of the field size was verified using beam’s eye view images on radiographic films. A thin metal wire was placed around the tumor, and the immobilized mouse on the robotic bed was adjusted to bring the tumor with wire outline into the center of the irradiation field. A piece of LD-V1 radiographic film was placed underneath the mouse, and a low-energy imaging exposure (42 kVp, 2 mAs) was delivered. The resulting image was used to verify that the wire ring completely fit within the irradiation field, confirming field localization. An illustration of the FLASH-SARRP system is displayed in Figure 1.

Radiation dosimetry was performed using EBT3 Gafchromic film (Ashland Inc., Wayne, NJ, USA) within a solid water phantom composed of 1 mm and 5 mm thick slabs. EBT3 film sheets were laser-cut and placed from 1 to 5 mm depths within the phantom, which was positioned on the robotic stage to mimic the geometry used during mouse irradiation experiments. Percentage depth dose-rate (PDDR), dose-rate profiles, and 2D dose-rate distribution were measured to characterize the dose delivered to the tumor and surrounding tissues. Film calibration was performed using a NIST-traceable 120 kVp X-ray source at the University of Wisconsin Accredited Dosimetry Calibration. A dose rate of 61.5 ± 3.5 Gy/s was delivered for FLASH-RT and 0.95 ± 0.05 Gy/s for CONV RT in this setup.

#### 2.5.3. Experimental Study Design

Mice were used pre-clinically due to their high degree of genetic homology with humans, with nearly 95% of genes in common [27]. Here, three individual studies were conducted to (1) identify the optimal RT dose for either Flash or CONV RT; (2) leverage the immune response using high dose Flash or CONV RT between 10 Gy and 20 Gy combined with LIFE biomaterial_anti-CD40 treatment; (3) influence the immune response using low dose Flash or CONV RT between 5 Gy and 8 Gy combined with LIFE biomaterial_anti-CD40 treatment. In total, 106 C57BL6 male and female mice with an average body weight of 22 g were utilized throughout the course of this study. The sample size for each experiment performed was chosen based on the availability of mice that were housed in our animal facility. There were no explicit criteria set for including experimental units unless the animals reached study endpoints such as tumor size reaching 2 cm, bleeding tumors, or concave ulcerated tumors, hunched behavior, or low body score <2.5, as described in the Johns Hopkins University Animal Care and Use Committee (ACUC) under protocol# MO24M298. Mice ulcers were treated daily with a Veterinus Derma Gel to help mitigate the ulcerated wound. No analgesics were used throughout the duration of these studies. Mice were simply randomized according to tumor size to ensure similar tumor sizes are represented in all the cohorts. Potential confounders were minimized by using mice of the same strain and age, housed in the same mouse holding room and animal facility.

**Figure 2:**
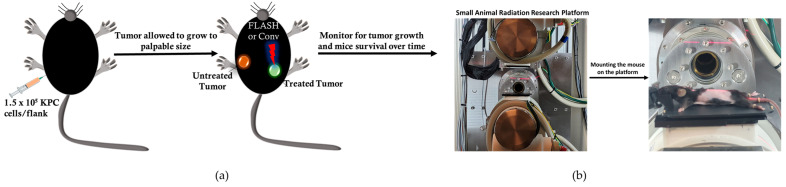
Schematic of the experimental setup. (**a**) C57BL6 mice were inoculated with 1.5 × 10^5^ pancreatic (KPC) cancer cells in bilateral flanks. Only one flank growth per mouse, out of the bilateral tumors, was irradiated, and the mouse cohorts were as follows: (1) no treatment; (2) Flash_5 Gy; (3) CONV_5 Gy; (4) Flash_10 Gy; (5) CONV_10 Gy; (6) Flash_15 Gy; and (7) CONV_15 Gy. (**b**) FLASH-SARRP was utilized to deliver X-rays to the targeted tumor. Anesthetized mice were reproducibly immobilized on the treatment bed and positioned within the radiation field using the robotic stage.

The anti-tumor effect of conventional versus flash photon RT was assessed in C57BL6 mice bearing bilateral subcutaneous tumors (size~3 mm), targeting only one tumor for treatment while observing both tumors to note any change in tumor growth post-treatment and prolonged mouse survival post-treatment, as illustrated in Figure 2a. The experimental unit consists of n = 4–5 mice per cage. The cohorts (n = 8/group) were allocated as follows: no treatment, Flash_5 Gy, CONV_5 Gy, Flash_10 Gy, CONV_10 Gy, Flash_15 Gy, and CONV_15 Gy. The mouse was set up in the FLASH-SARRP system, as illustrated in Figure 2b, FLASH and CONV irradiation of the targeted subcutaneous tumor. A total of 56 C57BL6 male mice bearing bilateral flank tumors were utilized for this analysis.

Next, a multiplex immunofluorescence analysis was performed to assess the immune cells infiltration (CD45, CD3, CD4 and CD8) within the tumor microenvironment at 34 days post-treatment for the following cohorts: No Treatment (n = 5); LIFE Biomaterial_20 µg-Anti-CD40 (n = 4); Flash_10 Gy_LIFE Biomaterial_20 µg-Anti-CD40 (n = 4); Conv_10 Gy_LIFE Biomaterial_20 µg-Anti-CD40 (n = 4); Flash_20 Gy_LIFE Biomaterial_20 µg-Anti-CD40 (n = 4); Conv_20 Gy_LIFE Biomaterial_20 µg-Anti-CD40 (n = 4). 25 C57BL6 female mice bearing bilateral flank tumors were utilized for this analysis. The experimental unit consists of n = 4–5 mice per cage.

Subsequently, 25 C57BL6 female mice bearing a subcutaneous pancreatic right flank tumor were utilized to perform immunohistochemistry analysis on tumor tissues collected 10 days post-treatment to assess immune cell infiltration in the tumor microenvironment. The experimental unit consists of n = 2–3 mice per cage. The assessed cohorts were (a) no treatment (n = 3); (b) LIFE Biomaterial_100 µg-Anti-CD40 (n = 2); (c) Flash_5 Gy (n = 3); (d) Conv_5 Gy (n = 3); (e) Flash_5 Gy_LIFE Biomaterial_100 µg-Anti-CD40 (n = 2); (f) Conv_5 Gy_LIFE Biomaterial_100 µg-Anti-CD40 (n = 2); (g) Flash_8 Gy (n = 3); (h) Conv_8 Gy (n = 3); (i) Flash_8 Gy_LIFE Biomaterial_100 µg-Anti-CD40 (n = 2); (j) Conv_8 Gy_LIFE Biomaterial_100 µg-Anti-CD40 (n = 2).

### 2.6. Histology Staining

The Johns Hopkins Oncology Tissue and Imaging Service (OTIS) Core’s histology technicians procure H&E and Unstained sections using a microtome. This high-precision instrument enables technicians to cut 4-micrometer-thick FFPE (formalin-fixed paraffin-embedded) tissue sections that are then suspended on a 41-degree Celsius warm water bath. Using specialized microscope slides, the technician retrieves the sections from the water bath subsequently mounting them onto the microscope sides for further processing, where the designated H&E sectioned slides are baked at 60 degrees Celsius for 30 min before auto staining for hematoxylin and eosin, and the designated Unstained sectioned slides are set aside—upright—on a specialized drip trays to air dry at room temperature for 48–72 h.

### 2.7. Immunohistochemistry Staining

Immunostaining was performed at the Oncology Tissue and Imaging Services Core of Johns Hopkins University. Immunolabeling for CD3-CD11b dual detection was performed on formalin-fixed, paraffin-embedded sections on a Ventana Discovery Ultra Auto Stainer (Roche Diagnostics, Indianapolis, IN, USA). Briefly, following dewaxing and rehydration on board, epitope retrieval was performed using Ventana Ultra CC1 buffer (catalog #6414575001, Roche Diagnostics, Indianapolis, IN, USA) at 96 °C for 64 min. Primary antibody, anti-CD3 (1:200 dilution; catalog #ab16669, Abcam, Waltham, MA, USA), was applied at 36 °C for 60 min. CD3 primary antibodies were detected using an anti-rabbit NP and anti-NP AP detection system (catalog numbers #07425317001 and #07425325001, Roche Diagnostics, Indianapolis, IN, USA), followed by the Discovery Yellow Detection Kit (Catalog #07698445001, Roche Diagnostics, Indianapolis, IN, USA). Following CD3 detection, primary and secondary antibodies from the first round of staining were stripped from the board using Ventana Ultra CC1 buffer at 93 °C for 8 min. Primary antibody, anti-CD11b (1:10,000 dilution; catalog #ab133357, Abcam, Waltham, MA, USA), was applied at 36 °C for 60 min. CD11b primary antibodies were detected using an anti-rabbit HQ detection system (catalog #7017936001 and #7017812001, Roche Diagnostics, Indianapolis, IN, USA), followed by the Discovery Teal Detection kit (catalog #8254338001, Roche Diagnostics, Indianapolis, IN, USA), counterstaining with Mayer’s hematoxylin, dehydration, and mounting. Immunolabeling for CD8-CD4 dual detection was per-formed on formalin-fixed, paraffin-embedded sections on a Ventana Discovery Ultra Auto Stainer (Roche Diagnostics, Indianapolis, IN, USA). Briefly, following dewaxing and rehydration on board, epitope retrieval was performed using Ventana Ultra CC1 buffer (catalog #6414575001, Roche Diagnostics, Indianapolis, IN, USA) at 96 °C for 64 min. Primary antibody, anti-CD8 (1:125 dilution; catalog #14-0195-82, ThermoFisher Scientific, Waltham, MA, USA), was applied at 36 °C for 60 min, followed by rabbit anti-rat linker antibody (1:500 dilution; catalog #AI4001, Vector Labs, Newark, CA, USA) at 36 °C for 32 min. Linker antibodies were detected using an anti-rabbit NP and anti-NP AP detection system (catalog numbers #07425317001 and #07425325001, Roche Diagnostics, Indianapolis, IN, USA) followed by the Discovery Yellow Detection Kit (Catalog #07698445001, Roche Diagnostics). Following CD8 detection, primary and secondary antibodies from the first round of staining were stripped on the board using Ventana Ultra CC1 buffer at 93 °C for 8 min. Primary antibody, anti-CD4 (1:1000 dilution; catalog# ab183685, Abcam, Waltham, MA, USA), was applied at 36 °C for 60 min. CD4 primary antibodies were detected using an anti-rabbit HQ detection system (catalog #7017936001 and #7017812001, Roche Diagnostics, Indianapolis, IN, USA) followed by the Discovery Teal Detection kit (catalog #8254338001, Roche Diagnostics, Indianapolis, IN, USA), counterstaining with Mayer’s hematoxylin, dehydration, and mounting.

### 2.8. Multiplex Immunofluorescence Methods

Immunostaining was performed at the Oncology Tissue Services Core of Johns Hopkins University School of Medicine. Quadruple immunolabeling for CD8 + CD45 + CD4 + CD3 was performed on formalin-fixed, paraffin-embedded sections on a Ventana Discovery Ultra auto stainer (Roche Diagnostics, Indianapolis, IN, USA). Following dewaxing and rehydration on board, epitope retrieval was performed using Ventana Ultra CC1 buffer (catalog #6414575001, Roche Diagnostics, Indianapolis, IN, USA) at 96 °C for 64 min. Primary antibody, anti-CD8 (1:125 dilution; catalog# 14-0808, eBioscience, Inc., San Diego, CA, USA) was applied at 36 °C for 40 min, followed by rabbit anti-rat linker antibody (1:500 dilution; catalog# AI4001, Vector Labs, Newark, CA, USA) at 36 °C for 32 min. Linker antibodies were detected using an anti-rabbit HQ detection system (catalog# 7017936001 and 7017812001, Roche Diagnostics, Indianapolis, IN, USA), followed by OPAL 520 (NEL871001KT, Akoya Biosciences, Marlborough, MA, USA) diluted 1:150 in 1X Plus Amplification Diluent (catalog # FP1498, Akoya Biosciences, Marlborough, MA, USA). Following CD8 detection, primary and secondary antibodies from the first staining round were stripped on board using Ventana Ultra CC1 buffer at 95 °C for 12 min and neutralization using Discovery Inhibitor (catalog #7017944001, Roche Diagnostics, Indianapolis, IN, USA). Primary antibody, anti-CD45 (1:200 dilution; catalog #702575S, Cell Signaling Technology, Danvers, MA, USA) was applied at 36 °C for 40 min. CD45 primary antibodies were detected using an anti-rabbit HQ detection system (catalog# 7017936001 and 7017812001, Roche Diagnostics, Basel, Switzerland) followed by OPAL 570 (NEL871001KT, Akoya Biosciences, Marlborough, MA, USA) diluted 1:150 in 1X Plus Amplification Diluent (catalog # FP1498, Akoya Biosciences, Marlborough, MA, USA). Immunofluorescence stains were analyzed using Zeiss ZEN Lite software version 3.10. Images were first processed by performing a background subtraction and then an enhanced contour. The resulting images were then analyzed by evaluating the histograms of the images and their corresponding wavelength values. Arithmetic Mean Intensity Values and Sum of all pixel intensities from each channel were extracted. Averages were then calculated for the CD8 marker.

### 2.9. Statistical Analysis

GraphPad Prism (version 10.2.1, GraphPad Software, San Diego, CA, USA) was used to generate Kaplan–Meier statistics to determine the *p*-value at * *p* < 0.05 for the survival curves and/or the sum fluorescence intensity comparing the immune cells infiltration within the treated cohorts compared to the controls. Non-significant data was denoted as n.s.

## 3. Results

### 3.1. LIFE Biomaterial Providing Image-Guidance During RT

**Figure 3:**
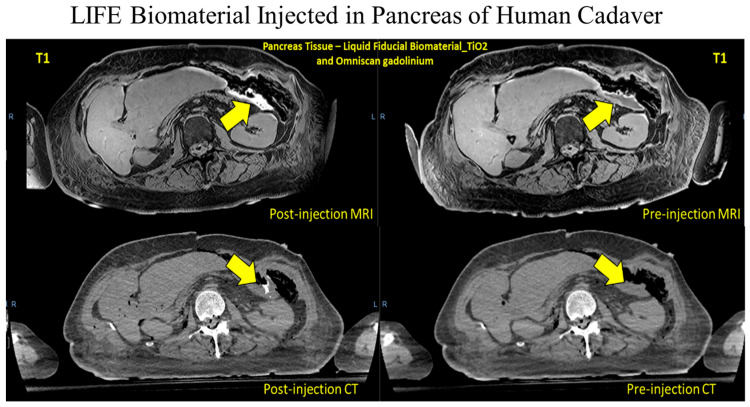
LIFE Biomaterial formulated with gadolinium-based nanoparticles in addition to titanium dioxide nanoparticles and loaded with an anti-CD40 antibody. CT and MRI contrasts were observed from the LIFE Biomaterial injection within the pancreas tissue of the cadaver, as indicated by the yellow arrows.

Smart RT biomaterials, such as LIFE Biomaterial, are made of a biodegradable and biocompatible mixture of natural polymers that undergo gelation within the tumor microenvironment, allowing for a sustained local release of the anti-mouse CD40 monoclonal antibody. A visual depiction of the bimodal imaging from LIFE Biomaterial is displayed in Figure 3 in a human cadaver’s pancreas, showing its potential to provide image-guidance during RT.

**Figure 4:**
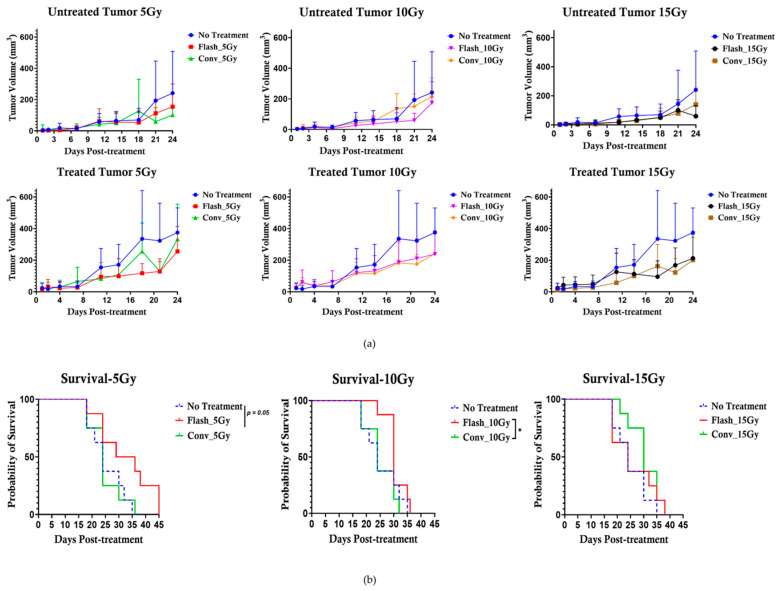
Efficacy of flash compared to conventional RT. (**a**) There was no difference in tumor control between conventional and flash radiotherapy at any of the tested doses from 5 Gy to 15 Gy. (**b**) Prolonged mouse survival was observed for the Flash_5 Gy group compared to Flash_10 Gy and Flash_15 Gy. There was a significant difference (*, *p* < 0.05) at 10 Gy between the survival in the flash group and conventional RT.

This study first investigated whether there is a difference in tumor control between CONV and FLASH RT using a syngeneic subcutaneous pancreatic tumor model. The results showed that there is no apparent difference in generating an anti-tumor effect between CONV and FLASH RT. This is illustrated in Figure 4a, where no significant difference in tumor regression was observed between the control and the treated cohorts exposed to either CONV or FLASH RT.

### 3.2. Effect of FLASH-RT Versus CONV RT on Pancreatic Cancer

The results in Figure 4a showed no significant difference in tumor control between FLASH and CONV irradiation. However, prolonged survival was observed in mice treated with Flash_5 Gy compared to control and Conv_5 Gy, as shown in Figure 4b. Significant survival (*, *p* < 0.05) was observed for mice treated with Flash_10 Gy compared to those treated with CONV_10 Gy.

Based on this initial finding, which identified 5 Gy as a potential optimal dose for FLASH-RT to improve survival, the next study investigated whether FLASH can also elicit an immune response leading to delayed tumor progression compared to control and CONV RT.

### 3.3. Assessment of T-Cell Infiltrations in the Pancreatic Tumor Following the Combination of FLASH-RT and LIFE Biomaterial

#### 3.3.1. Multiplex Immunofluorescence Results

**Figure 5:**
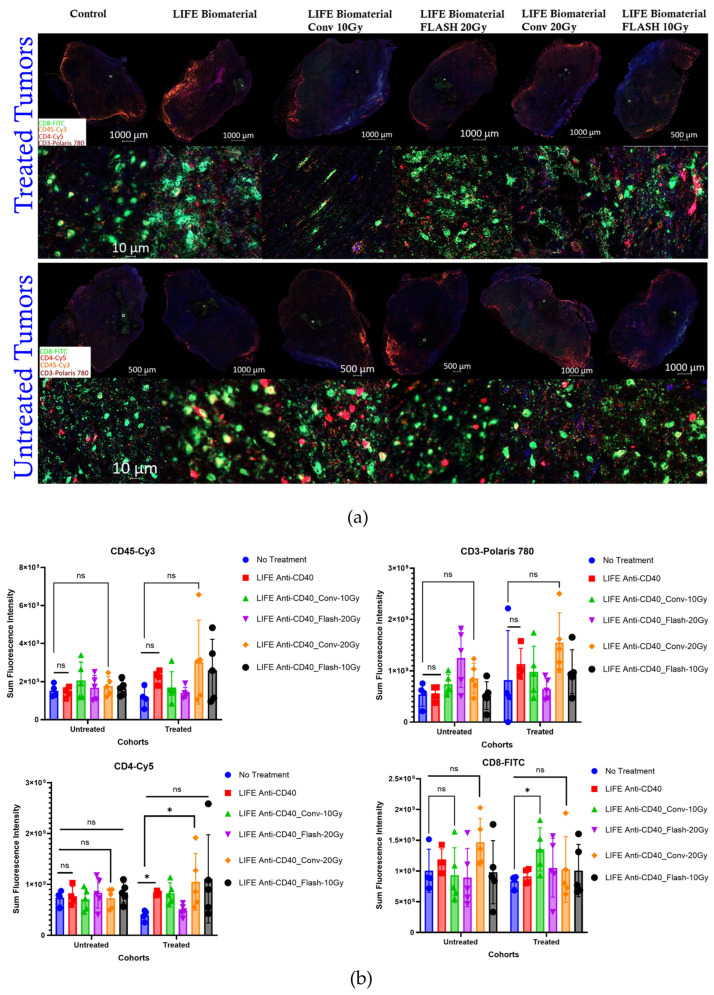
(**a**) Multiplex immunofluorescence images representing the immune cells’ infiltration within the tumor microenvironment. The markers represent the immune cells present in the tumor microenvironment, such as CD8 (FITC) and CD4 (Cy5) T-cells, CD3 (Polaris 780), and CD45 (Cy3). (**b**) Plots derived from immunofluorescence image analysis. The sum fluorescence intensity was analyzed from each tissue sample and plotted to discriminate the amount of immune cell infiltration according to the treatment administered compared to the control cohort. Higher CD4 T-cells infiltrations were only observed for the LIFE Biomaterial_20 µg-Anti-CD40 (*, *p* < 0.05) or those combined with Conv_20 Gy (*, *p* < 0.05) associated with the control group. Subsequently, higher CD8 cytotoxic T-cells (*, *p* < 0.05) were observed in the LIFE Biomaterial_Conv-10 Gy_20 µg-Anti-CD40-treated tumors compared to the no-treatment group.

The Sum Fluorescence Intensity was measured for each immune marker that was stained in the tumor tissues from the multiplex immunofluorescence analysis. Figure 5 showed significantly higher CD4 T-helper cell infiltrations in the tumors treated with either LIFE Biomaterial_20 µg-Anti-CD40 (*, *p* < 0.05) or in combination with Conv-20 Gy (*, *p* < 0.05), in contrast to the control group. However, significantly higher CD8 cytotoxic T-cells (*, *p* < 0.05) were observed for the LIFE Biomaterial_Conv-10 Gy_20 µg-Anti-CD40 treated tumors compared to the control group.

#### 3.3.2. Immunohistochemistry

**Figure 6:**
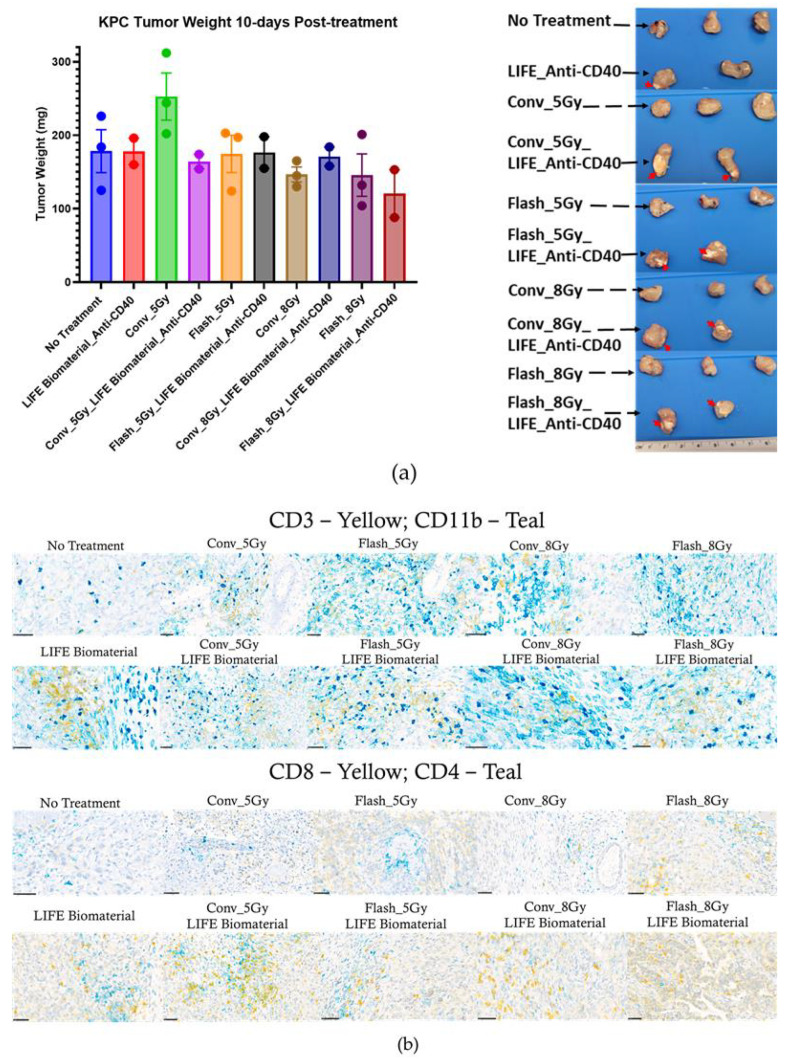

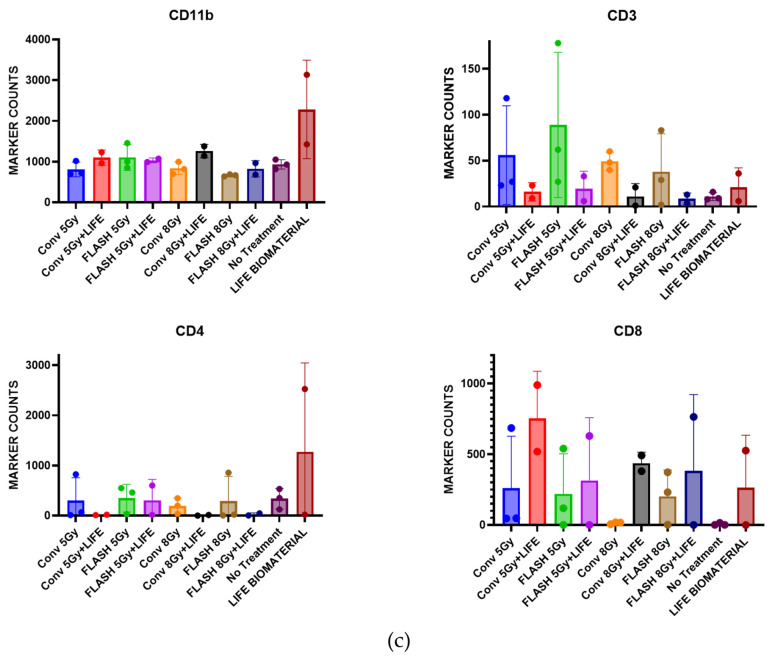
Immunohistochemistry. (**a**) Mice tumors were harvested and weighed 10 days post-treatment, with no significant difference observed between treatment cohorts. The red arrow indicates the presence of LIFE Biomaterial within the tumor tissue. (**b**) Representative immunohistochemistry images stained for CD3 (yellow), CD11b (teal), CD8 (yellow), and CD4 (teal) markers. Scale bar = 50 µm. The LIFE Biomaterial was visualized as a gray color within the tissue, as displayed in the IHC Images. (**c**) Quantification of marker expression was performed using an in-house Python, version 3.12.11 script developed in the Ding lab. Marker counts were plotted to compare the treated cohorts against the control group.

Figure 6a shows that there was no significant difference in the tumor weights of the mice per treatment group 10 days post-treatment. Although in Figure 6b,c, higher amounts of infiltrated CD11b and CD4+ T-cells were observed for the LIFE Biomaterial_anti-CD40 group compared to other treated and untreated cohorts. However, the Flash_5 Gy cohort showed a higher infiltration of CD3 compared to all other cohorts. Conv_5 Gy_LIFE Biomaterial_anti-CD40 showed higher CD8+ cytotoxic T-cell infiltration compared to all other cohorts.

The initial results of mice survival revealed Flash_5 Gy could be optimal in prolonging mice’s existence compared to no treatment or CONV at 10 Gy or 15 Gy groups. As summarized in Table 1, T-cell infiltrations in the pancreatic tumors treated with LIFE Biomaterial_anti-CD40 alone or combined with CONV_5 Gy, CONV_10 Gy, CONV_20 Gy, or Flash_5 Gy alone, respectively, have shown higher permeations of CD11b or CD3, CD4, and CD8 T-cells in the tumor microenvironment.

## 4. Discussion

The FLASH effect has attracted considerable research interest due to its unique ability to spare normal tissue while effectively targeting cancerous cells. Nevertheless, the precise parameters required to induce this effect are tissue-specific and remain broadly uncertain. Pre-clinical studies have increasingly explored strategies to enhance the therapeutic efficacy of FLASH-RT by combining it with other therapies, such as immunotherapy and chemotherapy, across various tumor types. In this study, we investigated the efficacy of FLASH-RT combined with LIFE Biomaterial loaded with anti-CD40 antibody, compared with CONV-RT delivered under similar conditions. The results demonstrate that FLASH-RT delivered at 5 Gy significantly prolonged mouse survival compared to the unirradiated controls and cohorts treated with higher doses (10 Gy or 15 Gy) using either FLASH or CONV dose rate. These findings suggest that FLASH-RT at an optimized dose may enhance survival outcomes when integrated with immune-modulating strategies such as LIFE Biomaterial_anti-CD40.

The combination of immunotherapy with either FLASH-RT or CONV-RT allows for immunophenotyping of infiltrating lymphocytes into the tumor microenvironment, helping to identify conditions that optimize the anti-tumor immune response. Fundamentally, the immune system plays a key role in normal tissue toxicity and tumor control following FLASH-RT [28]. Therefore, investigating immune cell responses at ultra-high dose rates is a momentous part of research [19,29]. The multiplex immunofluorescence analysis revealed higher CD3 infiltration in the tumors treated with LIFE Biomaterial_anti-CD40_Conv-20 Gy compared to the other cohorts. Significant infiltrations of CD4 were observed in tumors treated with either LIFE Biomaterial_anti-CD40 or LIFE Biomaterial_anti-CD40_Conv-20 Gy relative to the other groups. Cytotoxic T-cells significantly infiltrated the LIFE Biomaterial_anti-CD40_Conv-10 Gy tumors compared to the other groups.

An immunohistochemistry analysis of tumors harvested 10 days of post-treatment confirmed lymphocyte recruitment in the tumor microenvironment. Higher levels of CD11b antigen-presenting cells were observed in all the treated tumors compared with the no-treatment group. Elevated levels of CD3 were observed in the Flash-5 Gy group compared to the others. Increased CD4 T-cells were found in the LIFE Biomaterial_anti-CD40, while higher CD8 T-cells were observed in the LIFE Biomaterial_anti-CD40_Conv-5 Gy and all other treated cohorts compared to Conv-8 Gy and no treatment controls.

The limitations of this study include the small number of mice used to investigate the efficacy of FLASH-RT in eliciting the FLASH effect. A finer dose resolution between 5 Gy and 10 Gy doses evaluated in this study could better determine the optimal FLASH dose for achieving anti-tumor effect. Future studies should investigate immune responses at earlier time points (e.g., 7- and 14-day treatment) to determine if these responses are time-dependent. Future studies can focus on characterizing LIFE Biomaterial, to ensure its safety and efficiency pre-clinically. Overall, these results highlight the need for further investigation to identify optimal conditions that effectively elicit the FLASH effect, producing anti-tumor responses while sparing normal tissues from radiation-related toxicities.

Overall, this preliminary study has determined Flash-5 Gy to be the optimal dose for combining with LIFE Biomaterial loaded with anti-CD40 to generate an anti-tumor effect in prolonging the overall survival of the mice. It was observed that the immune system was leveraged by combining RT and LIFE Biomaterial loaded with anti-CD40, which could be beneficial for cancer patients.

## 5. Conclusions

In summary, this preliminary study on leveraging the immune system response to a combination treatment of LIFE Biomaterial_anti-CD40 and FLASH-RT showed the potential recruitment of the T-lymphocytes in the tumor microenvironment. Future studies will focus on optimizing the dose and dose rate for FLASH-RT in combination with LIFE Biomaterial loaded with immunotherapeutics to enhance the FLASH effect for higher therapeutic efficiency of cold tumors like pancreatic cancer.

## Figures and Tables

**Table 1:** This table highlights the efficacy of treatment groups compared to the control.

Treatment	Multiplex Immunofluorescence ^1^
LIFE Biomaterial_ 20 µg-Anti-CD40	↑ CD4+ (*p* < 0.05)
LIFE Biomaterial_20 µg -Anti-CD40_Conv_10 Gy	↑ CD8+ (*p* < 0.05)
LIFE Biomaterial_20 µg -Anti-CD40_Conv_20 Gy	↑ CD4+ (*p* < 0.05)
**Treatment**	**Immunohistochemistry** ^2^
LIFE Biomaterial_ 100 µg-Anti-CD40	↑ >2 fold increase CD11b
LIFE Biomaterial_ 100 µg-Anti-CD40	↑ >3.5 fold increase CD4
LIFE Biomaterial_100 µg -Anti-CD40_CONV 5 Gy	↑ >50 fold increase CD8
Flash_5 Gy	↑ >11 fold increase CD3

^1^ This table lists the significant increase in T cells in the treated cohorts in comparison to the control group as represented by the upward arrows. ^2^ The treated groups with the highest fold increase in immune cells (as represented by the upward arrows) compared to the no-treatment group are listed in this table.

## Data Availability

Data is available within this manuscript and in the Supplementary Materials. Further requests can be addressed to the corresponding authors.

## Supplementary Materials

The following supporting information can be downloaded at https://www.mdpi.com/article/10.3390/pharmaceutics17101273/s1. IHC Images; IHC folder has two distinct folders: CD3-CD11b and CD4-CD8.
