# Supplementary figures and images for: Leveraging the Immune Response from LIFE Biomaterial and Photon-Flash in Pre-Clinical Pancreatic Cancer Treatment (part 2 of 2)
Source: Pharmaceutics. 2025 Sep 29;17(10):1273. doi: 10.3390/pharmaceutics17101273 (PMC12566649; doi:10.3390/pharmaceutics17101273)

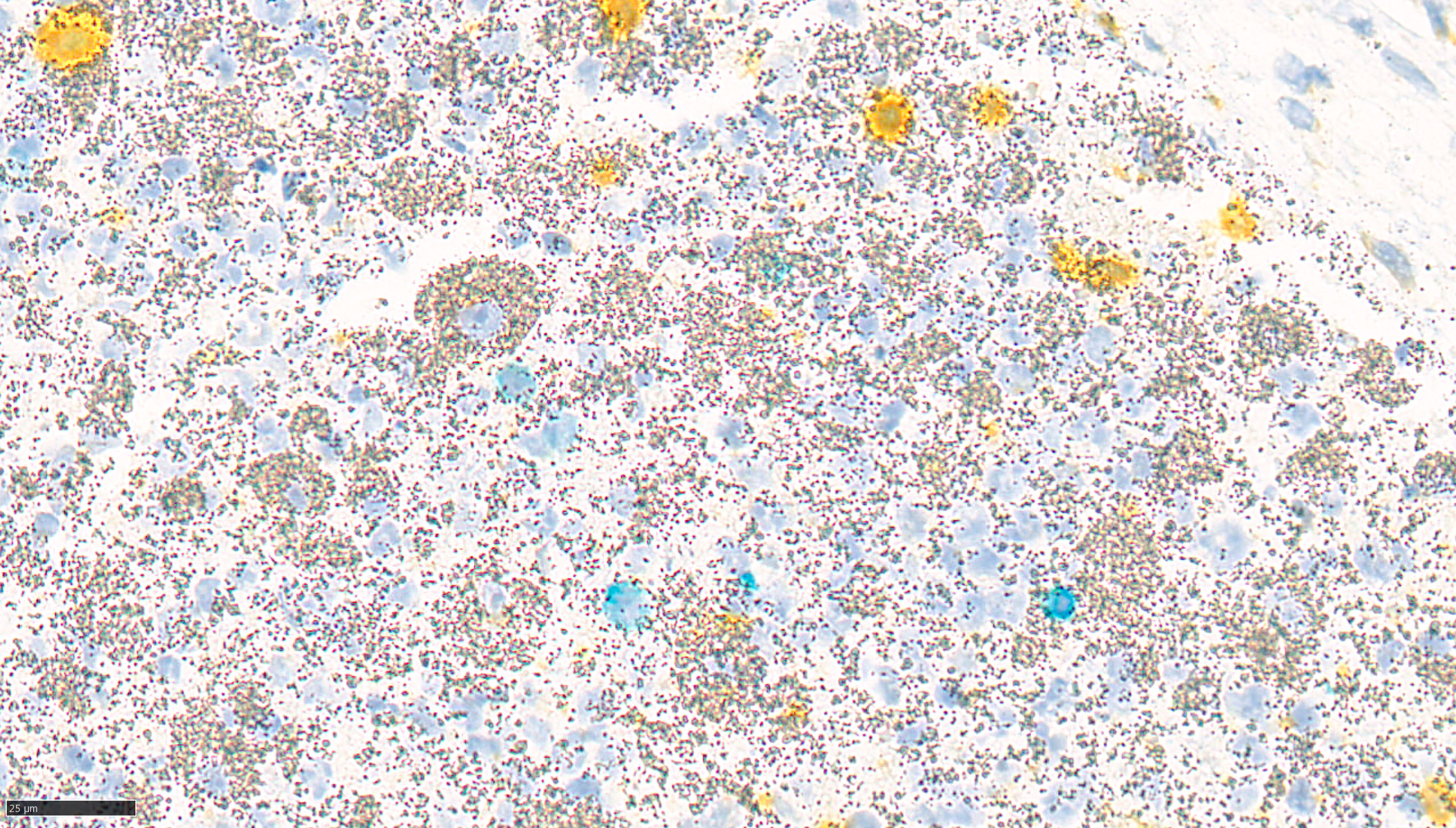

Supplement: Supplementary file 1 [file pharmaceutics-17-01273-s001.zip › IHC/CD4-CD8/LIFE BIOMATERIAL_FLASH 5Gy/F5-L1/F5-L1-2.jpg]

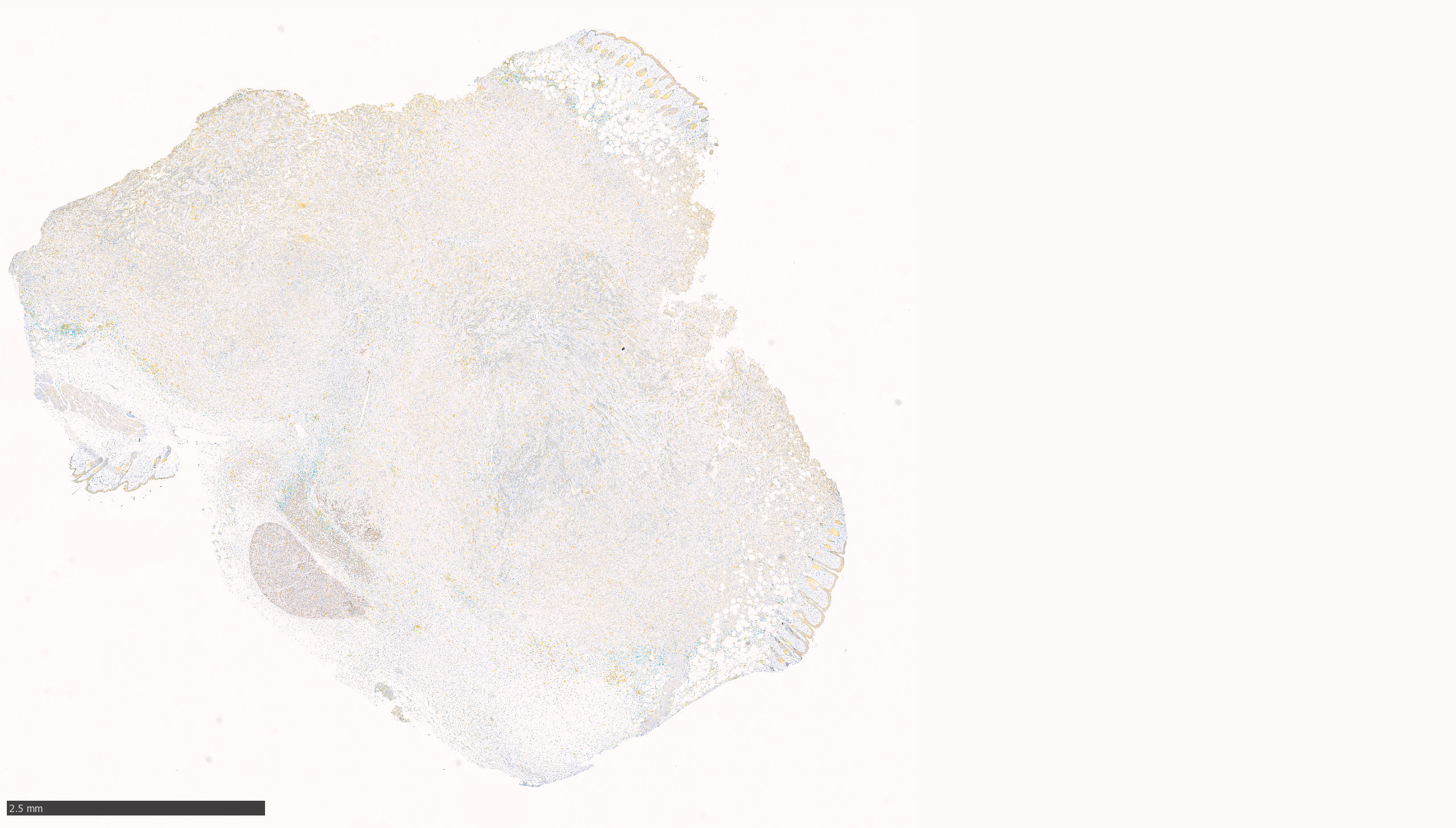

Supplement: Supplementary file 1 [file pharmaceutics-17-01273-s001.zip › IHC/CD4-CD8/LIFE BIOMATERIAL_FLASH 5Gy/F5-L1/F5-L1.jpg]

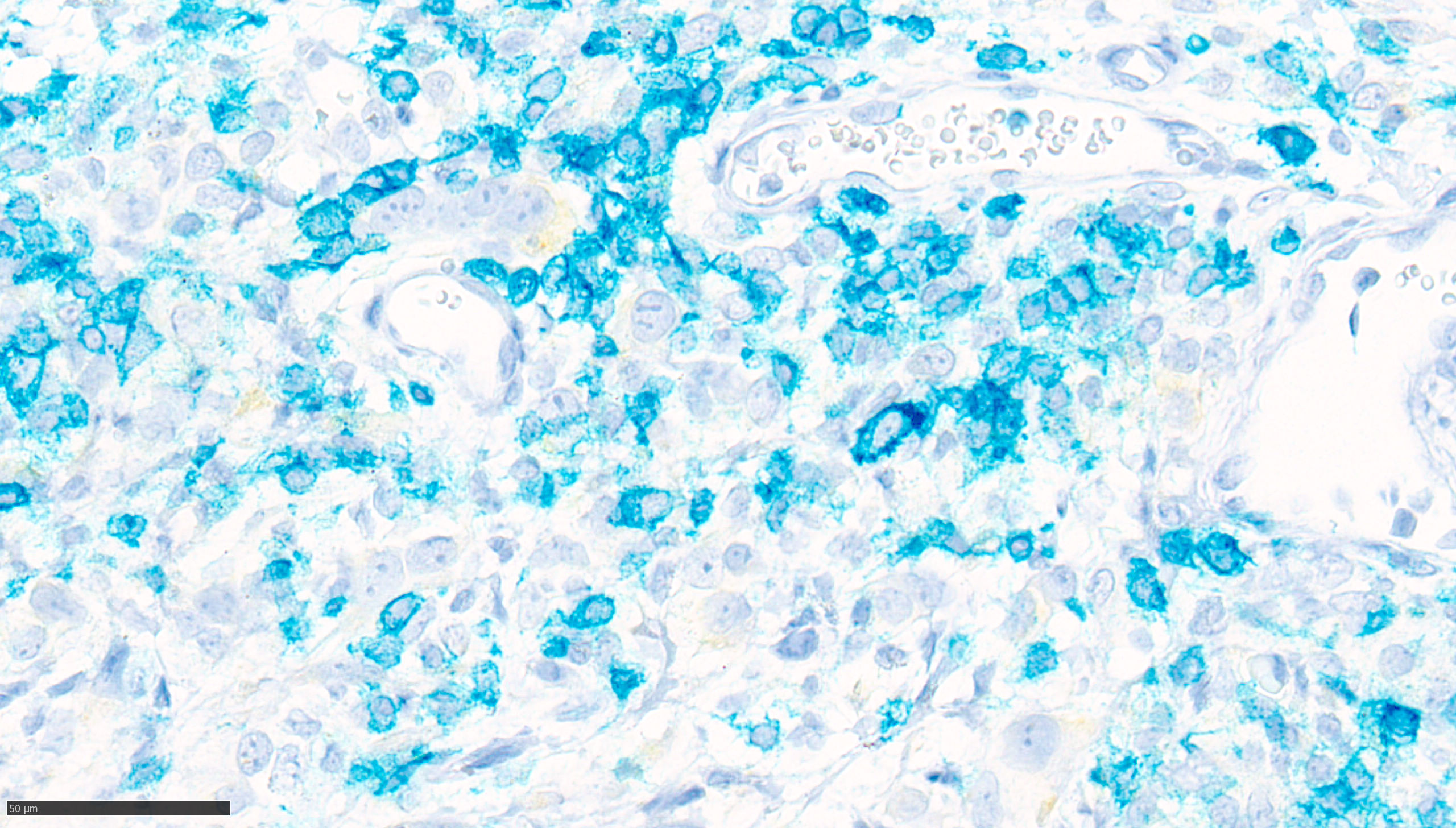

Supplement: Supplementary file 1 [file pharmaceutics-17-01273-s001.zip › IHC/CD4-CD8/LIFE BIOMATERIAL_FLASH 5Gy/F5-L2/F5-L2-1.jpg]

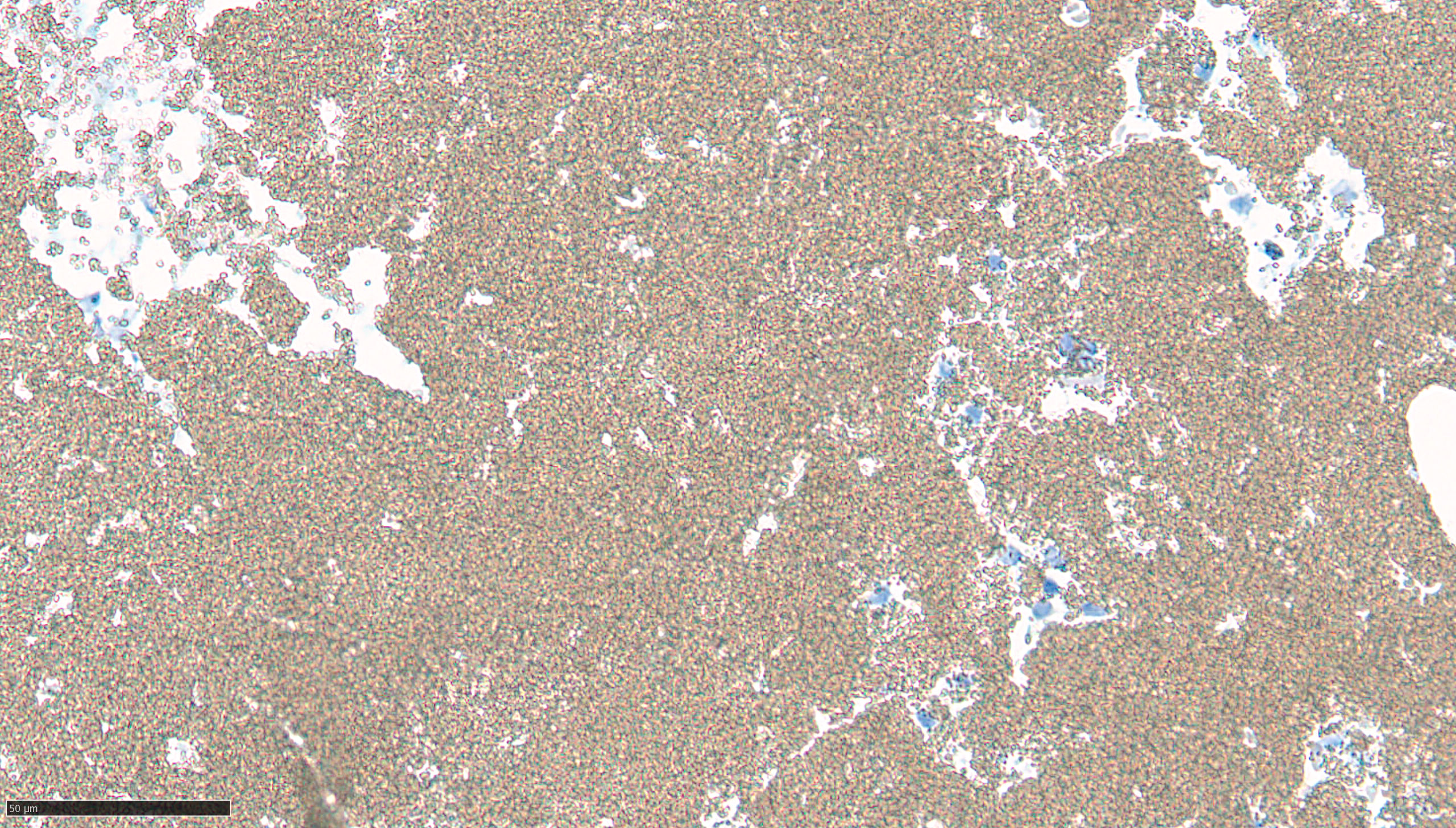

Supplement: Supplementary file 1 [file pharmaceutics-17-01273-s001.zip › IHC/CD4-CD8/LIFE BIOMATERIAL_FLASH 5Gy/F5-L2/F5-L2-2.jpg]

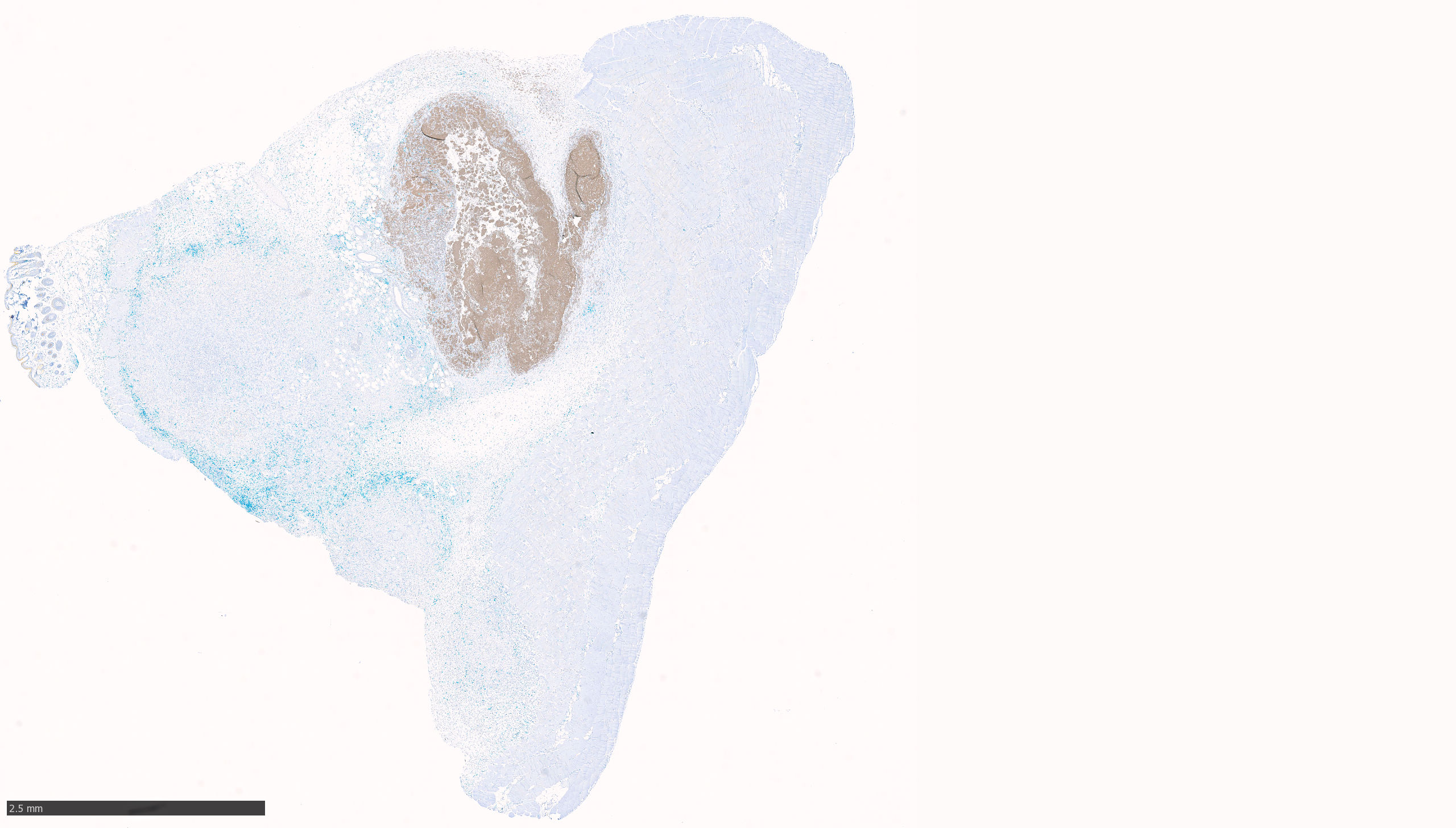

Supplement: Supplementary file 1 [file pharmaceutics-17-01273-s001.zip › IHC/CD4-CD8/LIFE BIOMATERIAL_FLASH 5Gy/F5-L2/F5-L2.jpg]

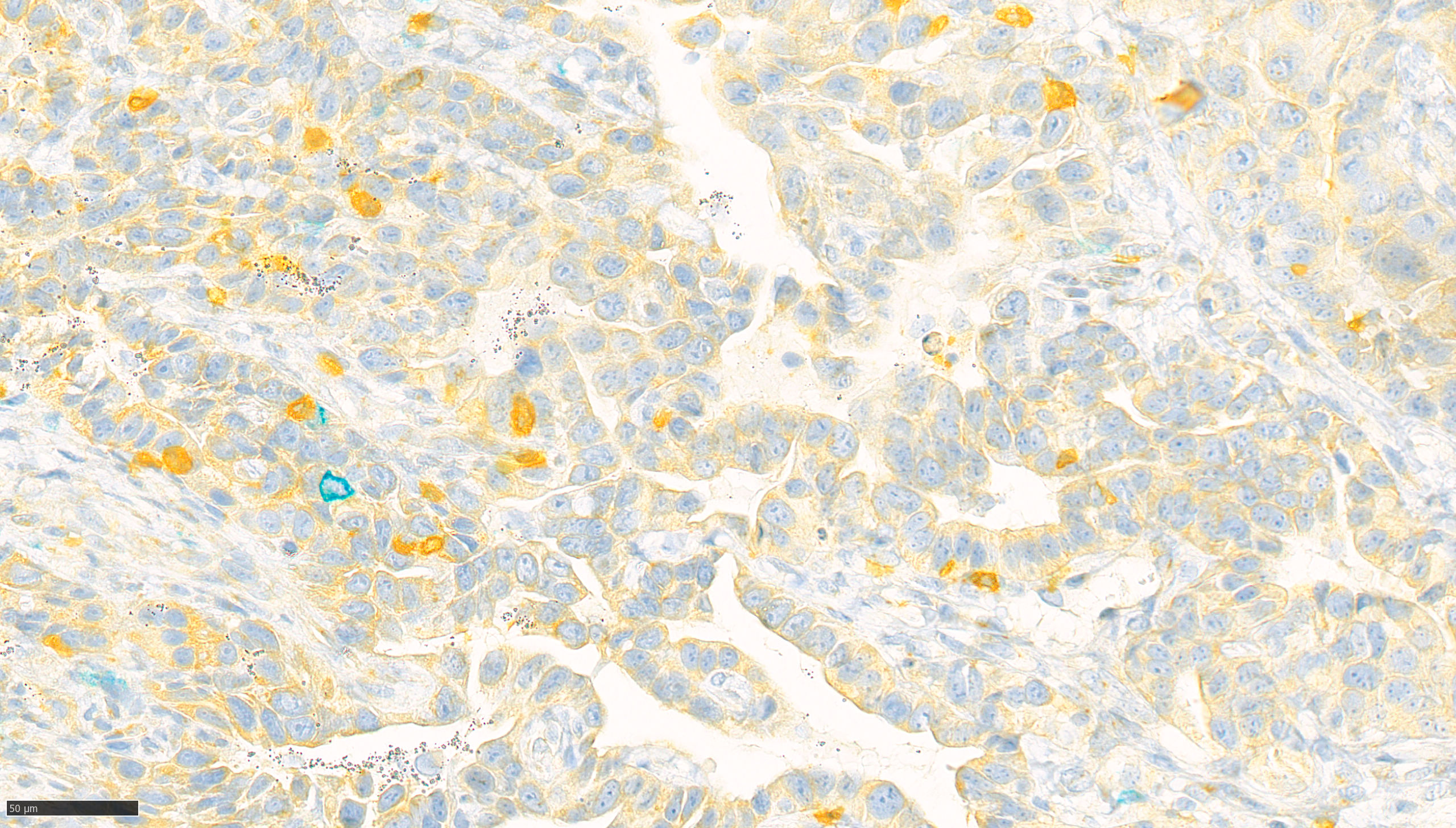

Supplement: Supplementary file 1 [file pharmaceutics-17-01273-s001.zip › IHC/CD4-CD8/LIFE BIOMATERIAL_FLASH-8Gy/F8-L1/F8-L1-1.jpg]

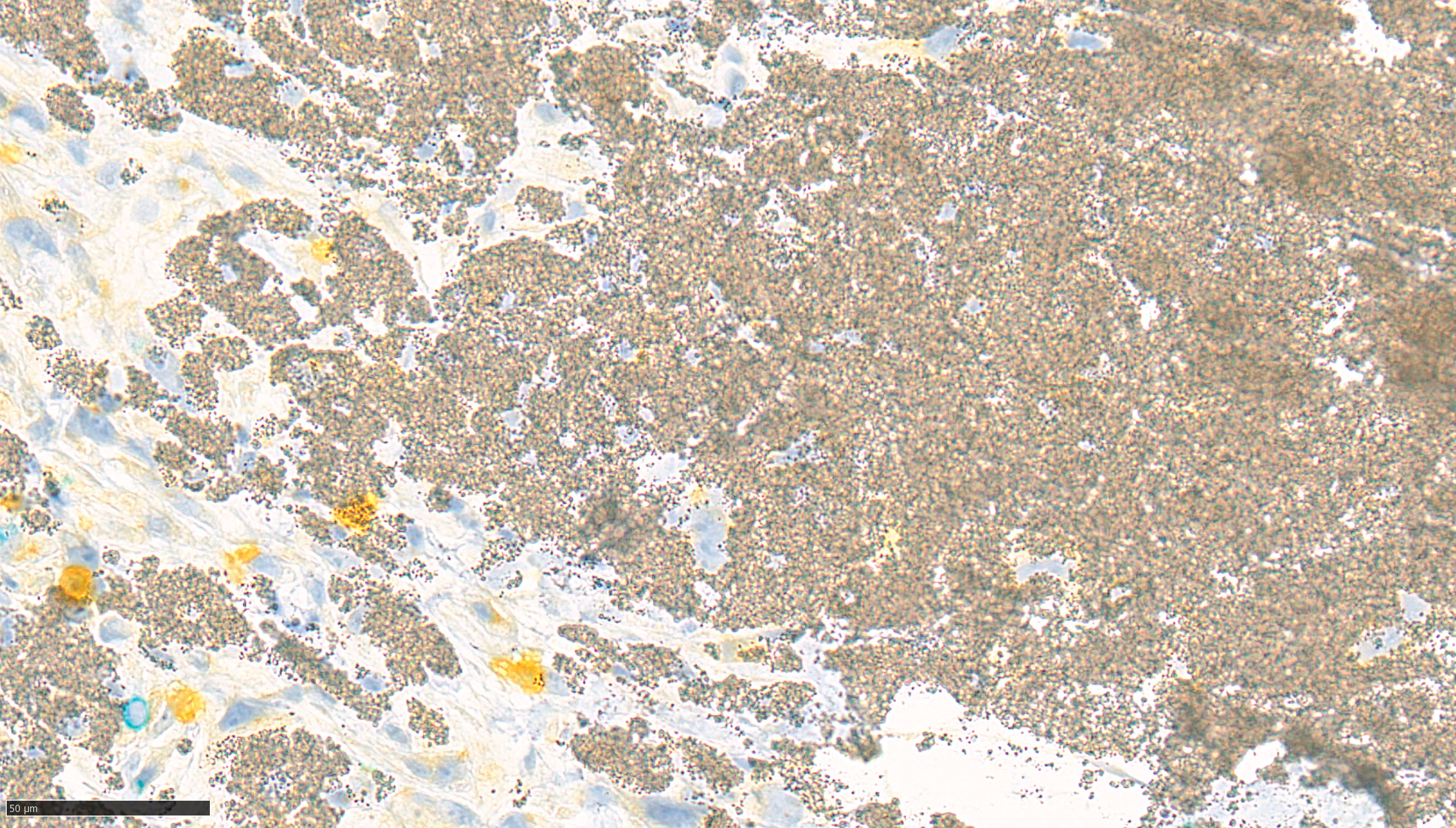

Supplement: Supplementary file 1 [file pharmaceutics-17-01273-s001.zip › IHC/CD4-CD8/LIFE BIOMATERIAL_FLASH-8Gy/F8-L1/F8-L1-2.jpg]

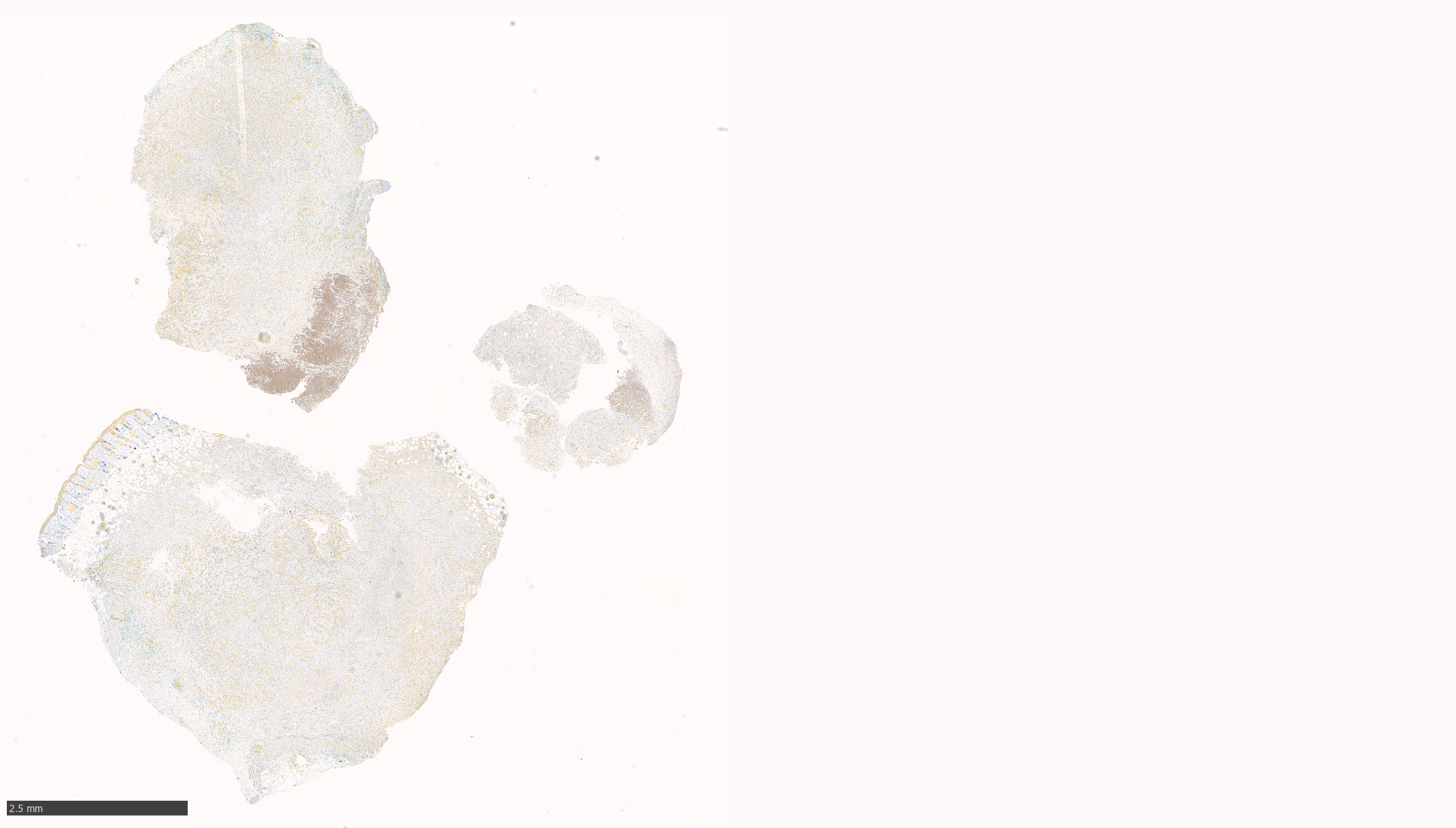

Supplement: Supplementary file 1 [file pharmaceutics-17-01273-s001.zip › IHC/CD4-CD8/LIFE BIOMATERIAL_FLASH-8Gy/F8-L1/F8-L1.jpg]

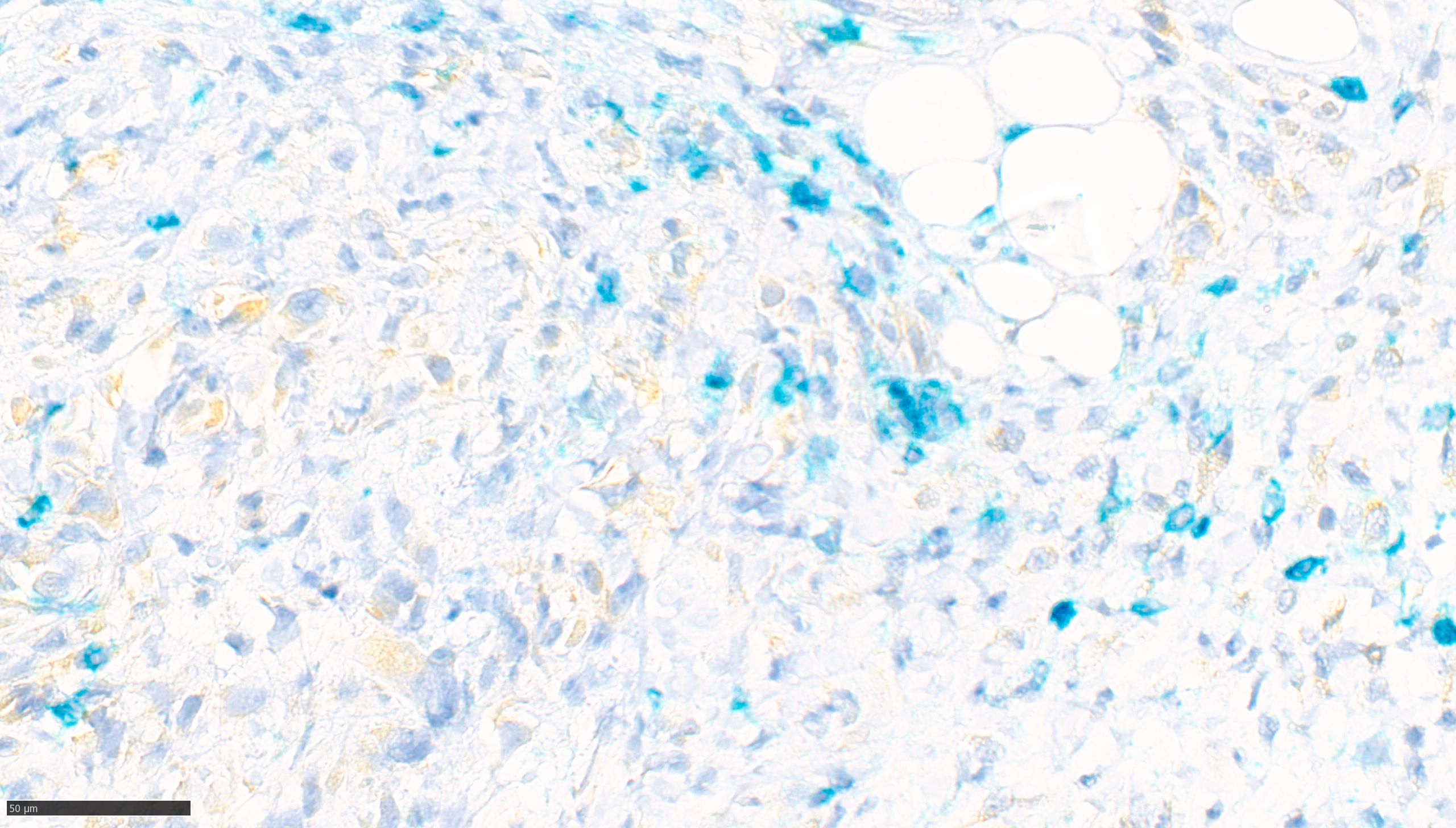

Supplement: Supplementary file 1 [file pharmaceutics-17-01273-s001.zip › IHC/CD4-CD8/LIFE BIOMATERIAL_FLASH-8Gy/F8-L2/F8-L2-1.jpg]

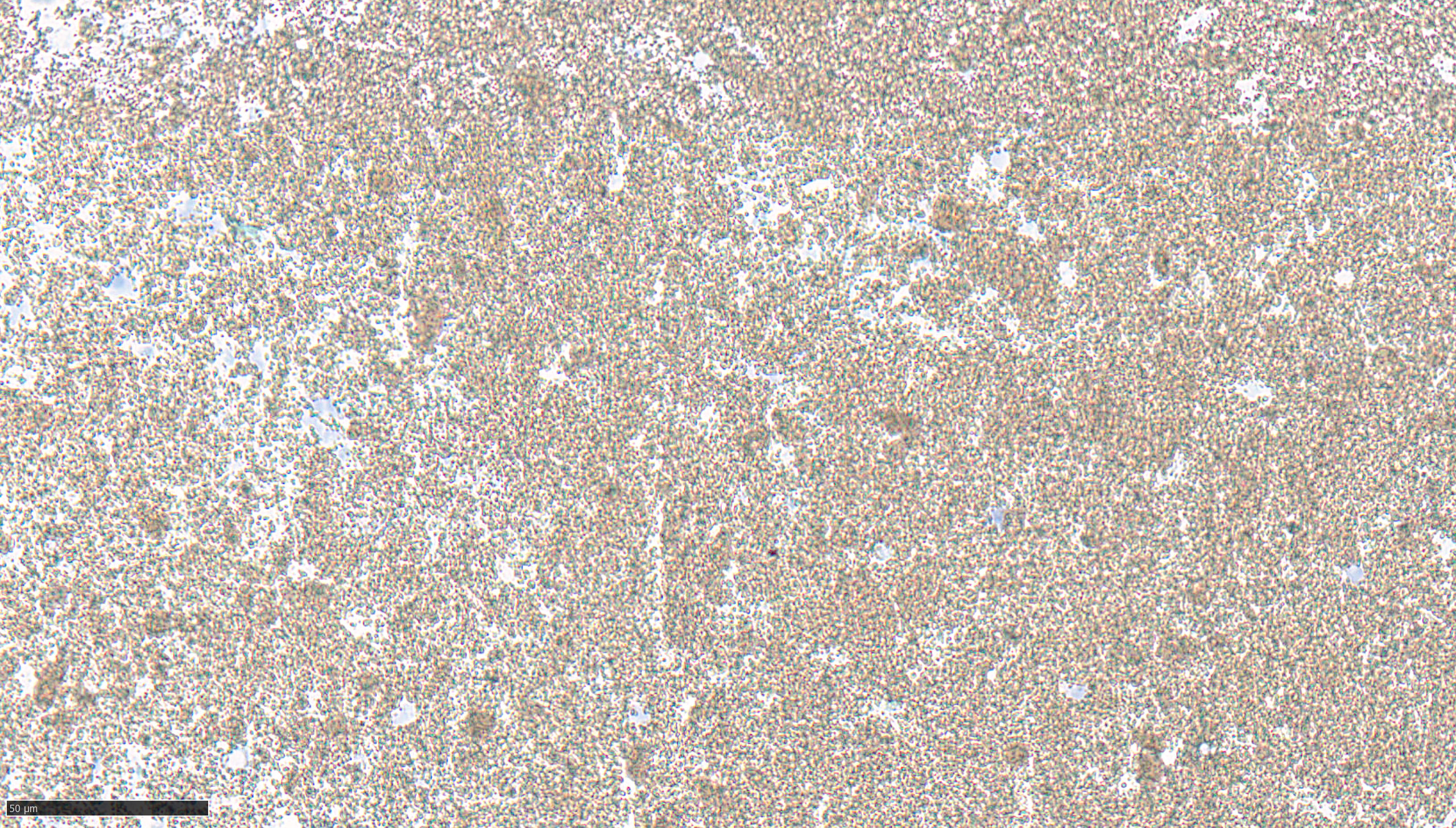

Supplement: Supplementary file 1 [file pharmaceutics-17-01273-s001.zip › IHC/CD4-CD8/LIFE BIOMATERIAL_FLASH-8Gy/F8-L2/F8-L2-2.jpg]

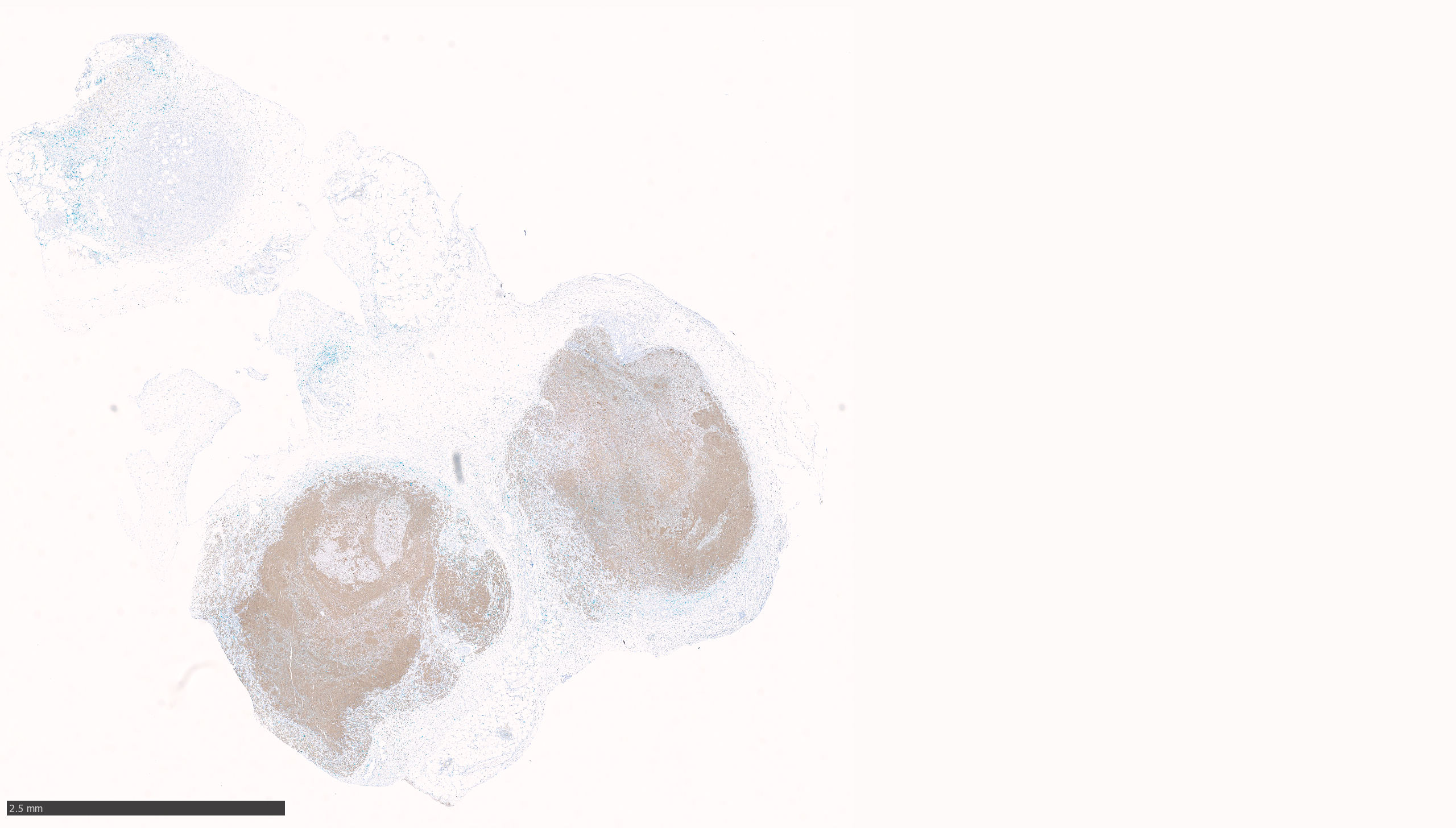

Supplement: Supplementary file 1 [file pharmaceutics-17-01273-s001.zip › IHC/CD4-CD8/LIFE BIOMATERIAL_FLASH-8Gy/F8-L2/F8-L2.jpg]

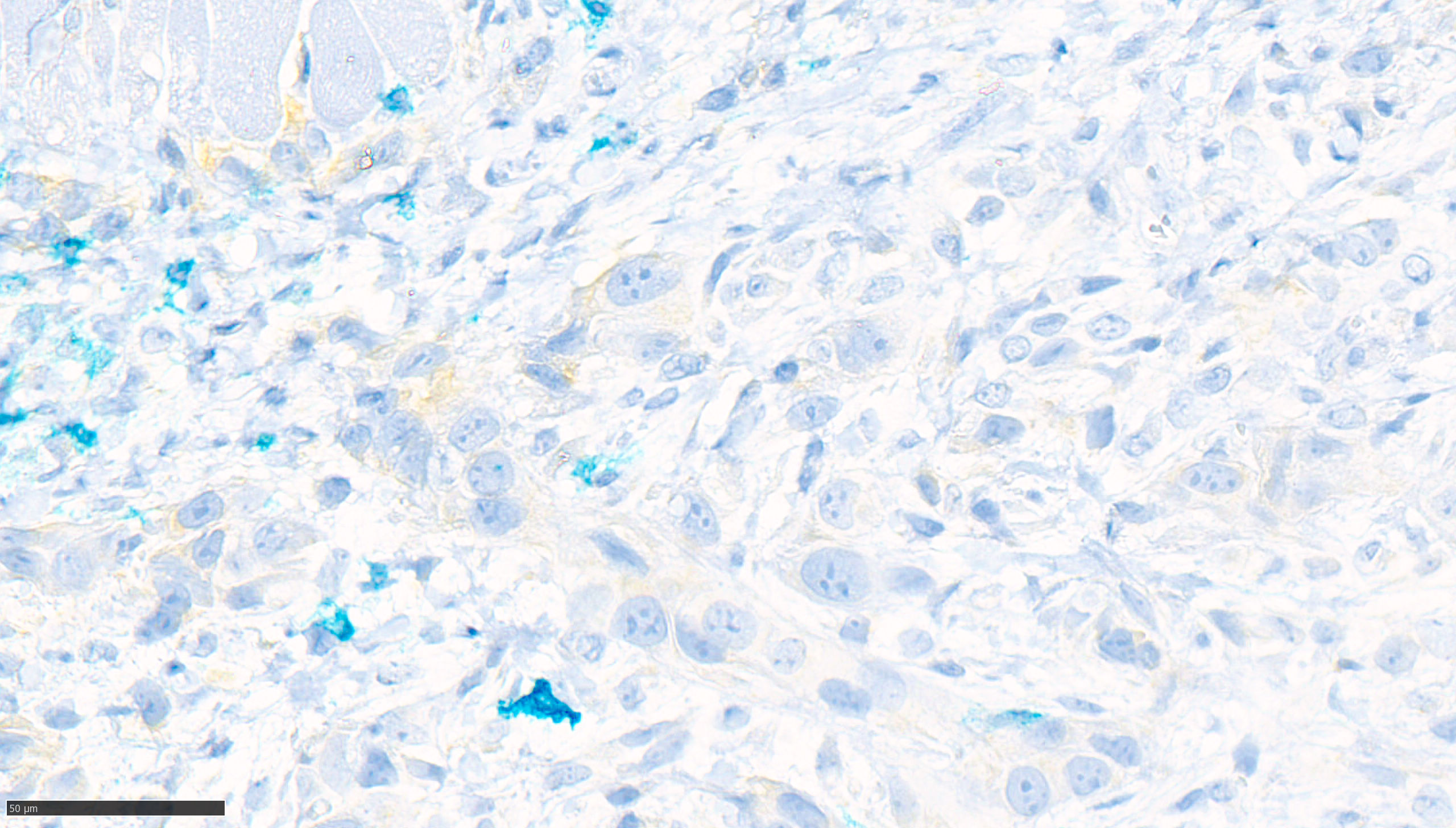

Supplement: Supplementary file 1 [file pharmaceutics-17-01273-s001.zip › IHC/CD4-CD8/NO TREATMENT/N1/N1-1.jpg]

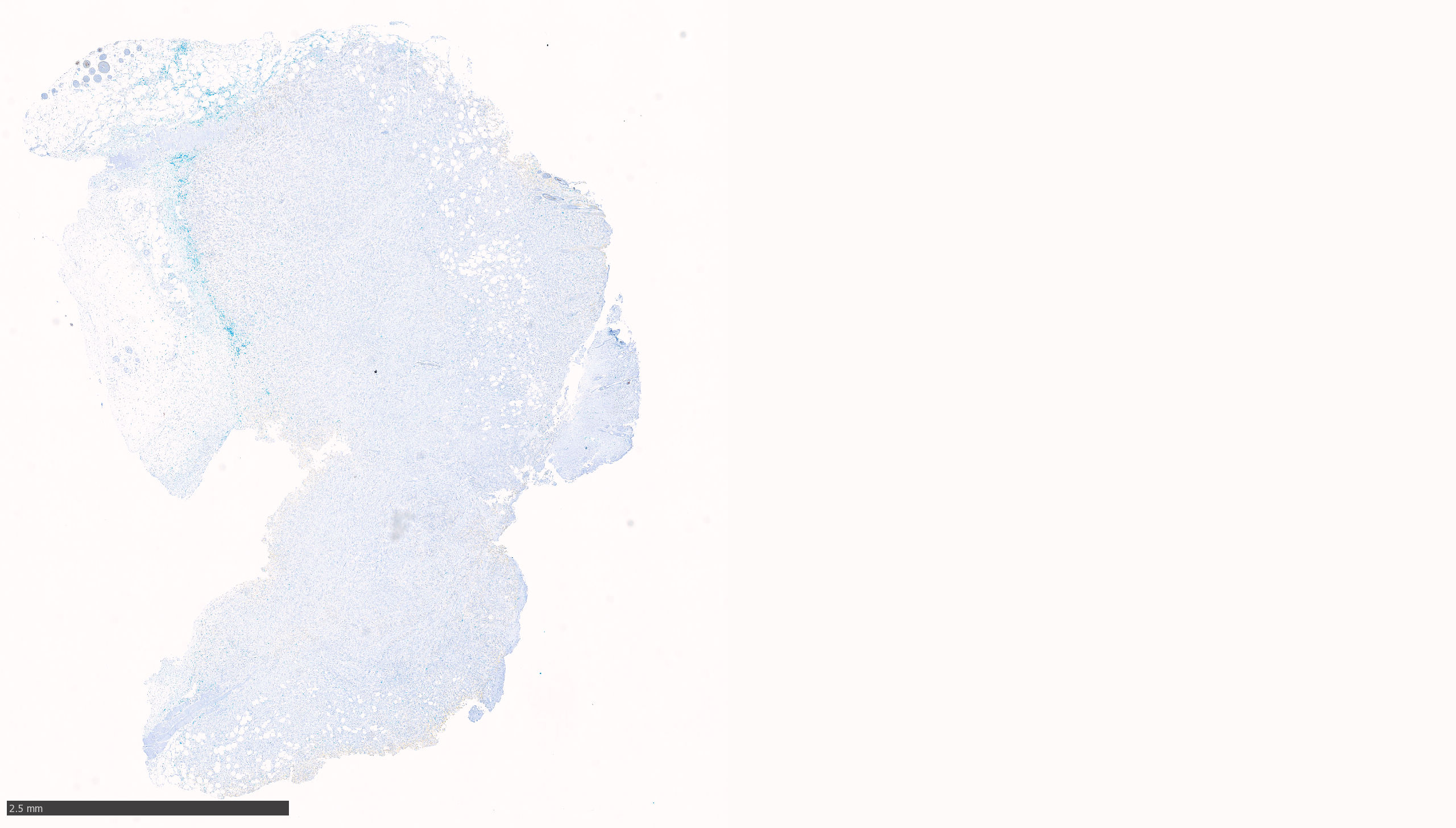

Supplement: Supplementary file 1 [file pharmaceutics-17-01273-s001.zip › IHC/CD4-CD8/NO TREATMENT/N1/N1.jpg]

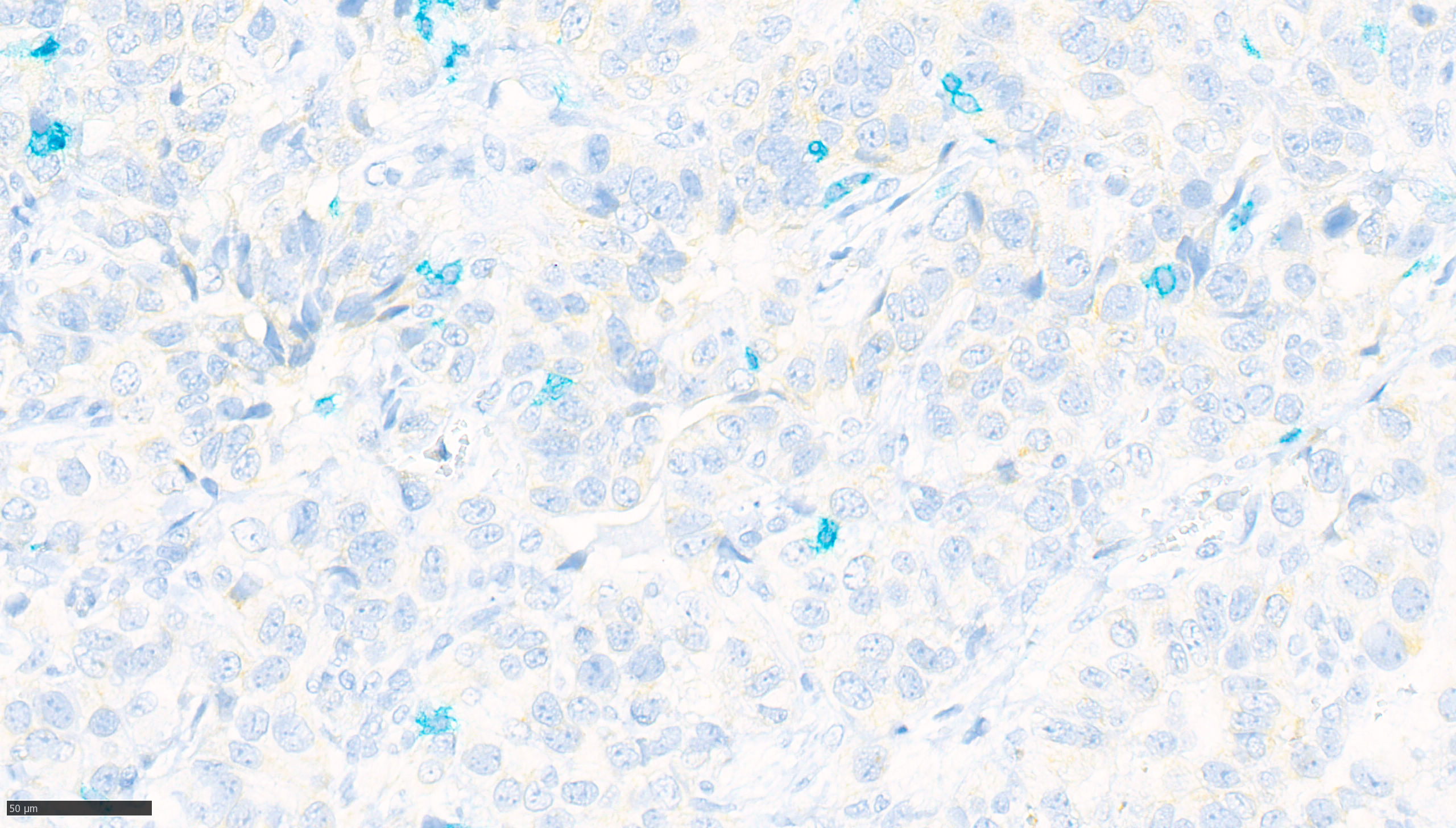

Supplement: Supplementary file 1 [file pharmaceutics-17-01273-s001.zip › IHC/CD4-CD8/NO TREATMENT/N2/N2-1.jpg]

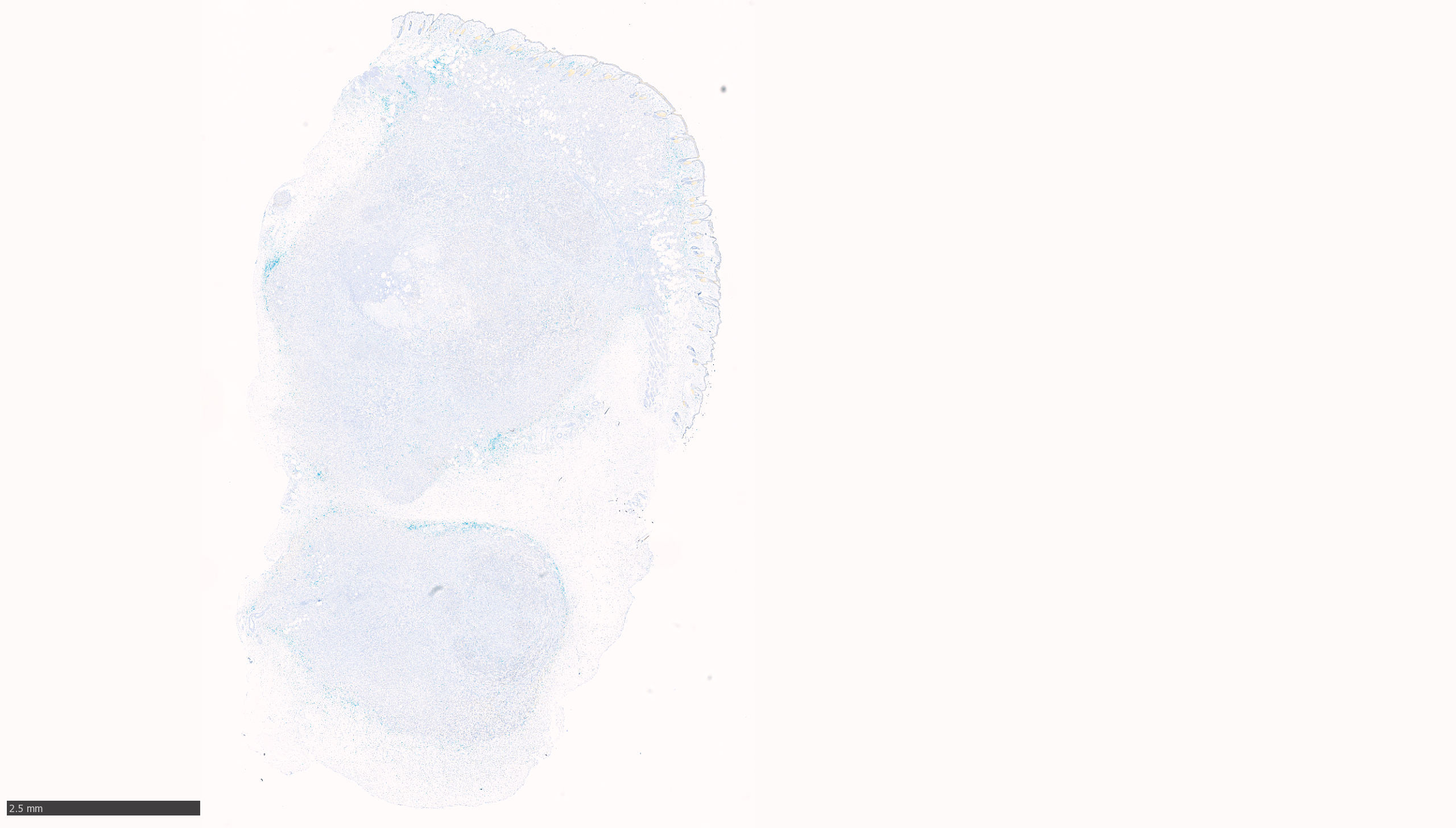

Supplement: Supplementary file 1 [file pharmaceutics-17-01273-s001.zip › IHC/CD4-CD8/NO TREATMENT/N2/N2.jpg]

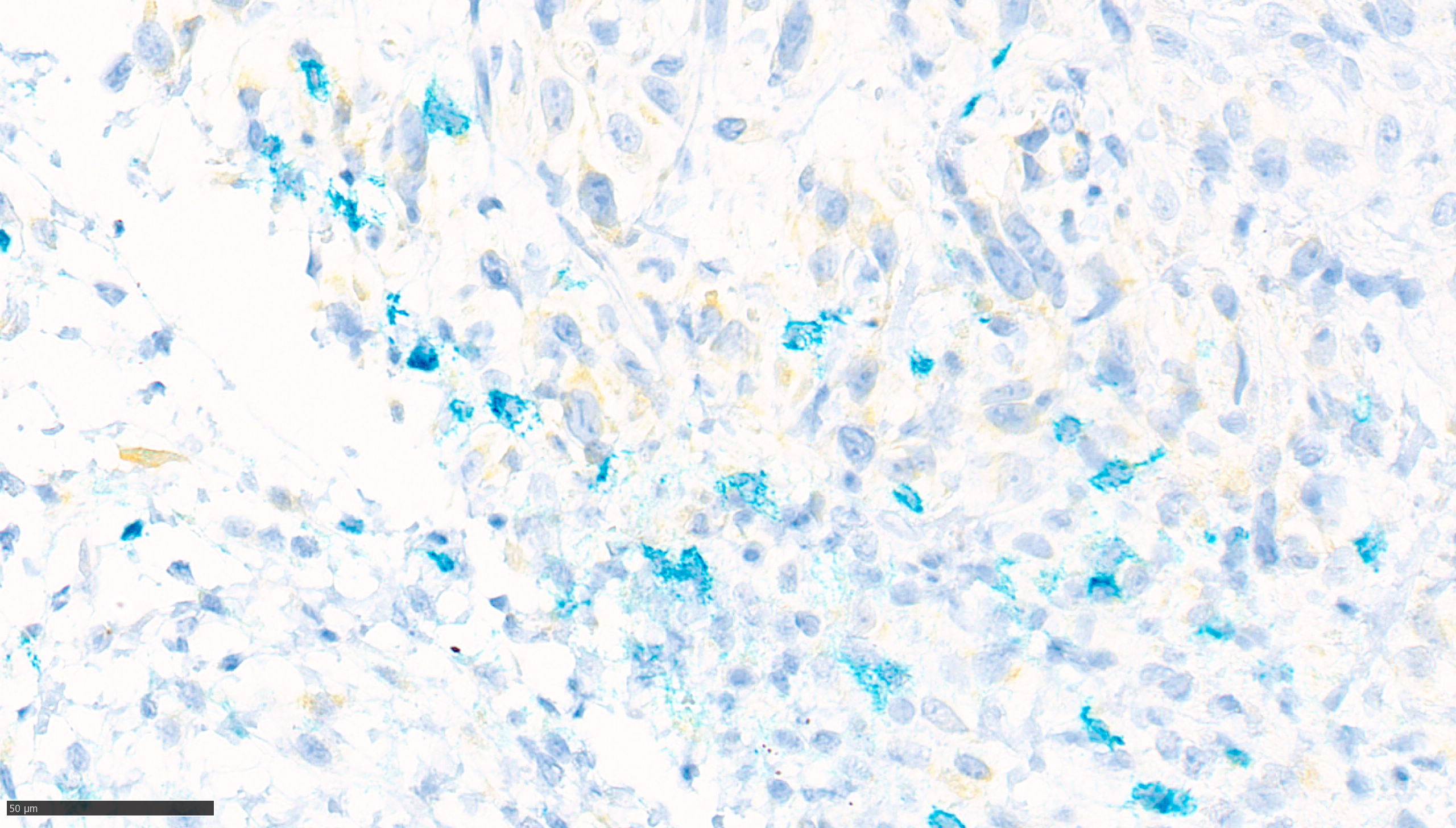

Supplement: Supplementary file 1 [file pharmaceutics-17-01273-s001.zip › IHC/CD4-CD8/NO TREATMENT/N3/N3-1.jpg]

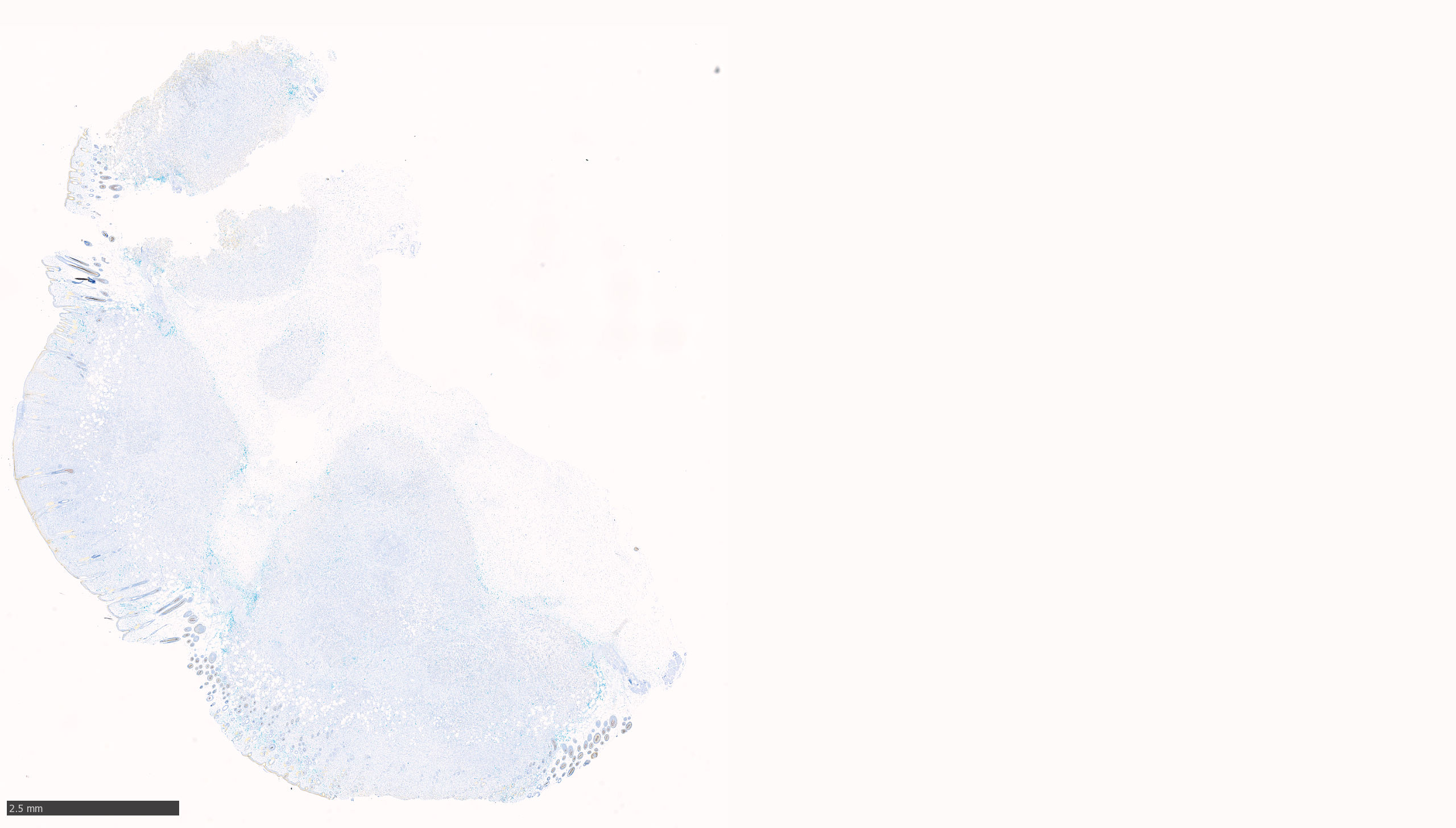

Supplement: Supplementary file 1 [file pharmaceutics-17-01273-s001.zip › IHC/CD4-CD8/NO TREATMENT/N3/N3.jpg]
