# Supplementary figures and images for: Leveraging the Immune Response from LIFE Biomaterial and Photon-Flash in Pre-Clinical Pancreatic Cancer Treatment (part 1 of 2)
Source: Pharmaceutics. 2025 Sep 29;17(10):1273. doi: 10.3390/pharmaceutics17101273 (PMC12566649; doi:10.3390/pharmaceutics17101273)

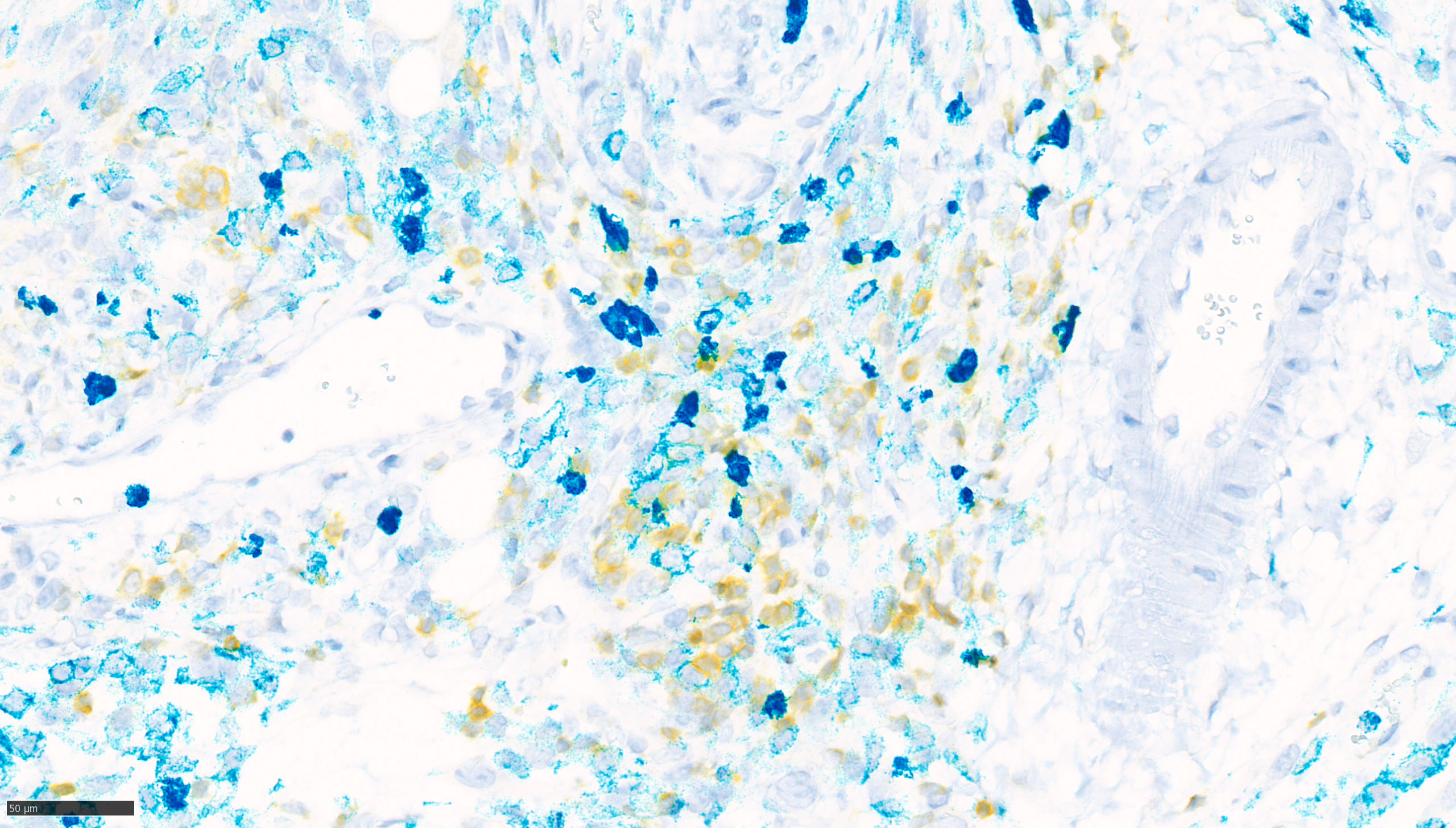

Supplement: Supplementary file 1 [file pharmaceutics-17-01273-s001.zip › IHC/CD3-CD11B/CONV-5Gy/C5-1/C5-1-1.jpg]

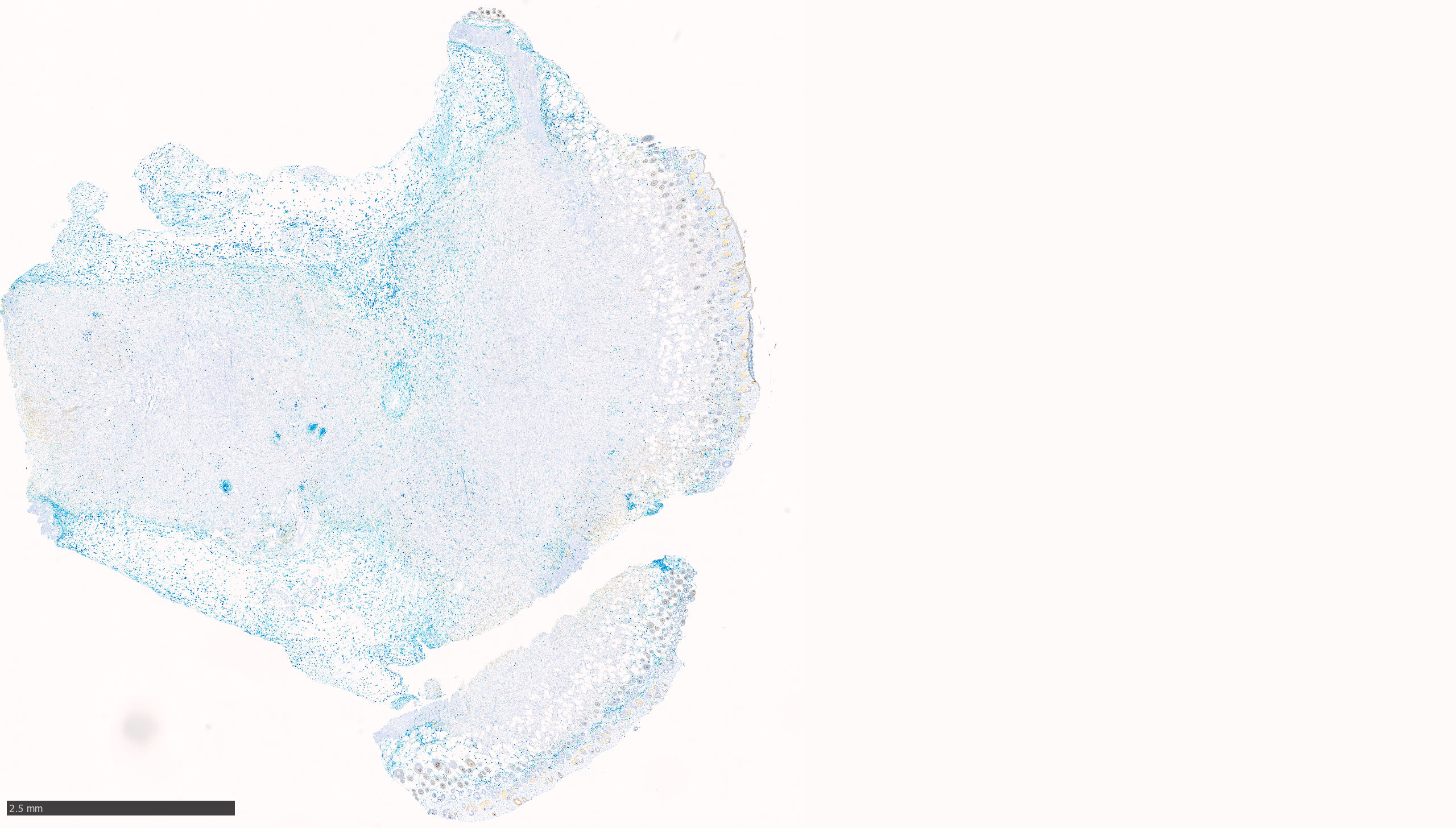

Supplement: Supplementary file 1 [file pharmaceutics-17-01273-s001.zip › IHC/CD3-CD11B/CONV-5Gy/C5-1/C5-1.jpg]

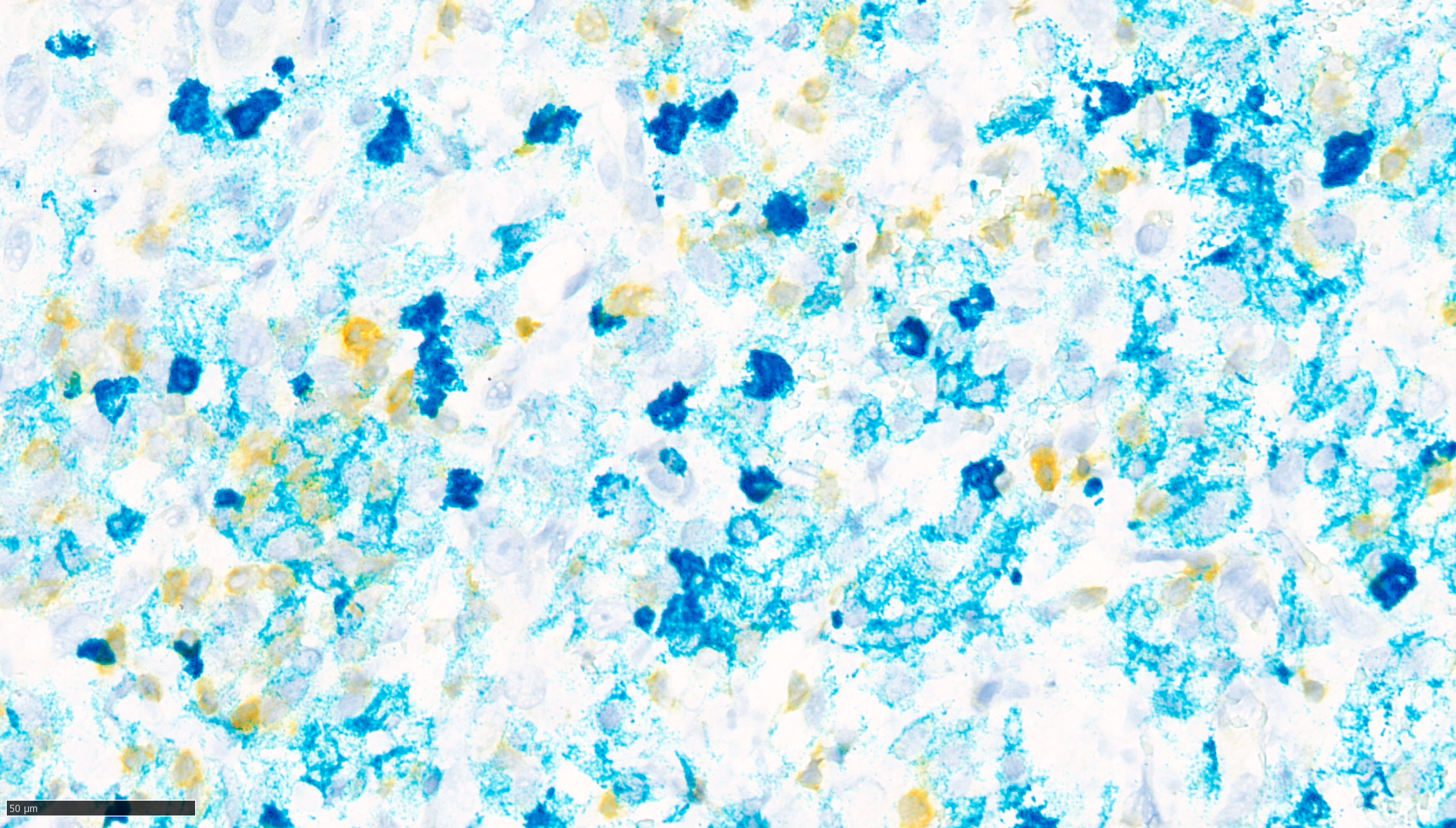

Supplement: Supplementary file 1 [file pharmaceutics-17-01273-s001.zip › IHC/CD3-CD11B/CONV-5Gy/C5-2/C5-2-1.jpg]

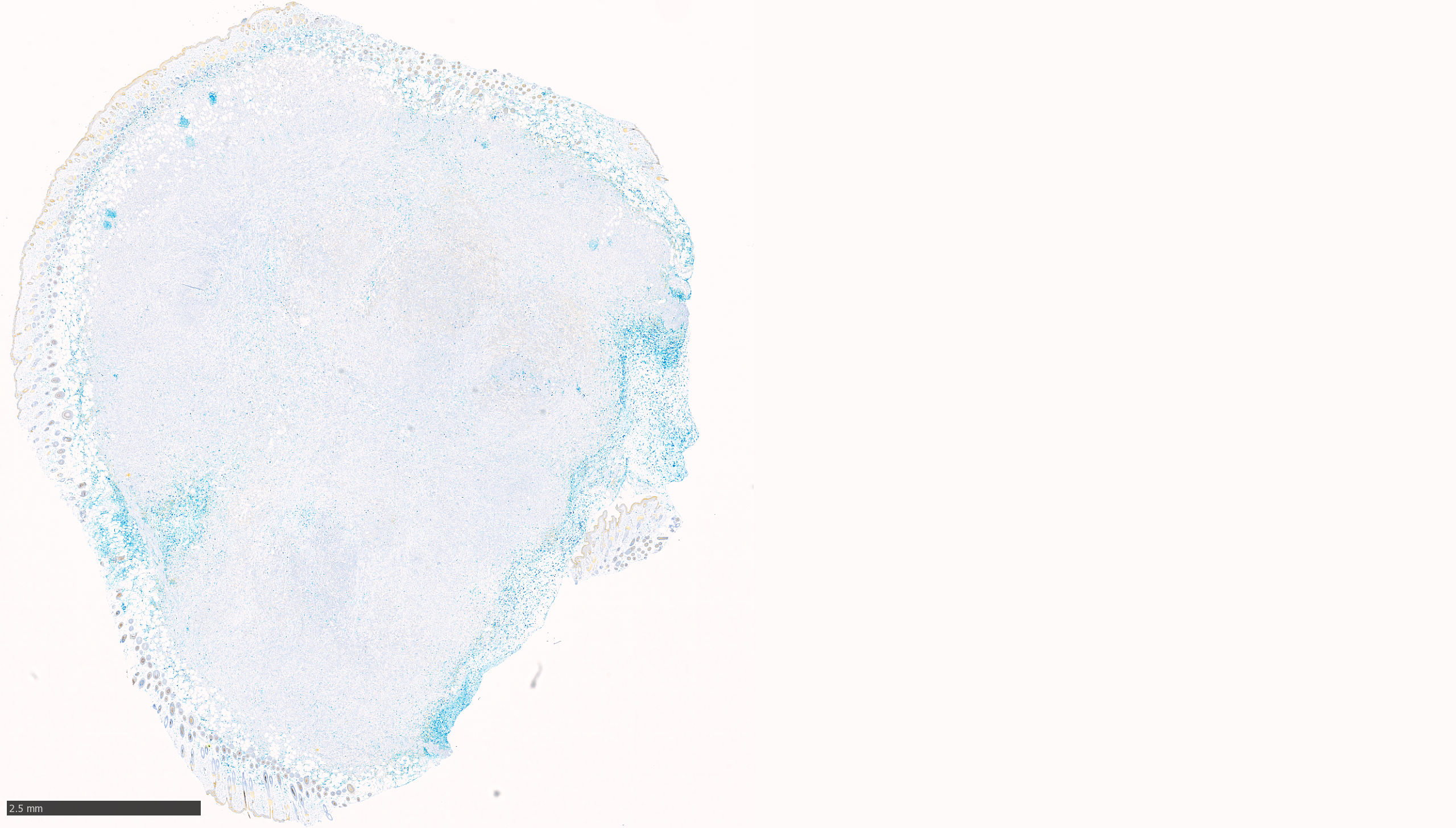

Supplement: Supplementary file 1 [file pharmaceutics-17-01273-s001.zip › IHC/CD3-CD11B/CONV-5Gy/C5-2/C5-2.jpg]

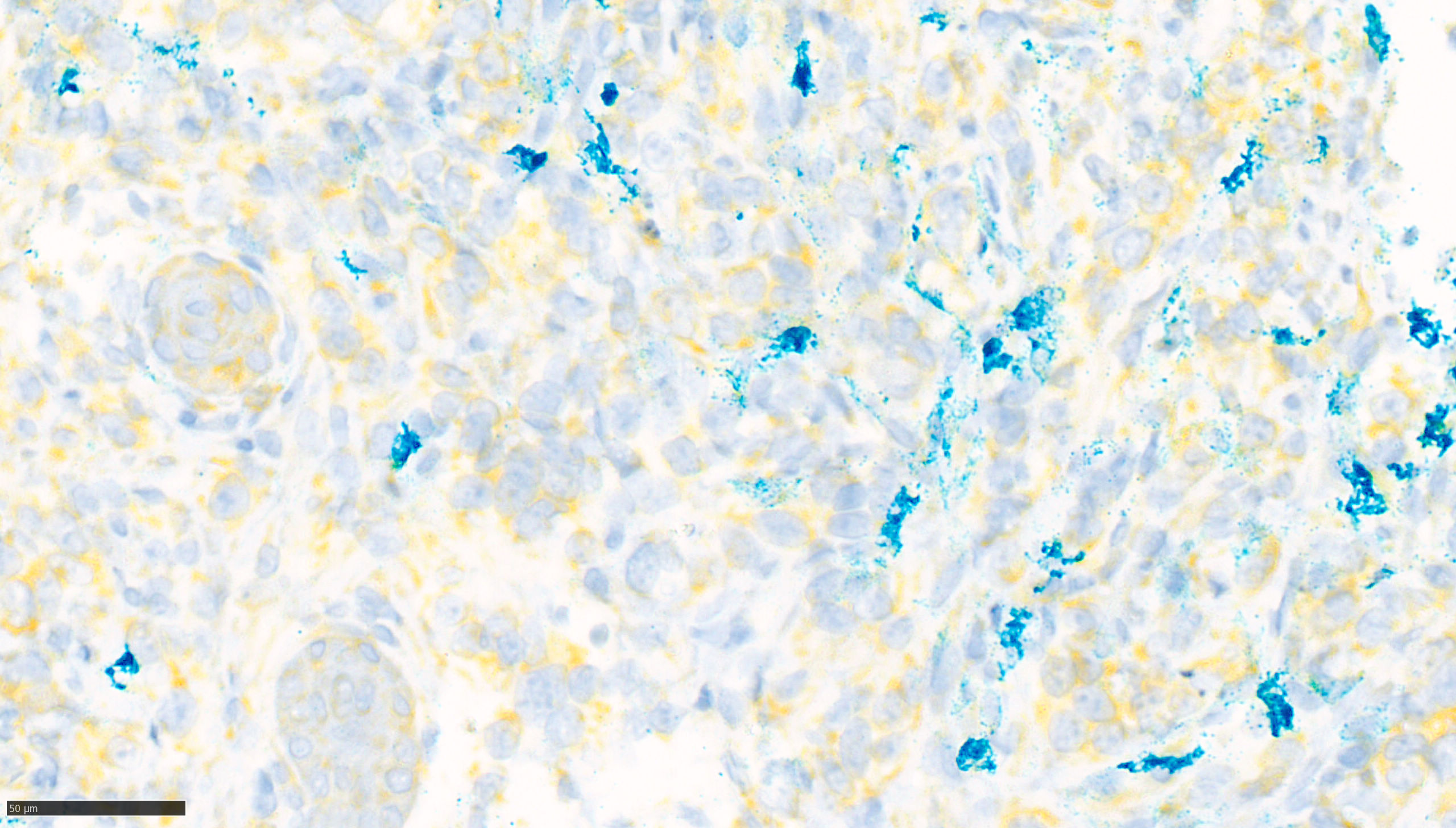

Supplement: Supplementary file 1 [file pharmaceutics-17-01273-s001.zip › IHC/CD3-CD11B/CONV-5Gy/C5-3/C5-3-1.jpg]

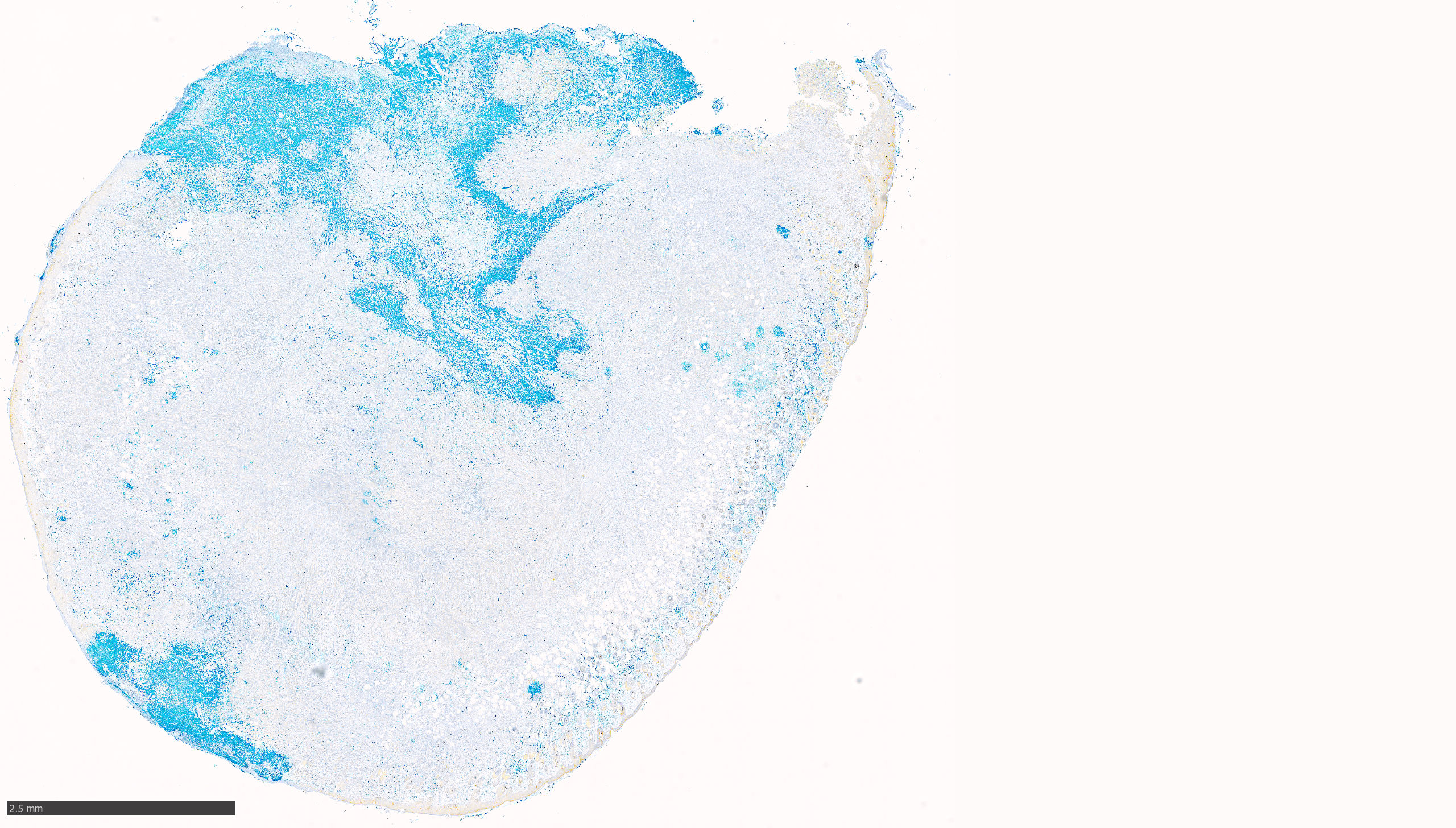

Supplement: Supplementary file 1 [file pharmaceutics-17-01273-s001.zip › IHC/CD3-CD11B/CONV-5Gy/C5-3/C5-3.jpg]

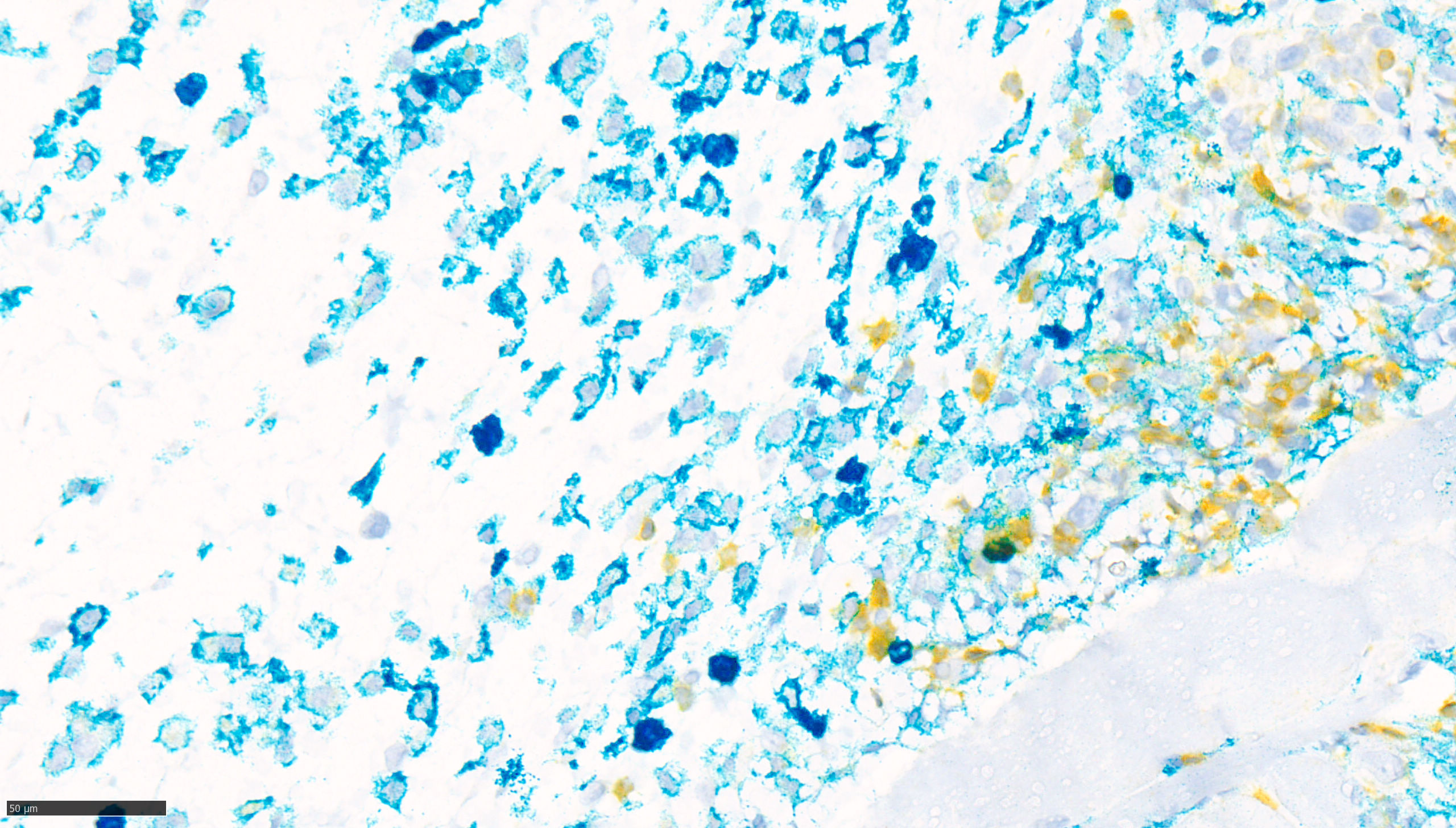

Supplement: Supplementary file 1 [file pharmaceutics-17-01273-s001.zip › IHC/CD3-CD11B/CONV-8Gy/C8-1/C8-1-1.jpg]

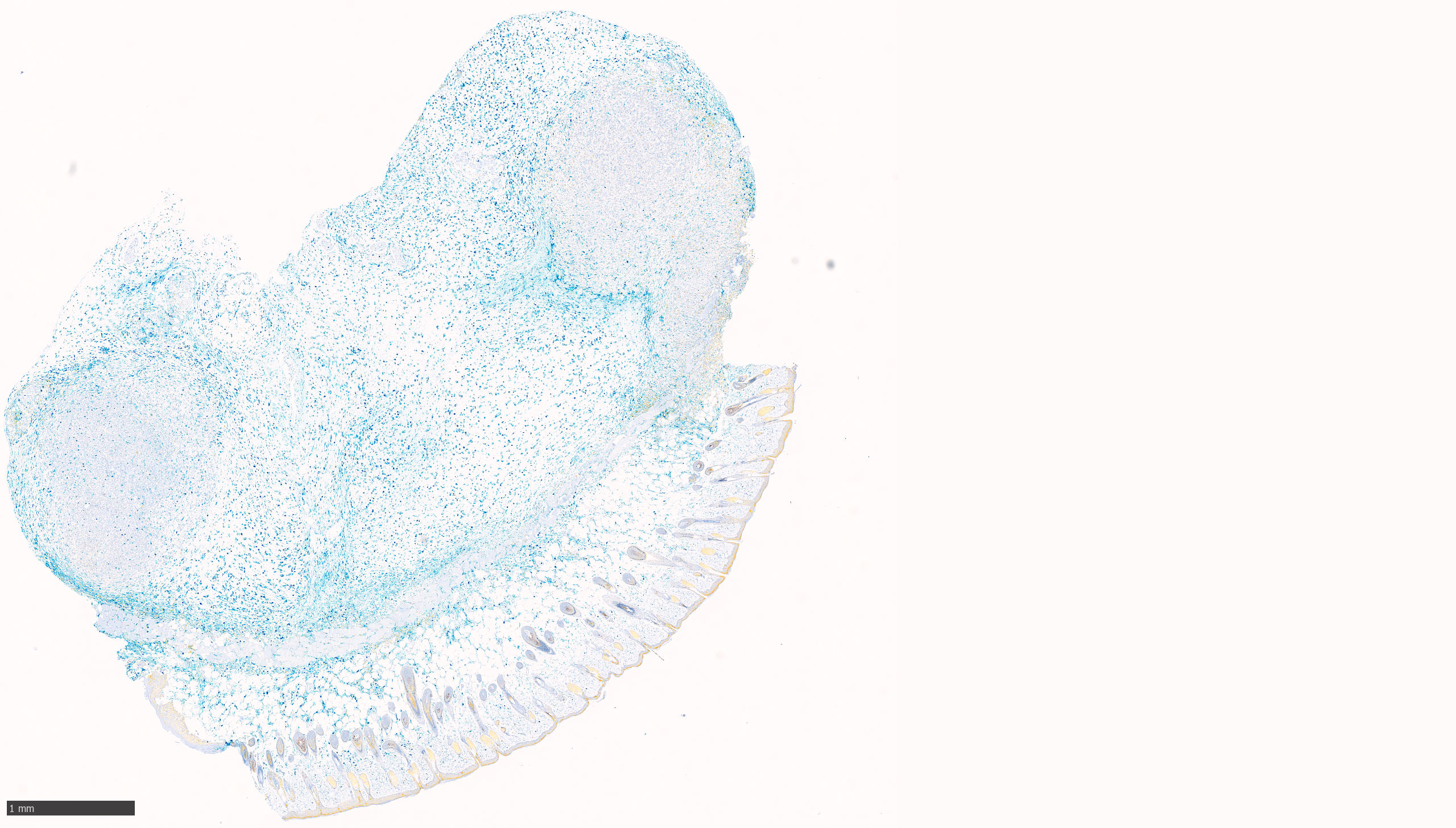

Supplement: Supplementary file 1 [file pharmaceutics-17-01273-s001.zip › IHC/CD3-CD11B/CONV-8Gy/C8-1/C8-1.jpg]

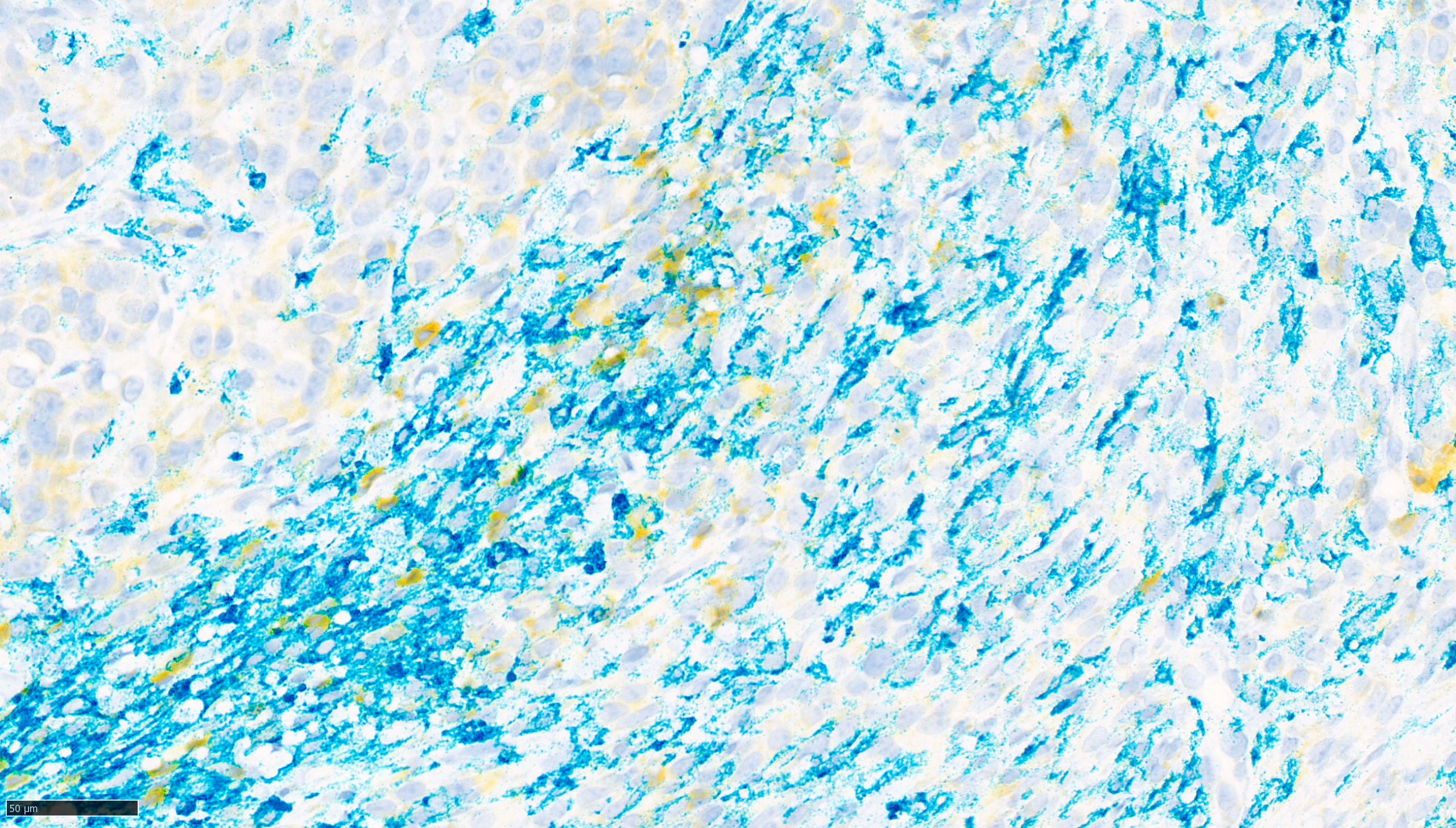

Supplement: Supplementary file 1 [file pharmaceutics-17-01273-s001.zip › IHC/CD3-CD11B/CONV-8Gy/C8-2/C8-2-1.jpg]

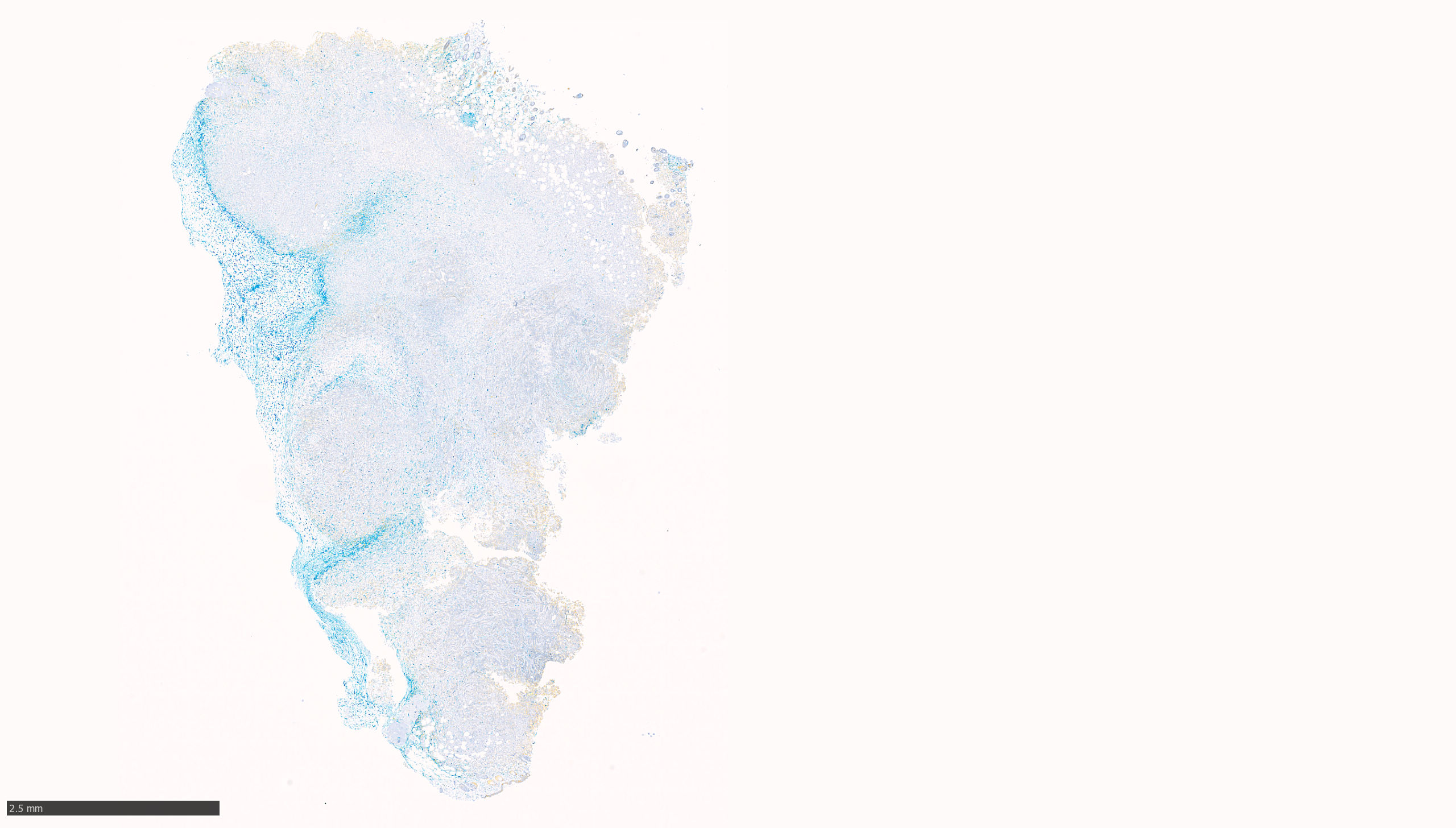

Supplement: Supplementary file 1 [file pharmaceutics-17-01273-s001.zip › IHC/CD3-CD11B/CONV-8Gy/C8-2/C8-2.jpg]

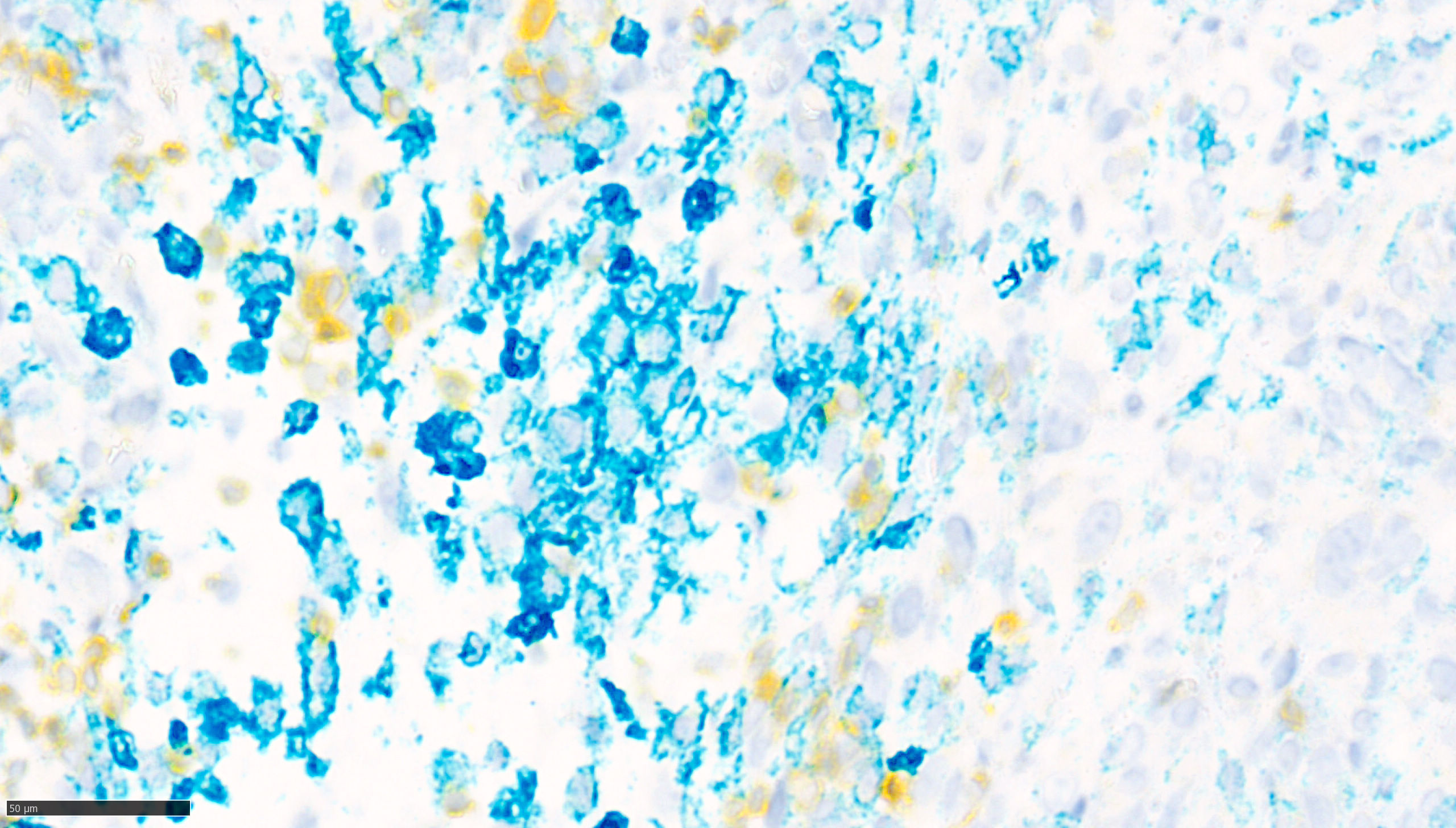

Supplement: Supplementary file 1 [file pharmaceutics-17-01273-s001.zip › IHC/CD3-CD11B/CONV-8Gy/C8-3/C8-3-1.jpg]

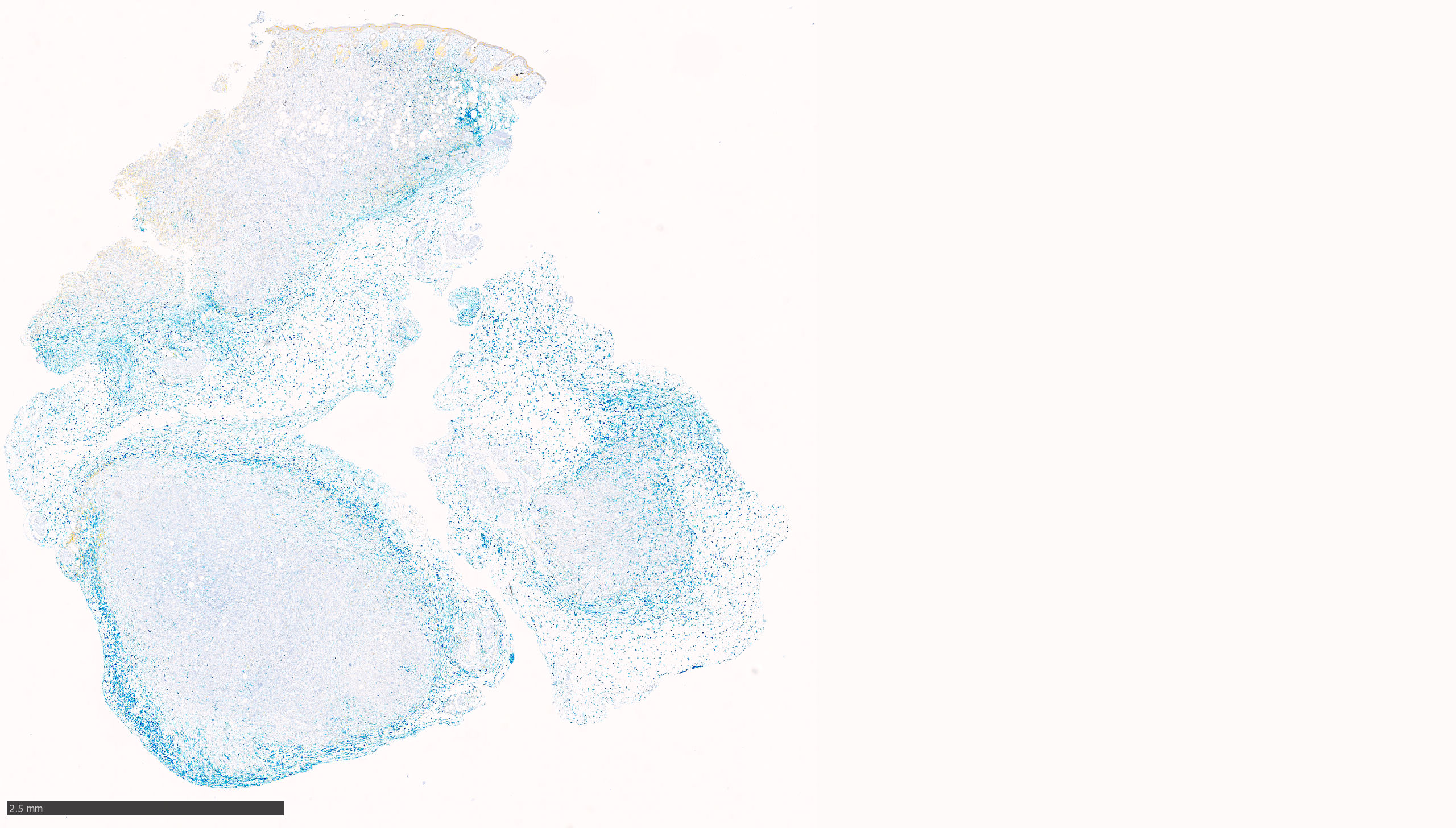

Supplement: Supplementary file 1 [file pharmaceutics-17-01273-s001.zip › IHC/CD3-CD11B/CONV-8Gy/C8-3/C8-3.jpg]

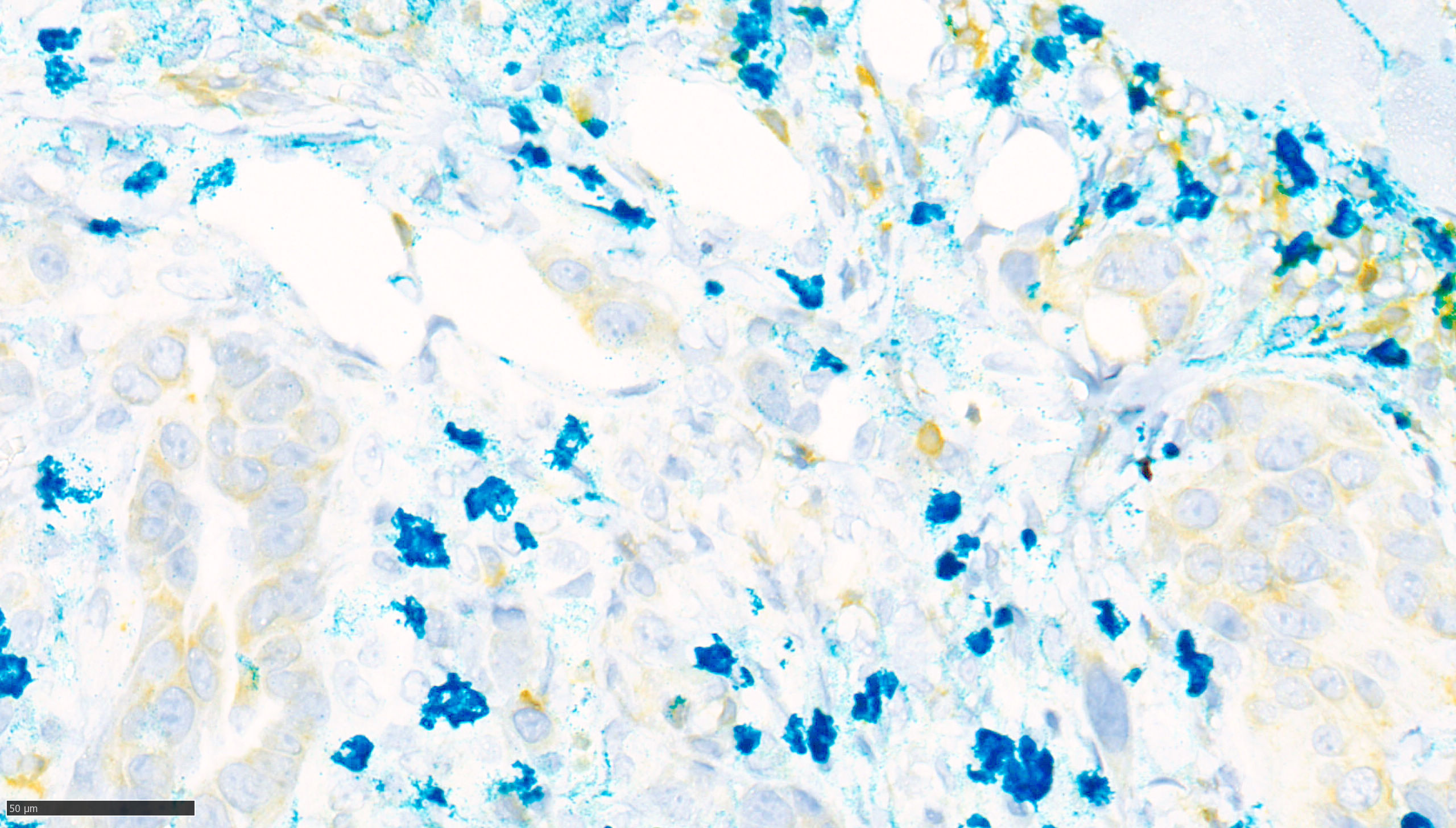

Supplement: Supplementary file 1 [file pharmaceutics-17-01273-s001.zip › IHC/CD3-CD11B/FLASH-5Gy/F5-1/F5-1-1.jpg]

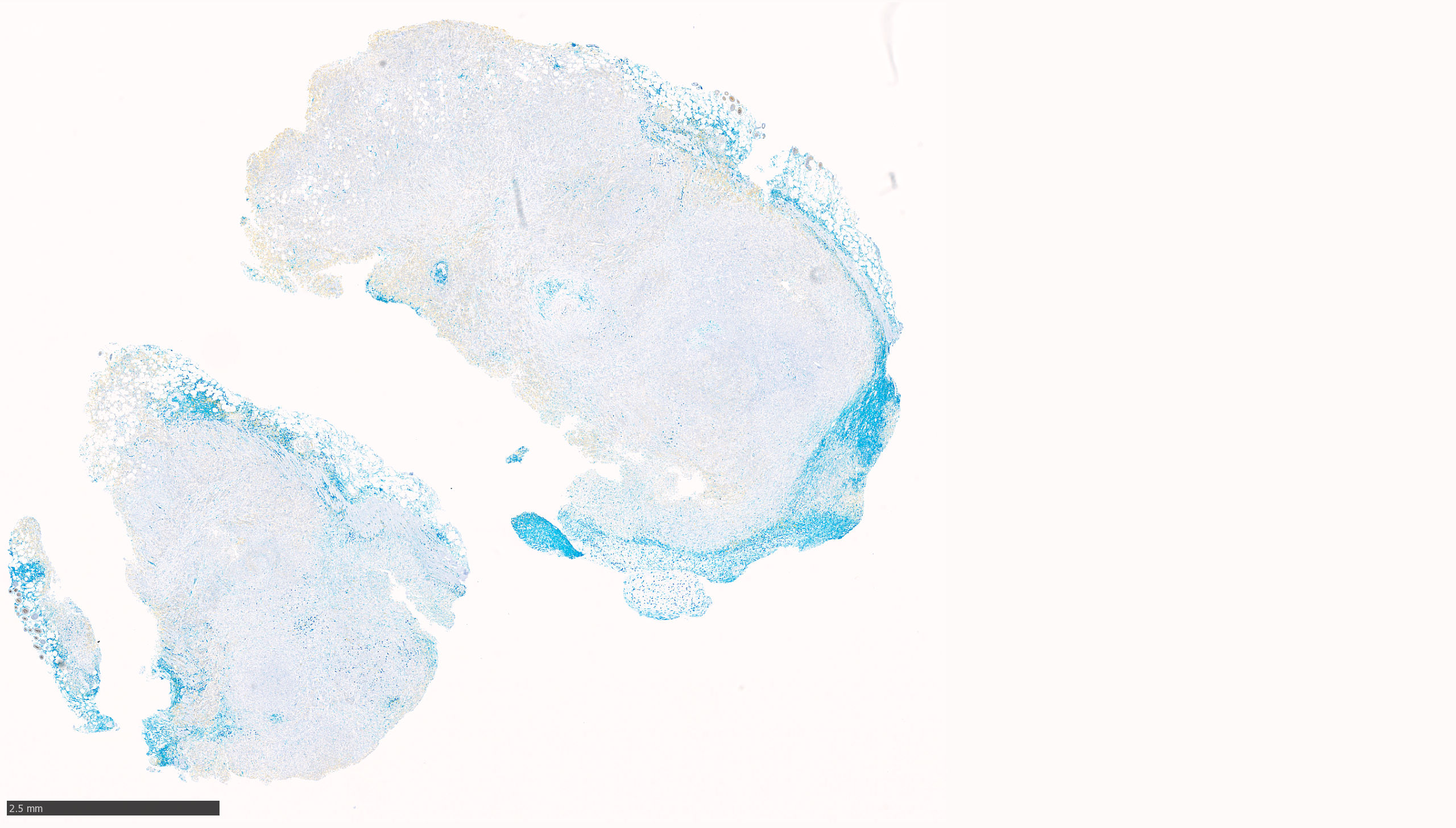

Supplement: Supplementary file 1 [file pharmaceutics-17-01273-s001.zip › IHC/CD3-CD11B/FLASH-5Gy/F5-1/F5-1.jpg]

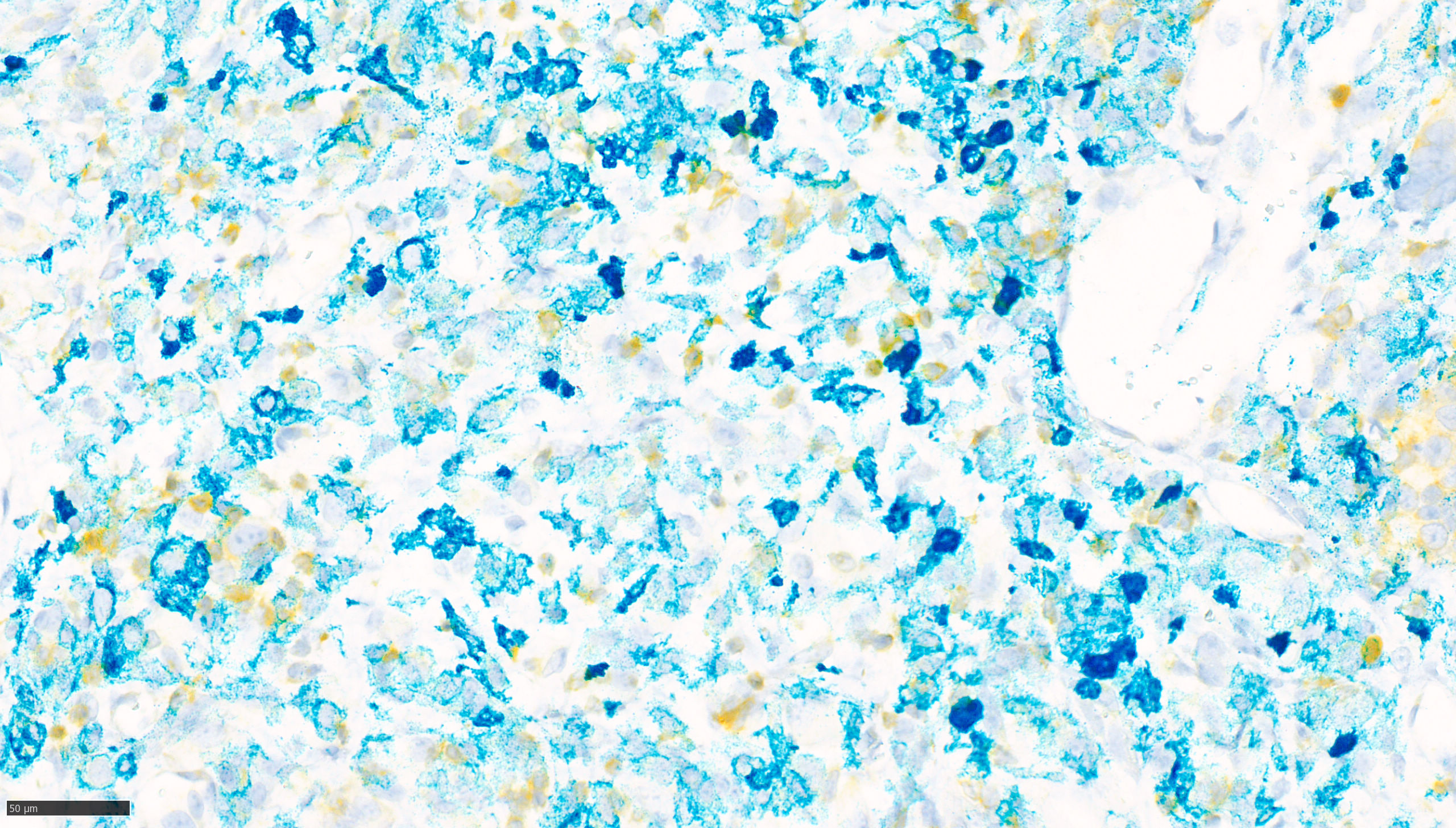

Supplement: Supplementary file 1 [file pharmaceutics-17-01273-s001.zip › IHC/CD3-CD11B/FLASH-5Gy/F5-2/F5-2-1.jpg]

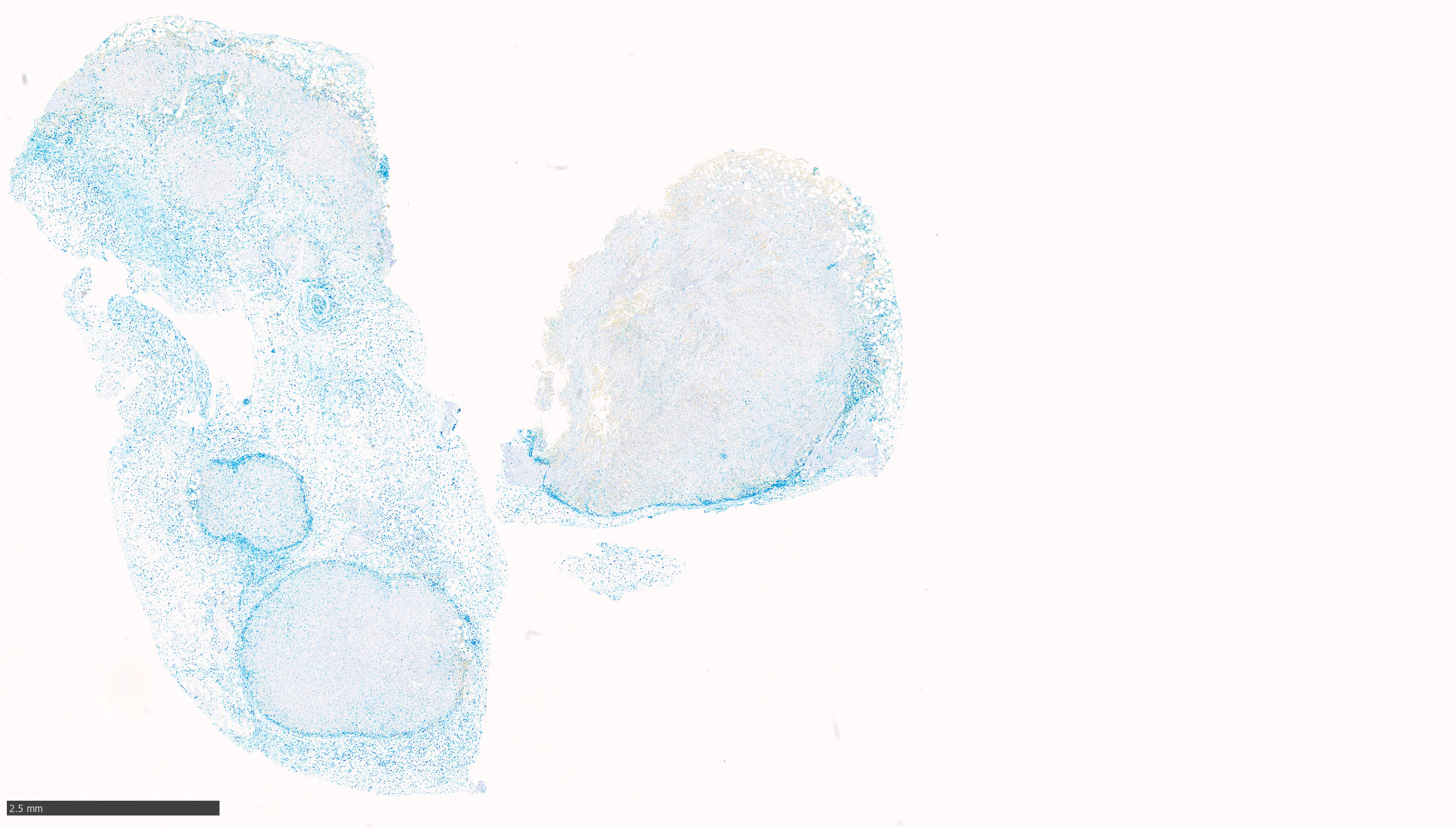

Supplement: Supplementary file 1 [file pharmaceutics-17-01273-s001.zip › IHC/CD3-CD11B/FLASH-5Gy/F5-2/F5-2.jpg]

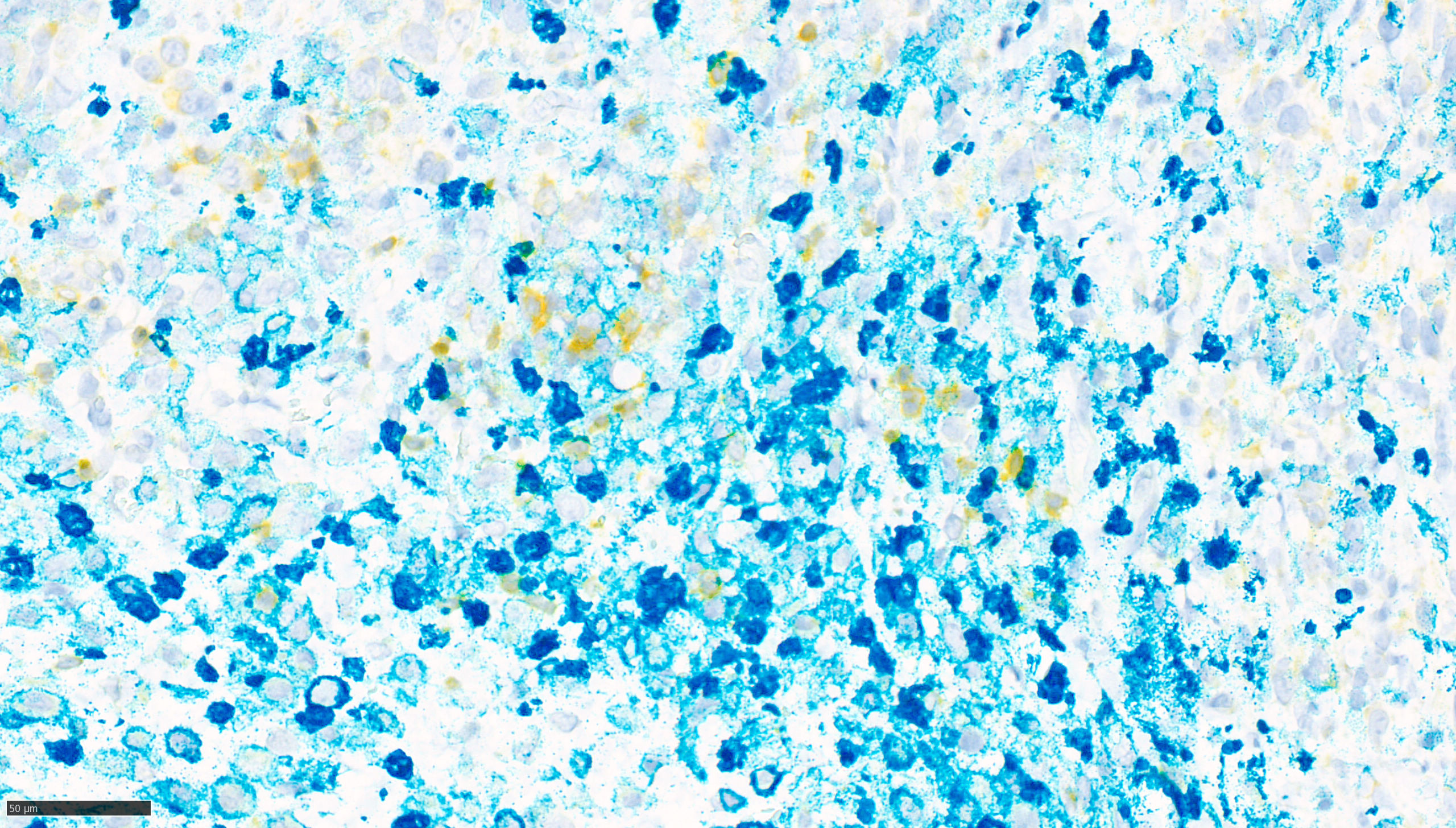

Supplement: Supplementary file 1 [file pharmaceutics-17-01273-s001.zip › IHC/CD3-CD11B/FLASH-5Gy/F5-3/F5-3-1.jpg]

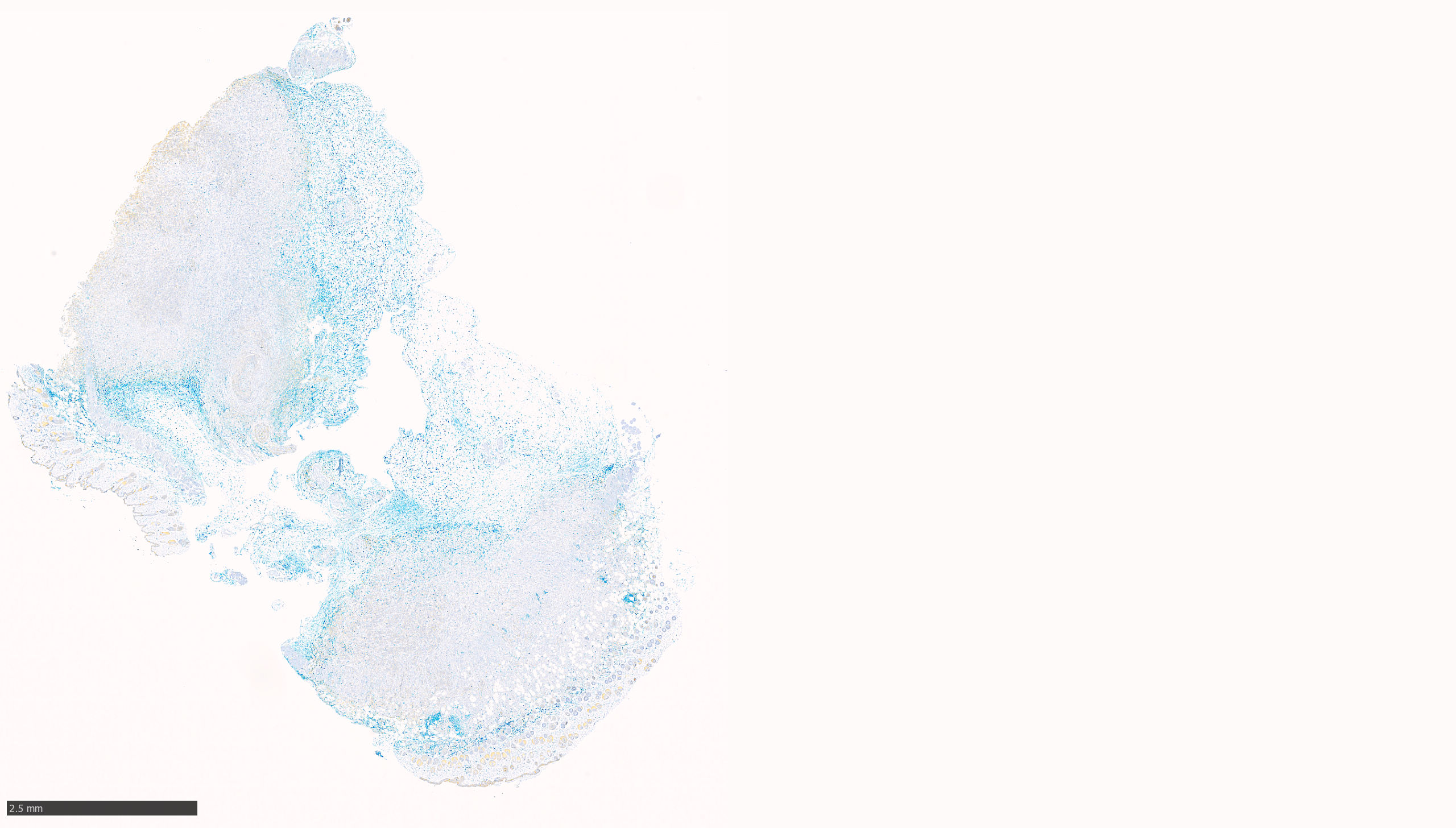

Supplement: Supplementary file 1 [file pharmaceutics-17-01273-s001.zip › IHC/CD3-CD11B/FLASH-5Gy/F5-3/F5-3.jpg]

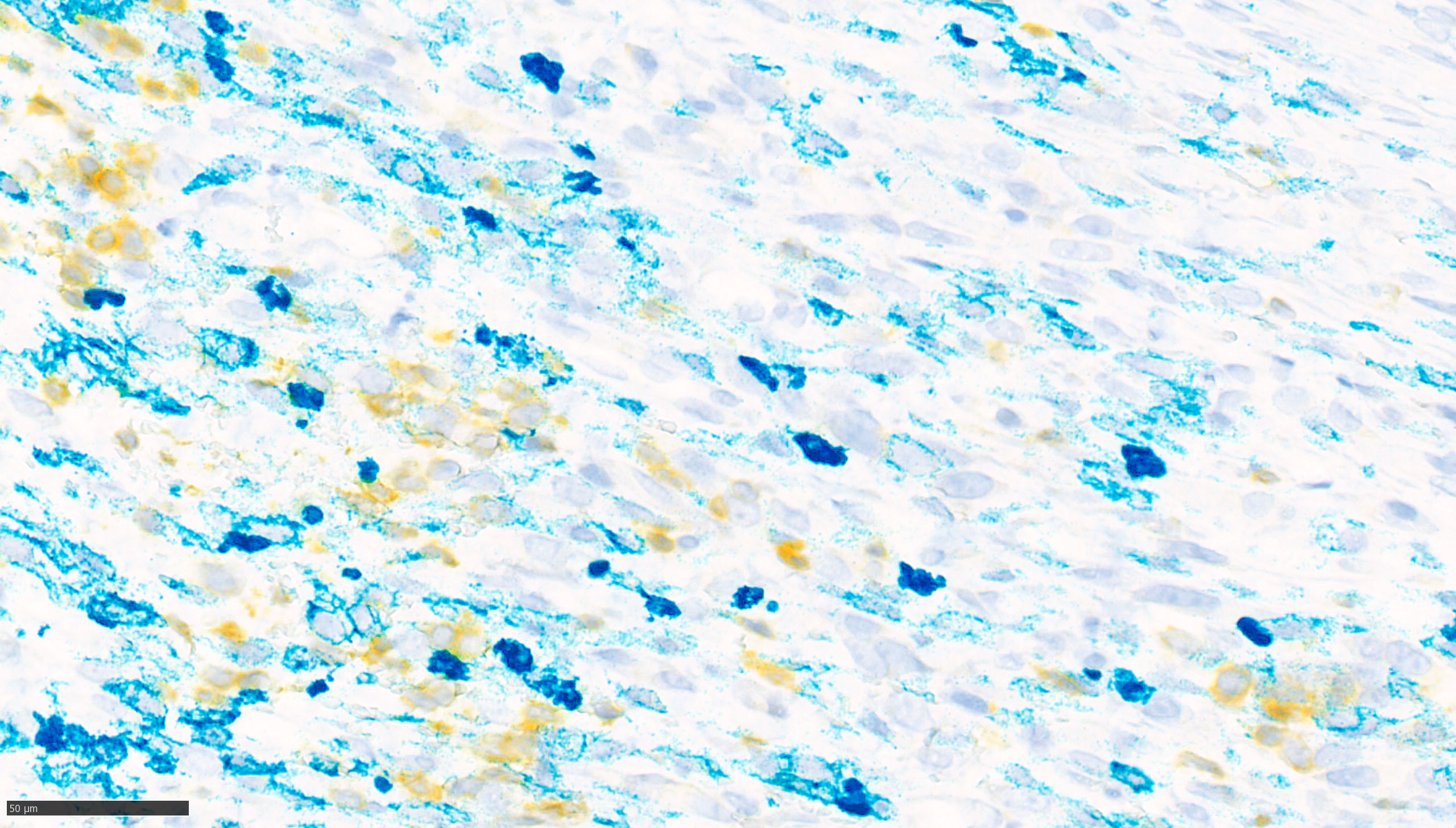

Supplement: Supplementary file 1 [file pharmaceutics-17-01273-s001.zip › IHC/CD3-CD11B/FLASH-8Gy/F8-1/F8-1-1.jpg]

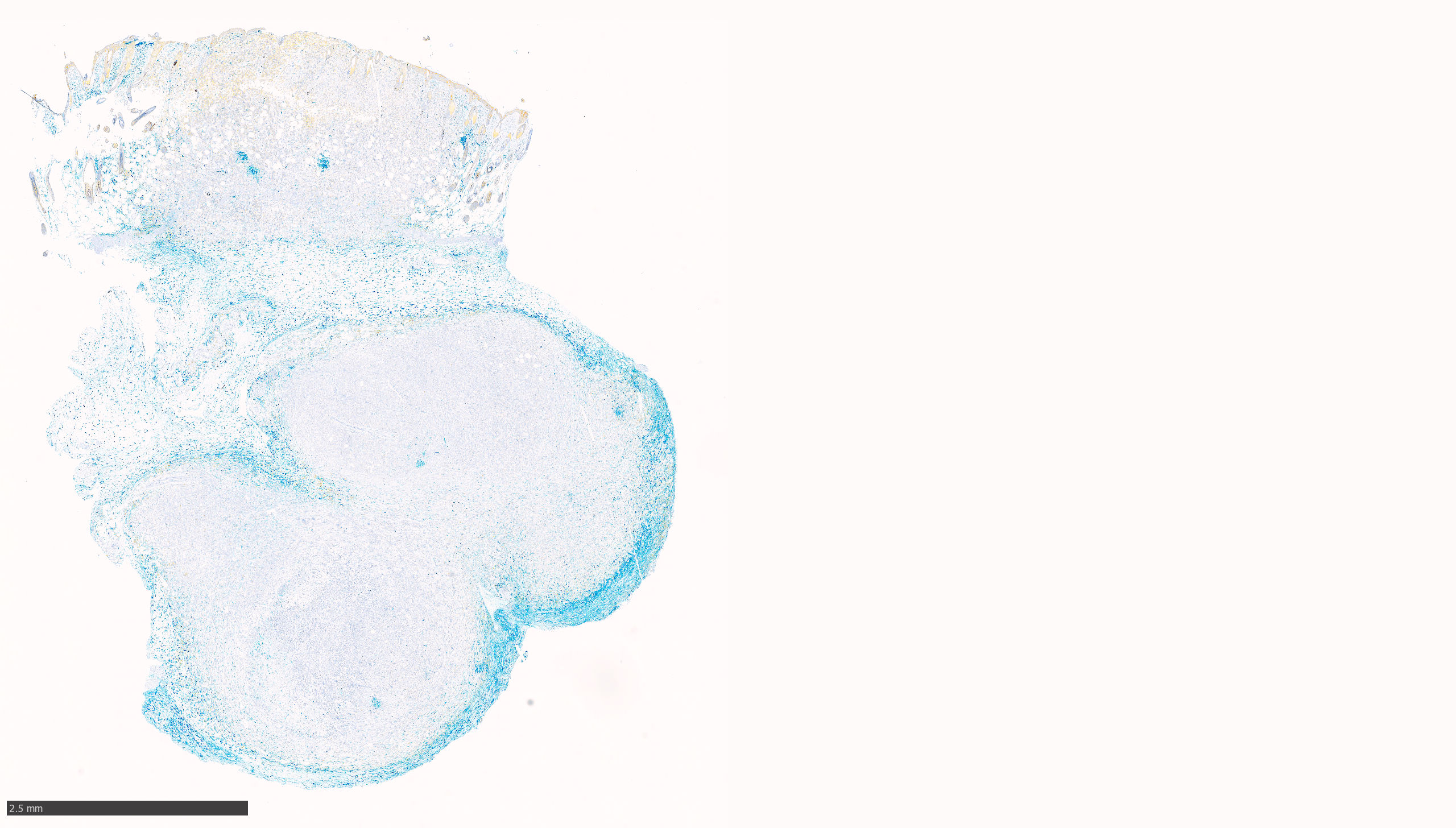

Supplement: Supplementary file 1 [file pharmaceutics-17-01273-s001.zip › IHC/CD3-CD11B/FLASH-8Gy/F8-1/F8-1.jpg]

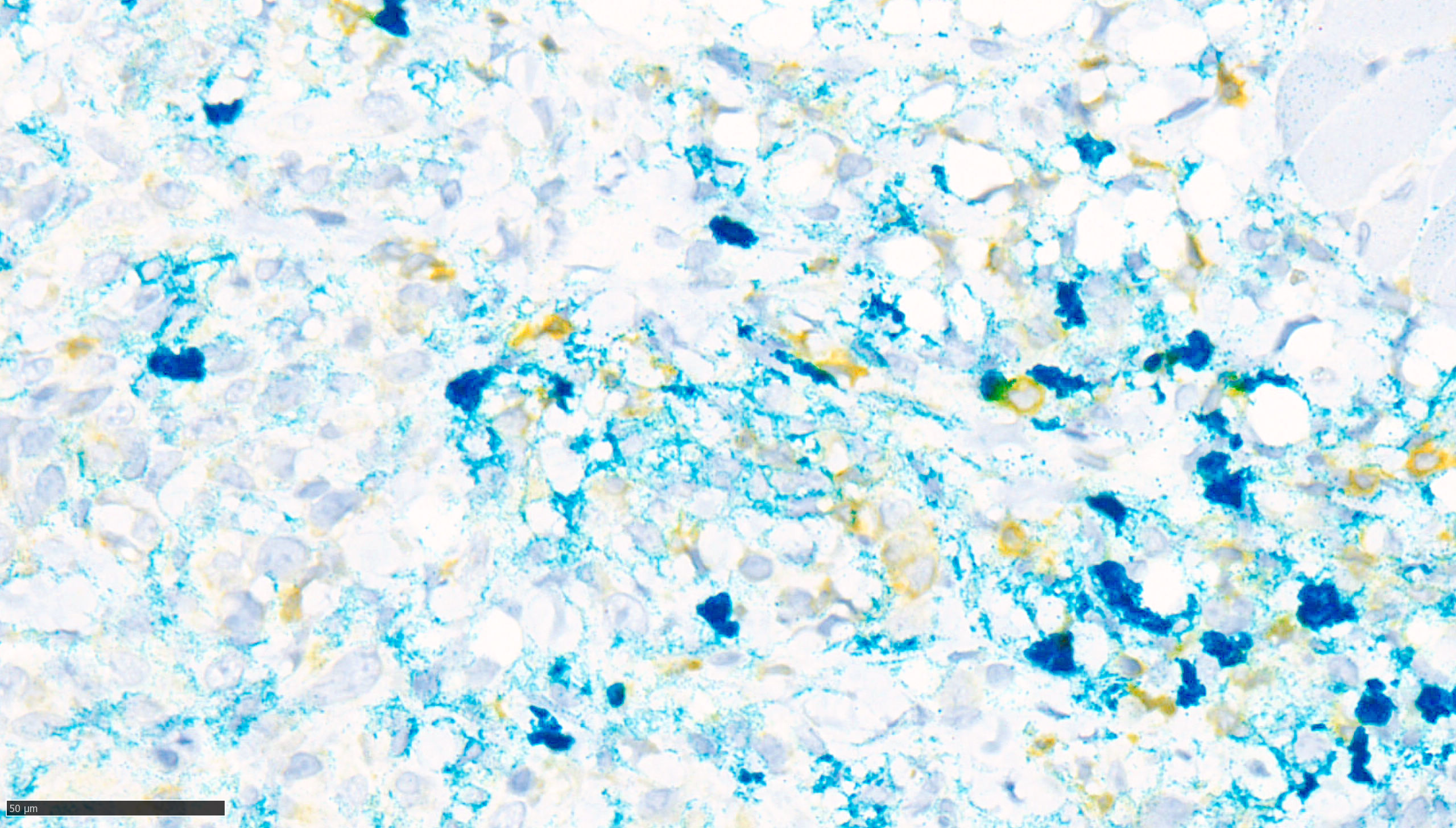

Supplement: Supplementary file 1 [file pharmaceutics-17-01273-s001.zip › IHC/CD3-CD11B/FLASH-8Gy/F8-2/F8-2-1.jpg]

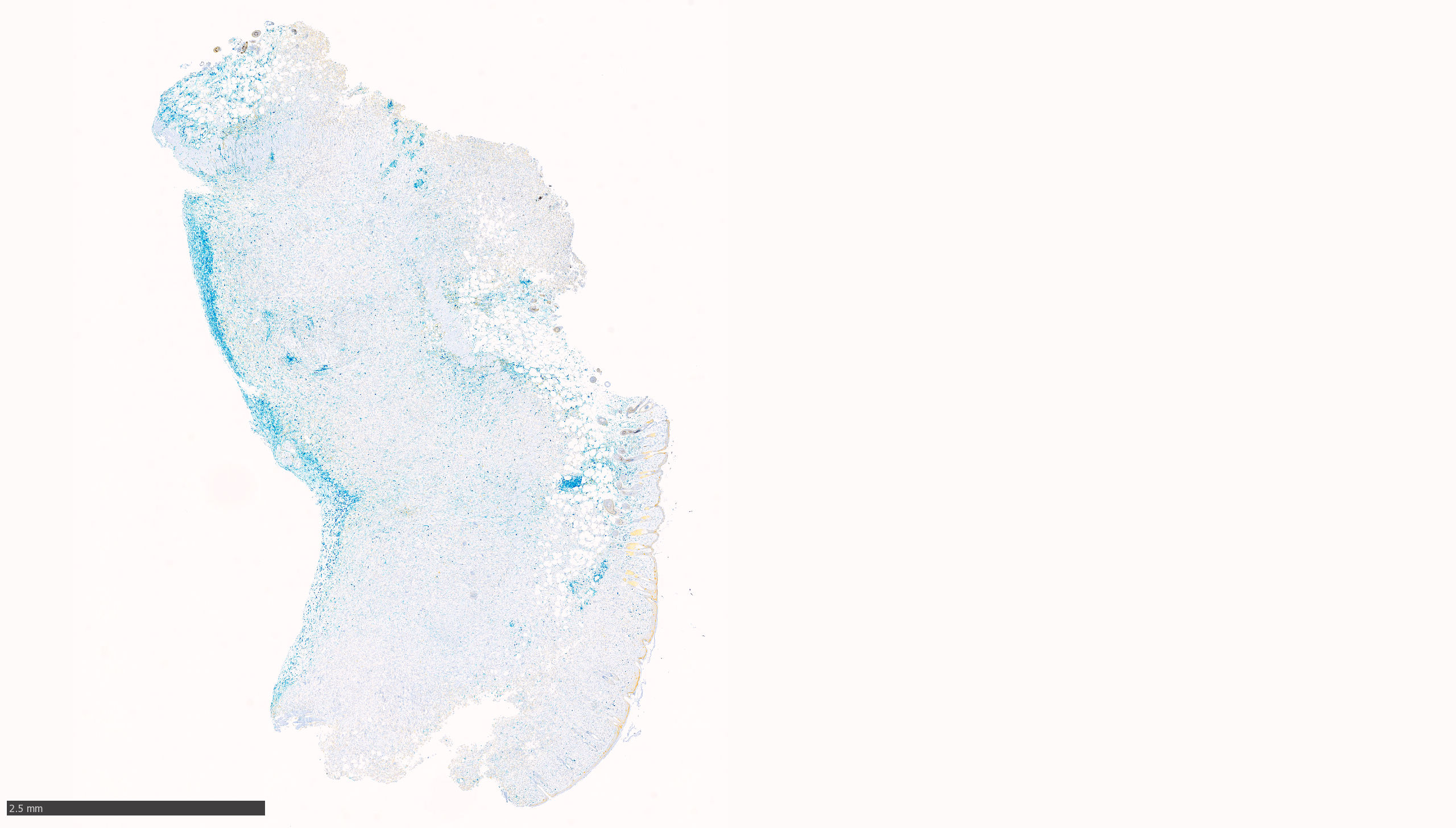

Supplement: Supplementary file 1 [file pharmaceutics-17-01273-s001.zip › IHC/CD3-CD11B/FLASH-8Gy/F8-2/F8-2.jpg]

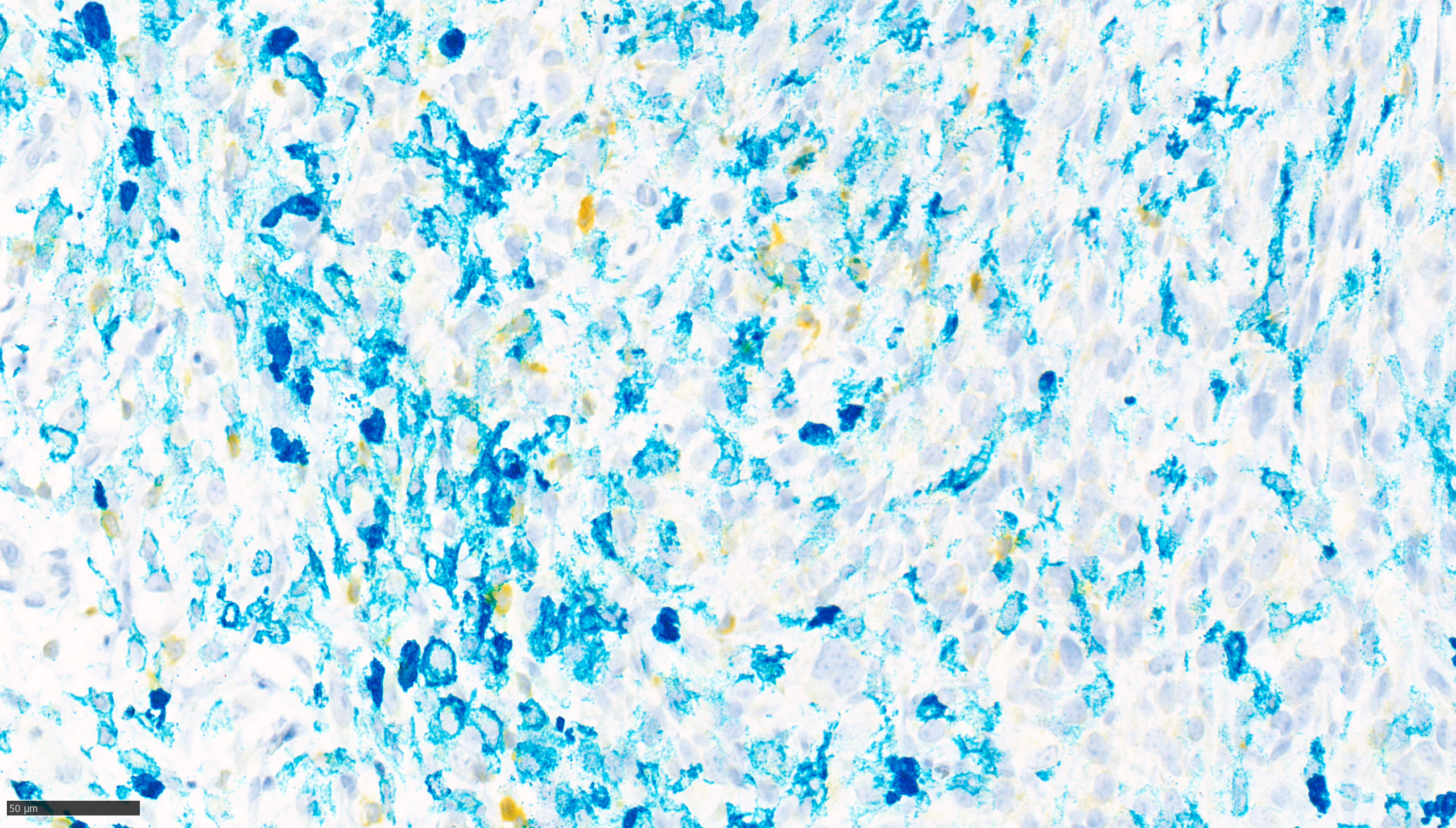

Supplement: Supplementary file 1 [file pharmaceutics-17-01273-s001.zip › IHC/CD3-CD11B/FLASH-8Gy/F8-3/F8-3-1.jpg]

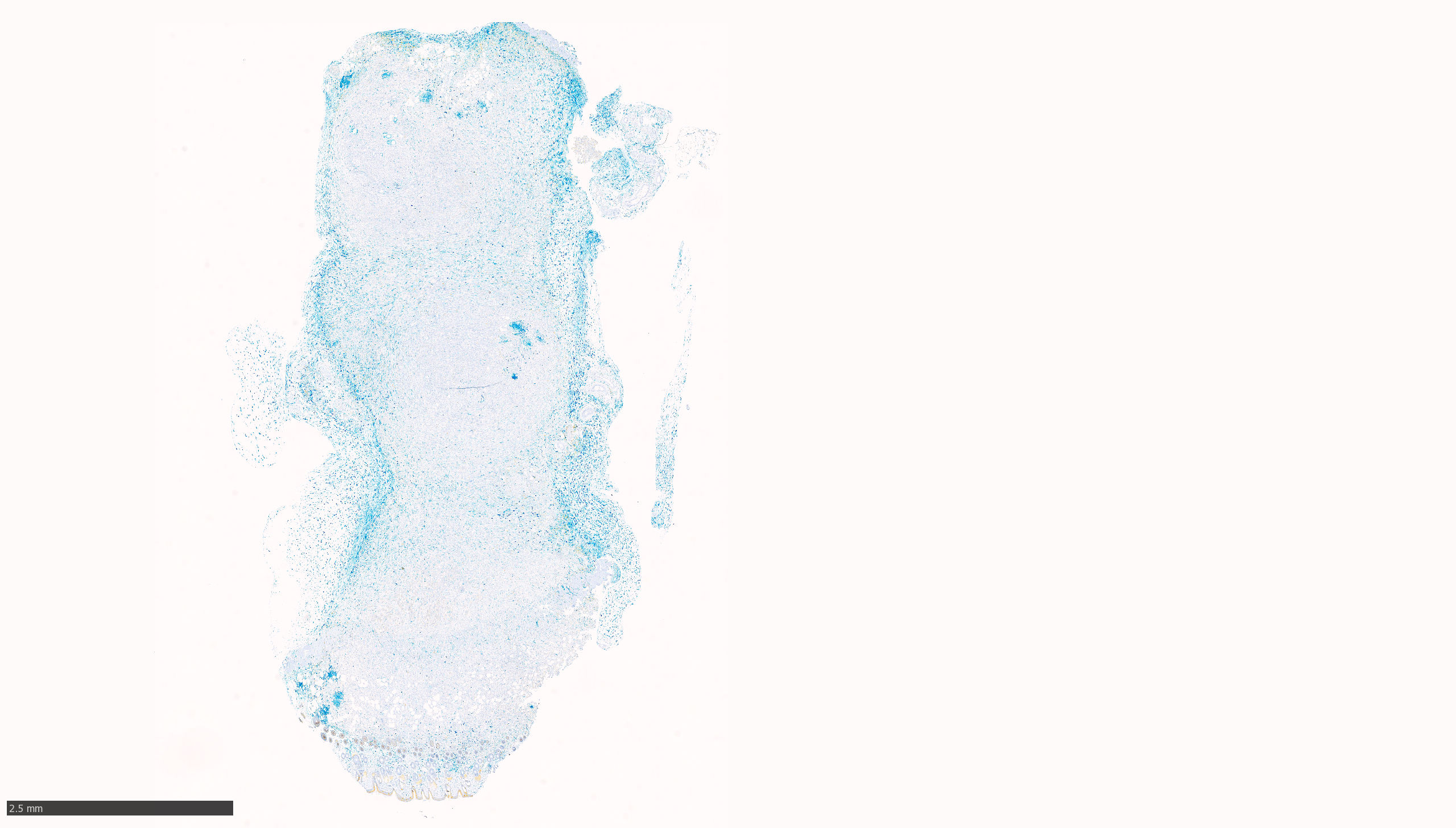

Supplement: Supplementary file 1 [file pharmaceutics-17-01273-s001.zip › IHC/CD3-CD11B/FLASH-8Gy/F8-3/F8-3.jpg]

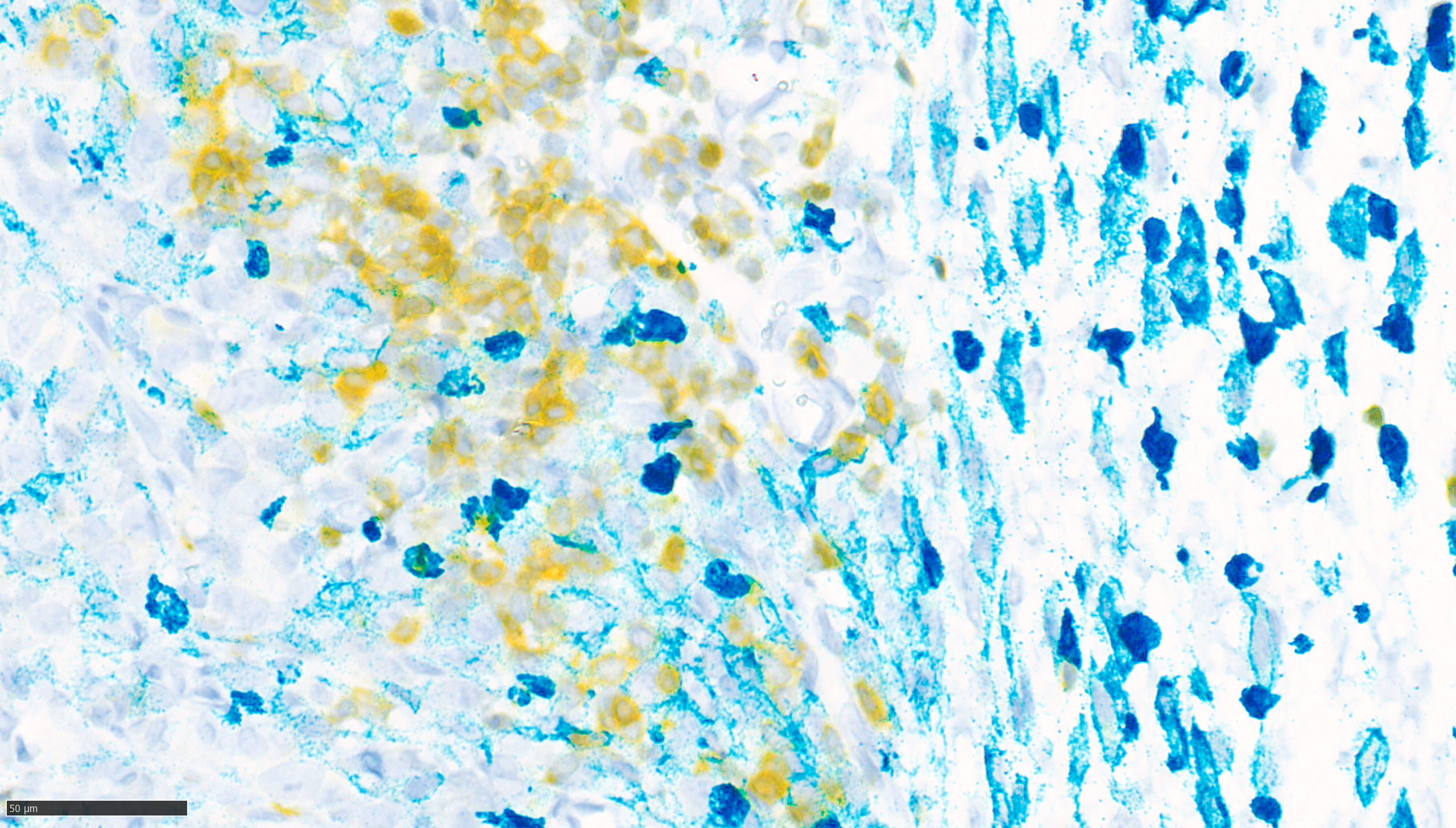

Supplement: Supplementary file 1 [file pharmaceutics-17-01273-s001.zip › IHC/CD3-CD11B/LIFE BIOMATERIAL/L1/L1-1.jpg]

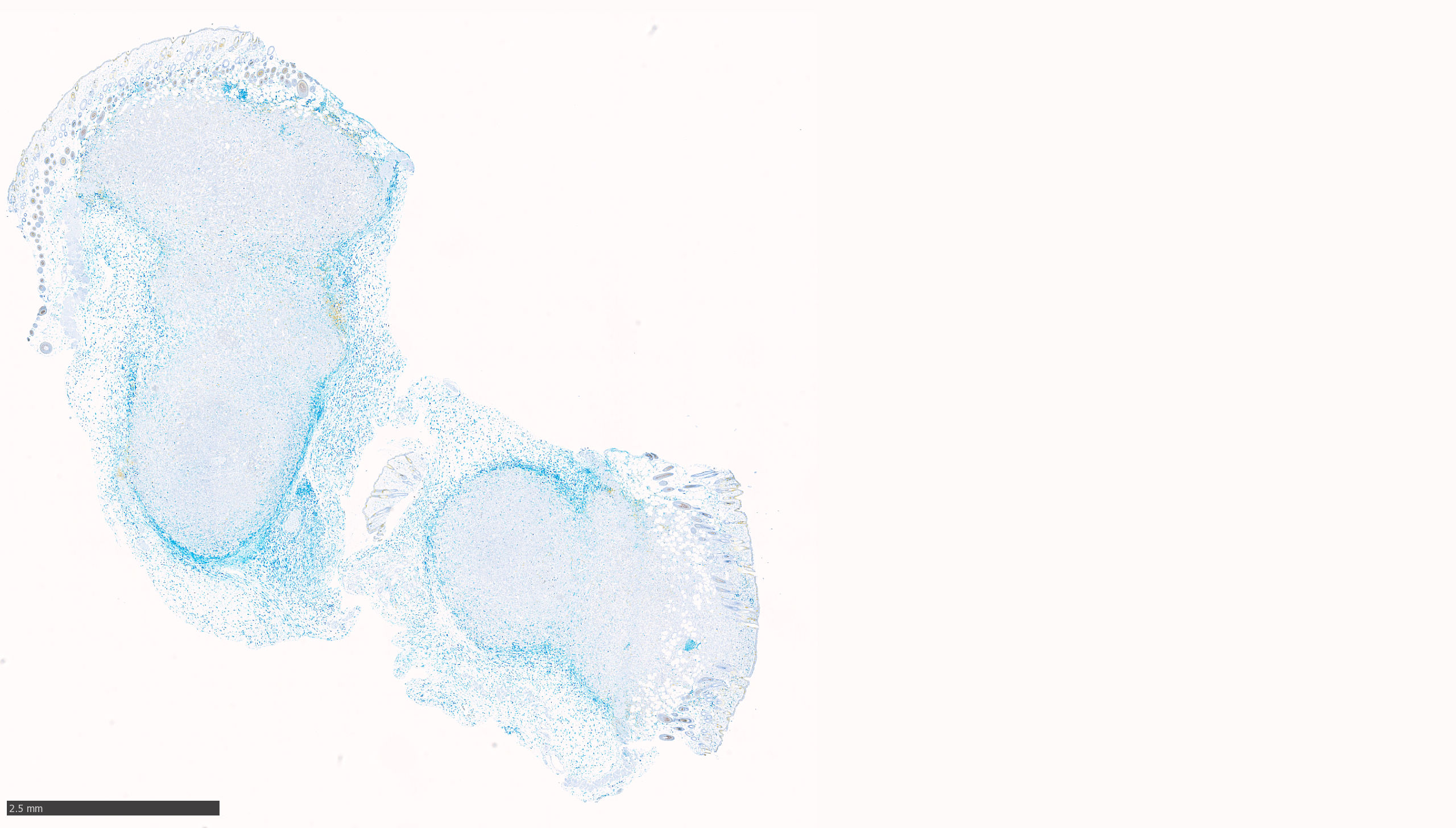

Supplement: Supplementary file 1 [file pharmaceutics-17-01273-s001.zip › IHC/CD3-CD11B/LIFE BIOMATERIAL/L1/L1.jpg]

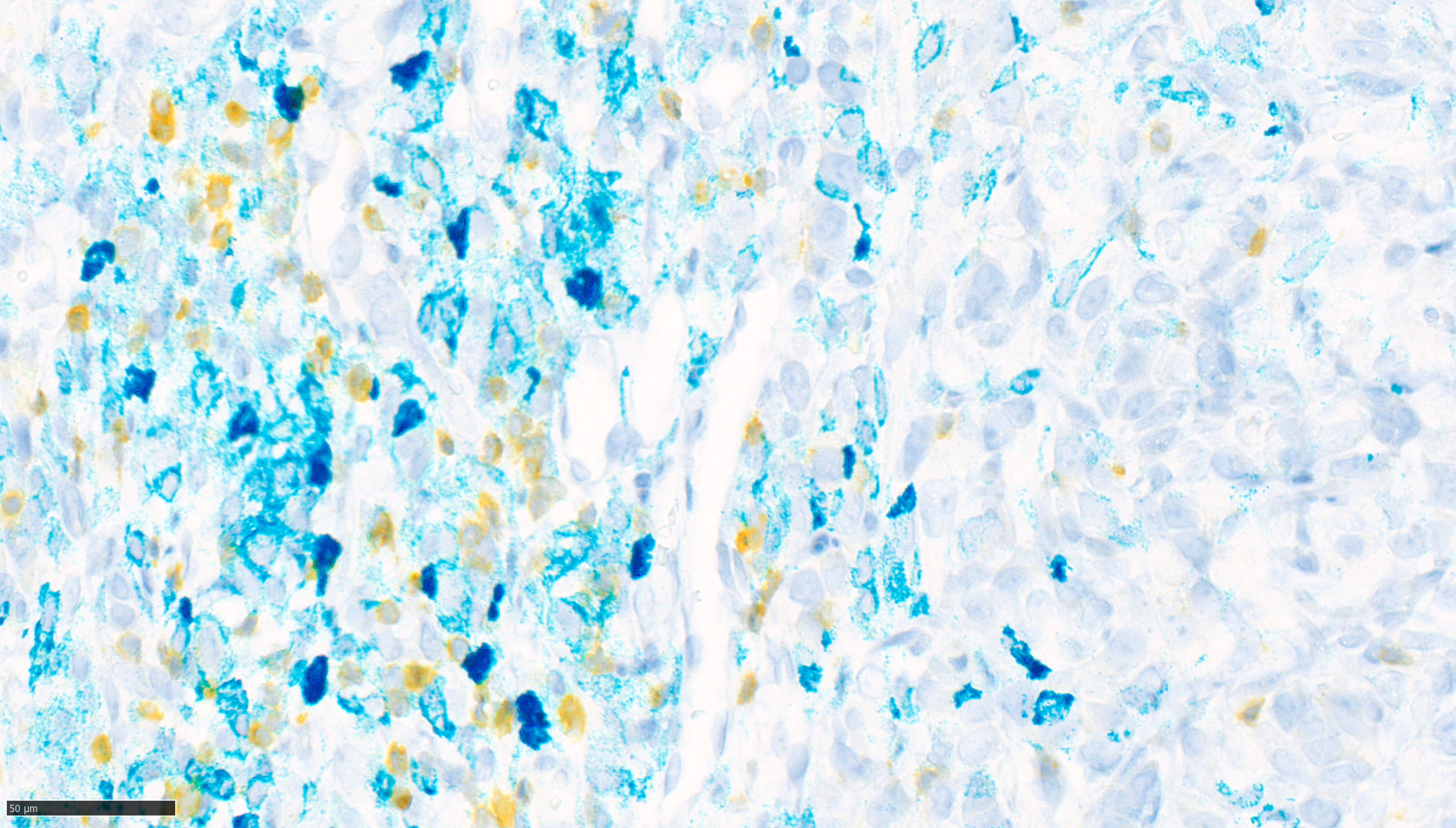

Supplement: Supplementary file 1 [file pharmaceutics-17-01273-s001.zip › IHC/CD3-CD11B/LIFE BIOMATERIAL/L2/L2-1.jpg]

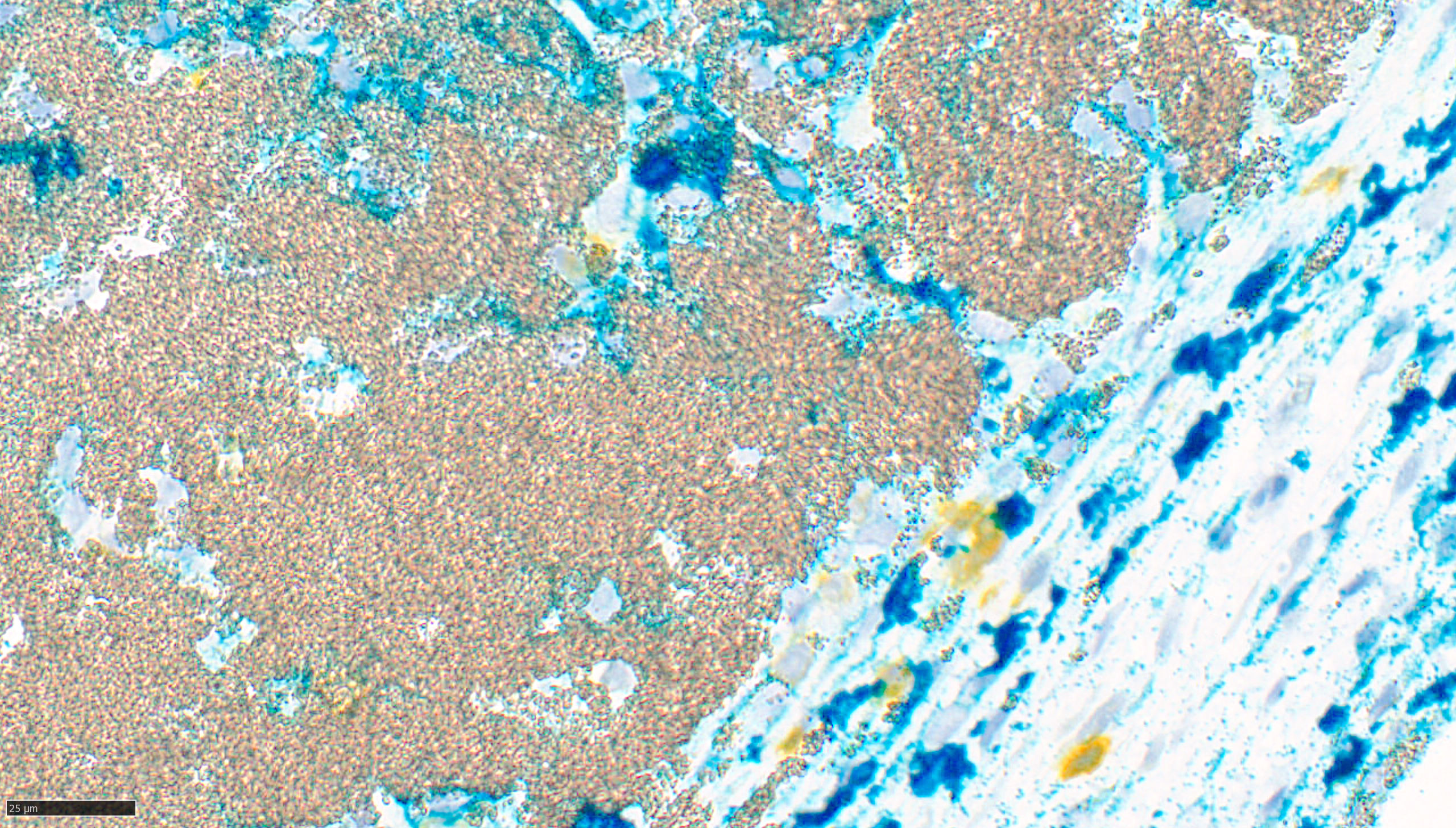

Supplement: Supplementary file 1 [file pharmaceutics-17-01273-s001.zip › IHC/CD3-CD11B/LIFE BIOMATERIAL/L2/L2-2.jpg]

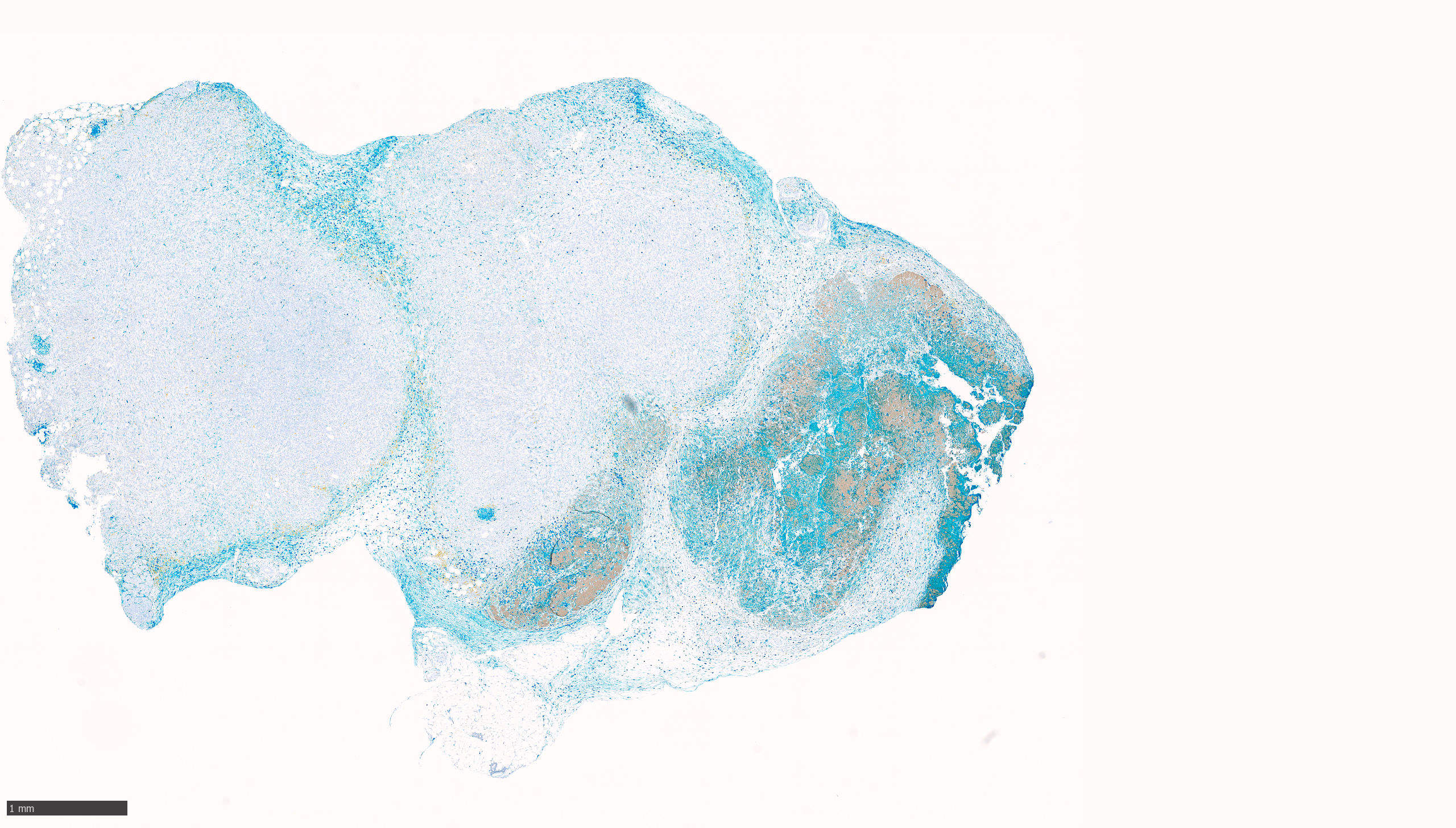

Supplement: Supplementary file 1 [file pharmaceutics-17-01273-s001.zip › IHC/CD3-CD11B/LIFE BIOMATERIAL/L2/L2.jpg]

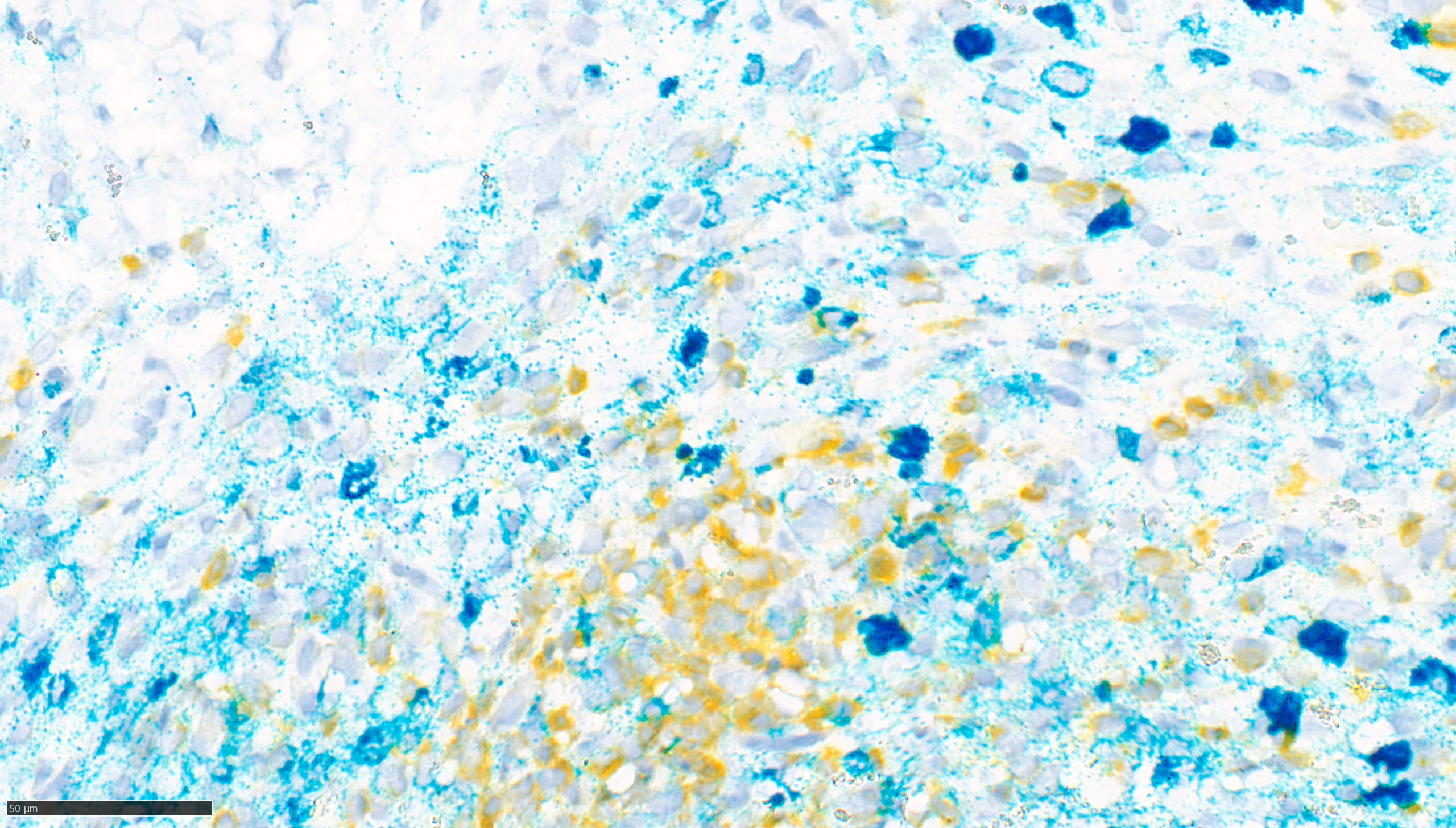

Supplement: Supplementary file 1 [file pharmaceutics-17-01273-s001.zip › IHC/CD3-CD11B/LIFE BIOMATERIAL_CONV-5Gy/C5-L1/C5-L1-1.jpg]

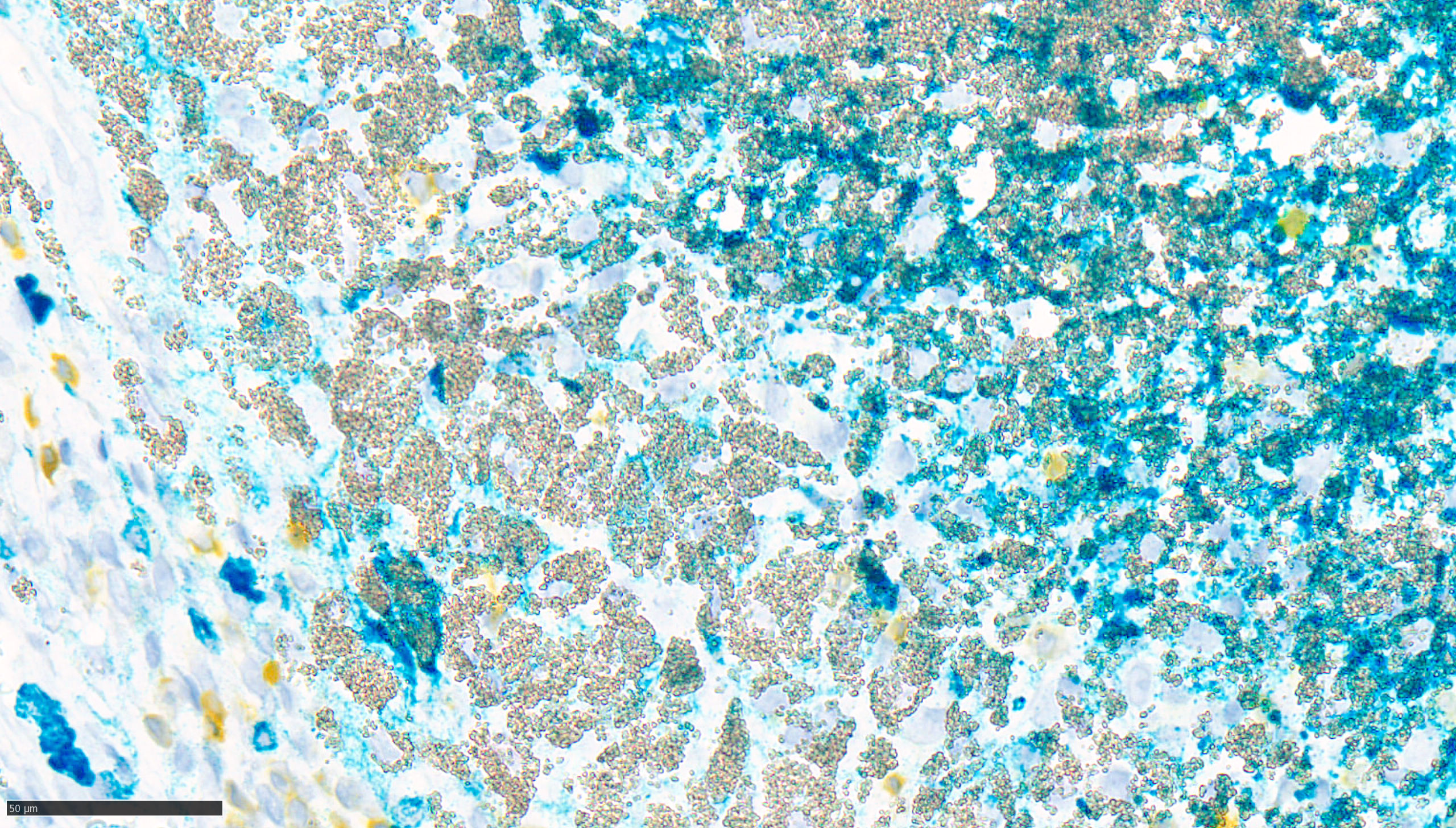

Supplement: Supplementary file 1 [file pharmaceutics-17-01273-s001.zip › IHC/CD3-CD11B/LIFE BIOMATERIAL_CONV-5Gy/C5-L1/C5-L1-2.jpg]

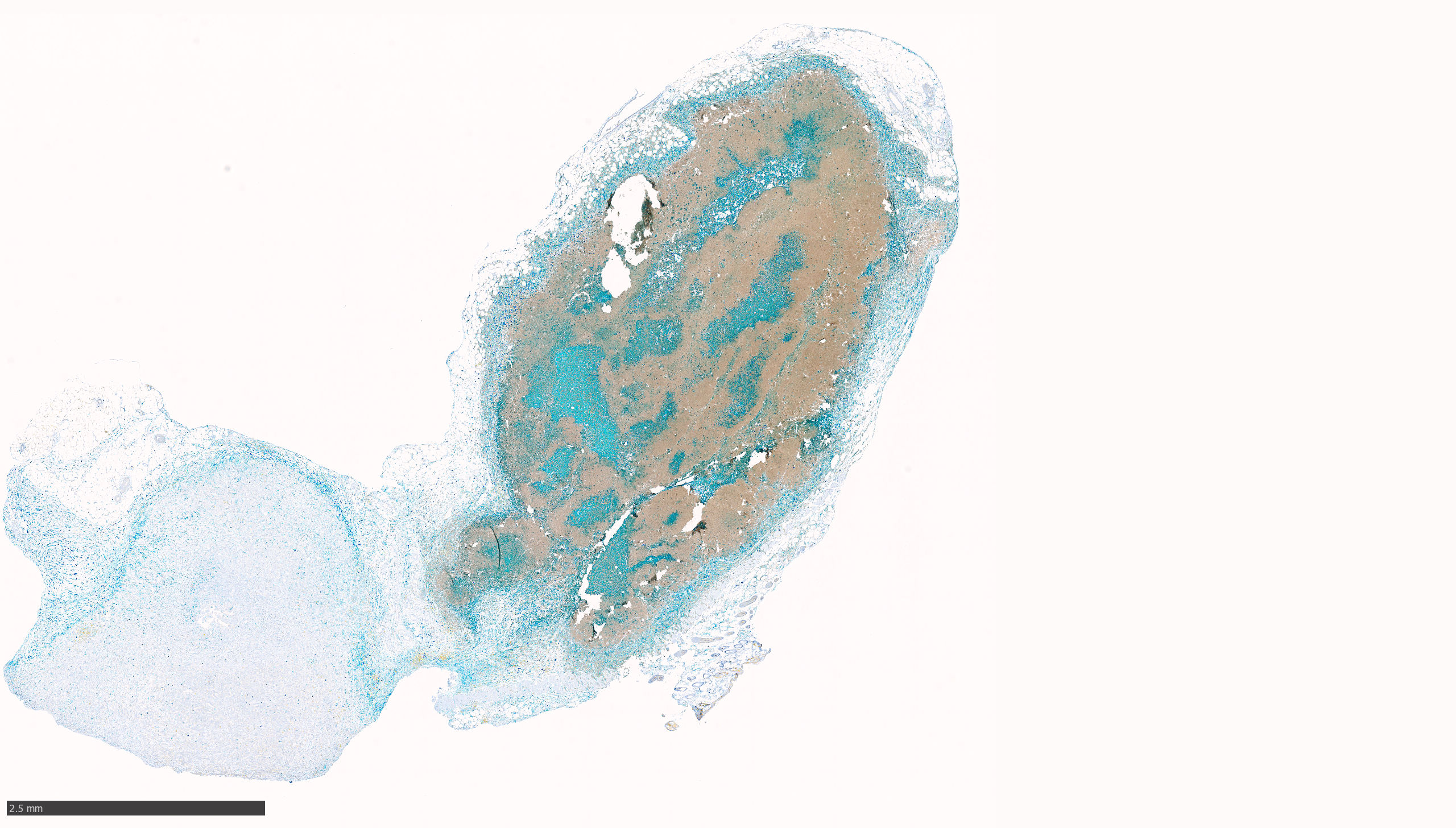

Supplement: Supplementary file 1 [file pharmaceutics-17-01273-s001.zip › IHC/CD3-CD11B/LIFE BIOMATERIAL_CONV-5Gy/C5-L1/C5-L1.jpg]

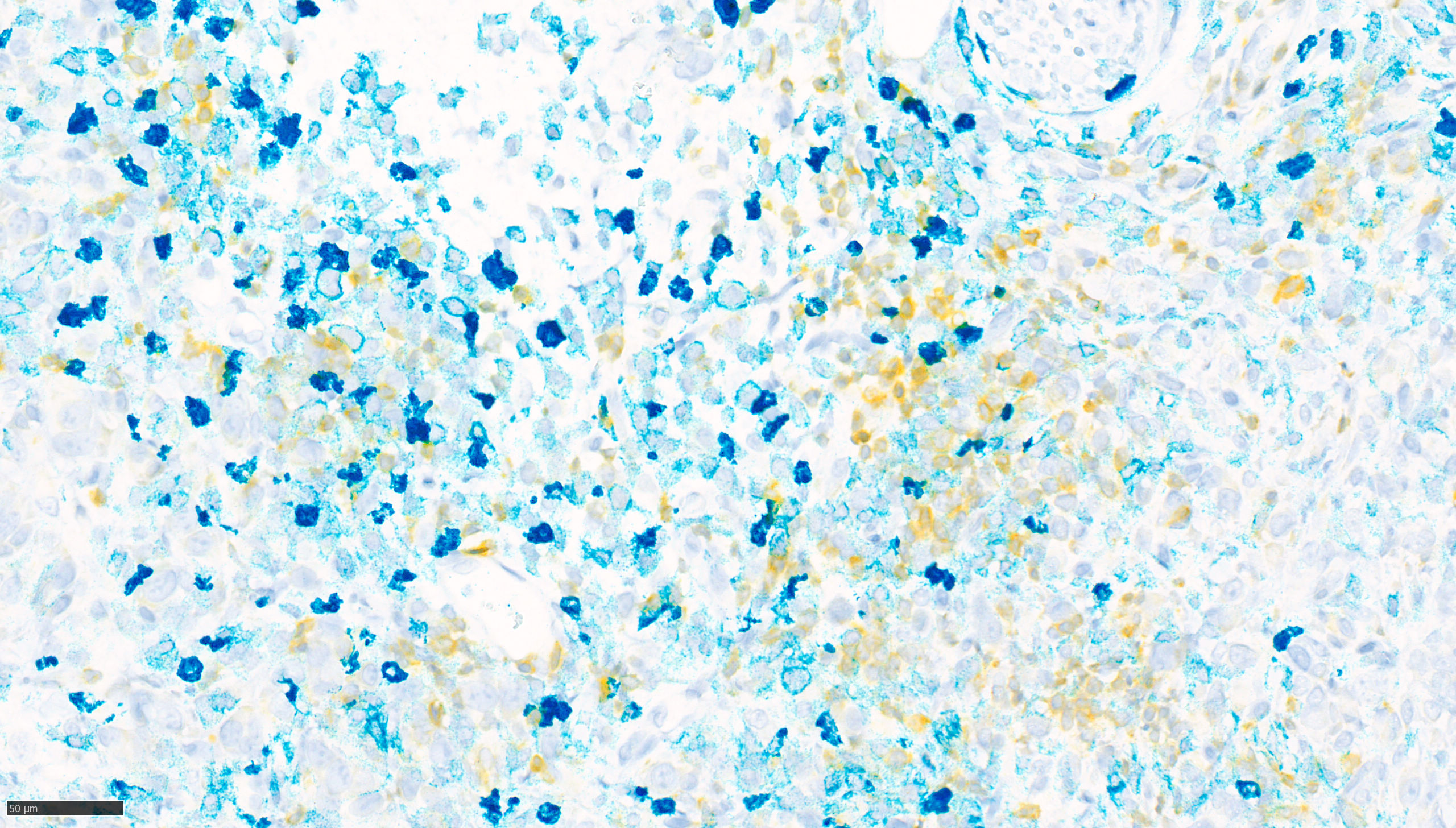

Supplement: Supplementary file 1 [file pharmaceutics-17-01273-s001.zip › IHC/CD3-CD11B/LIFE BIOMATERIAL_CONV-5Gy/C5-L2/C5-L2-1.jpg]

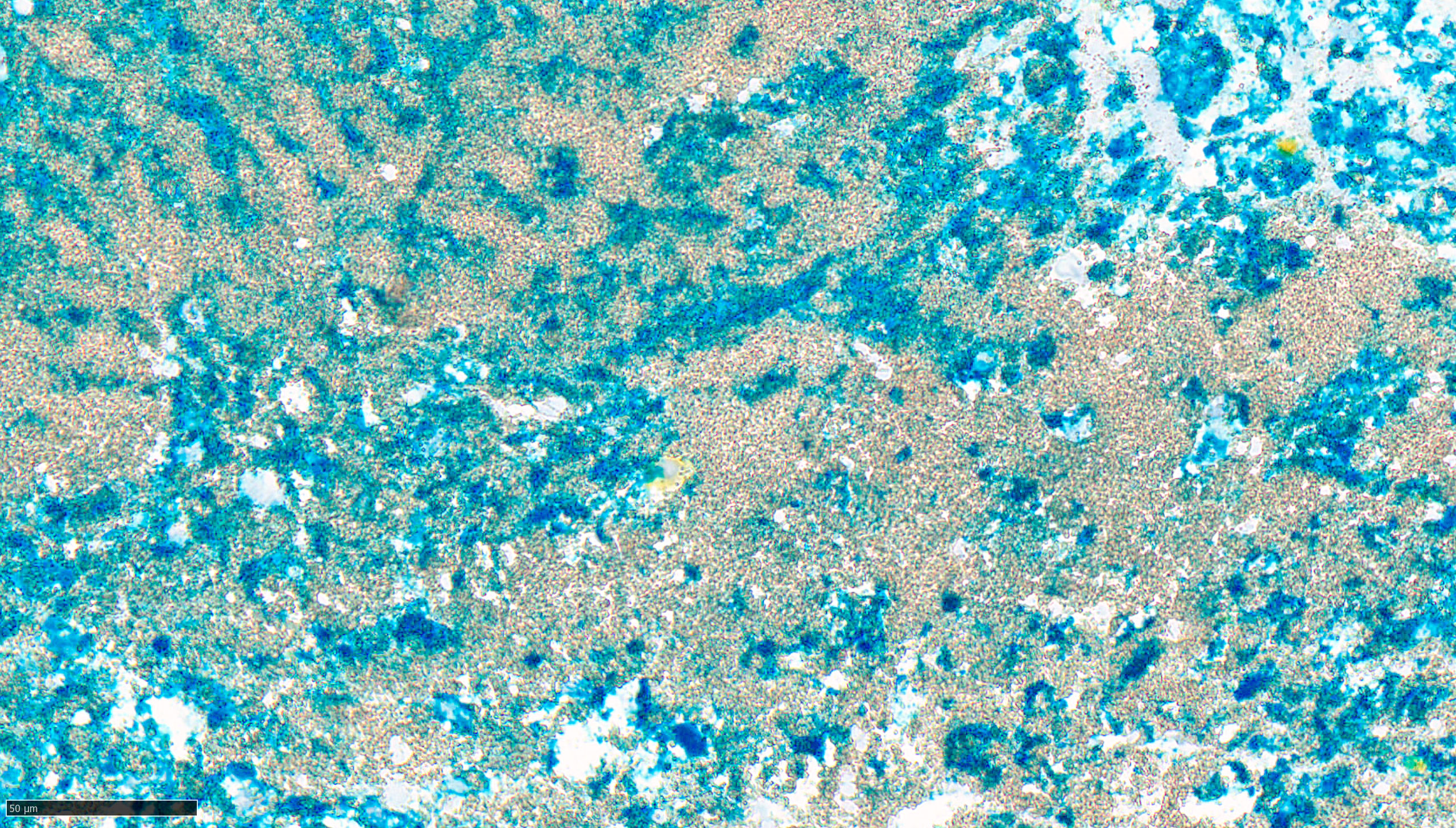

Supplement: Supplementary file 1 [file pharmaceutics-17-01273-s001.zip › IHC/CD3-CD11B/LIFE BIOMATERIAL_CONV-5Gy/C5-L2/C5-L2-2.jpg]

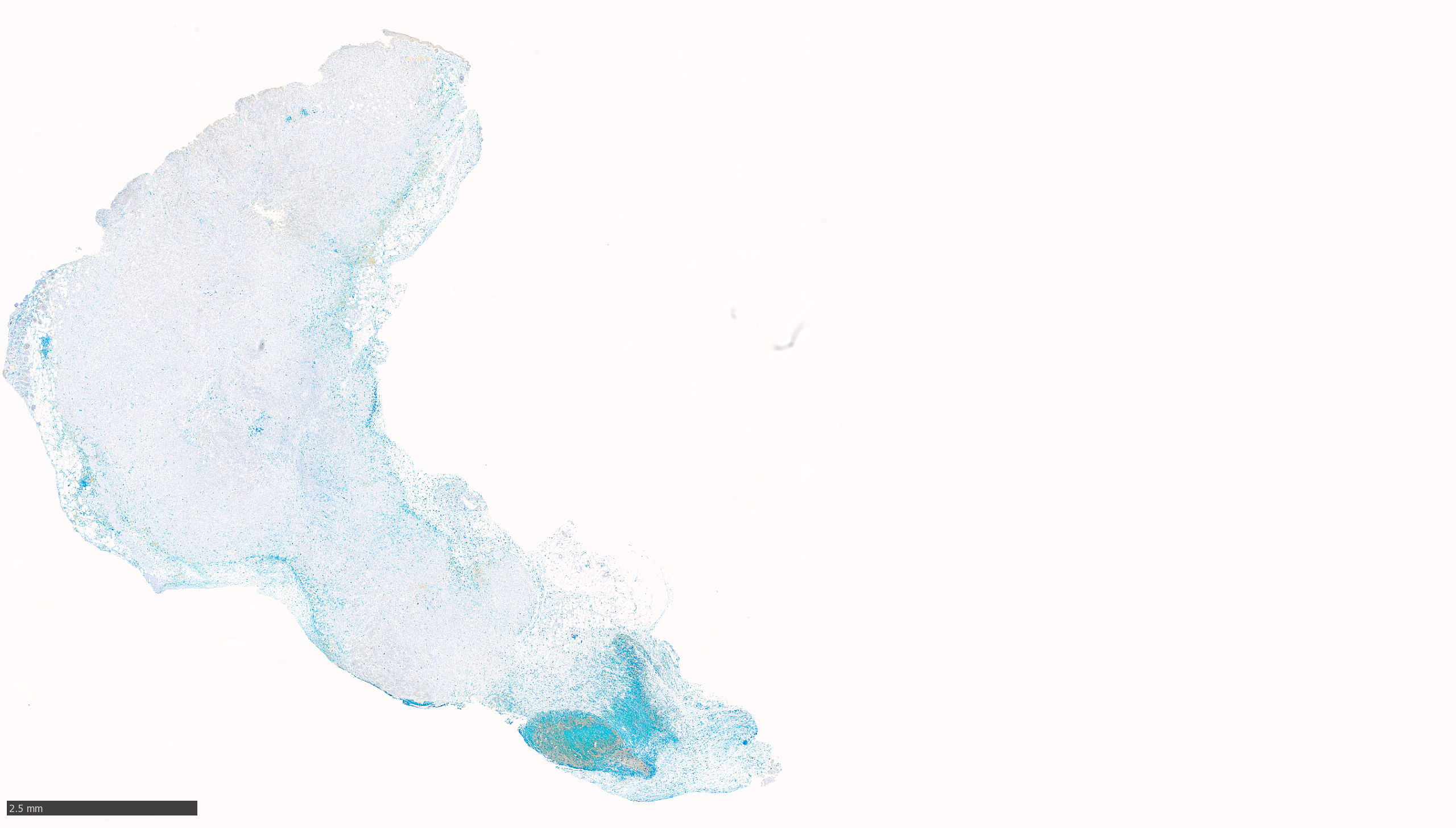

Supplement: Supplementary file 1 [file pharmaceutics-17-01273-s001.zip › IHC/CD3-CD11B/LIFE BIOMATERIAL_CONV-5Gy/C5-L2/C5-L2.jpg]

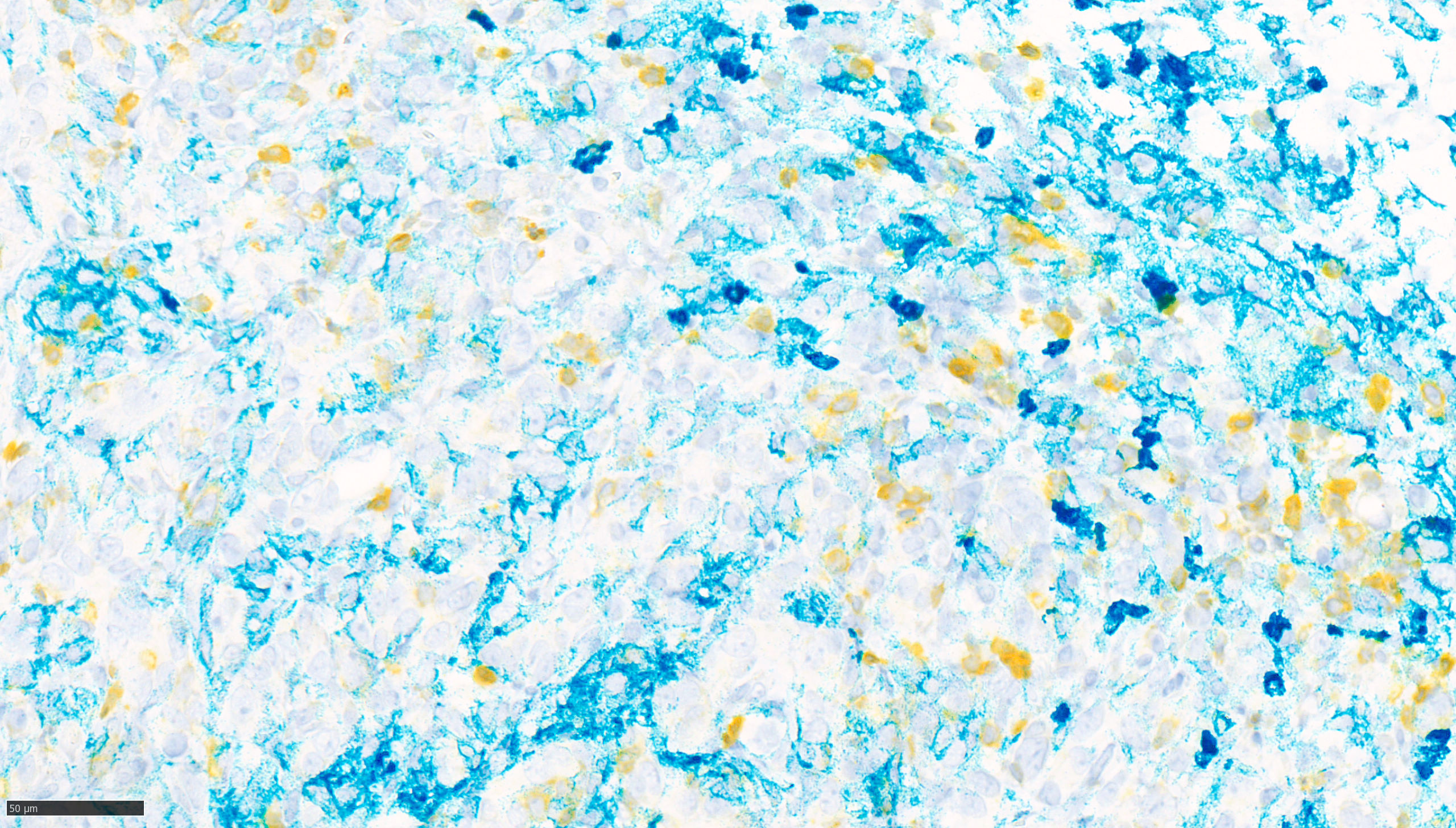

Supplement: Supplementary file 1 [file pharmaceutics-17-01273-s001.zip › IHC/CD3-CD11B/LIFE BIOMATERIAL_CONV-8Gy/C8-L1/C8-L1-1.jpg]

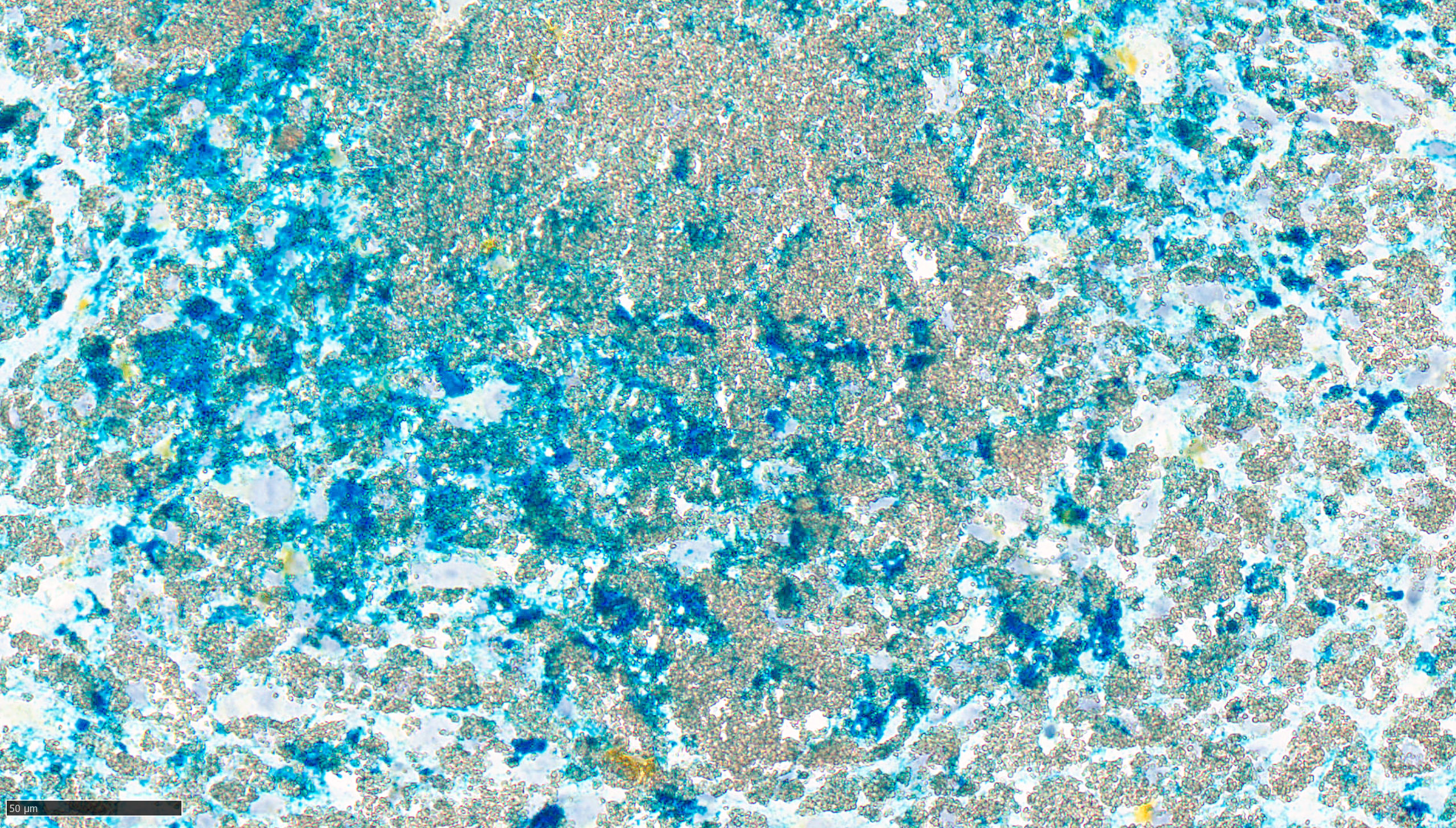

Supplement: Supplementary file 1 [file pharmaceutics-17-01273-s001.zip › IHC/CD3-CD11B/LIFE BIOMATERIAL_CONV-8Gy/C8-L1/C8-L1-2.jpg]

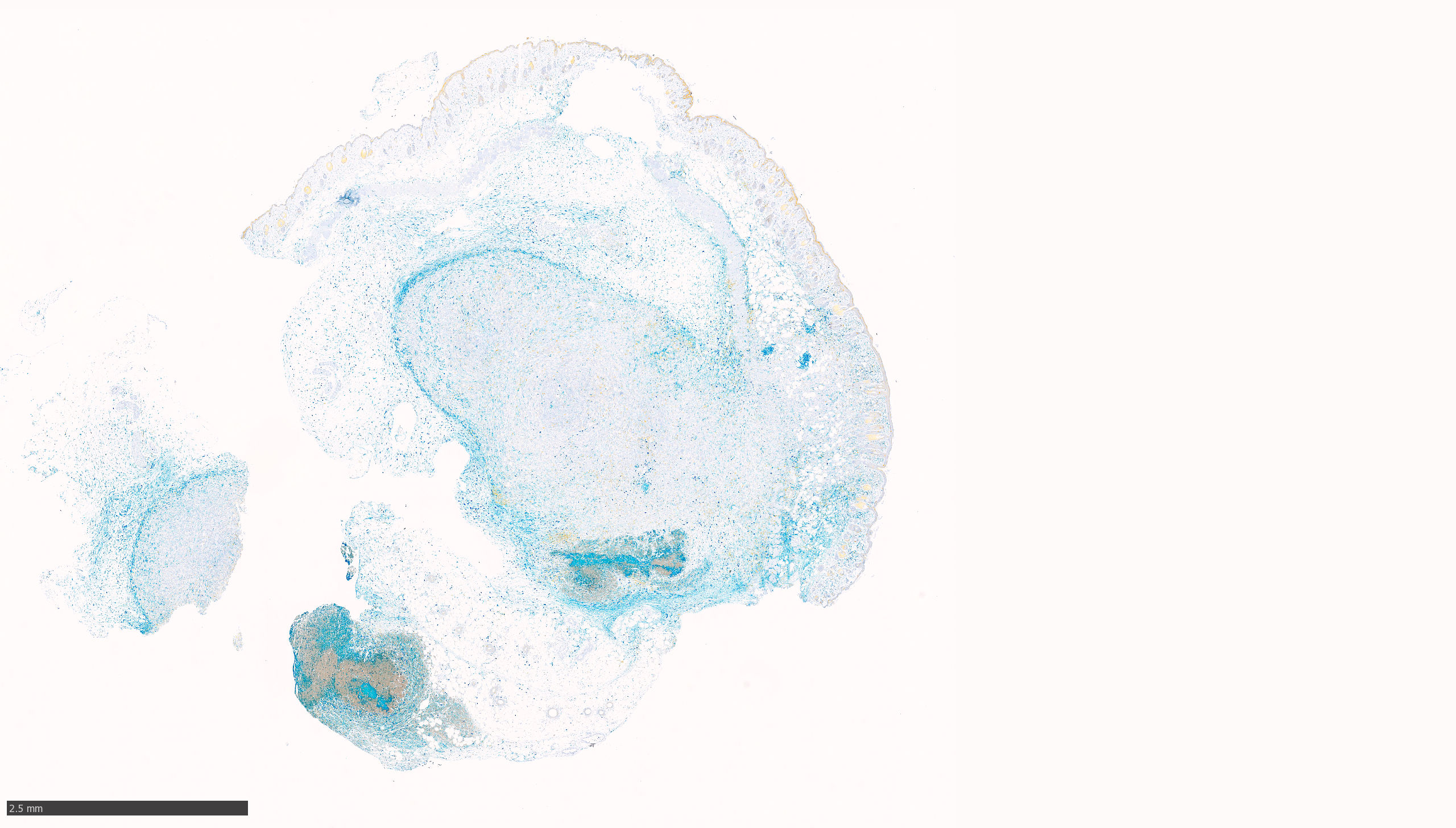

Supplement: Supplementary file 1 [file pharmaceutics-17-01273-s001.zip › IHC/CD3-CD11B/LIFE BIOMATERIAL_CONV-8Gy/C8-L1/C8-L1.jpg]

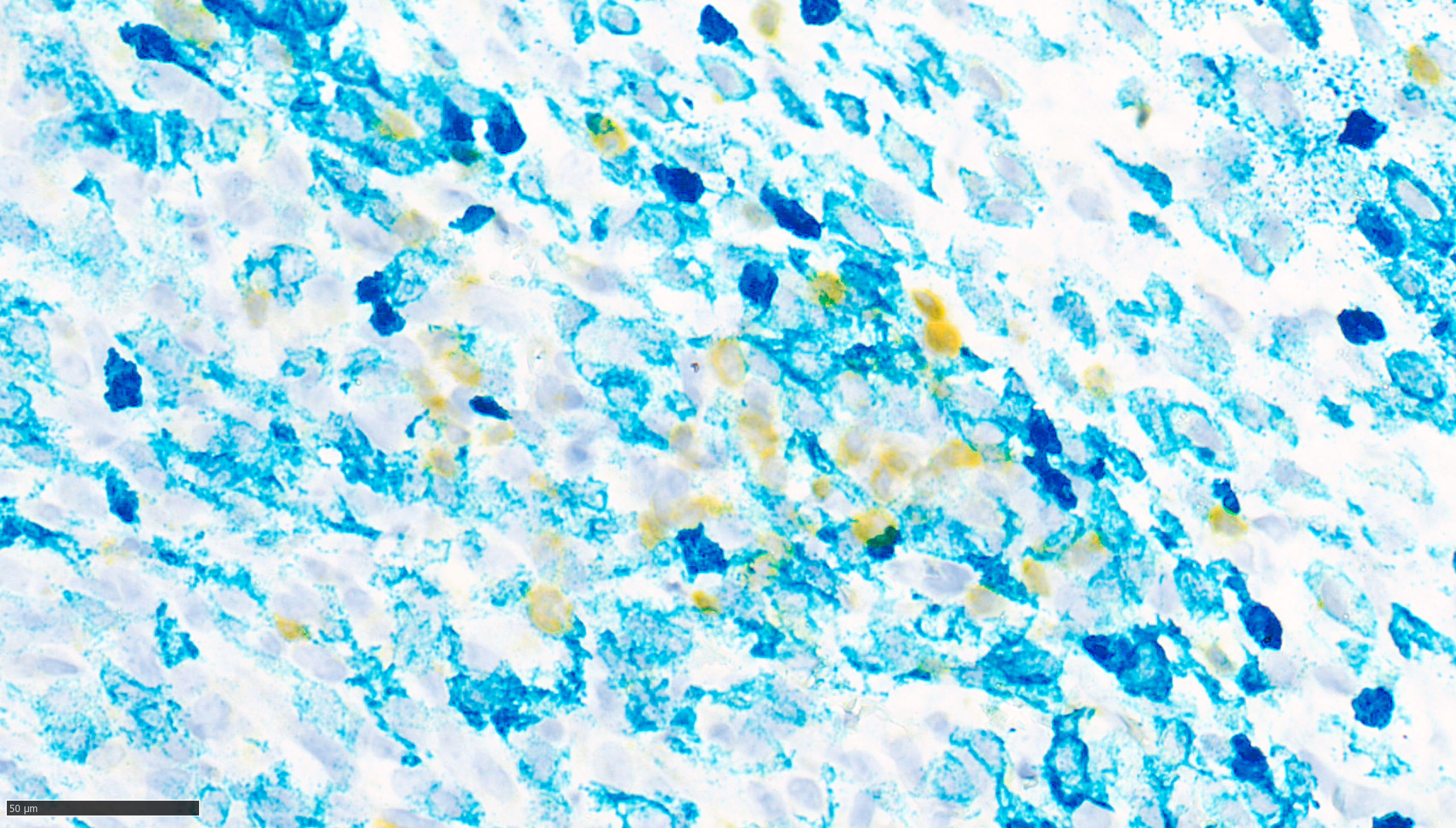

Supplement: Supplementary file 1 [file pharmaceutics-17-01273-s001.zip › IHC/CD3-CD11B/LIFE BIOMATERIAL_CONV-8Gy/C8-L2/C8-L2-1.jpg]

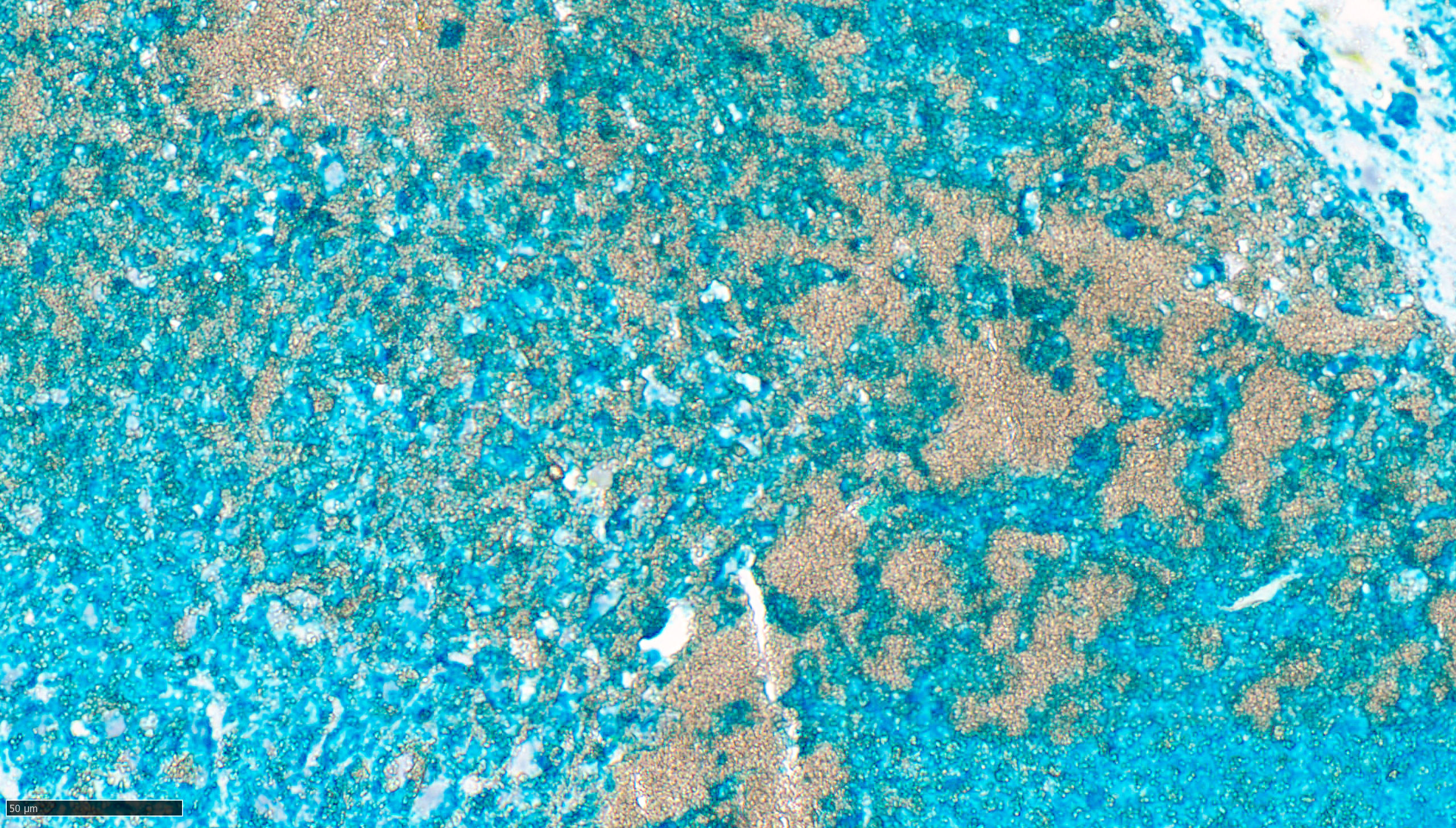

Supplement: Supplementary file 1 [file pharmaceutics-17-01273-s001.zip › IHC/CD3-CD11B/LIFE BIOMATERIAL_CONV-8Gy/C8-L2/C8-L2-2.jpg]

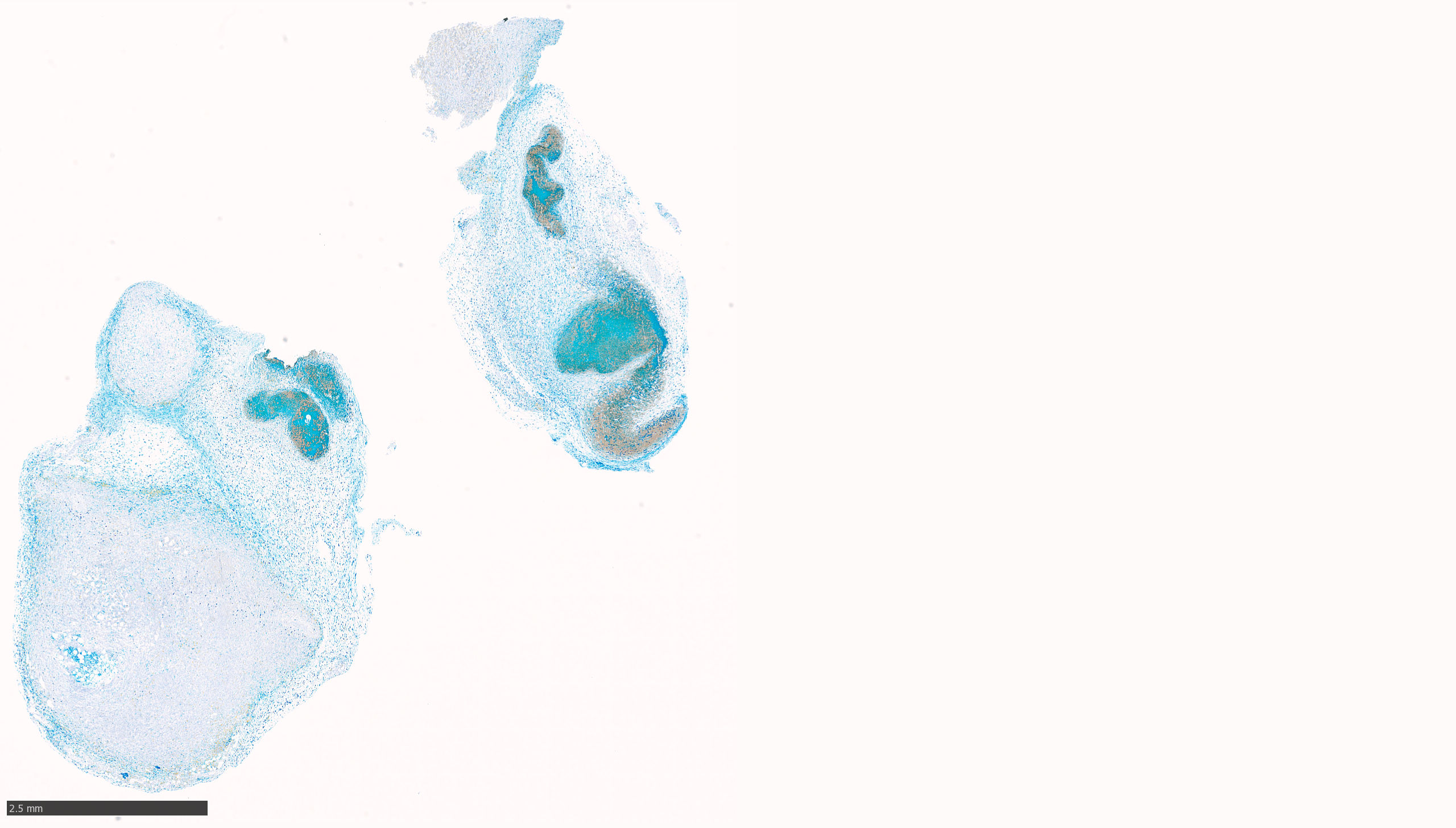

Supplement: Supplementary file 1 [file pharmaceutics-17-01273-s001.zip › IHC/CD3-CD11B/LIFE BIOMATERIAL_CONV-8Gy/C8-L2/C8-L2.jpg]

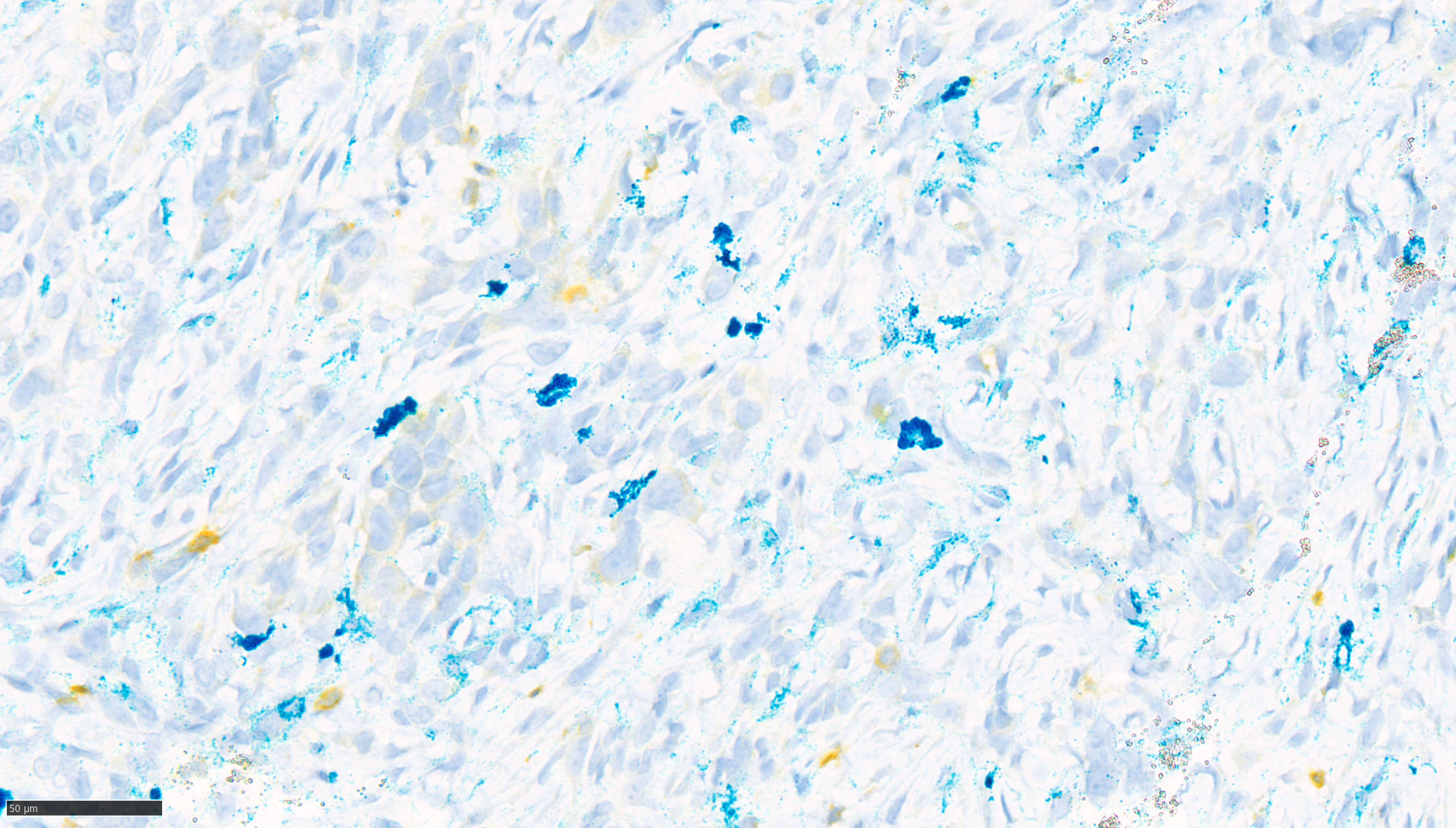

Supplement: Supplementary file 1 [file pharmaceutics-17-01273-s001.zip › IHC/CD3-CD11B/LIFE BIOMATERIAL_FLASH-5Gy/F5-L1/F5-L1-1.jpg]

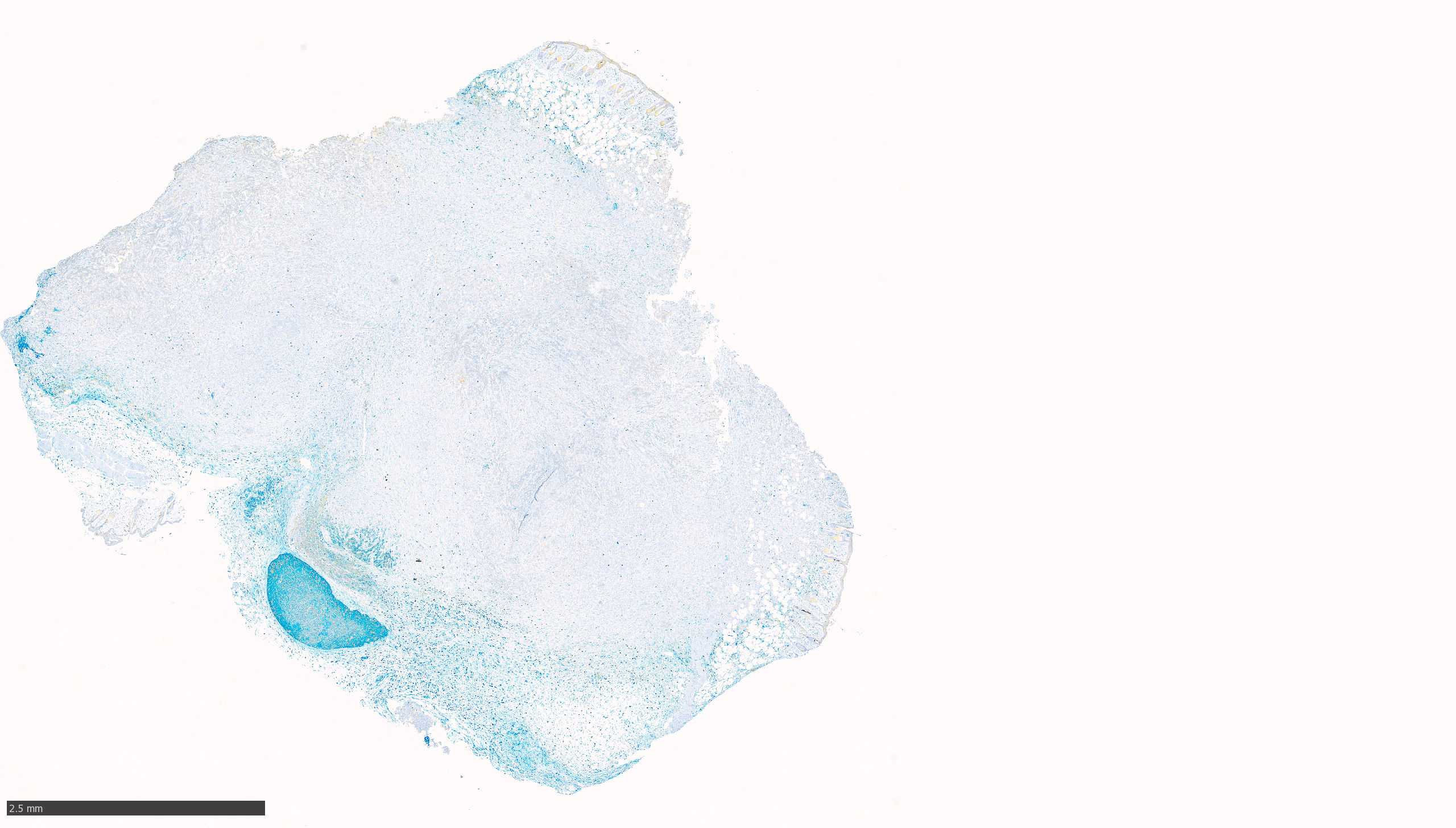

Supplement: Supplementary file 1 [file pharmaceutics-17-01273-s001.zip › IHC/CD3-CD11B/LIFE BIOMATERIAL_FLASH-5Gy/F5-L1/F5-L1.jpg]

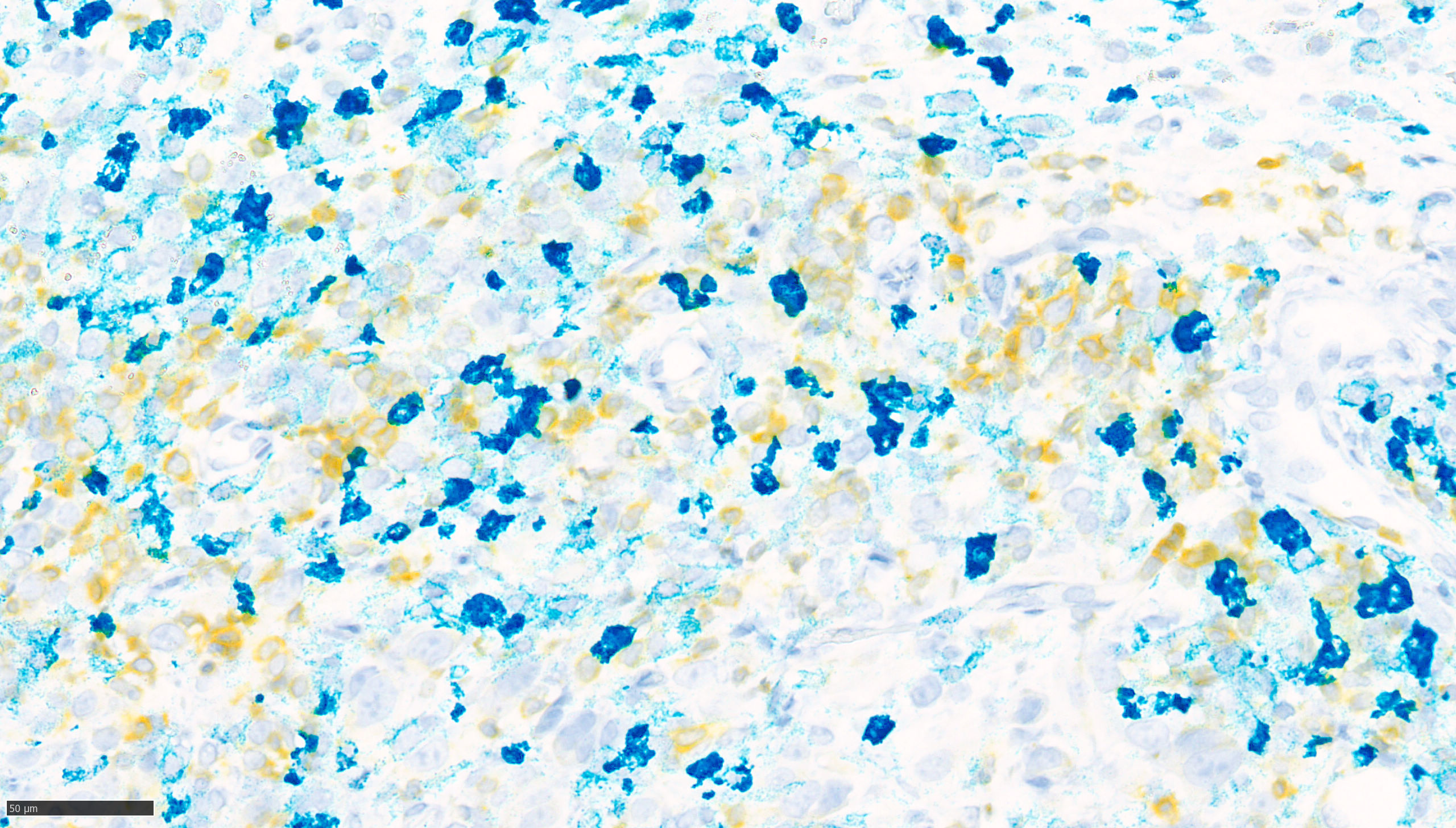

Supplement: Supplementary file 1 [file pharmaceutics-17-01273-s001.zip › IHC/CD3-CD11B/LIFE BIOMATERIAL_FLASH-5Gy/F5-L2/F5-L2-1.jpg]

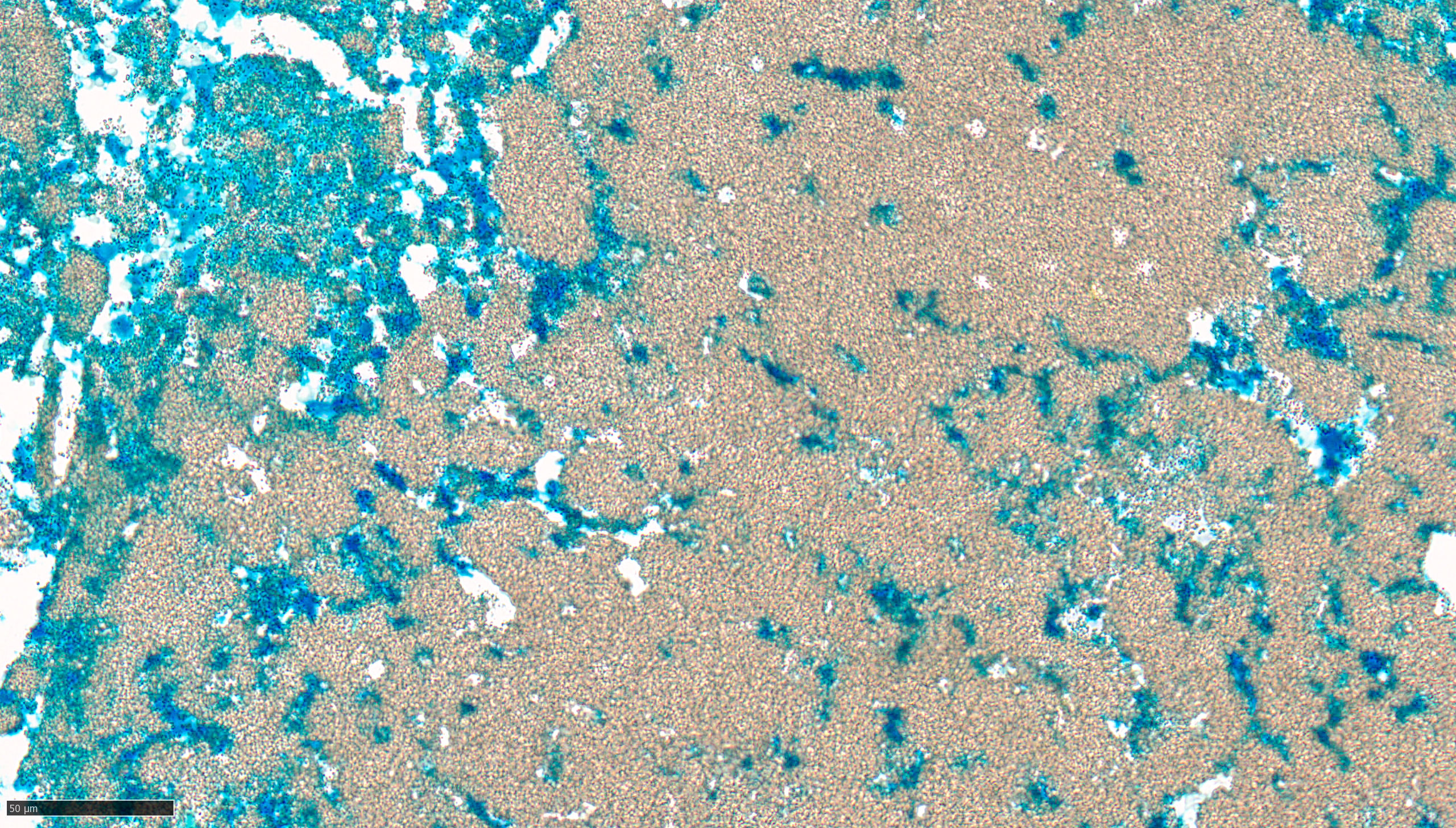

Supplement: Supplementary file 1 [file pharmaceutics-17-01273-s001.zip › IHC/CD3-CD11B/LIFE BIOMATERIAL_FLASH-5Gy/F5-L2/F5-L2-2.jpg]

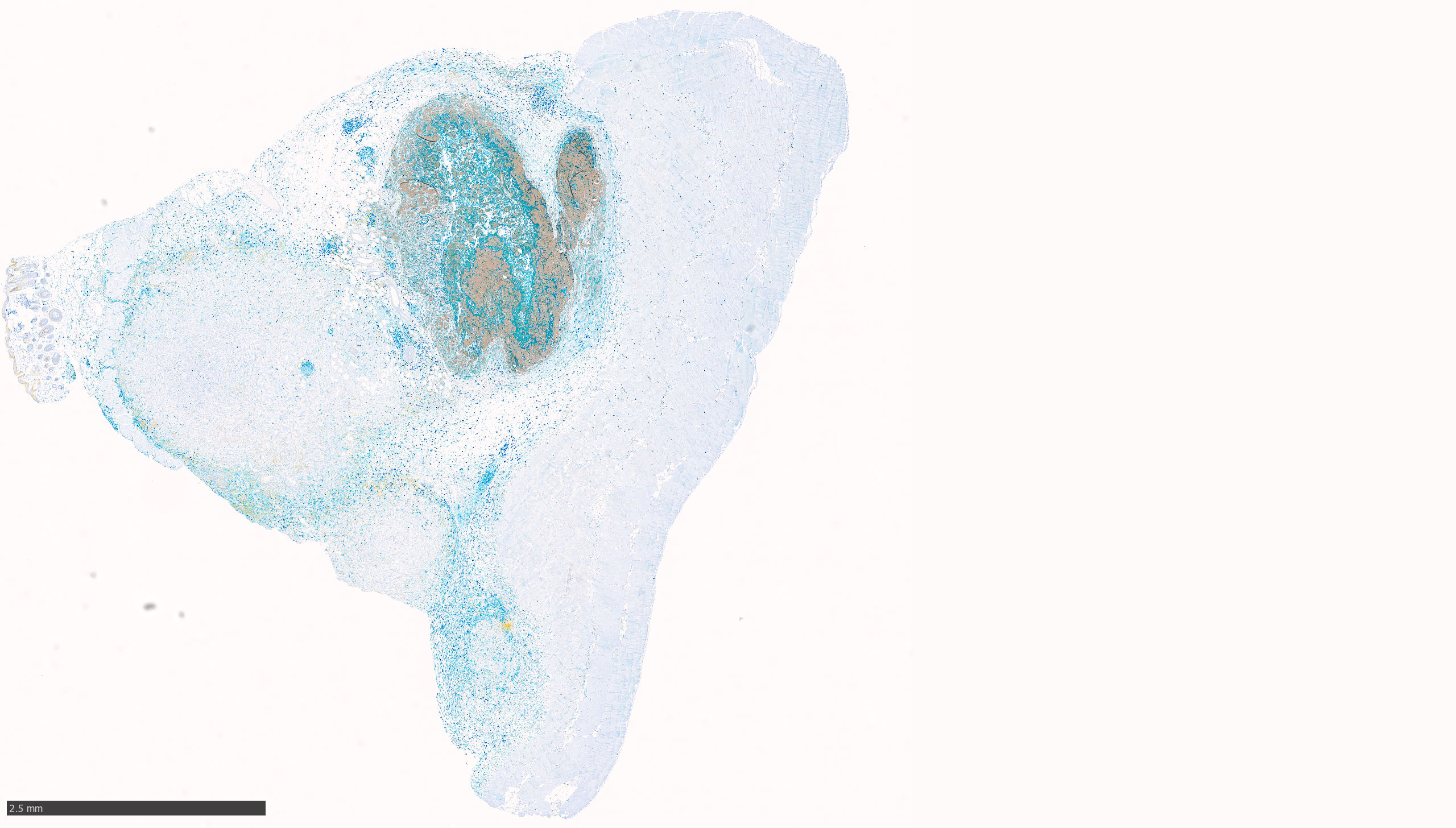

Supplement: Supplementary file 1 [file pharmaceutics-17-01273-s001.zip › IHC/CD3-CD11B/LIFE BIOMATERIAL_FLASH-5Gy/F5-L2/F5-L2.jpg]

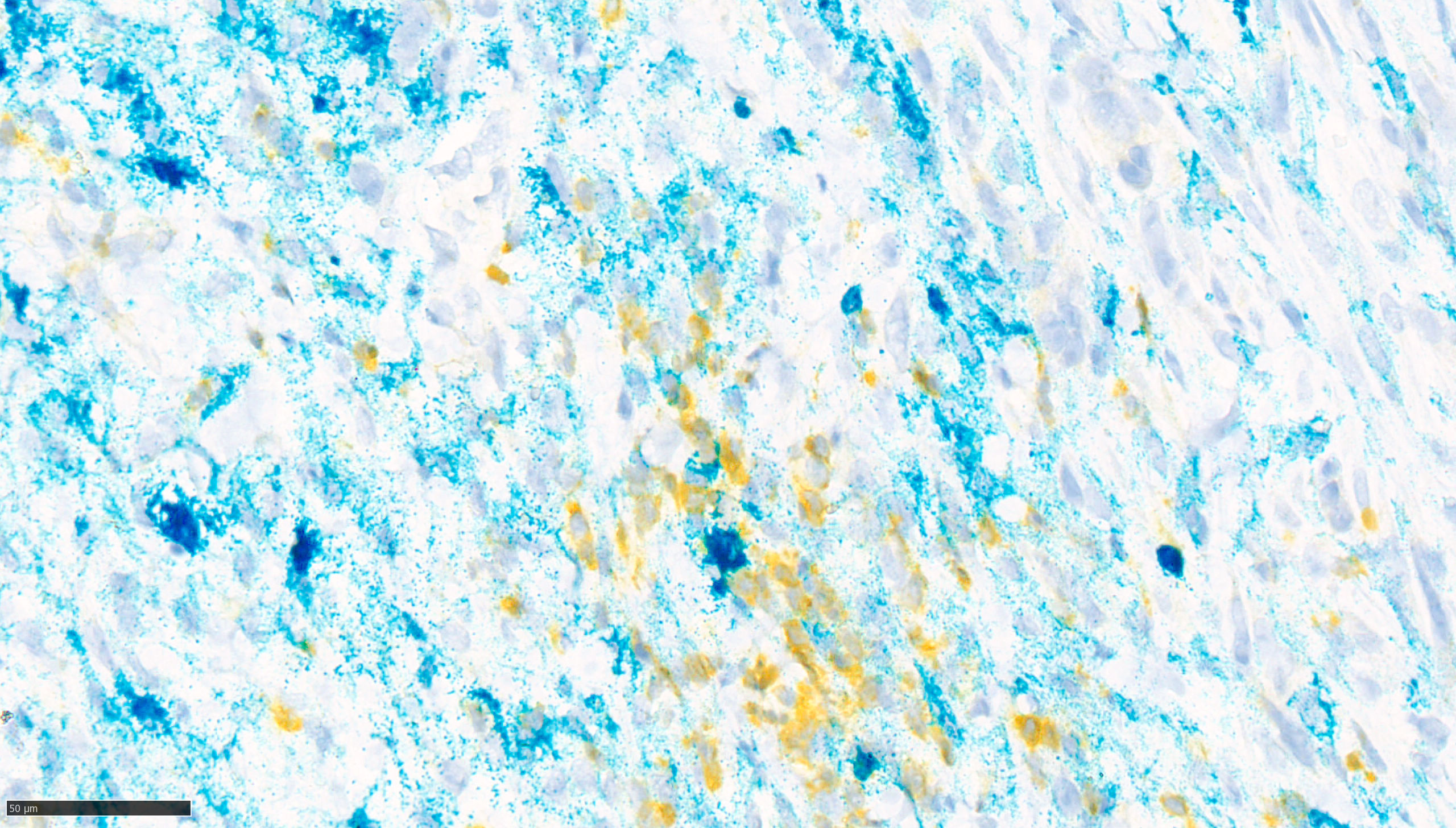

Supplement: Supplementary file 1 [file pharmaceutics-17-01273-s001.zip › IHC/CD3-CD11B/LIFE BIOMATERIAL_FLASH-8Gy/F8-L1/F8-L1-1.jpg]

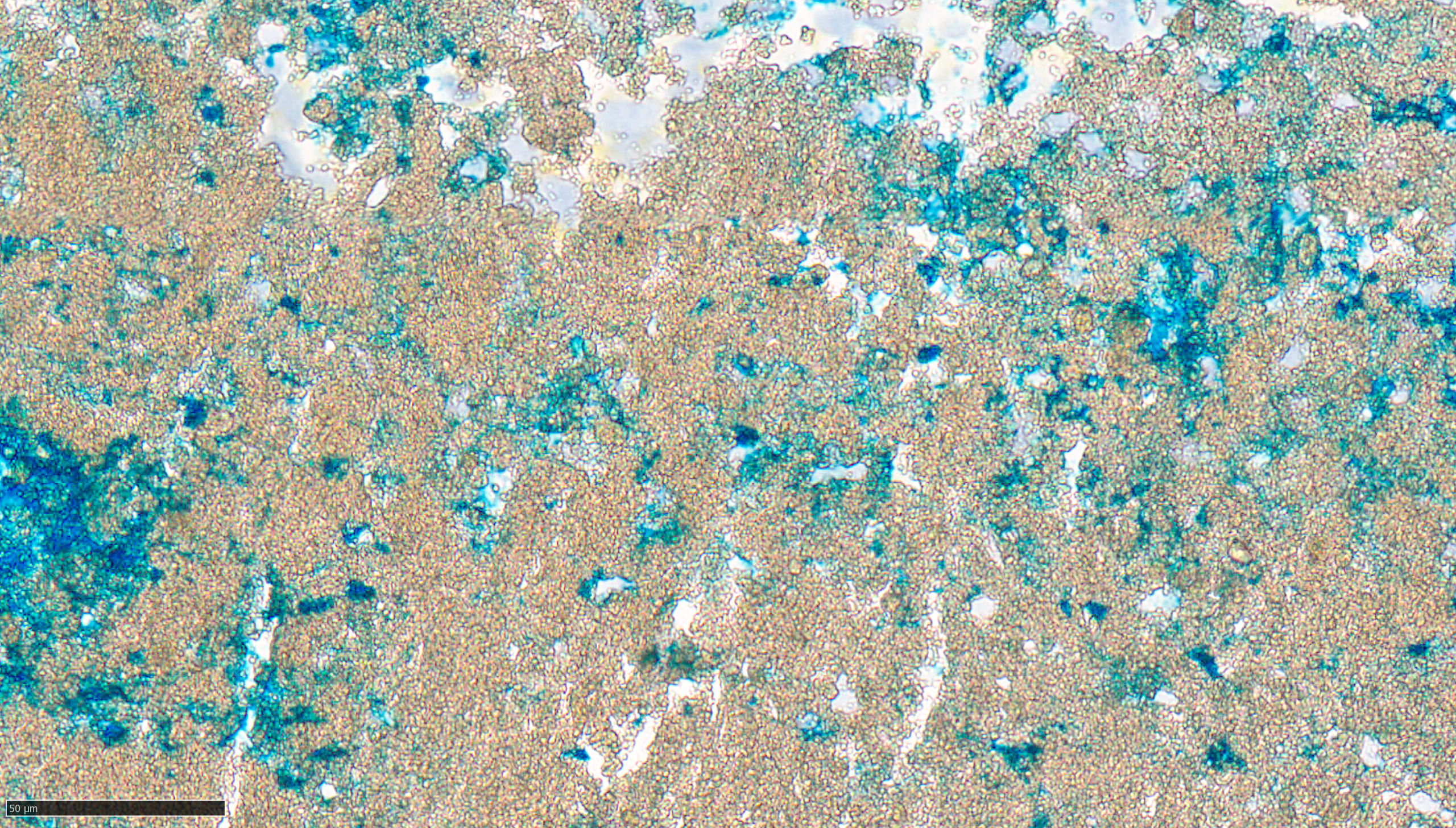

Supplement: Supplementary file 1 [file pharmaceutics-17-01273-s001.zip › IHC/CD3-CD11B/LIFE BIOMATERIAL_FLASH-8Gy/F8-L1/F8-L1-2.jpg]

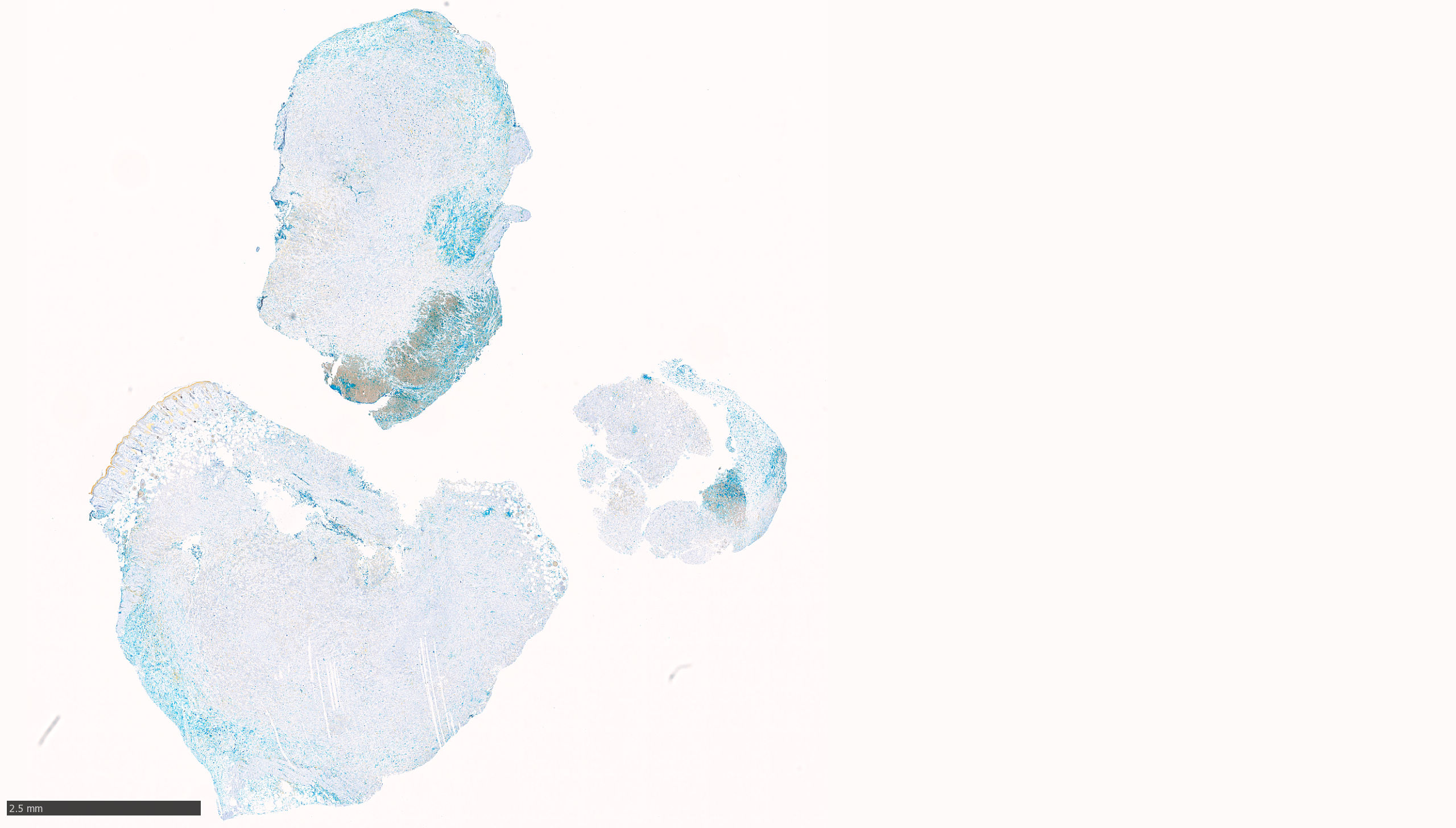

Supplement: Supplementary file 1 [file pharmaceutics-17-01273-s001.zip › IHC/CD3-CD11B/LIFE BIOMATERIAL_FLASH-8Gy/F8-L1/F8-L1.jpg]

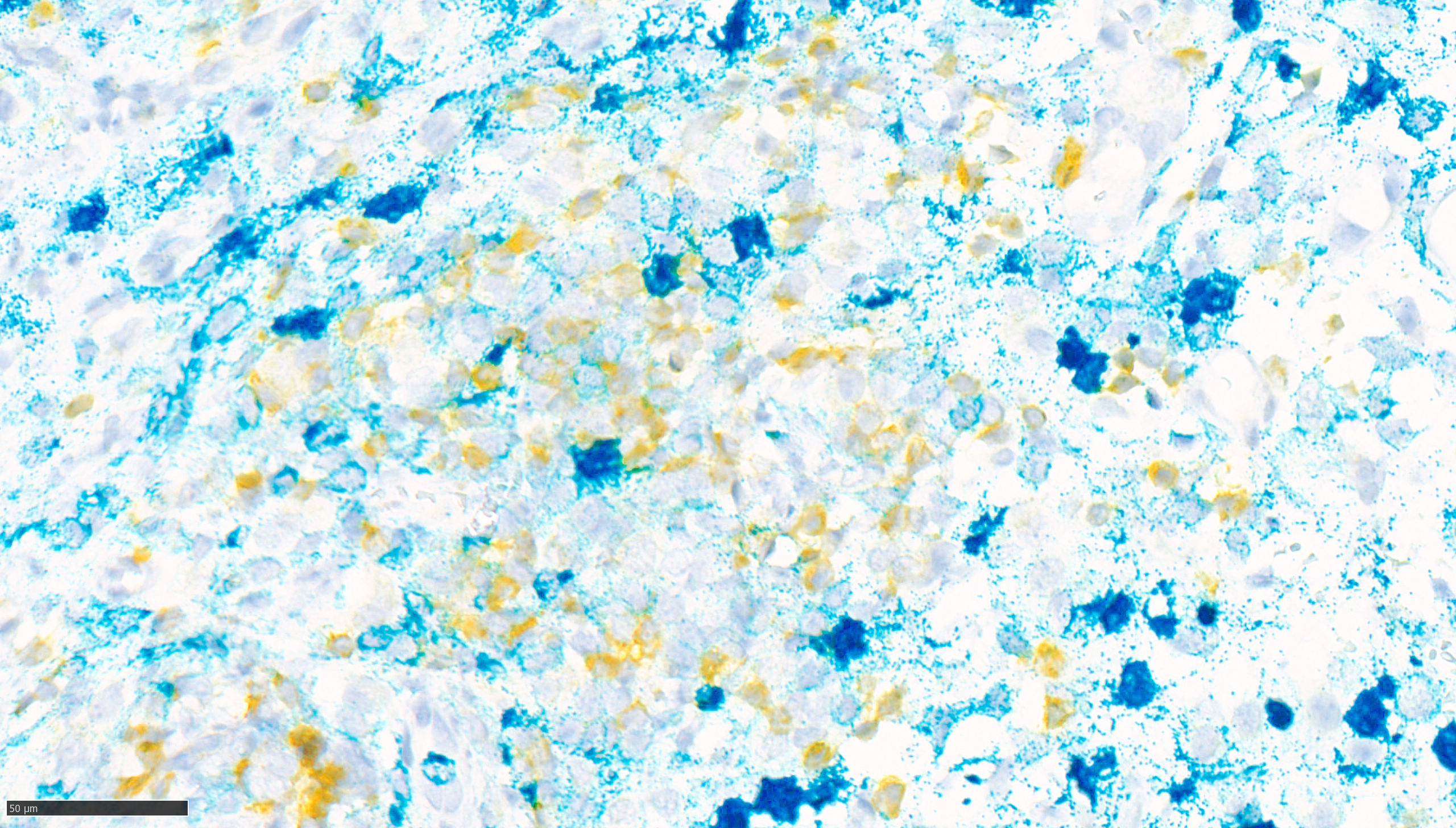

Supplement: Supplementary file 1 [file pharmaceutics-17-01273-s001.zip › IHC/CD3-CD11B/LIFE BIOMATERIAL_FLASH-8Gy/F8-L2/F8-L2-1.jpg]

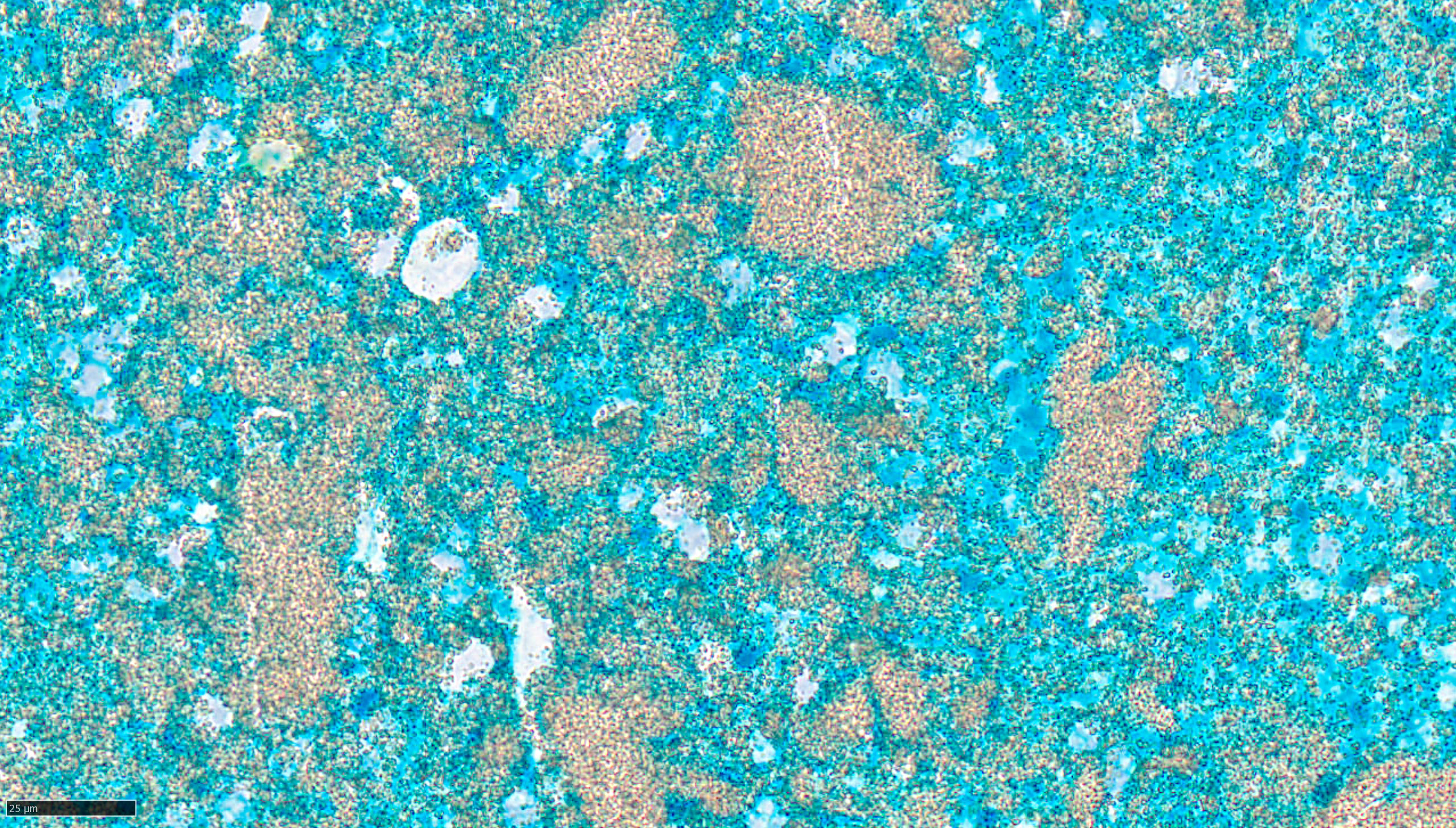

Supplement: Supplementary file 1 [file pharmaceutics-17-01273-s001.zip › IHC/CD3-CD11B/LIFE BIOMATERIAL_FLASH-8Gy/F8-L2/F8-L2-2.jpg]

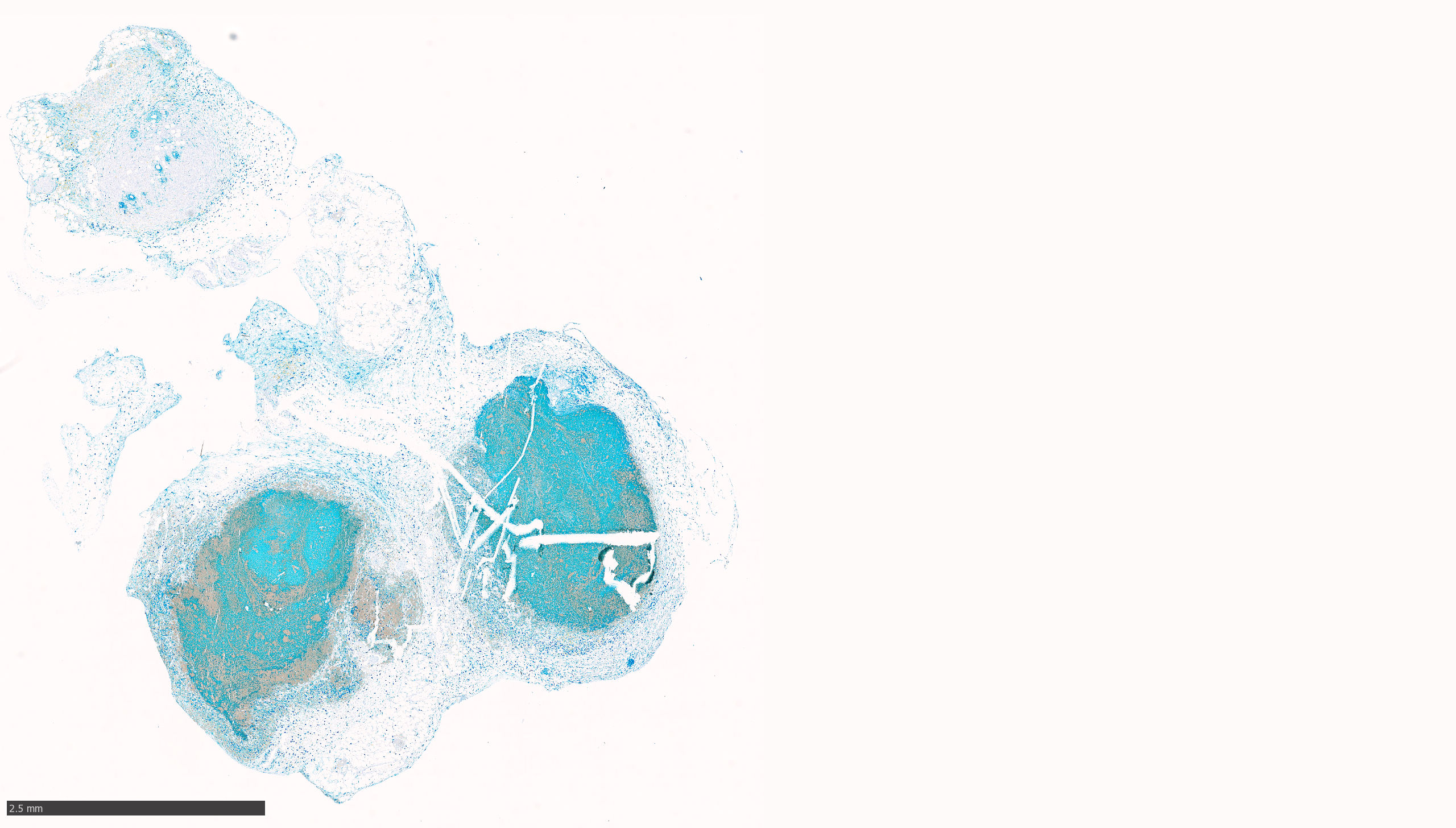

Supplement: Supplementary file 1 [file pharmaceutics-17-01273-s001.zip › IHC/CD3-CD11B/LIFE BIOMATERIAL_FLASH-8Gy/F8-L2/F8-L2.jpg]

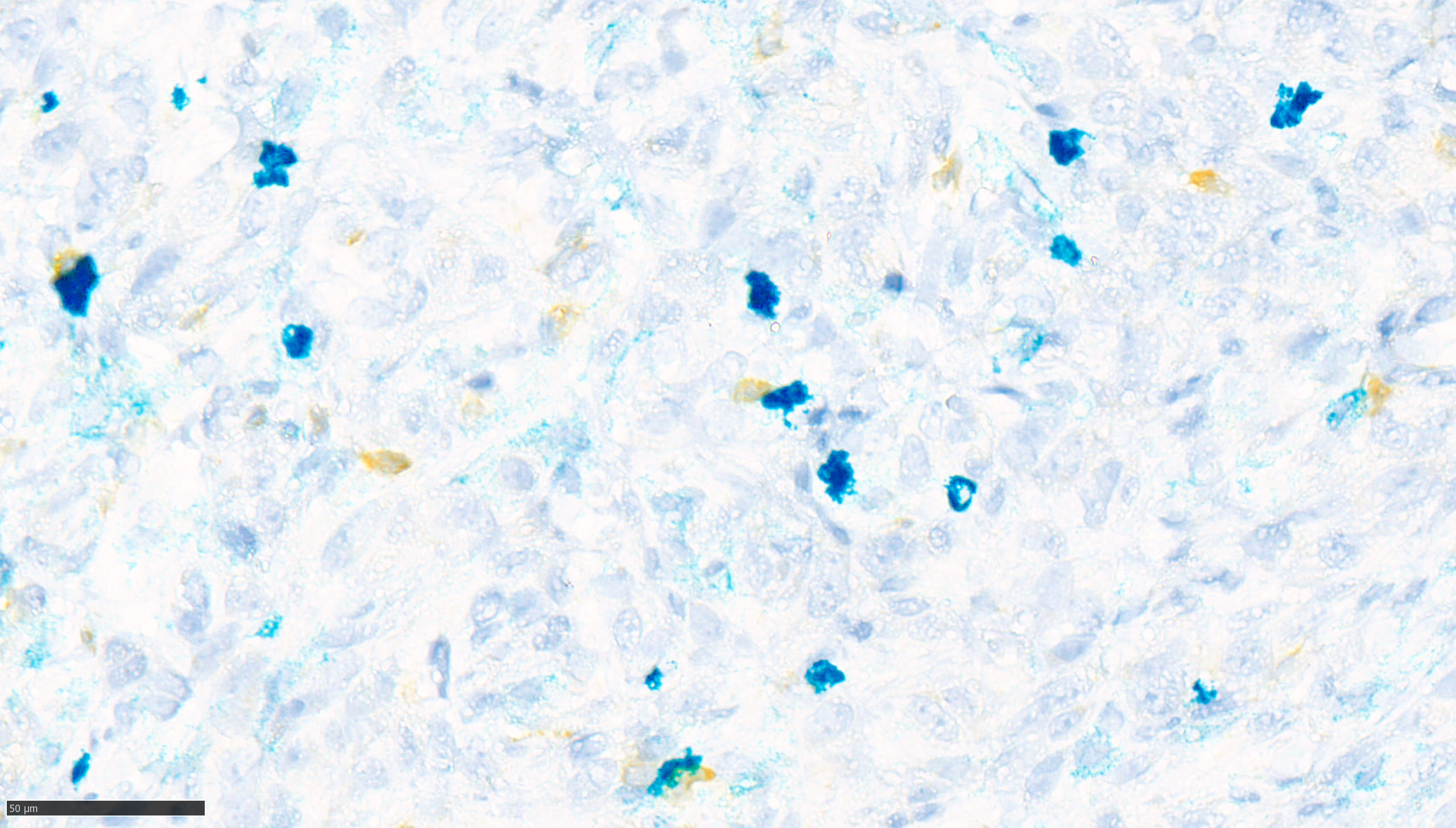

Supplement: Supplementary file 1 [file pharmaceutics-17-01273-s001.zip › IHC/CD3-CD11B/NO TREATMENT/N1/N1-1.jpg]

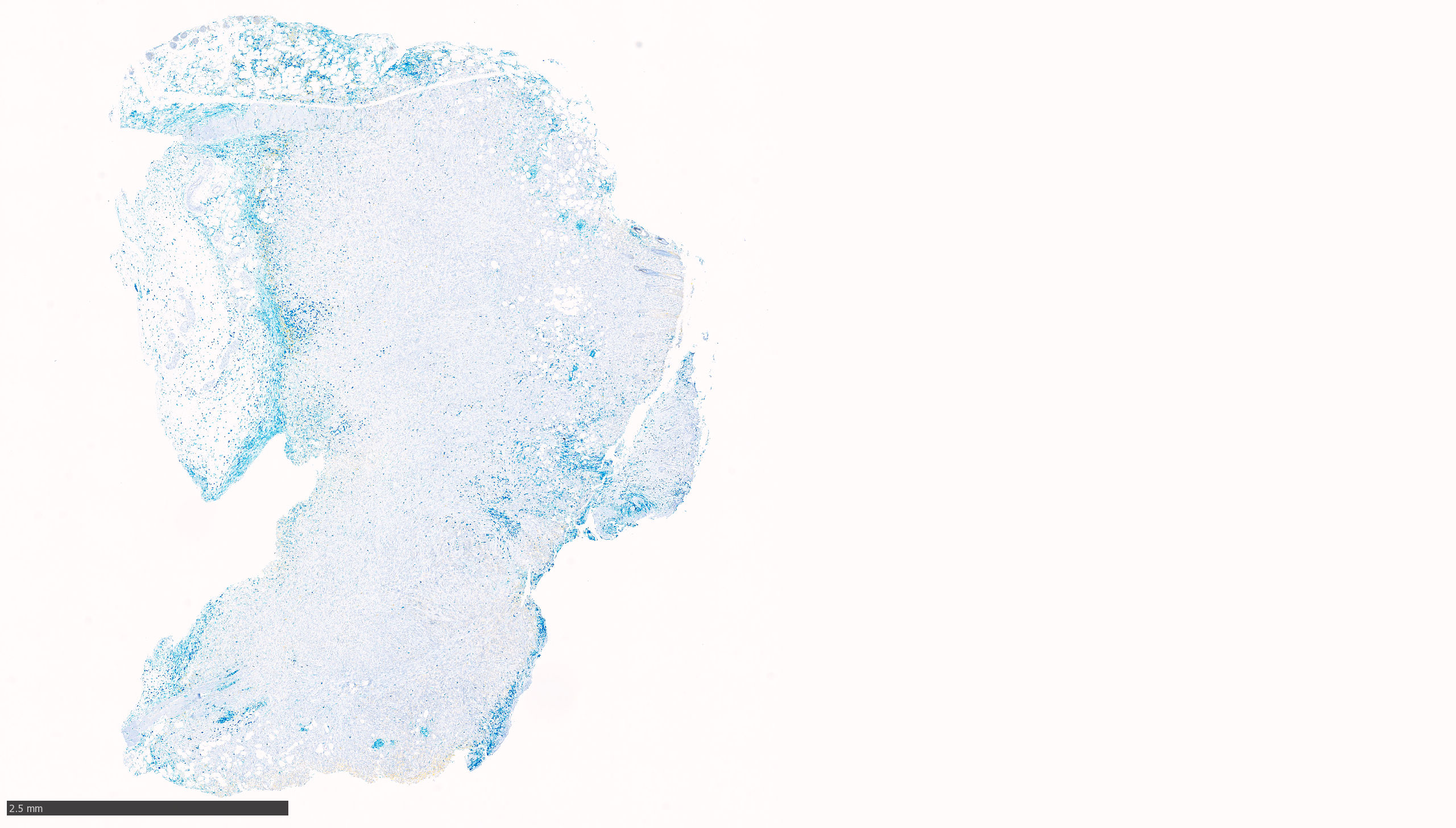

Supplement: Supplementary file 1 [file pharmaceutics-17-01273-s001.zip › IHC/CD3-CD11B/NO TREATMENT/N1/N1.jpg]

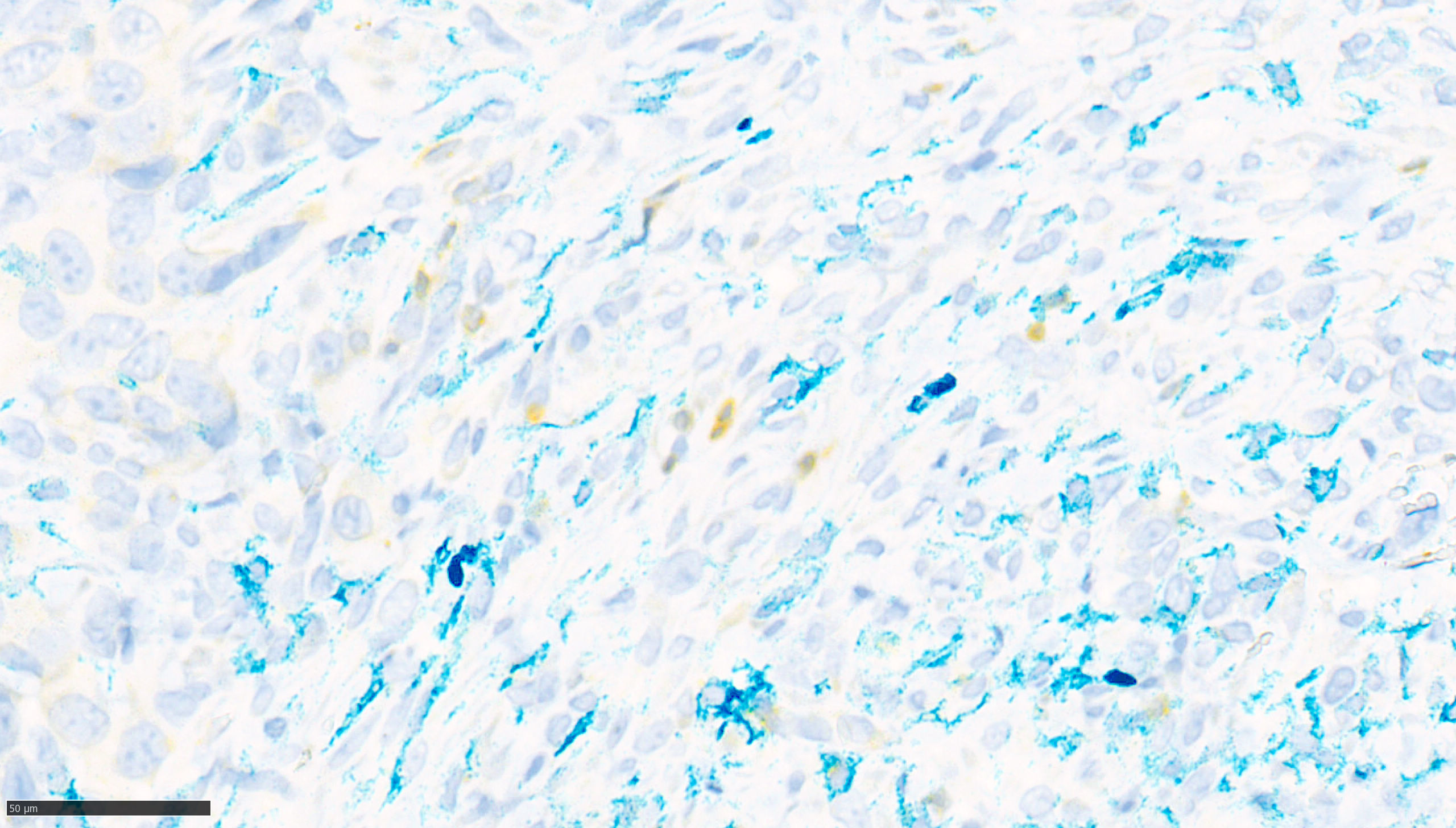

Supplement: Supplementary file 1 [file pharmaceutics-17-01273-s001.zip › IHC/CD3-CD11B/NO TREATMENT/N2/N2-1.jpg]

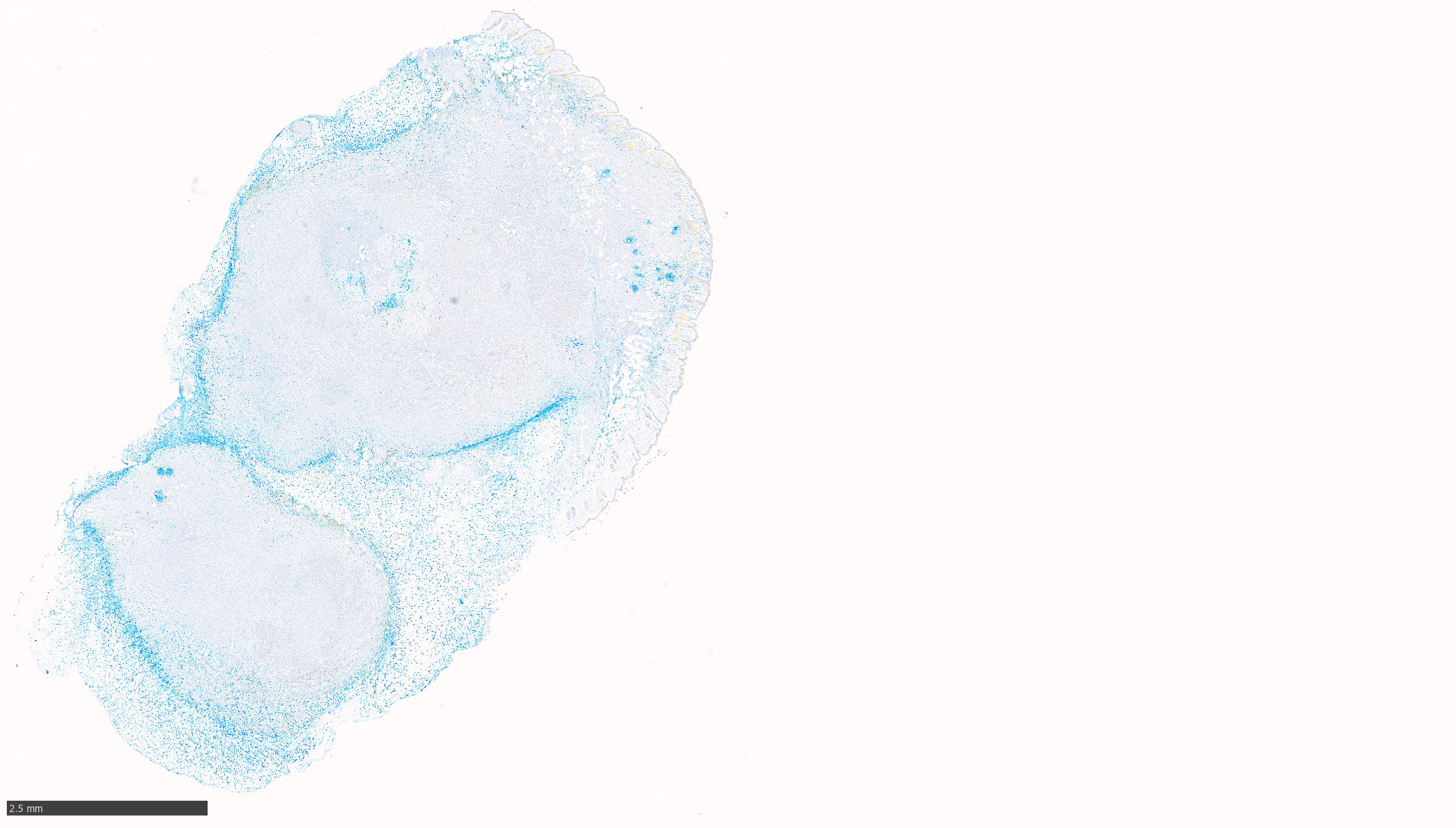

Supplement: Supplementary file 1 [file pharmaceutics-17-01273-s001.zip › IHC/CD3-CD11B/NO TREATMENT/N2/N2.jpg]

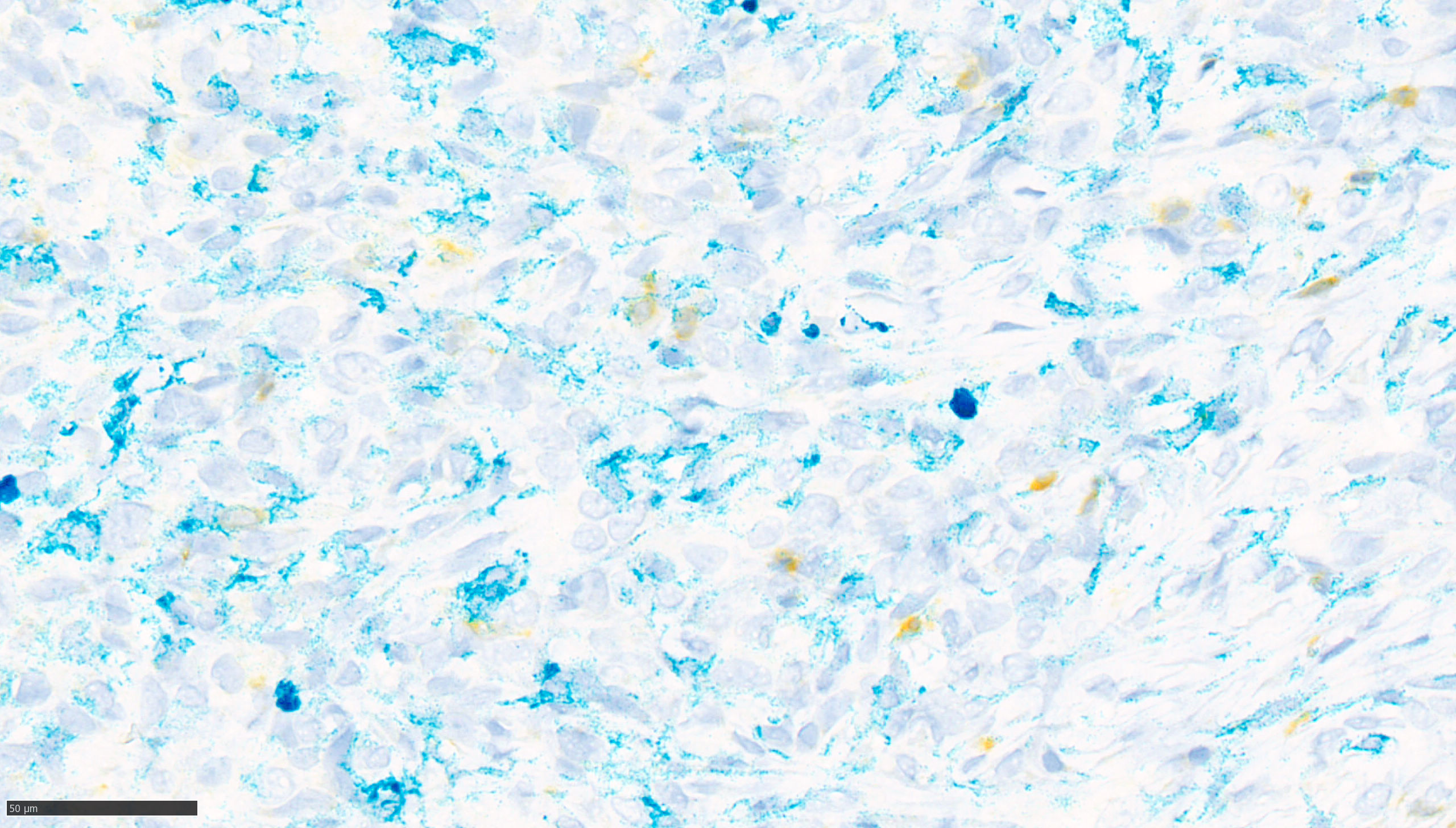

Supplement: Supplementary file 1 [file pharmaceutics-17-01273-s001.zip › IHC/CD3-CD11B/NO TREATMENT/N3/N3-1.jpg]

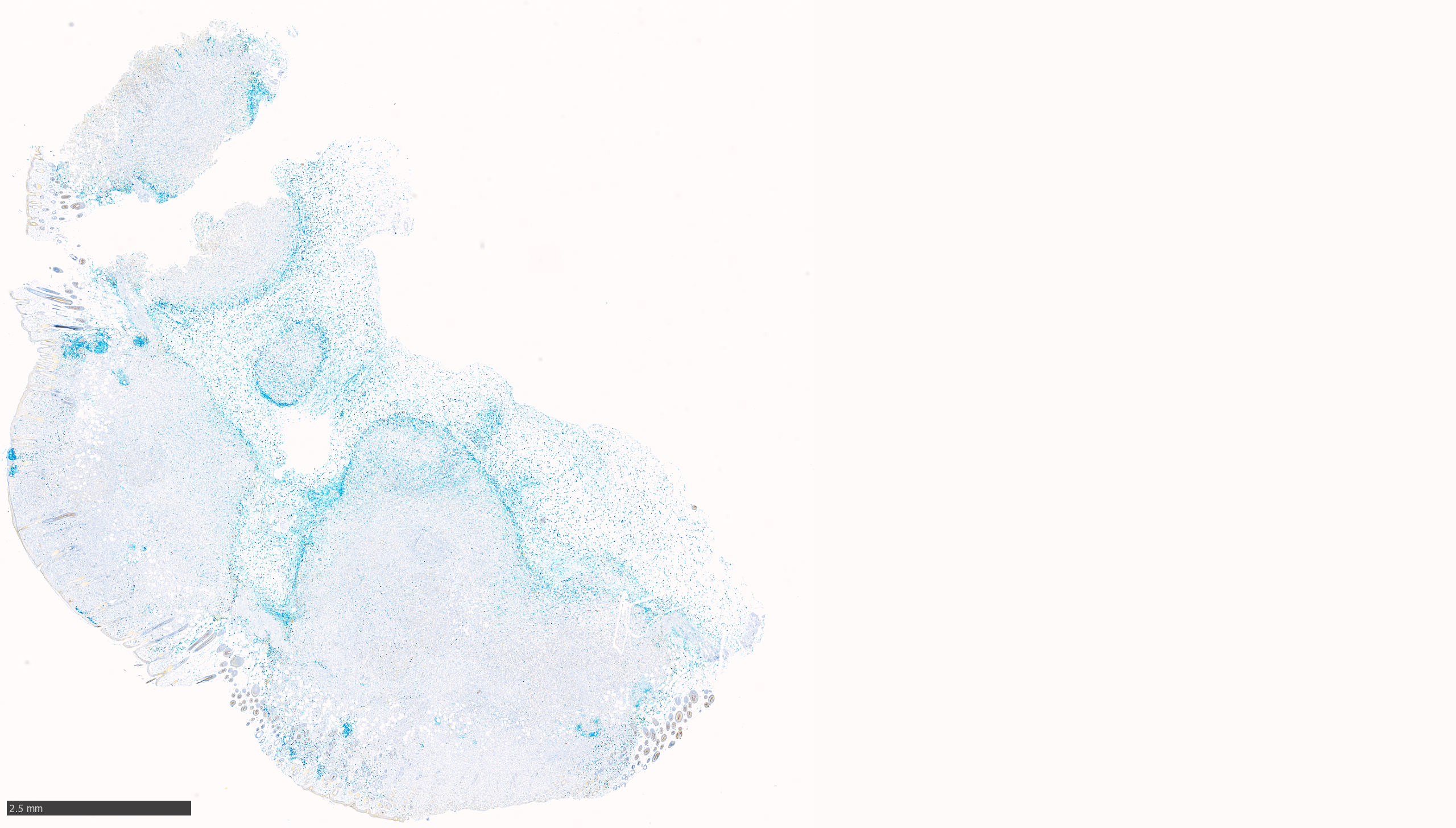

Supplement: Supplementary file 1 [file pharmaceutics-17-01273-s001.zip › IHC/CD3-CD11B/NO TREATMENT/N3/N3.jpg]

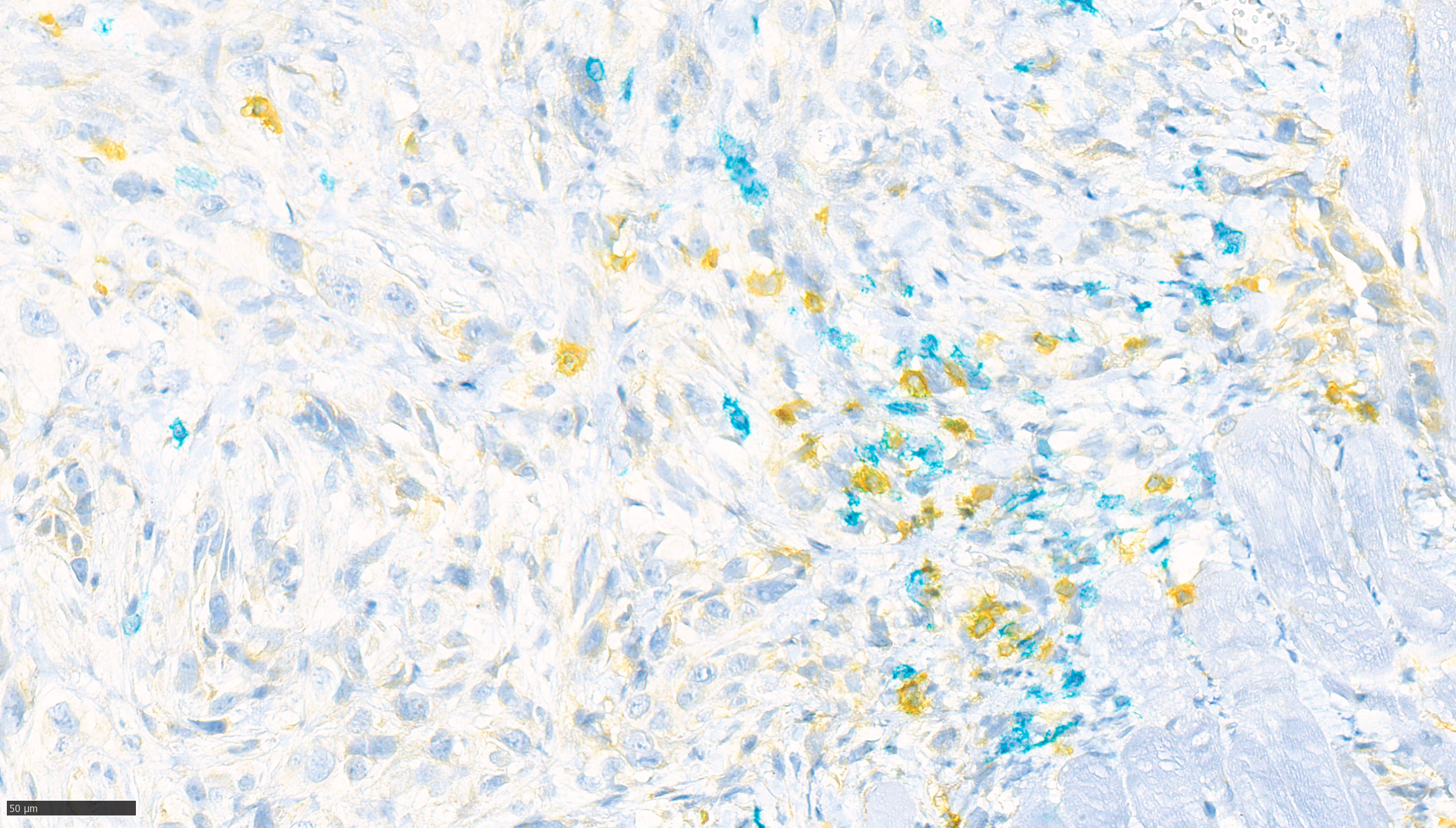

Supplement: Supplementary file 1 [file pharmaceutics-17-01273-s001.zip › IHC/CD4-CD8/CONV-5Gy/C5-1/C5-1-1.jpg]

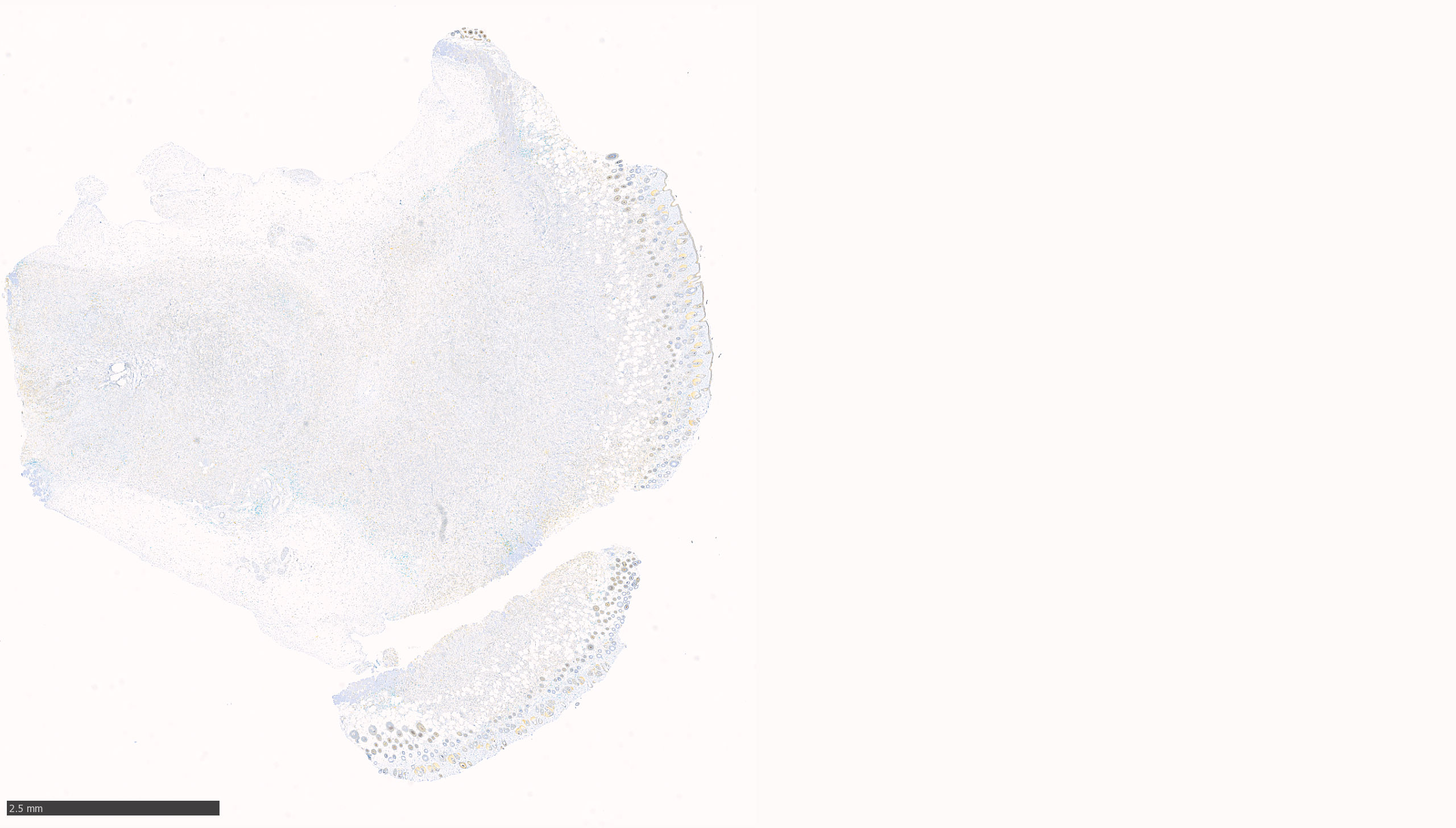

Supplement: Supplementary file 1 [file pharmaceutics-17-01273-s001.zip › IHC/CD4-CD8/CONV-5Gy/C5-1/C5-1.jpg]

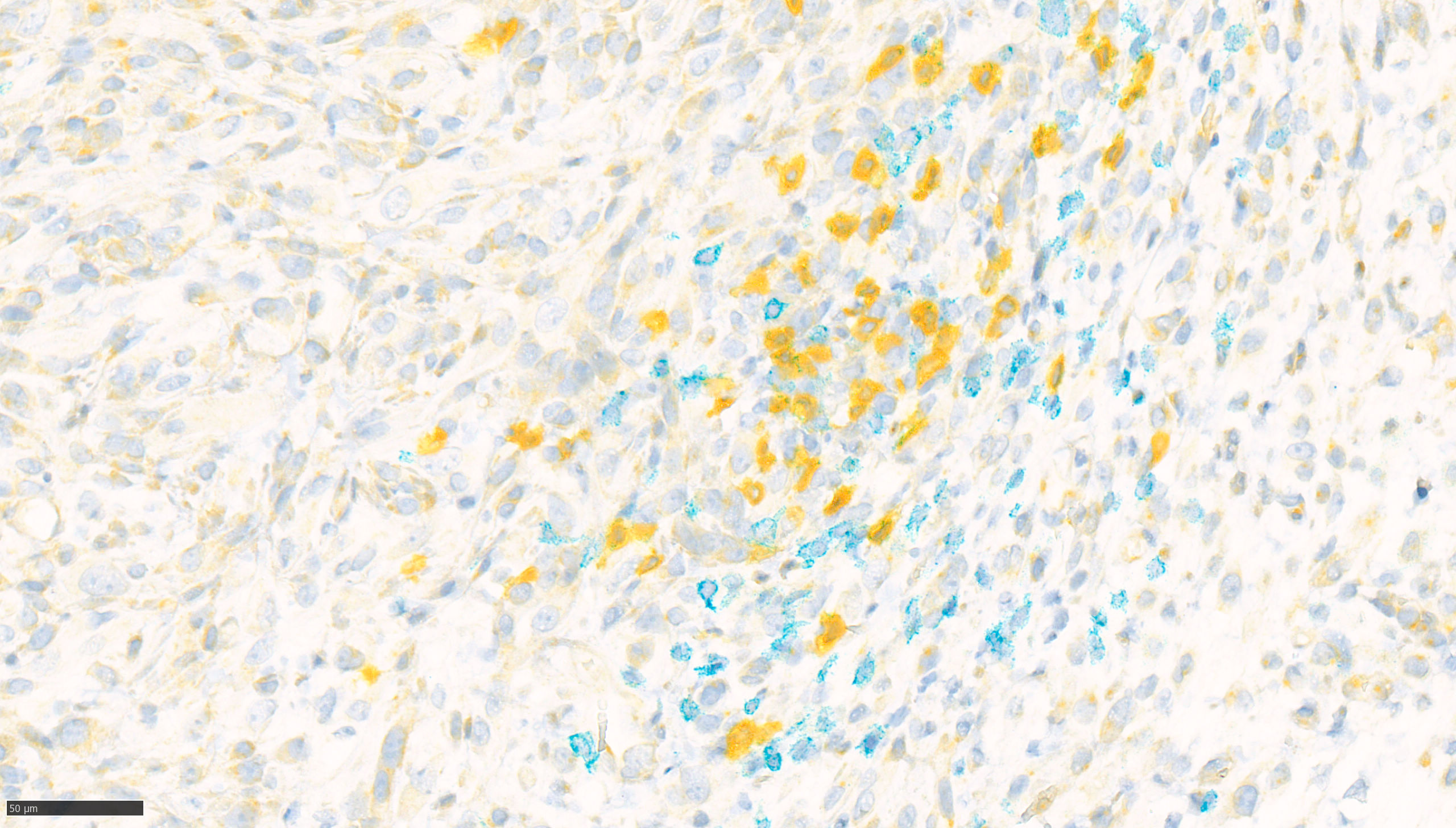

Supplement: Supplementary file 1 [file pharmaceutics-17-01273-s001.zip › IHC/CD4-CD8/CONV-5Gy/C5-2/C5-2-1.jpg]

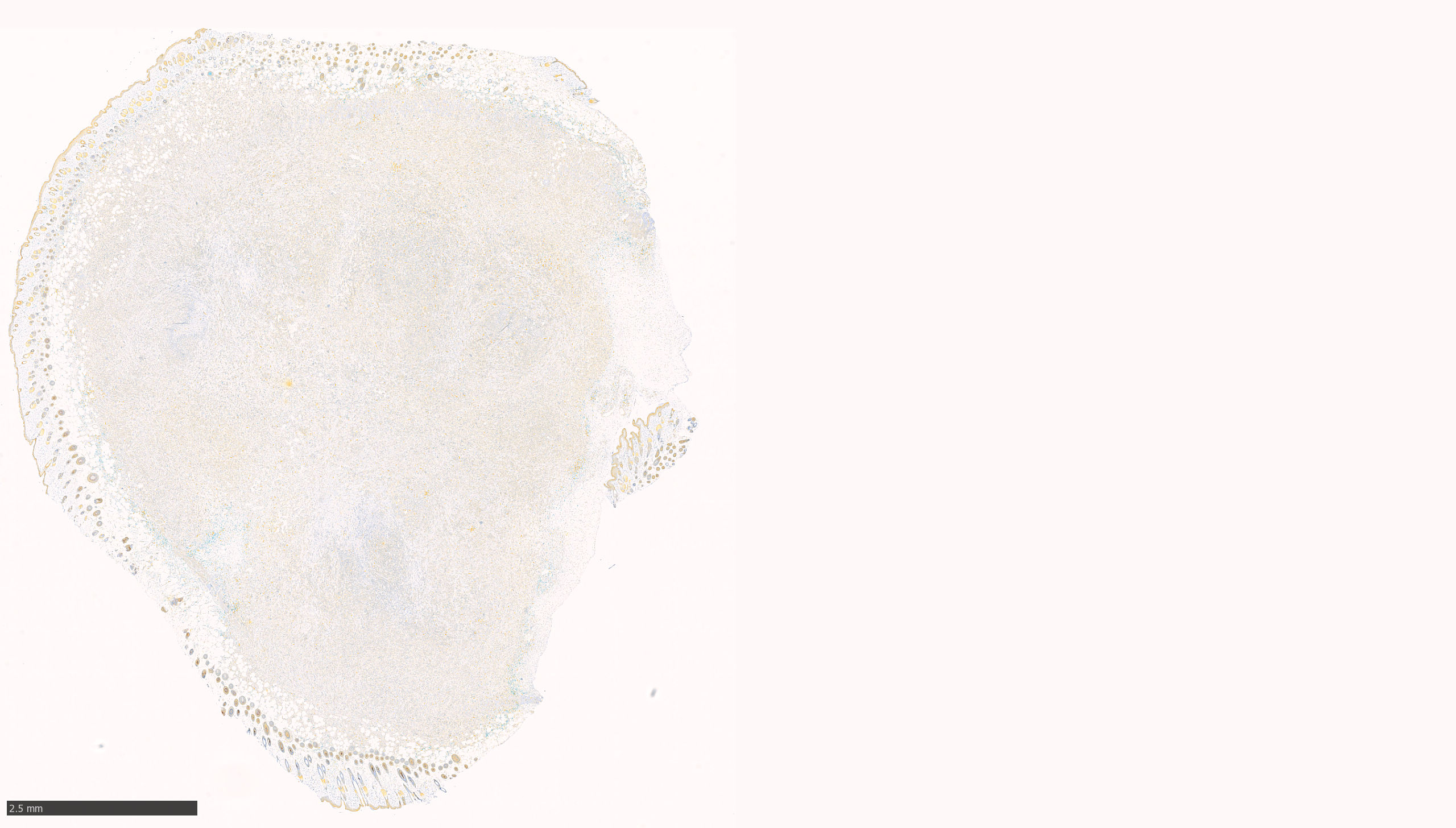

Supplement: Supplementary file 1 [file pharmaceutics-17-01273-s001.zip › IHC/CD4-CD8/CONV-5Gy/C5-2/C5-2.jpg]

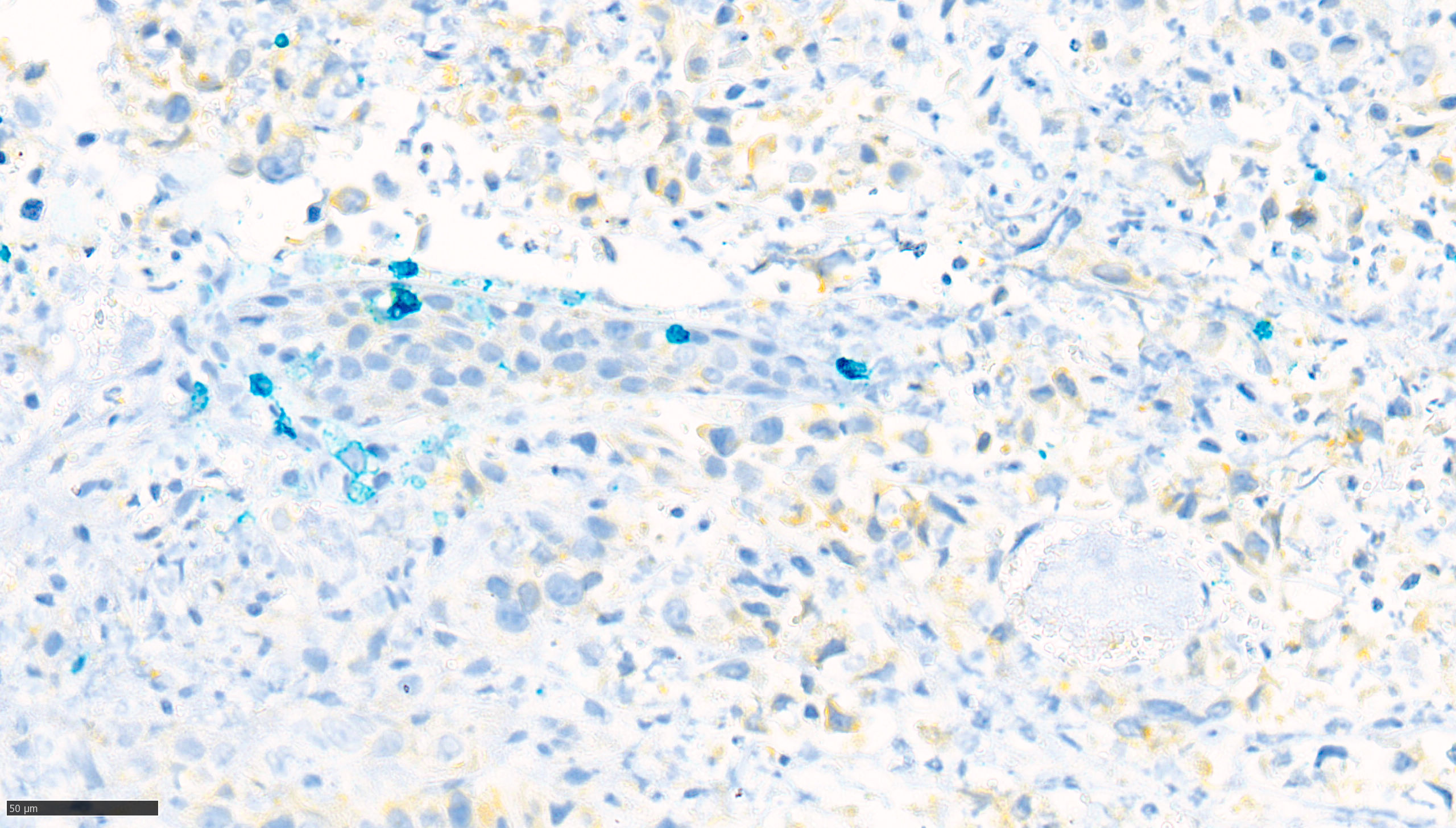

Supplement: Supplementary file 1 [file pharmaceutics-17-01273-s001.zip › IHC/CD4-CD8/CONV-5Gy/C5-3/C5-3-1.jpg]

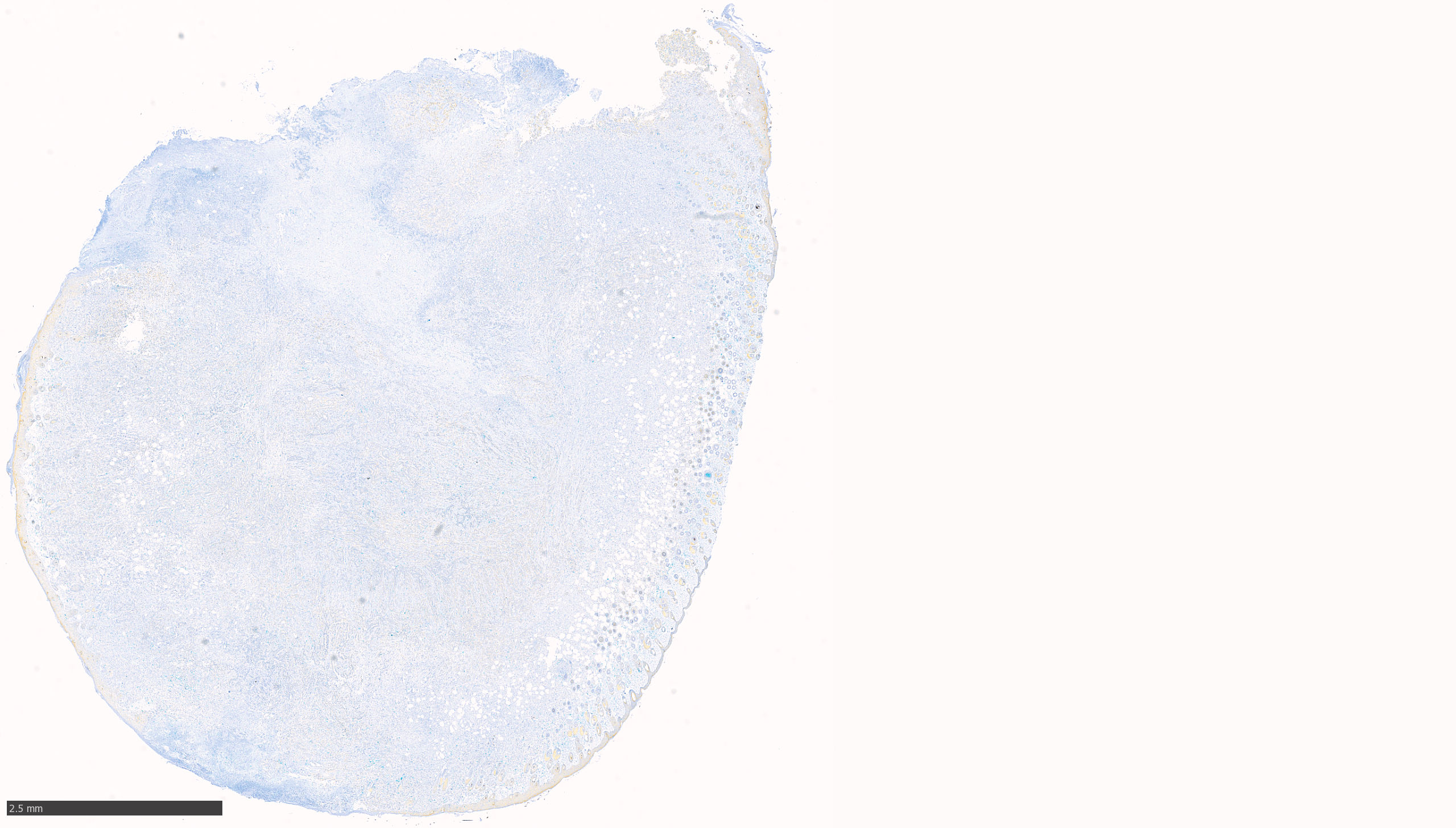

Supplement: Supplementary file 1 [file pharmaceutics-17-01273-s001.zip › IHC/CD4-CD8/CONV-5Gy/C5-3/C5-3.jpg]

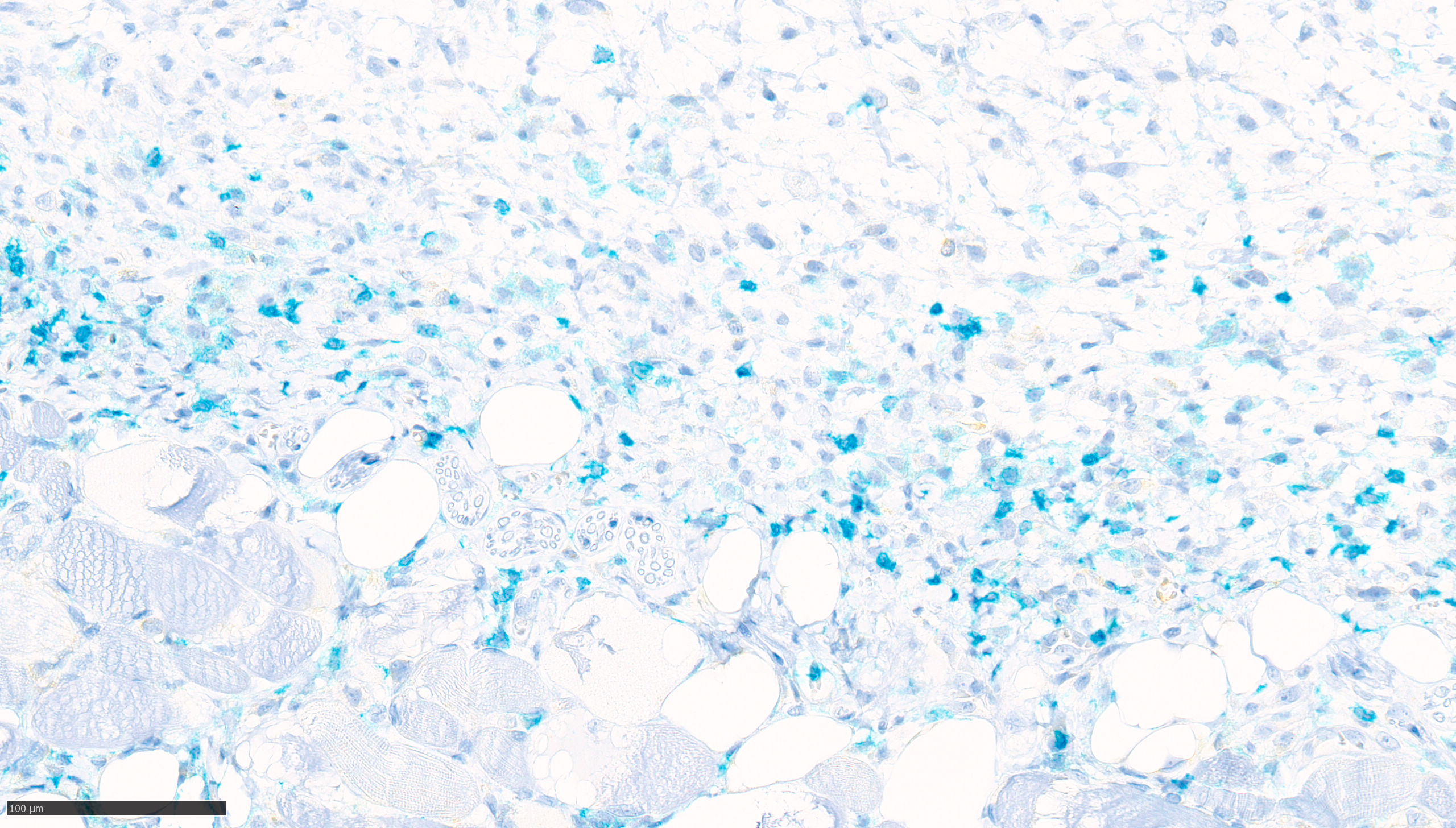

Supplement: Supplementary file 1 [file pharmaceutics-17-01273-s001.zip › IHC/CD4-CD8/CONV-8Gy/C8-1/C8-1-1.jpg]

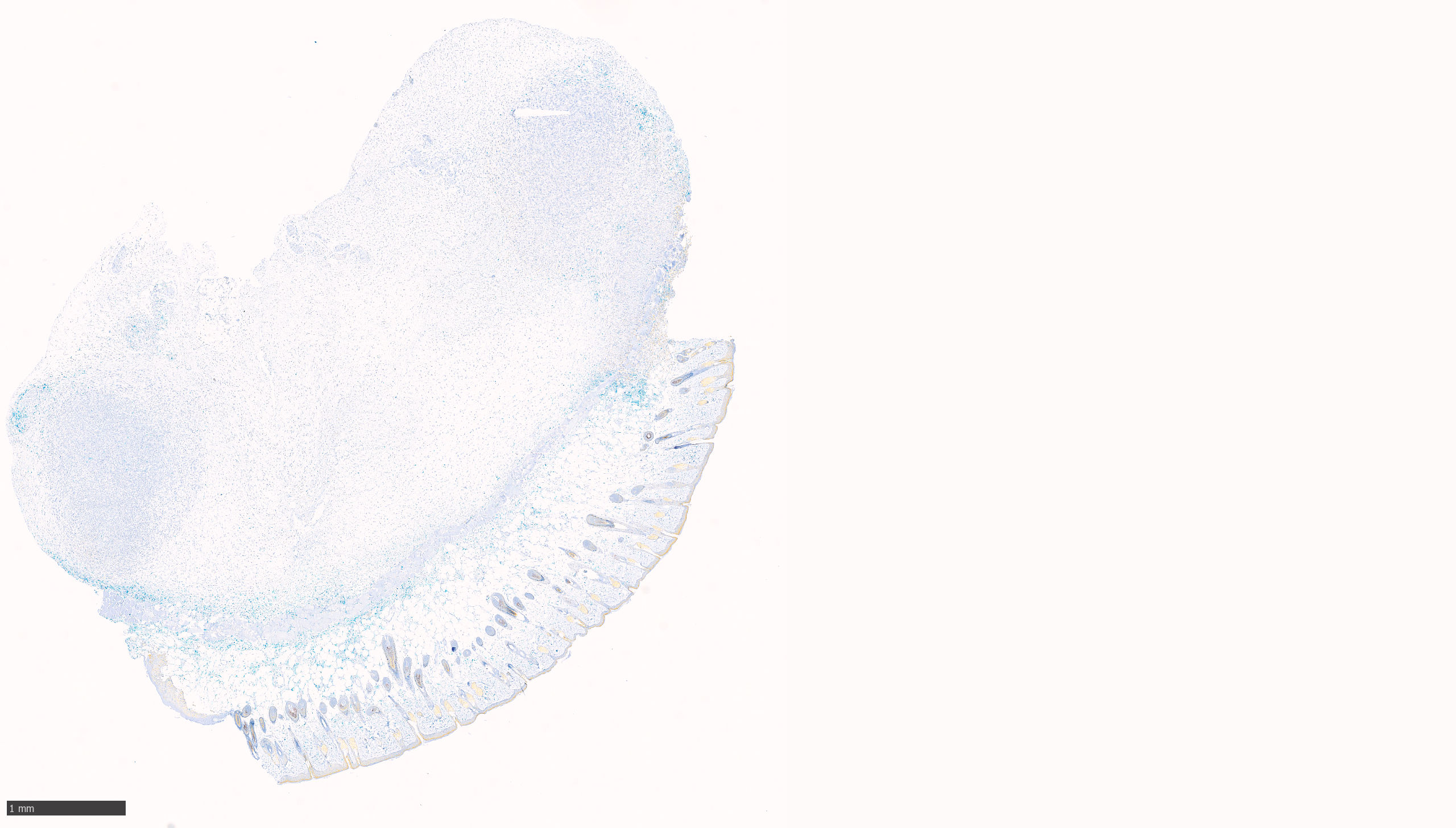

Supplement: Supplementary file 1 [file pharmaceutics-17-01273-s001.zip › IHC/CD4-CD8/CONV-8Gy/C8-1/C8-1.jpg]

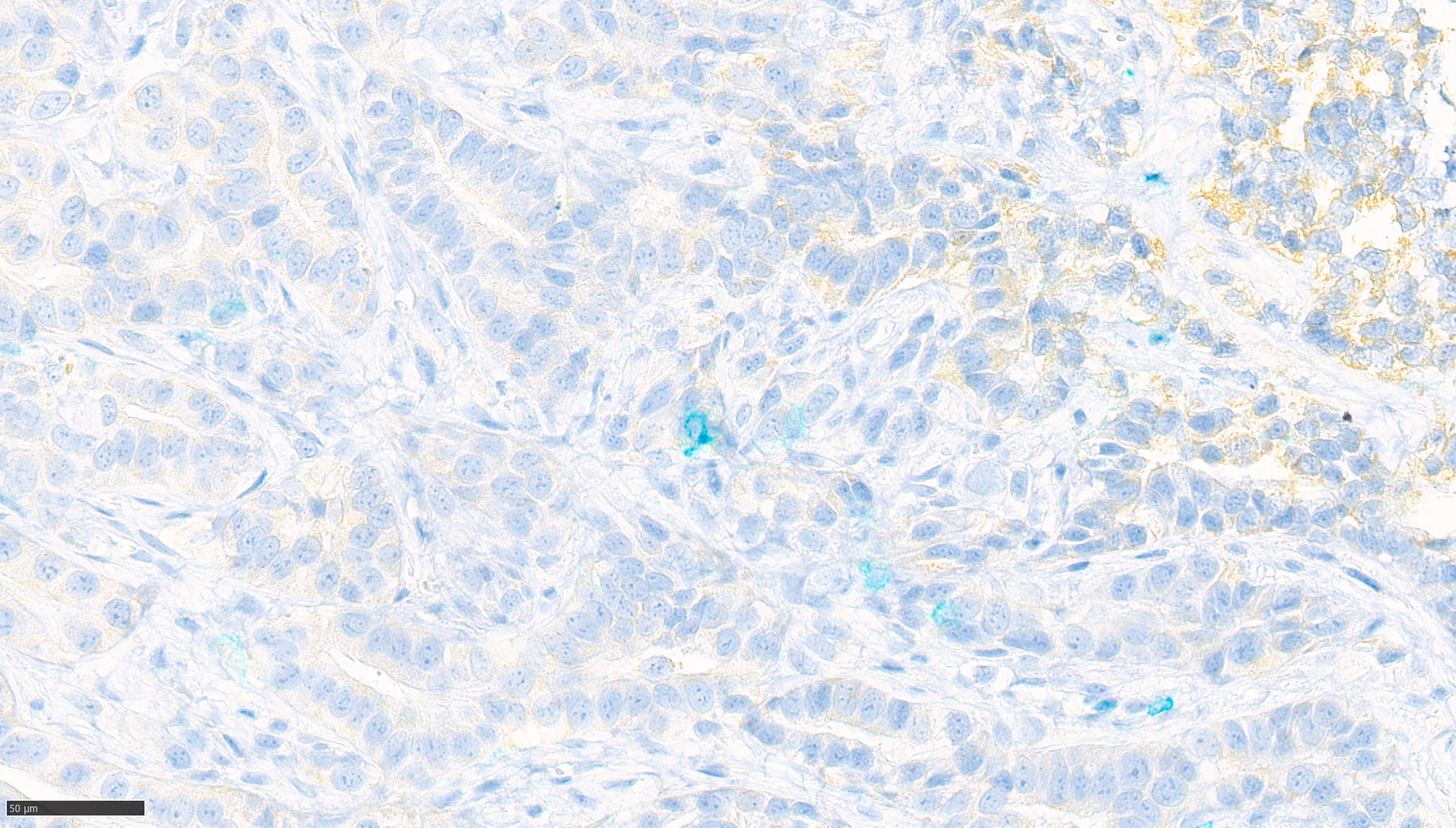

Supplement: Supplementary file 1 [file pharmaceutics-17-01273-s001.zip › IHC/CD4-CD8/CONV-8Gy/C8-2/C8-2-1.jpg]

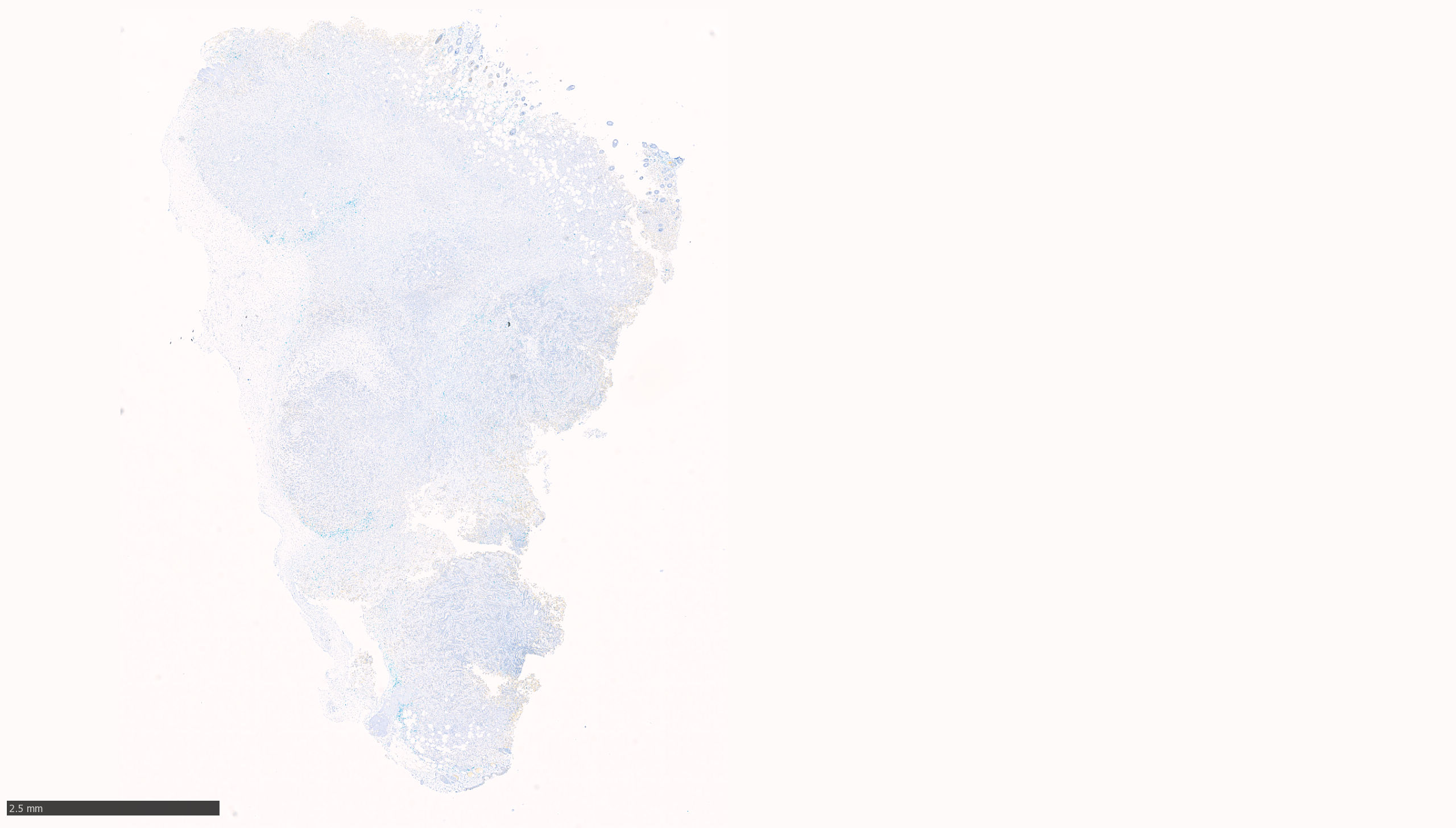

Supplement: Supplementary file 1 [file pharmaceutics-17-01273-s001.zip › IHC/CD4-CD8/CONV-8Gy/C8-2/C8-2.jpg]

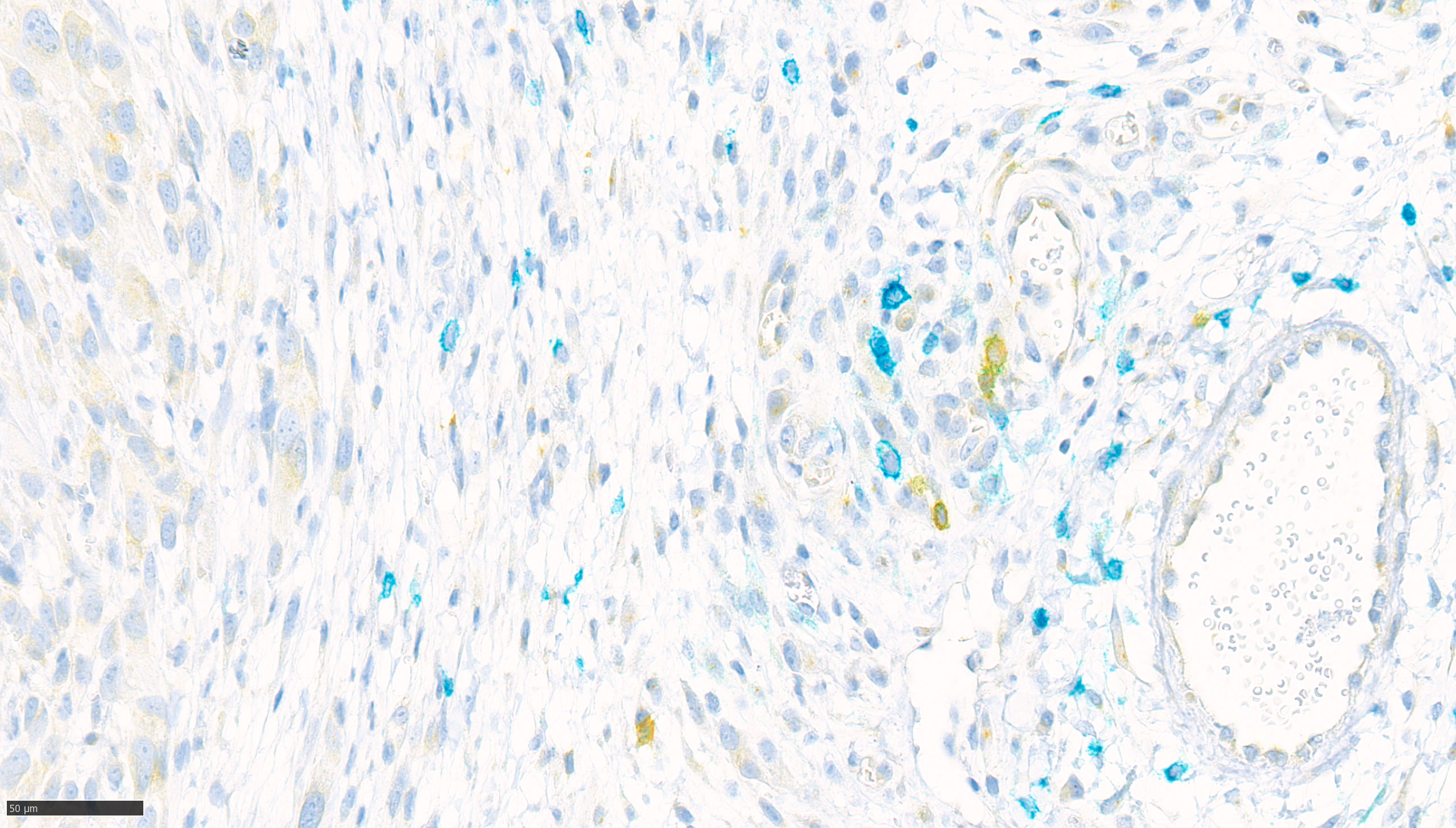

Supplement: Supplementary file 1 [file pharmaceutics-17-01273-s001.zip › IHC/CD4-CD8/CONV-8Gy/C8-3/C8-3-1.jpg]

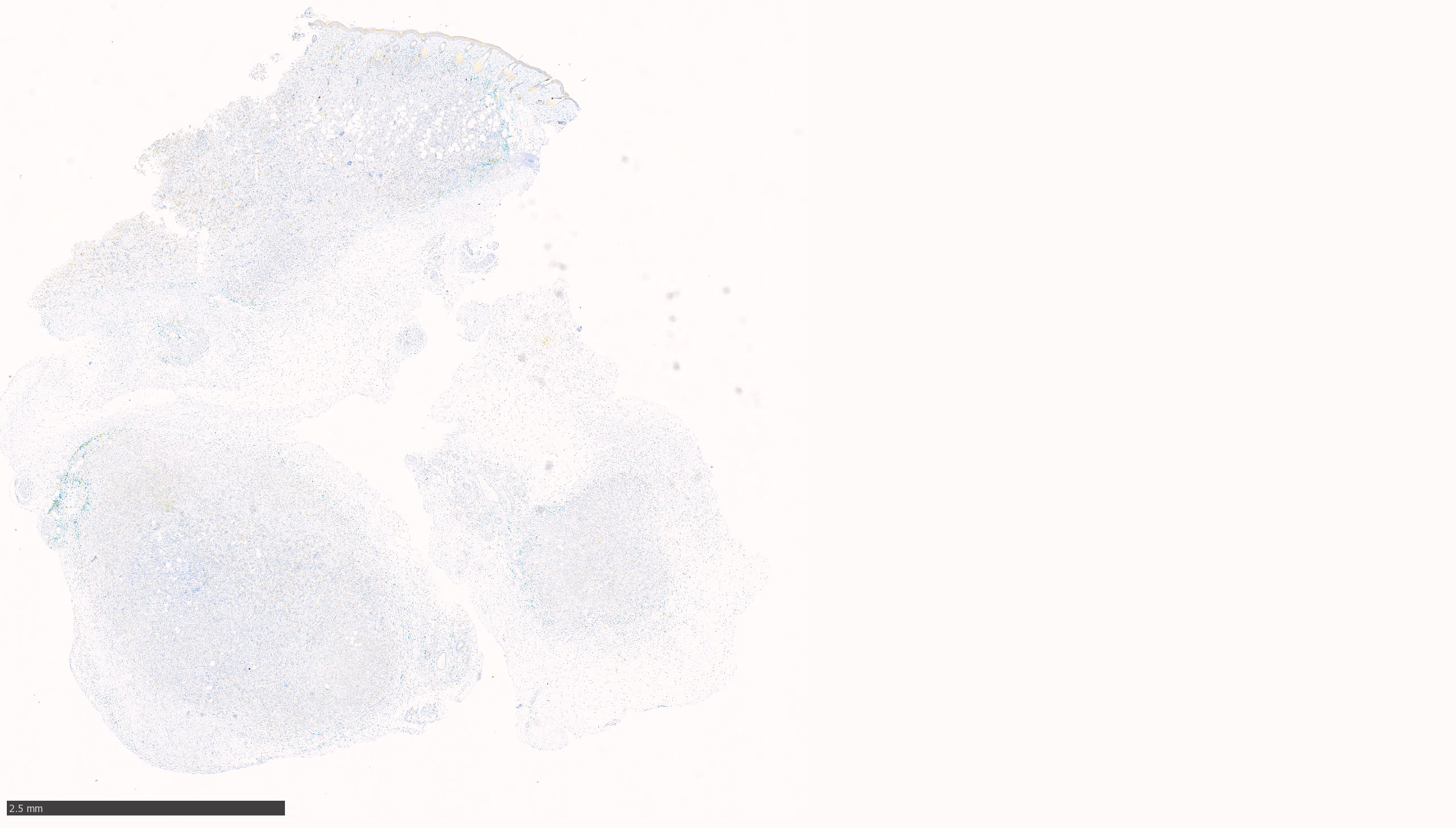

Supplement: Supplementary file 1 [file pharmaceutics-17-01273-s001.zip › IHC/CD4-CD8/CONV-8Gy/C8-3/C8-3.jpg]

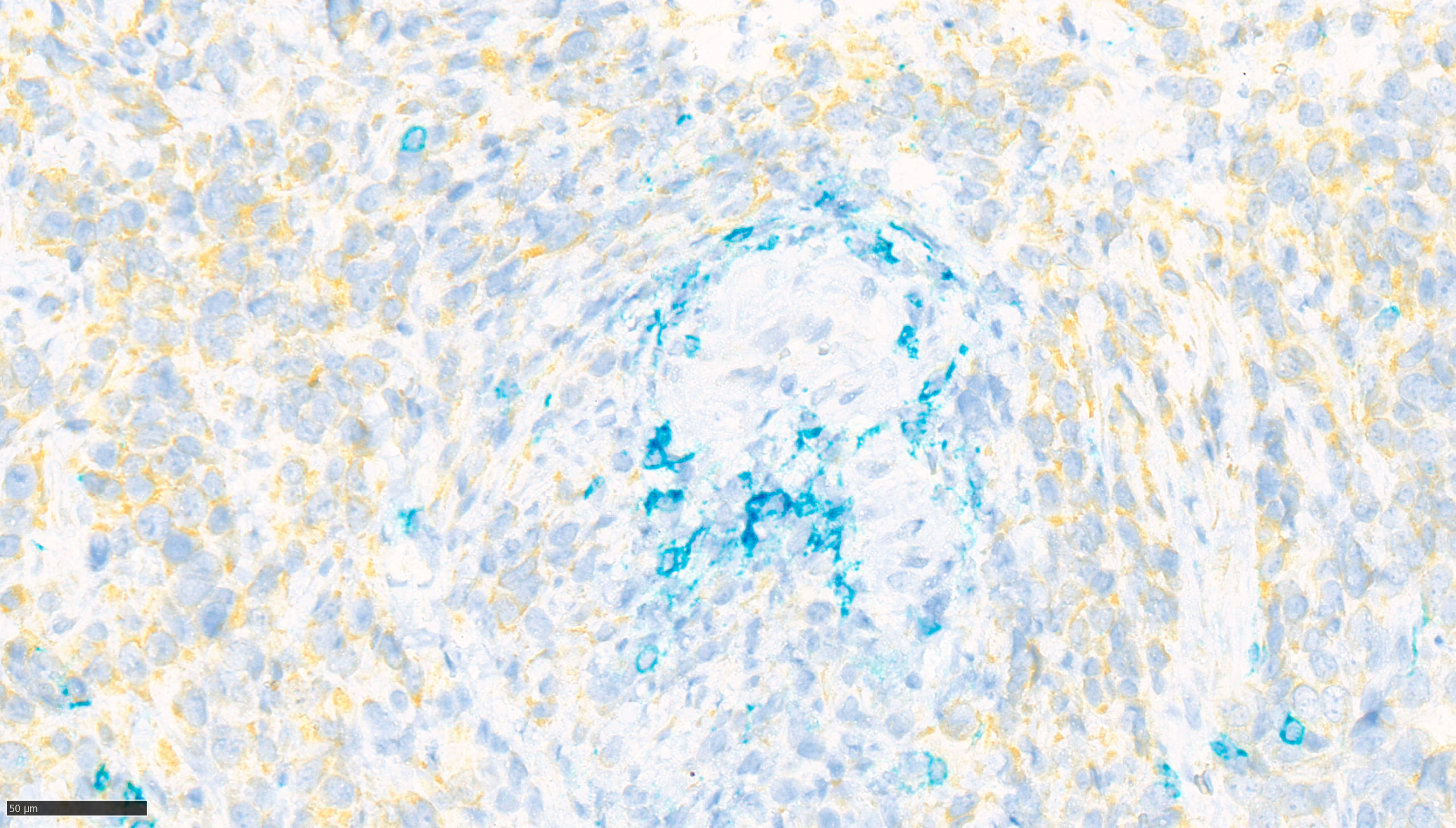

Supplement: Supplementary file 1 [file pharmaceutics-17-01273-s001.zip › IHC/CD4-CD8/FLASH-5Gy/F5-1/F5-1-1.jpg]

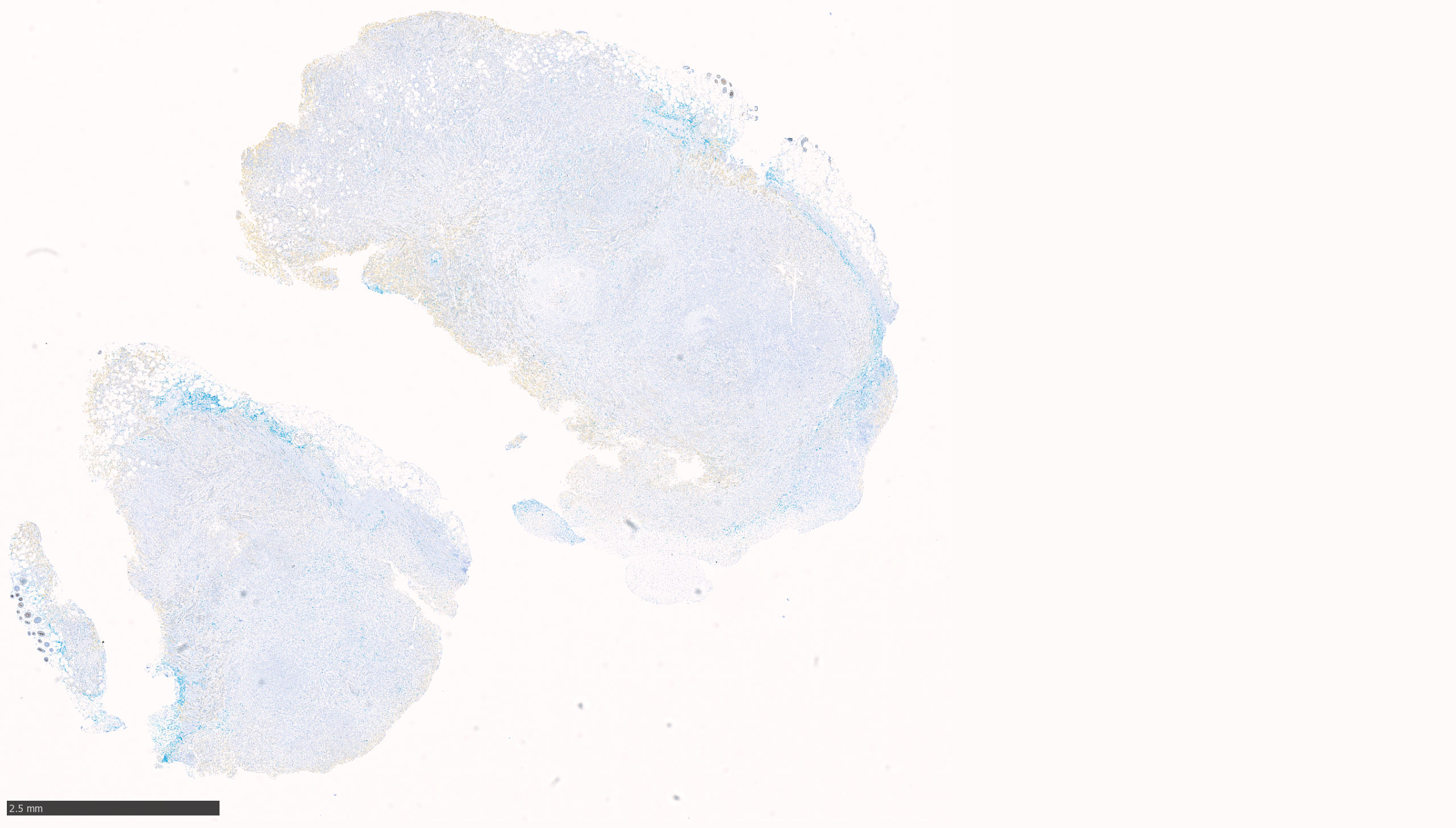

Supplement: Supplementary file 1 [file pharmaceutics-17-01273-s001.zip › IHC/CD4-CD8/FLASH-5Gy/F5-1/F5-1.jpg]

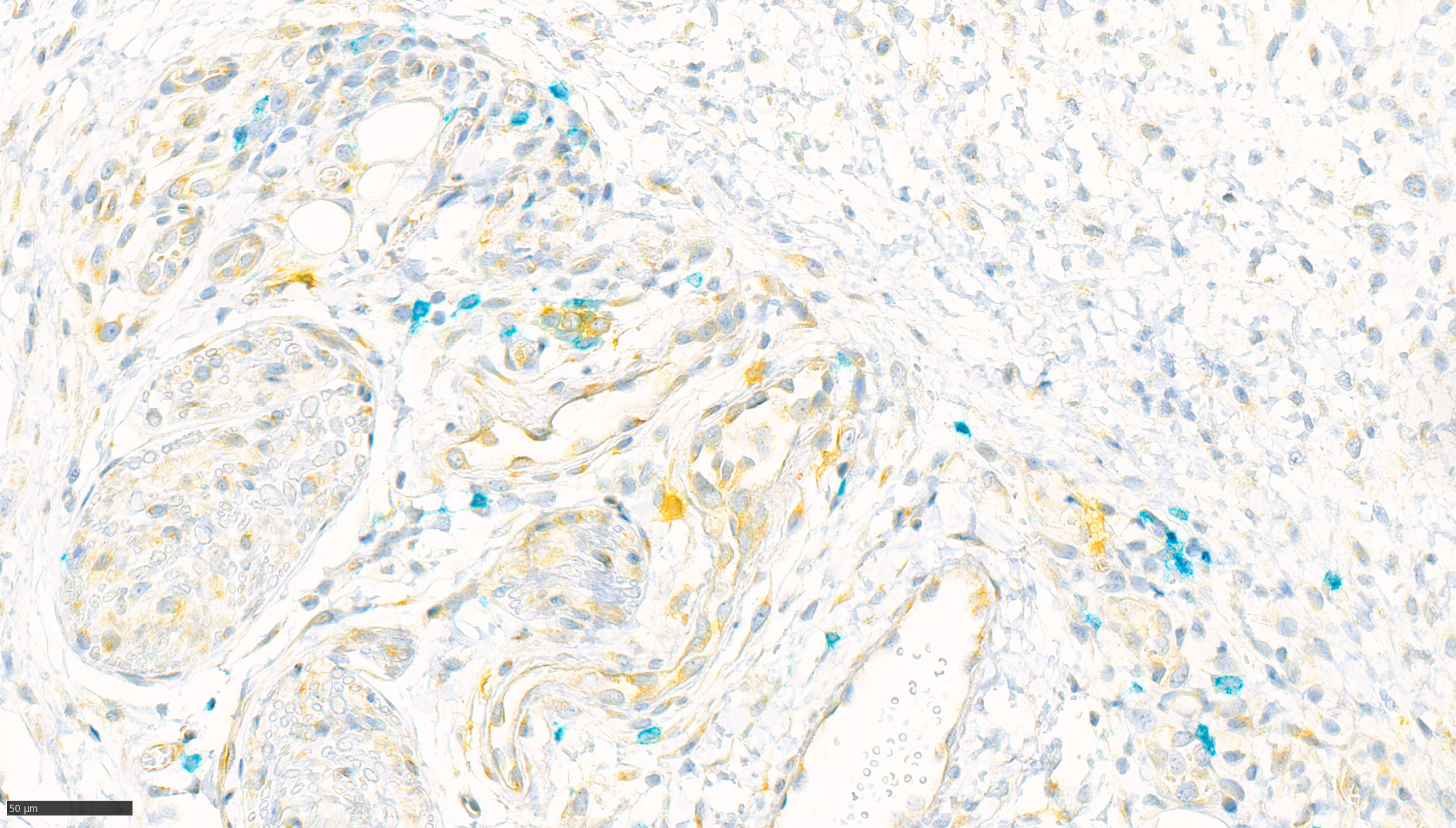

Supplement: Supplementary file 1 [file pharmaceutics-17-01273-s001.zip › IHC/CD4-CD8/FLASH-5Gy/F5-2/F5-2-1.jpg]

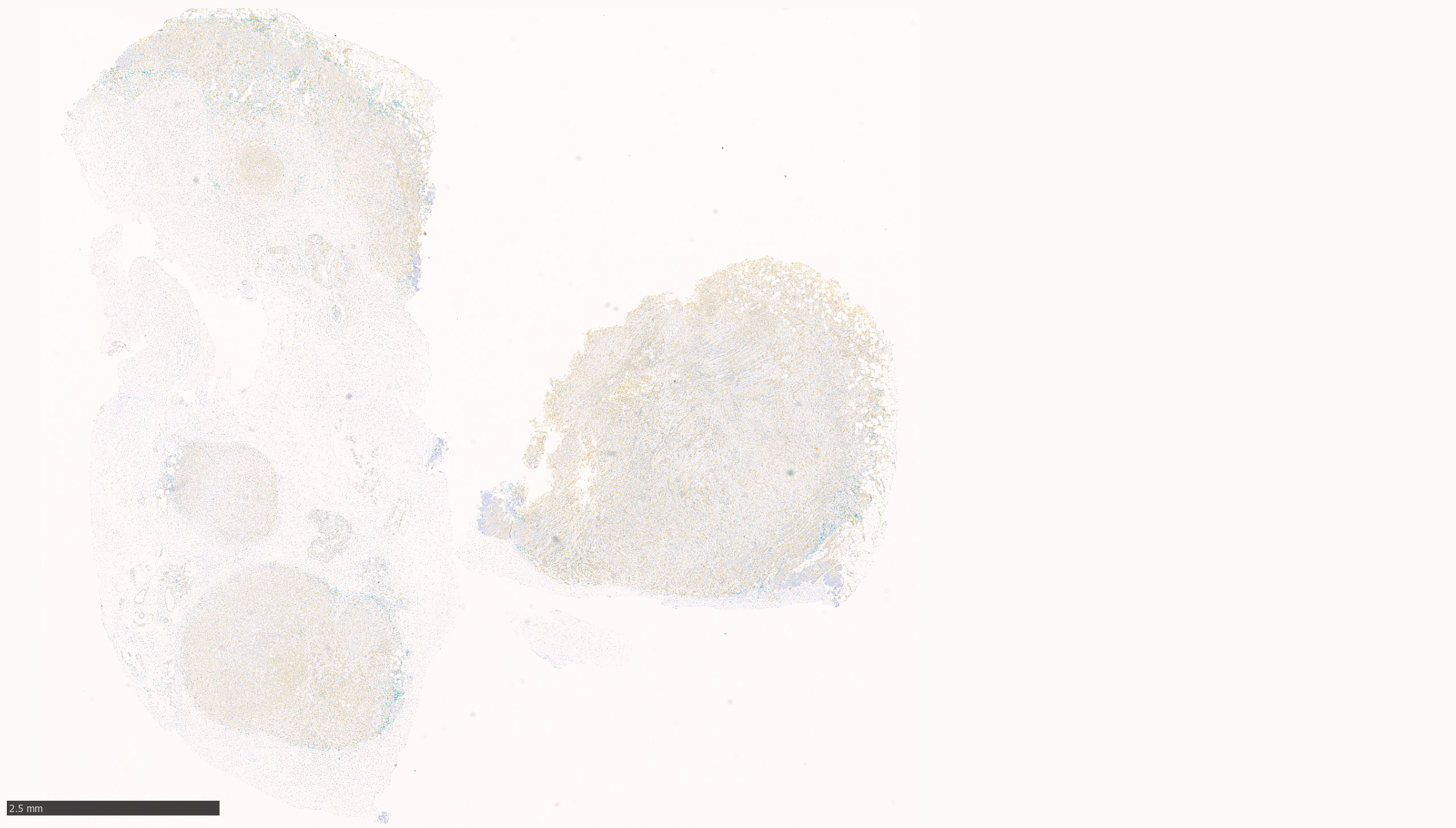

Supplement: Supplementary file 1 [file pharmaceutics-17-01273-s001.zip › IHC/CD4-CD8/FLASH-5Gy/F5-2/F5-2.jpg]

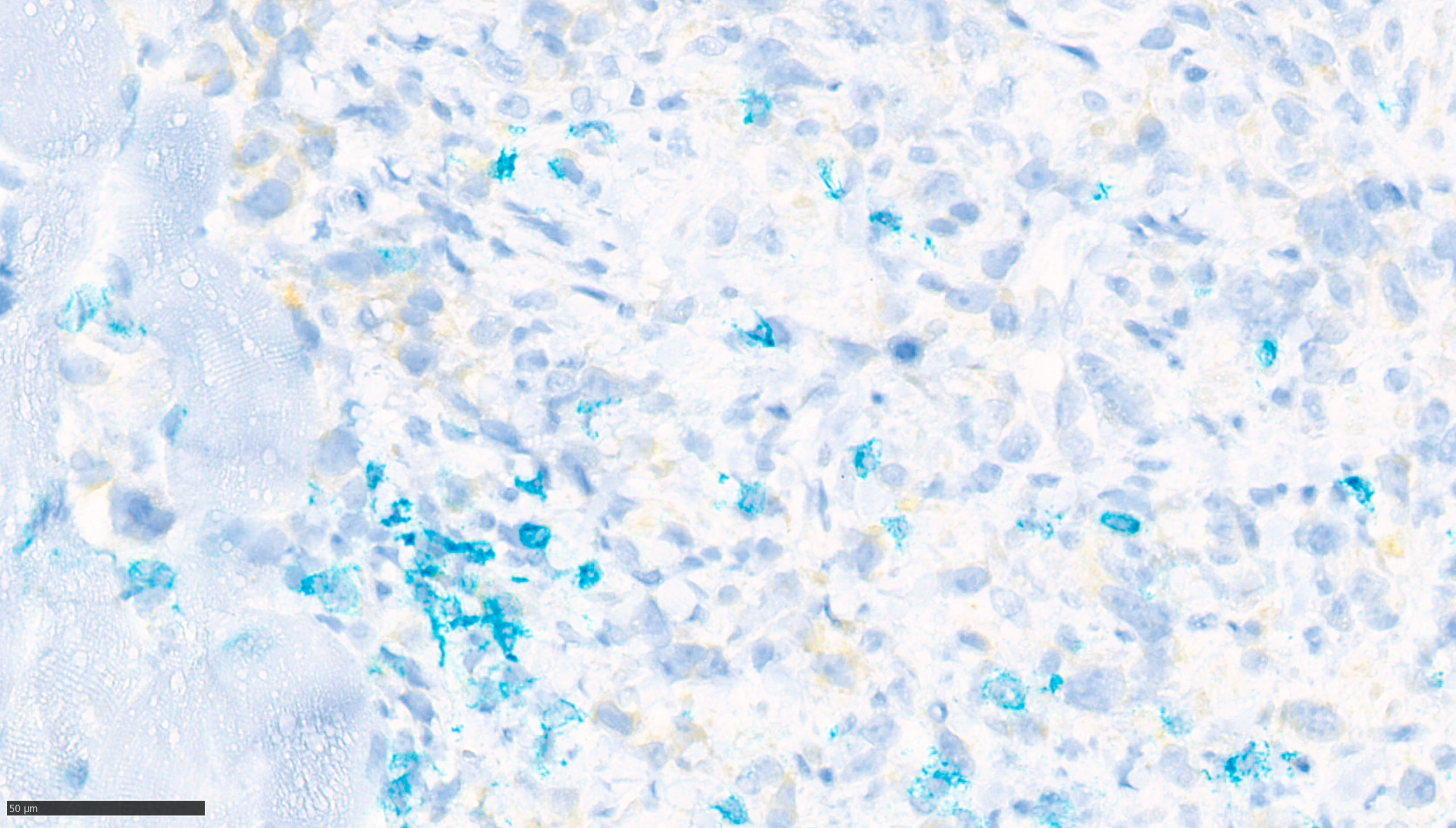

Supplement: Supplementary file 1 [file pharmaceutics-17-01273-s001.zip › IHC/CD4-CD8/FLASH-5Gy/F5-3/F5-3-1.jpg]

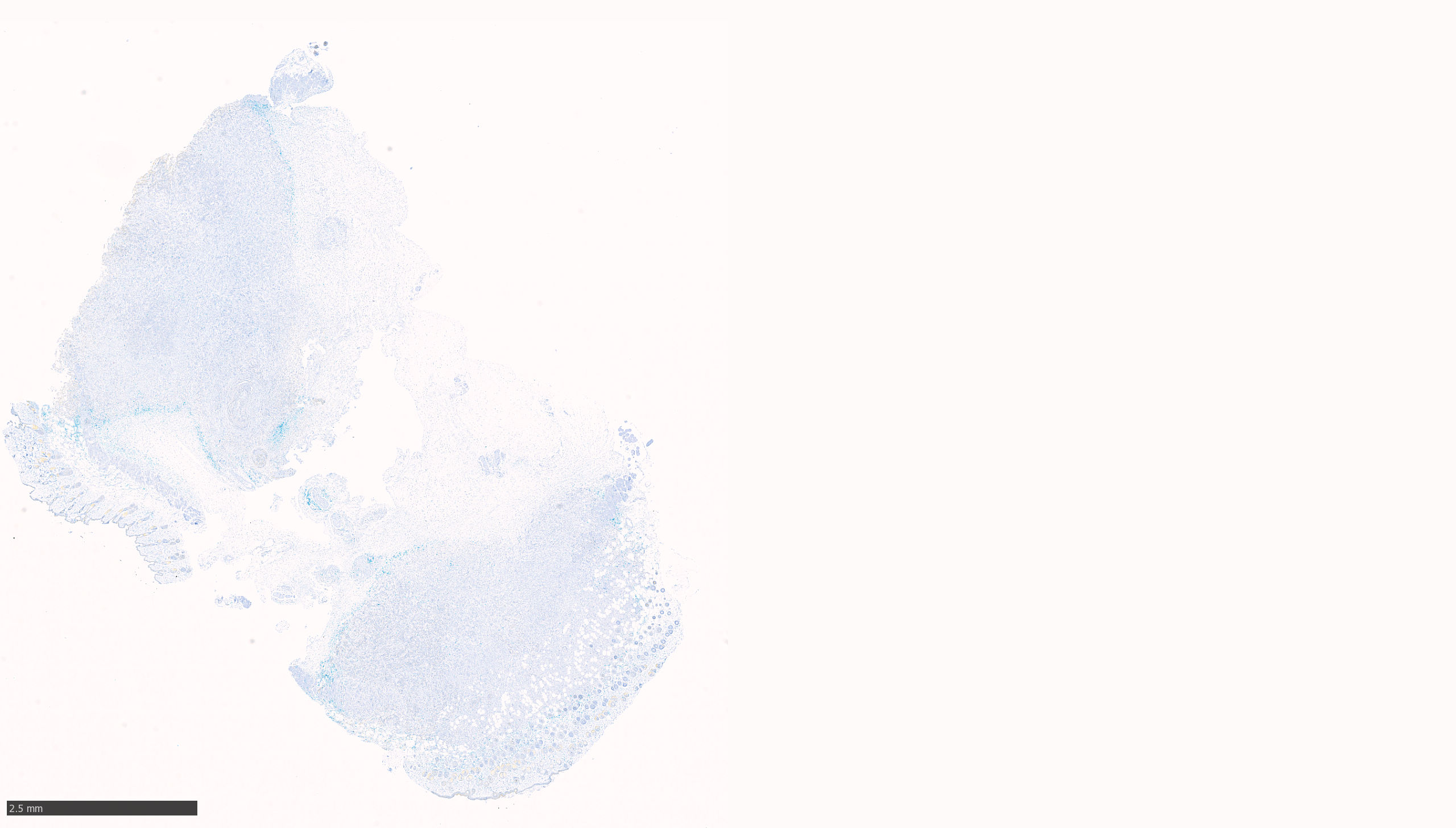

Supplement: Supplementary file 1 [file pharmaceutics-17-01273-s001.zip › IHC/CD4-CD8/FLASH-5Gy/F5-3/F5-3.jpg]

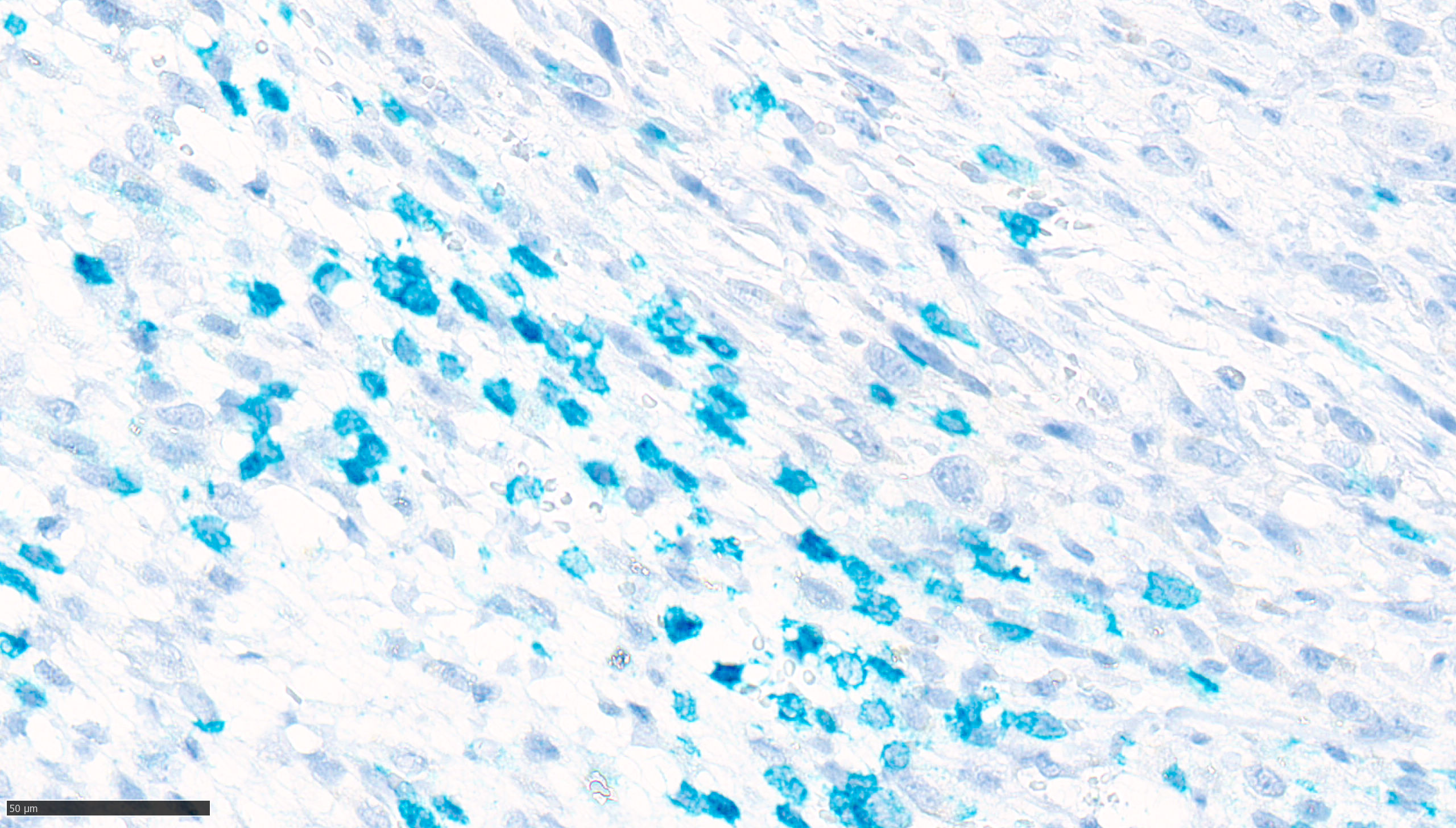

Supplement: Supplementary file 1 [file pharmaceutics-17-01273-s001.zip › IHC/CD4-CD8/FLASH-8Gy/F8-1/F8-1-1.jpg]

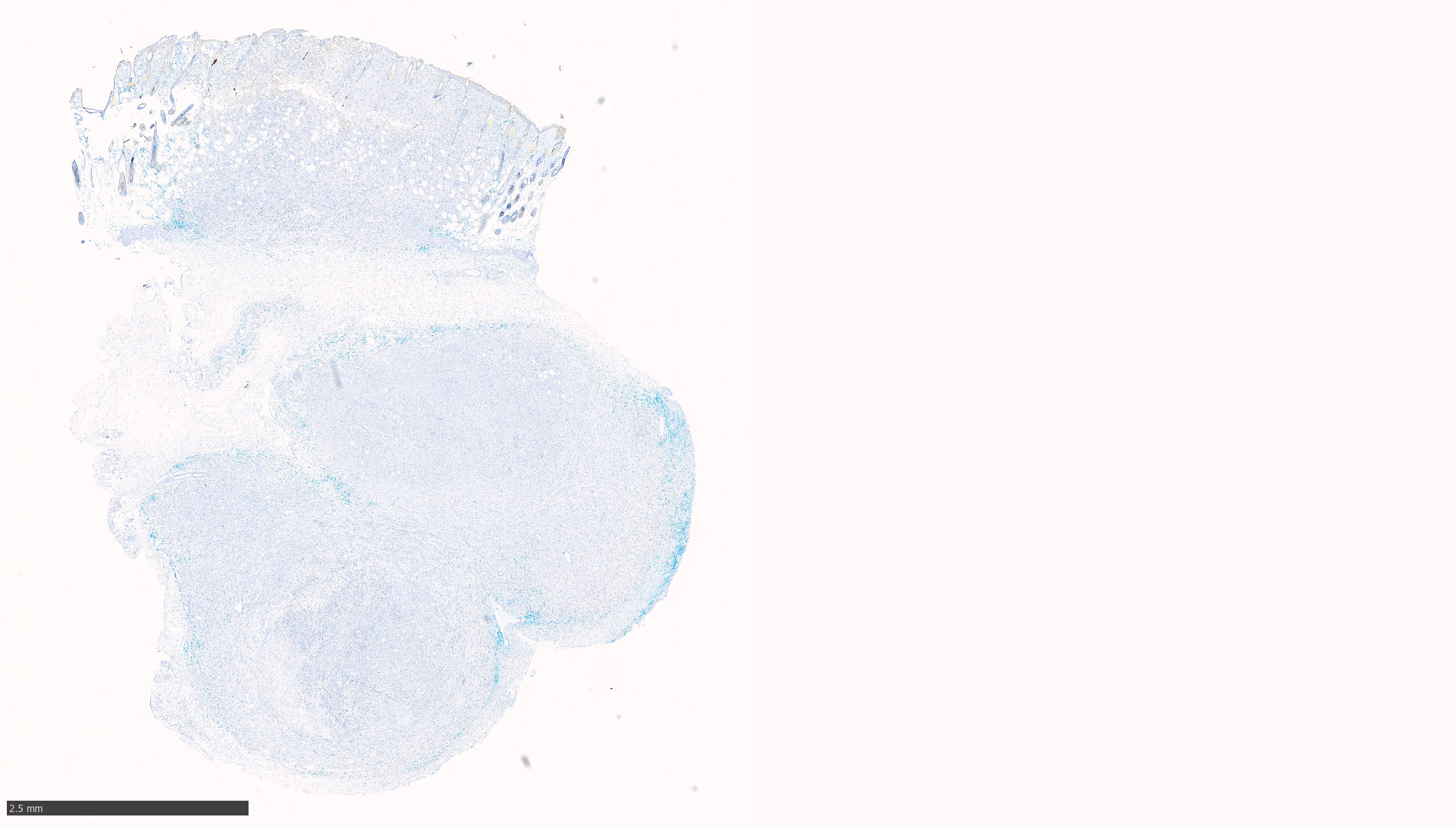

Supplement: Supplementary file 1 [file pharmaceutics-17-01273-s001.zip › IHC/CD4-CD8/FLASH-8Gy/F8-1/F8-1.jpg]

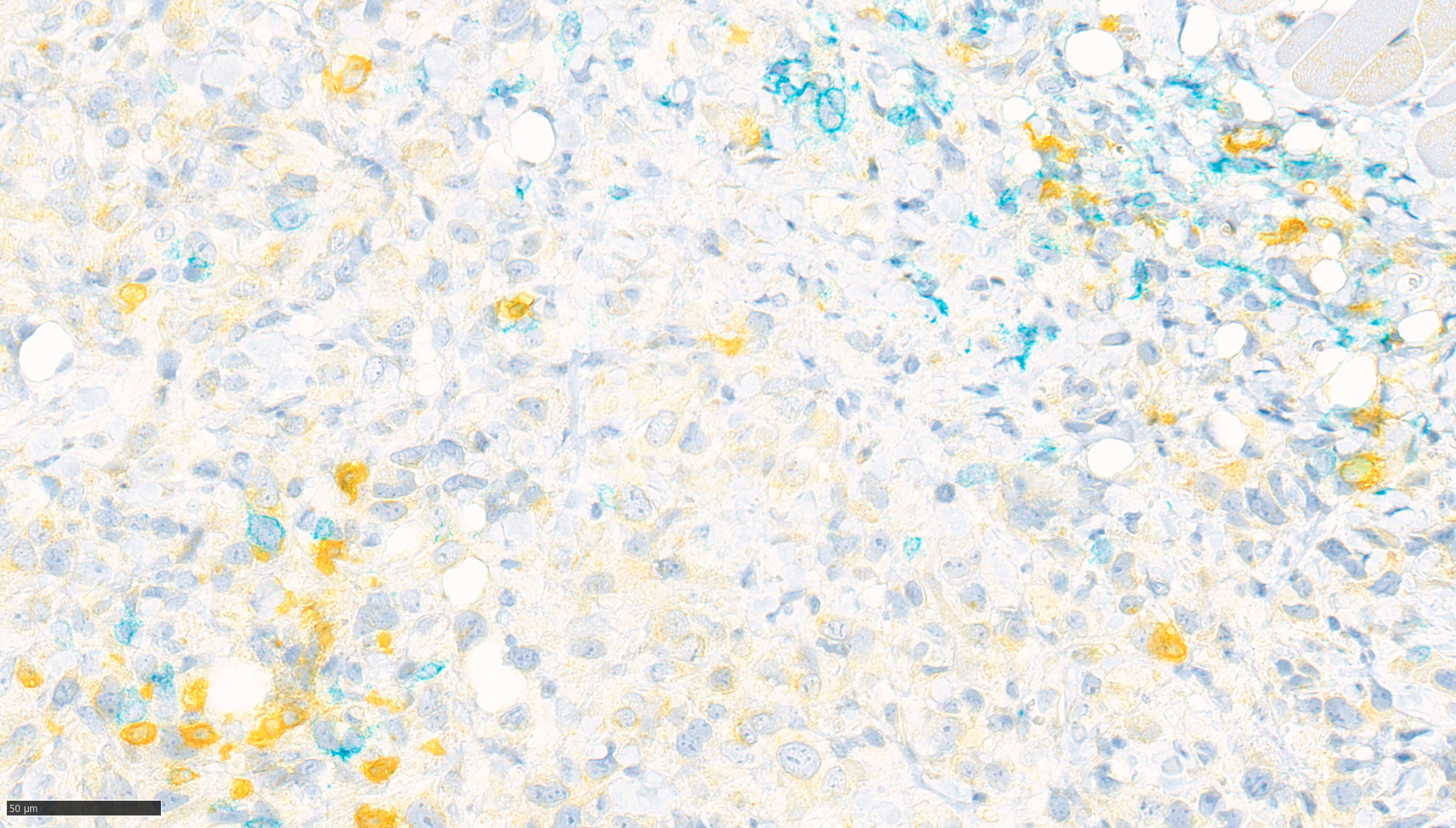

Supplement: Supplementary file 1 [file pharmaceutics-17-01273-s001.zip › IHC/CD4-CD8/FLASH-8Gy/F8-2/F8-2-1.jpg]

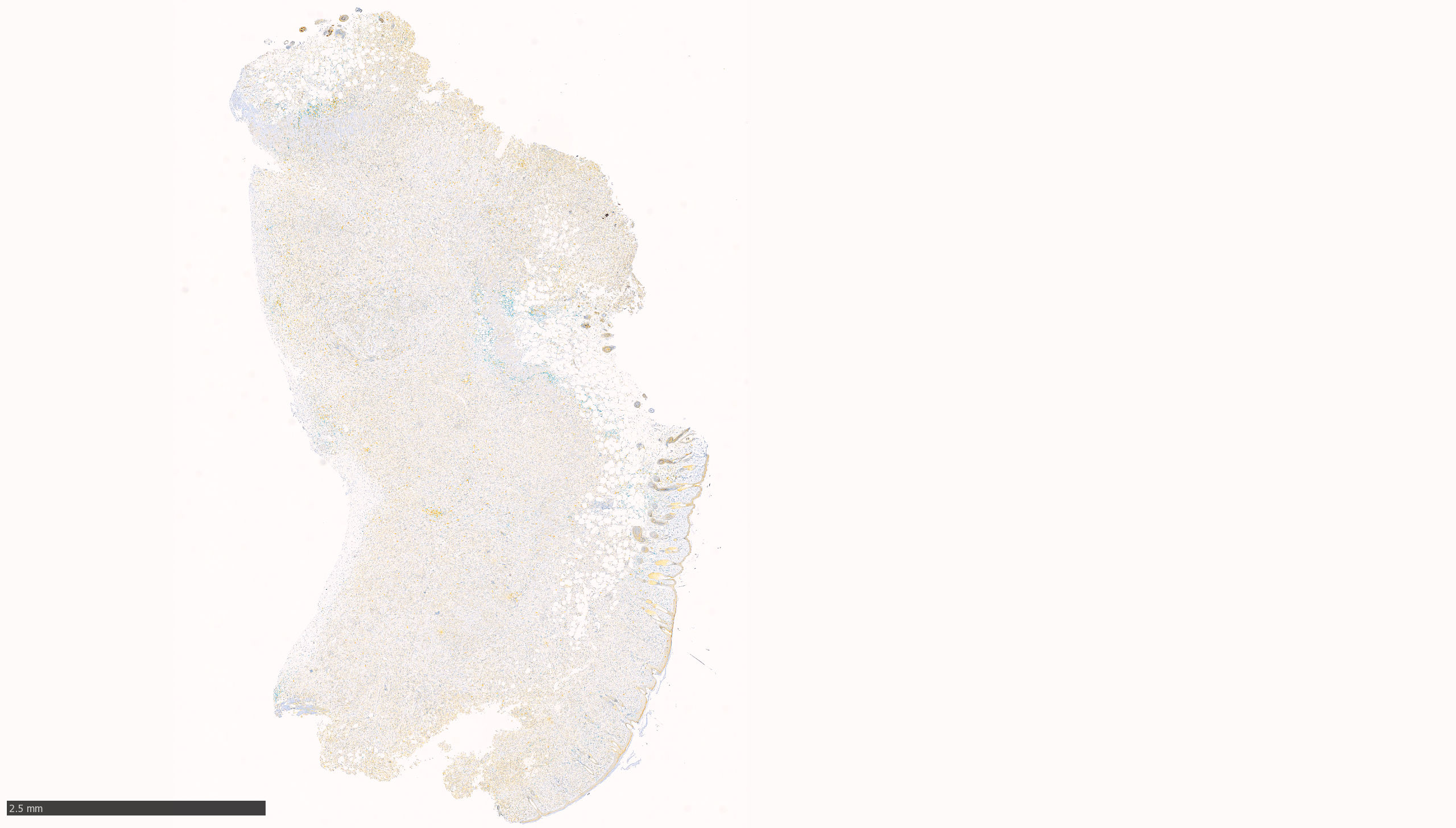

Supplement: Supplementary file 1 [file pharmaceutics-17-01273-s001.zip › IHC/CD4-CD8/FLASH-8Gy/F8-2/F8-2.jpg]

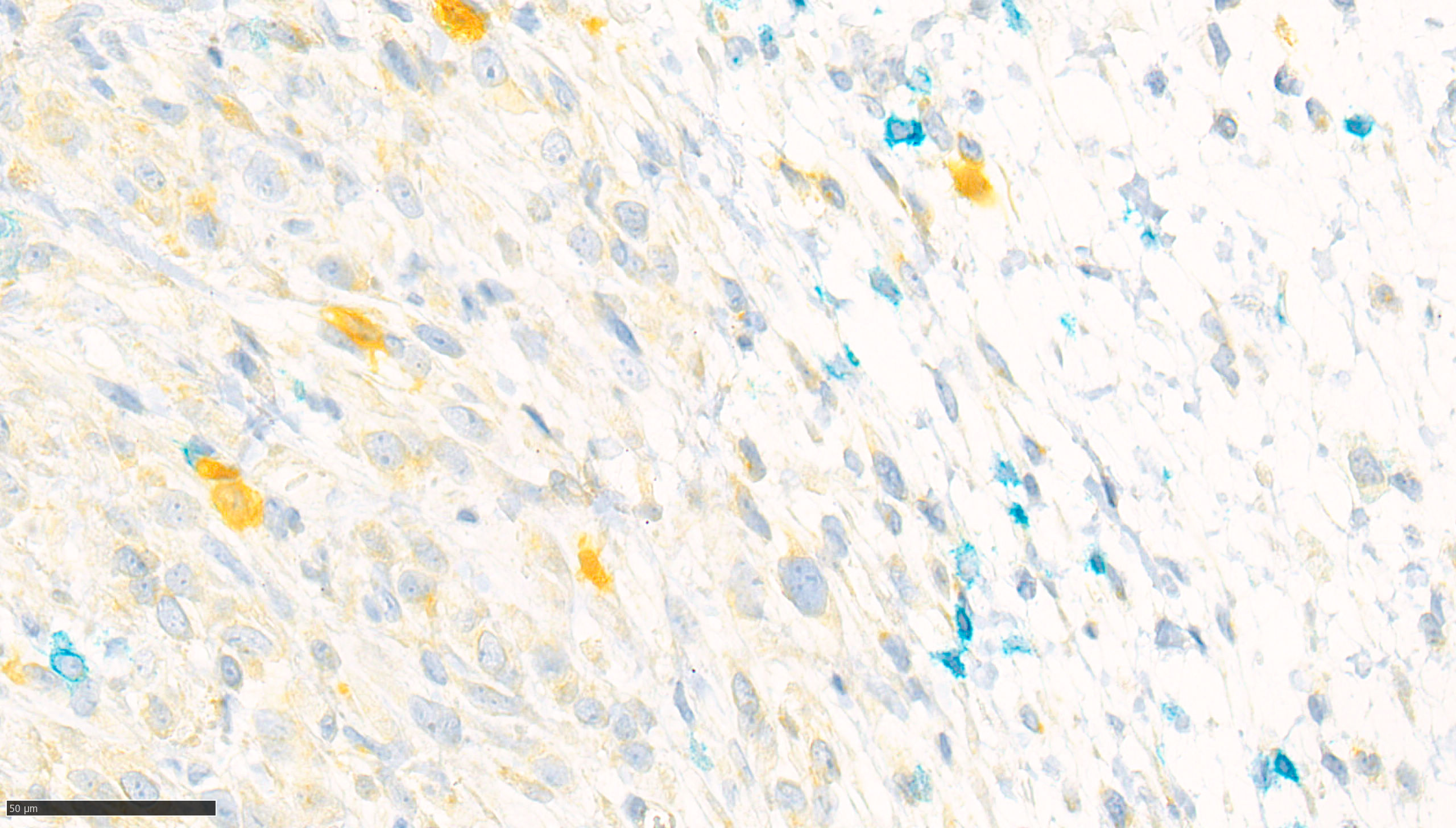

Supplement: Supplementary file 1 [file pharmaceutics-17-01273-s001.zip › IHC/CD4-CD8/FLASH-8Gy/F8-3/F8-3-1.jpg]

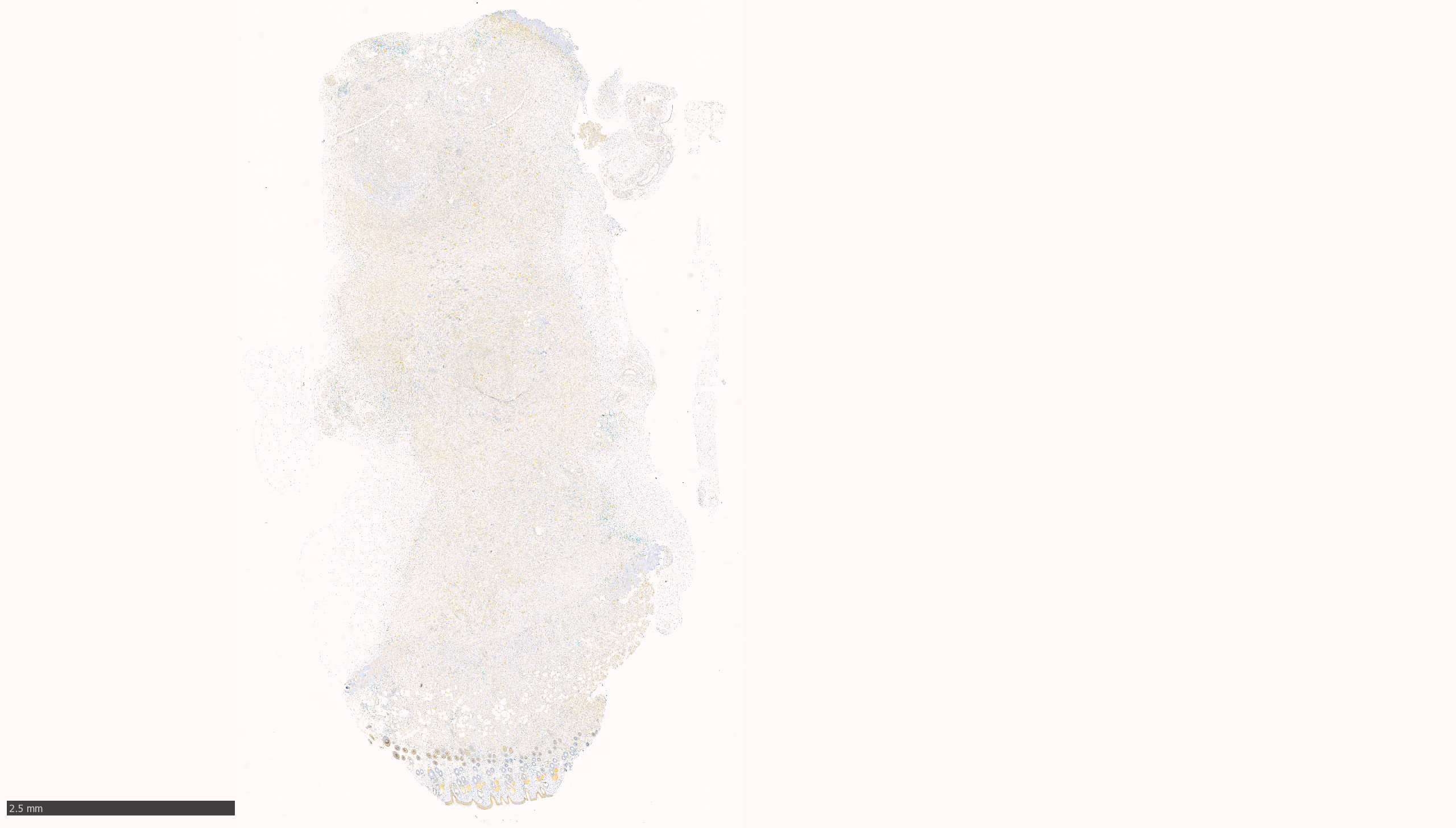

Supplement: Supplementary file 1 [file pharmaceutics-17-01273-s001.zip › IHC/CD4-CD8/FLASH-8Gy/F8-3/F8-3.jpg]

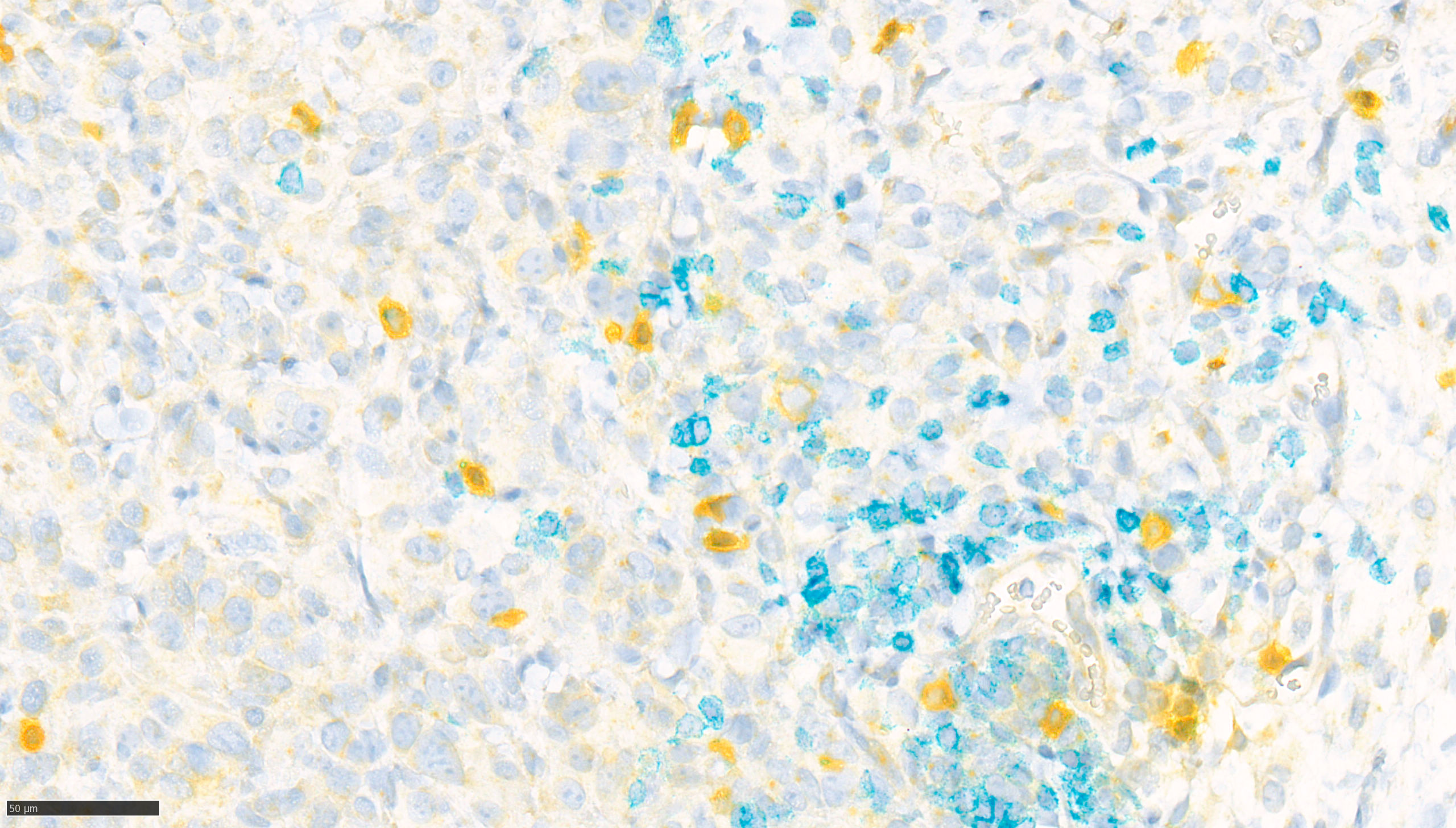

Supplement: Supplementary file 1 [file pharmaceutics-17-01273-s001.zip › IHC/CD4-CD8/LIFE BIOMATERIAL/L1/L1-1.jpg]

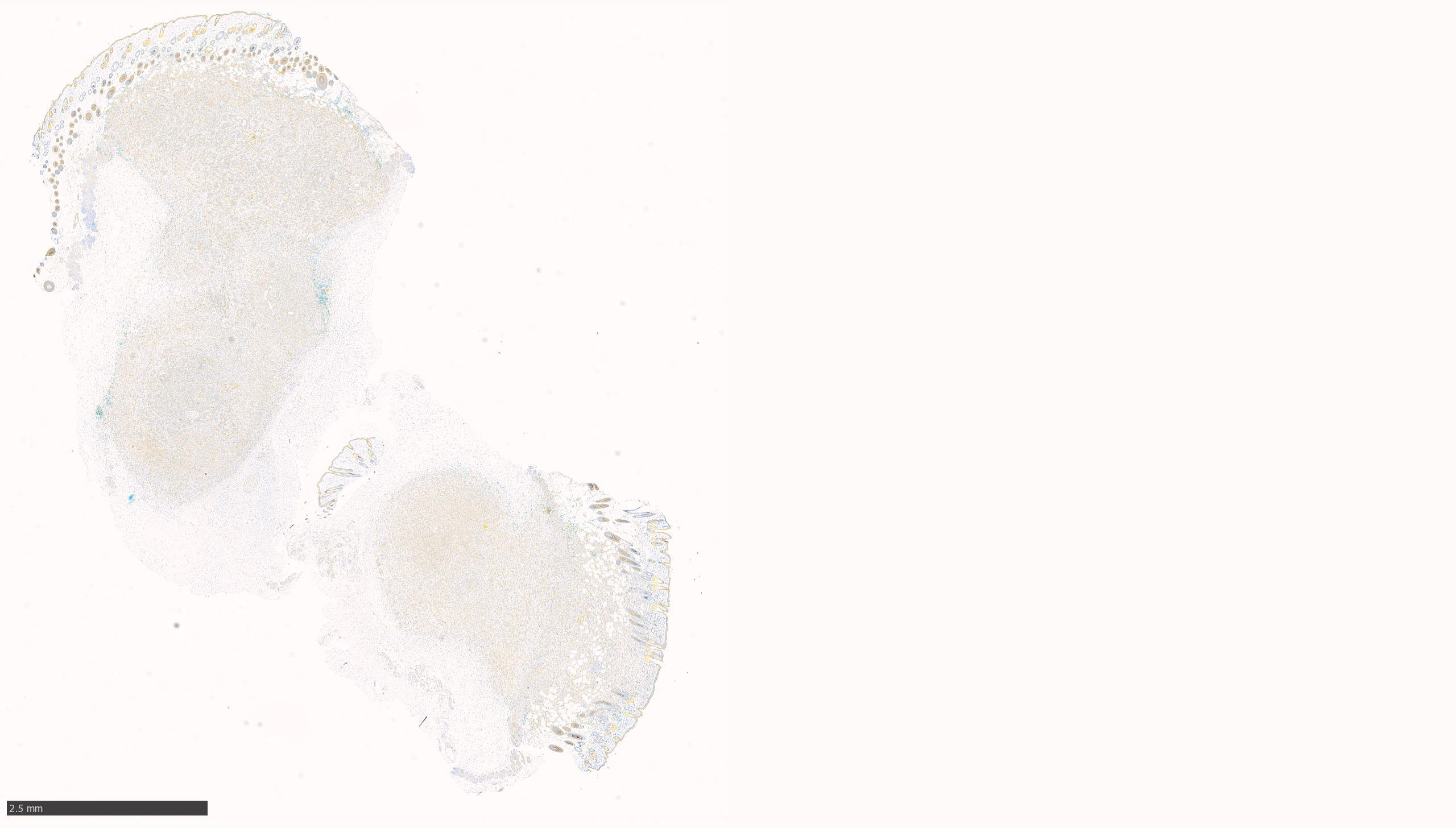

Supplement: Supplementary file 1 [file pharmaceutics-17-01273-s001.zip › IHC/CD4-CD8/LIFE BIOMATERIAL/L1/L1.jpg]

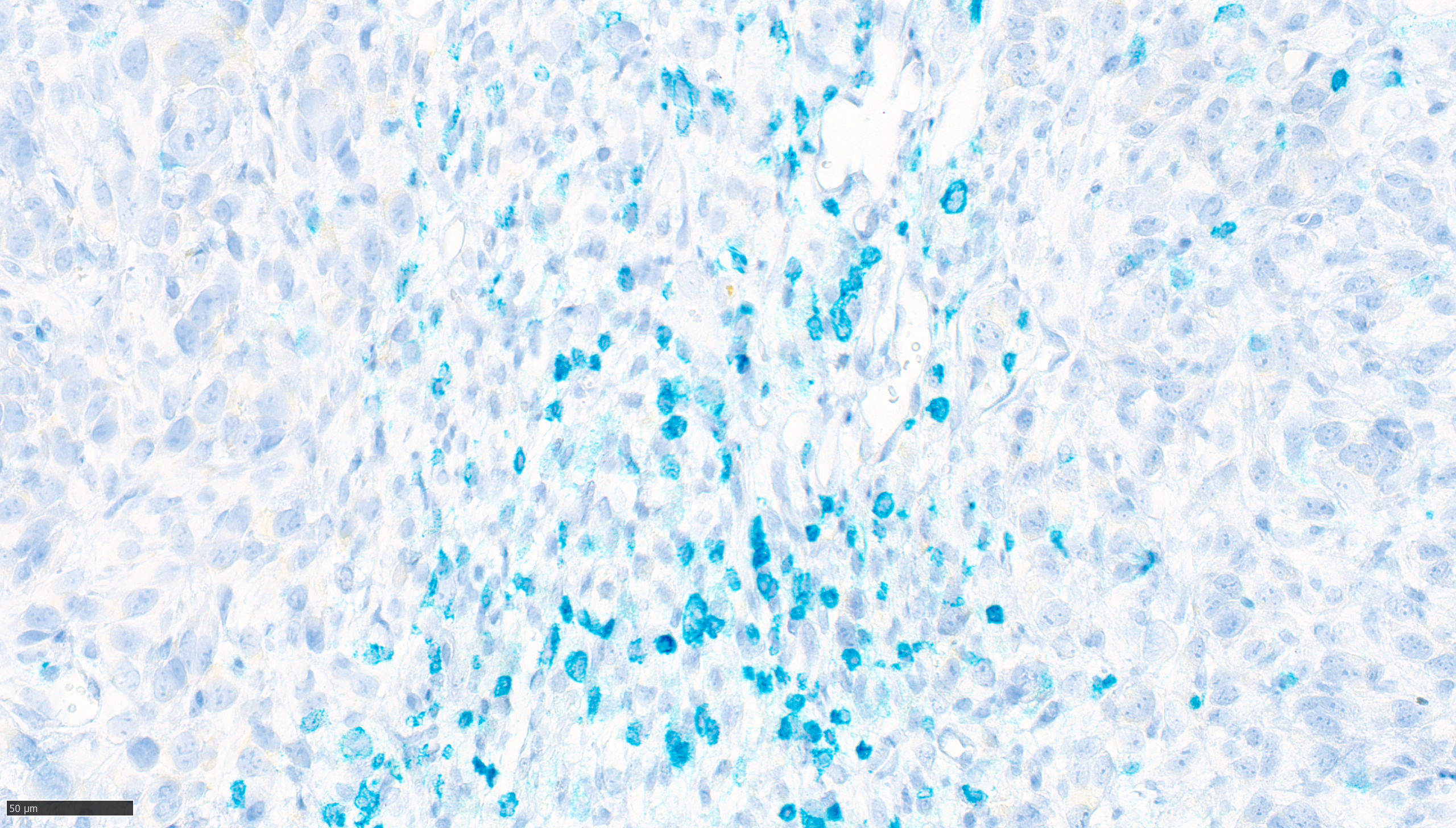

Supplement: Supplementary file 1 [file pharmaceutics-17-01273-s001.zip › IHC/CD4-CD8/LIFE BIOMATERIAL/L2/L2-1.jpg]

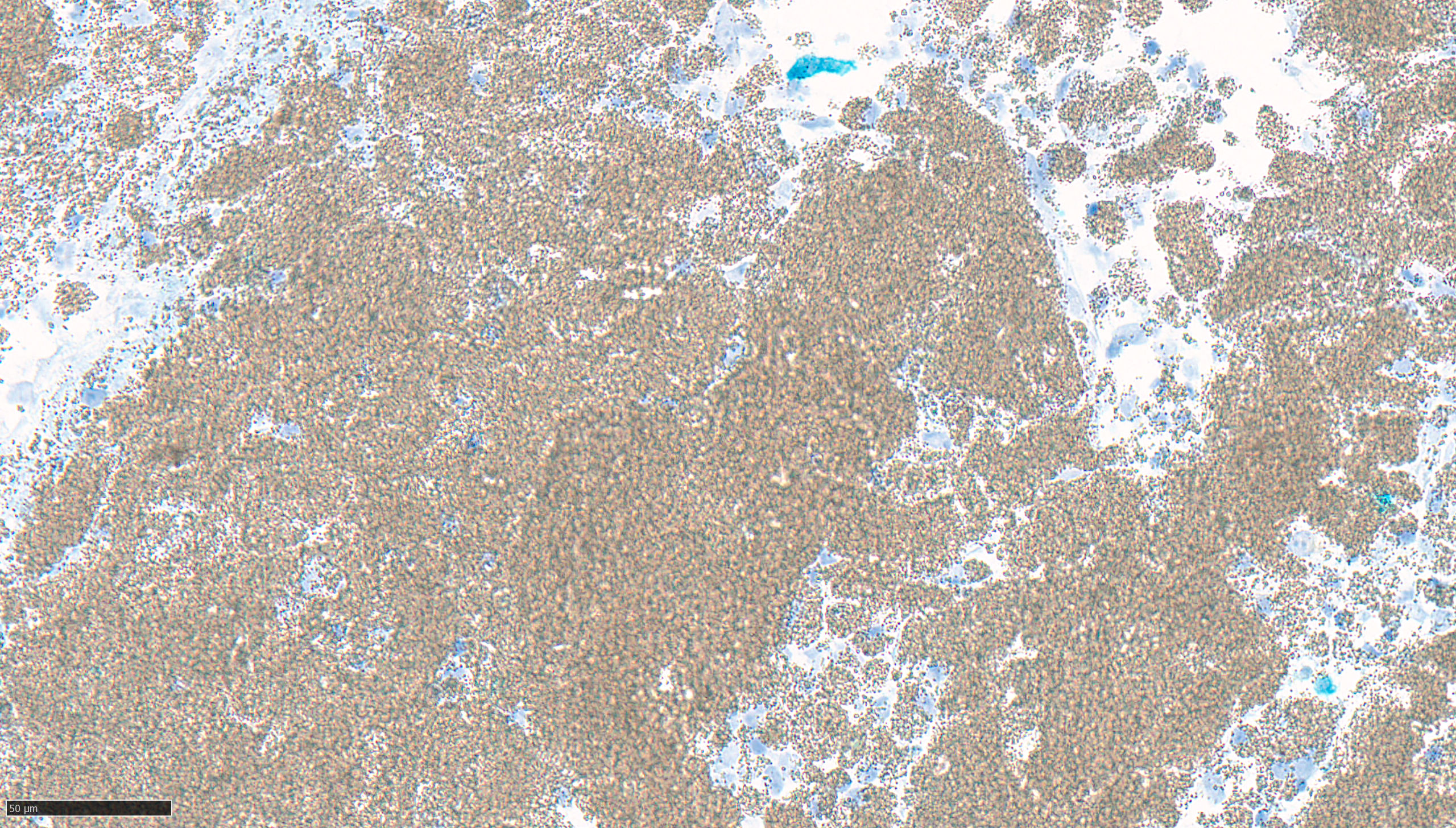

Supplement: Supplementary file 1 [file pharmaceutics-17-01273-s001.zip › IHC/CD4-CD8/LIFE BIOMATERIAL/L2/L2-2.jpg]

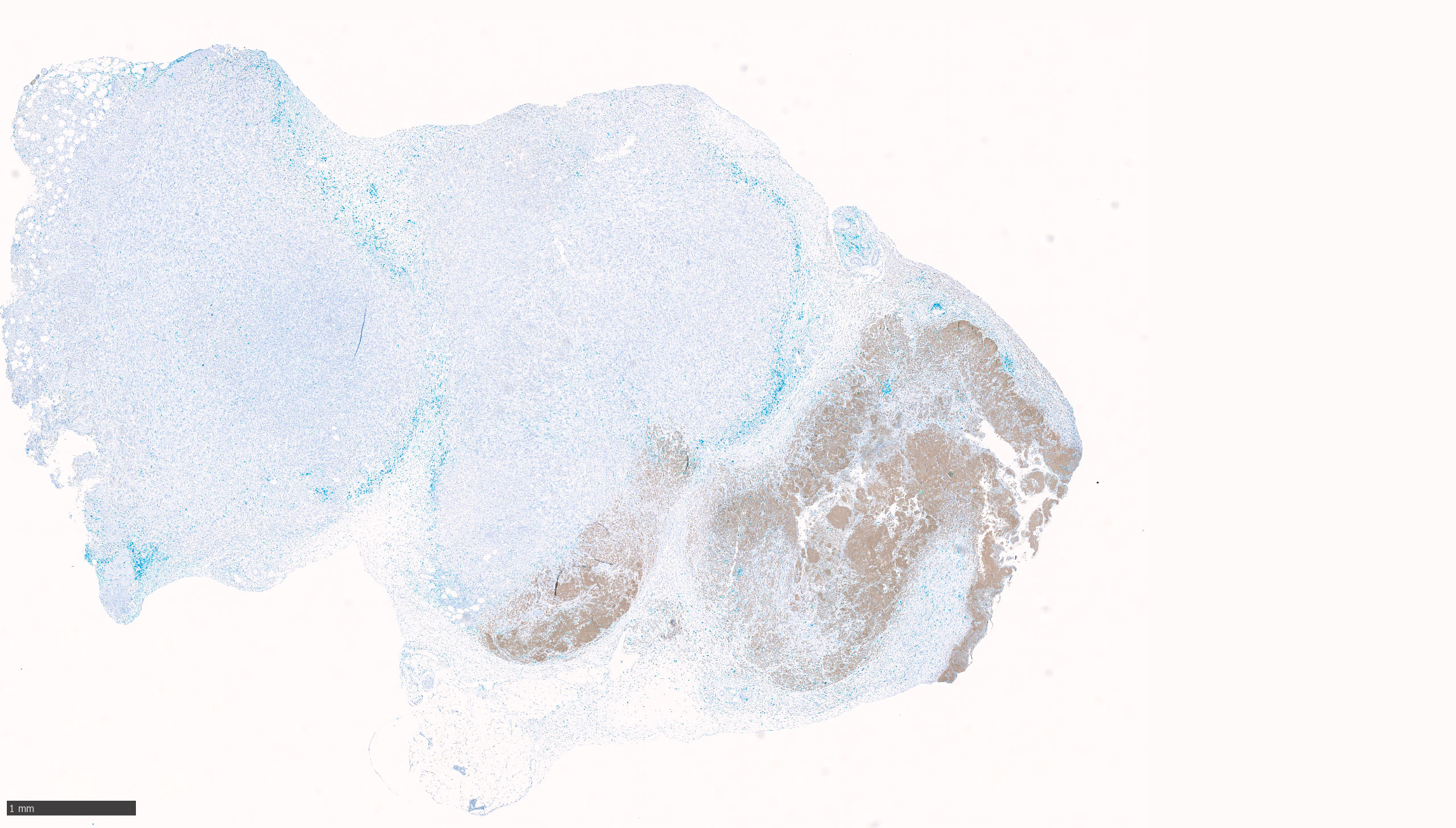

Supplement: Supplementary file 1 [file pharmaceutics-17-01273-s001.zip › IHC/CD4-CD8/LIFE BIOMATERIAL/L2/L2.jpg]

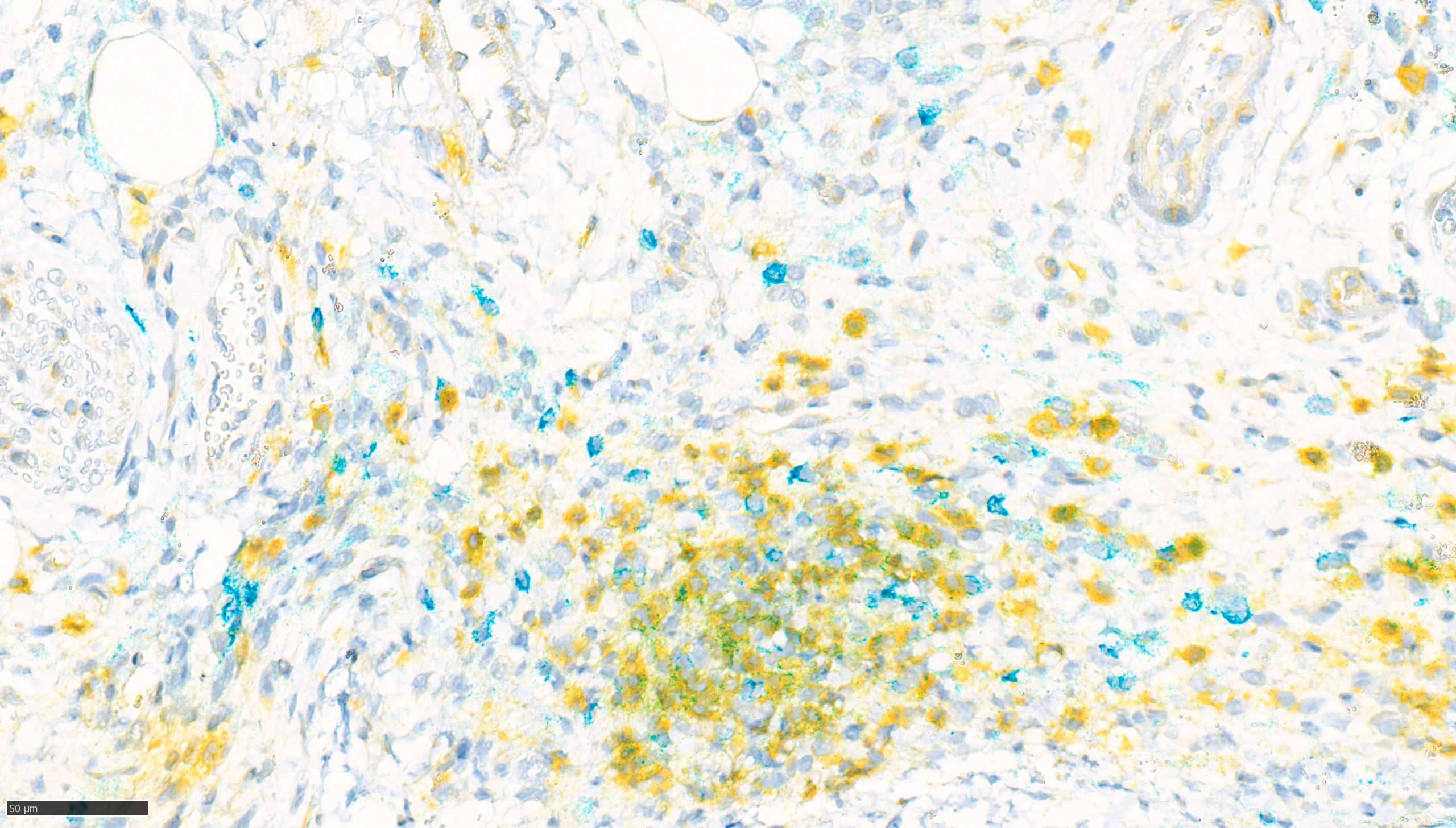

Supplement: Supplementary file 1 [file pharmaceutics-17-01273-s001.zip › IHC/CD4-CD8/LIFE BIOMATERIAL_CONV-5Gy/C5-L1/C5-L1-1.jpg]

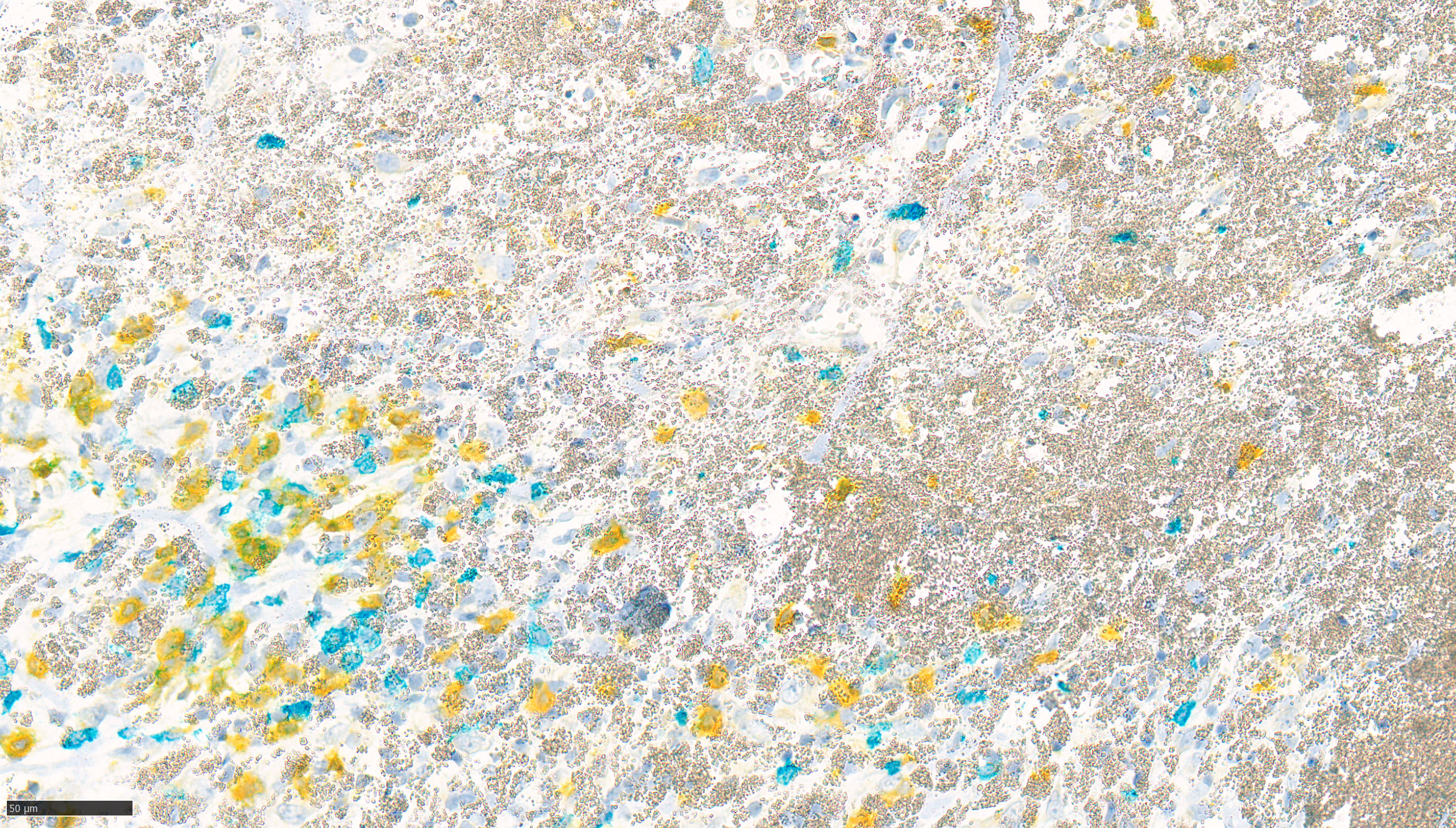

Supplement: Supplementary file 1 [file pharmaceutics-17-01273-s001.zip › IHC/CD4-CD8/LIFE BIOMATERIAL_CONV-5Gy/C5-L1/C5-L1-2.jpg]

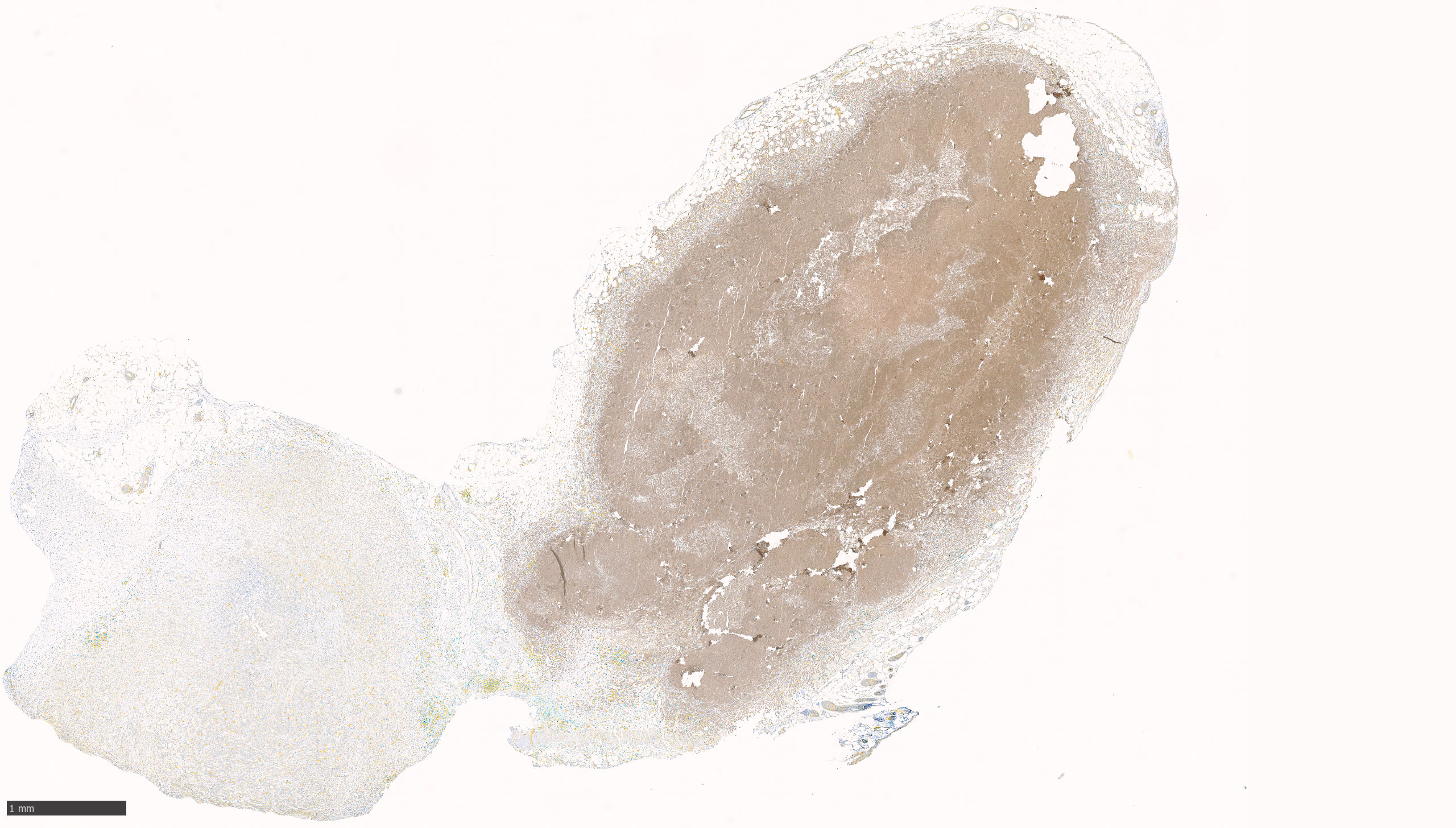

Supplement: Supplementary file 1 [file pharmaceutics-17-01273-s001.zip › IHC/CD4-CD8/LIFE BIOMATERIAL_CONV-5Gy/C5-L1/C5-L1.jpg]

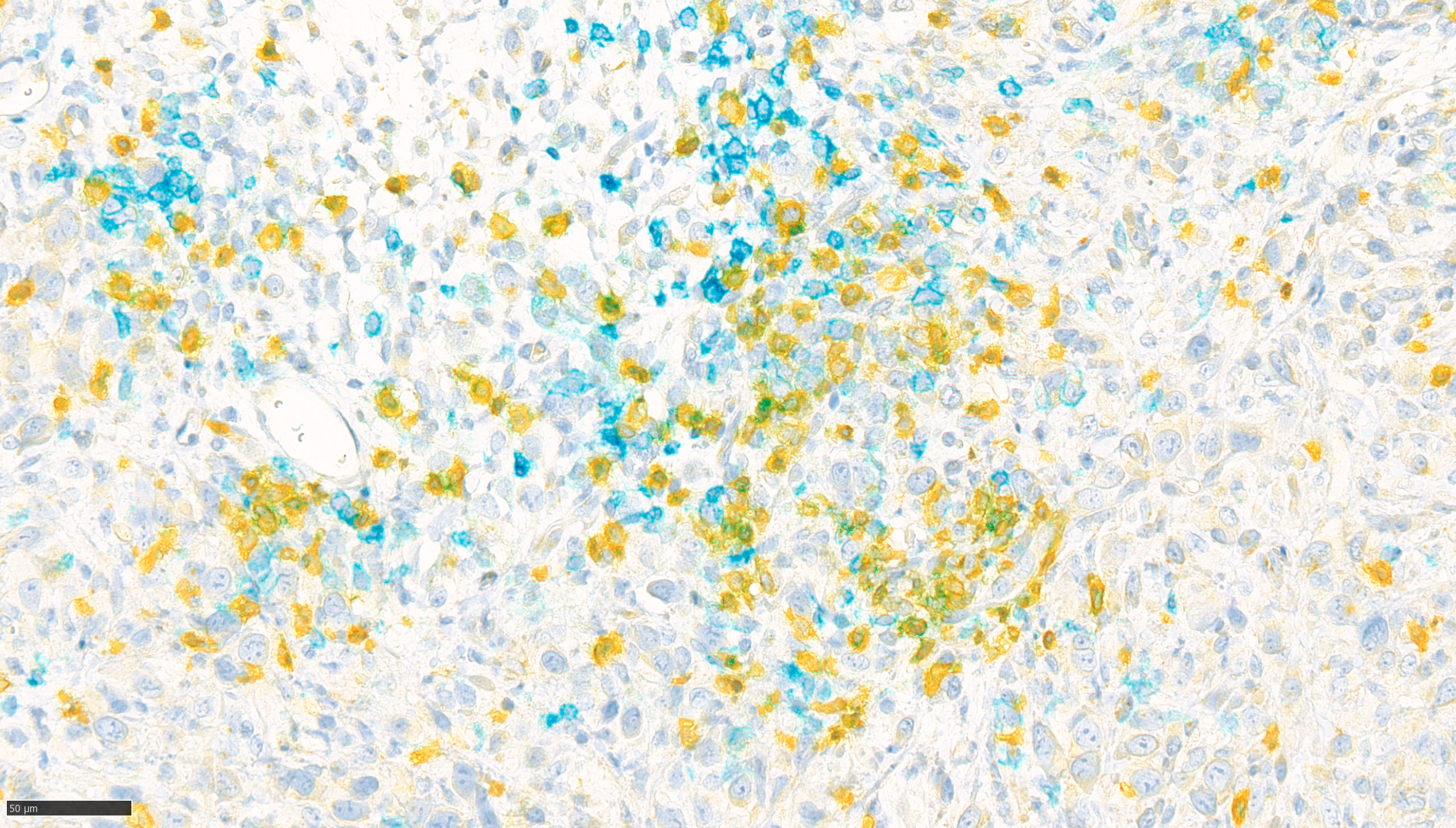

Supplement: Supplementary file 1 [file pharmaceutics-17-01273-s001.zip › IHC/CD4-CD8/LIFE BIOMATERIAL_CONV-5Gy/C5-L2/C5-L2-1.jpg]

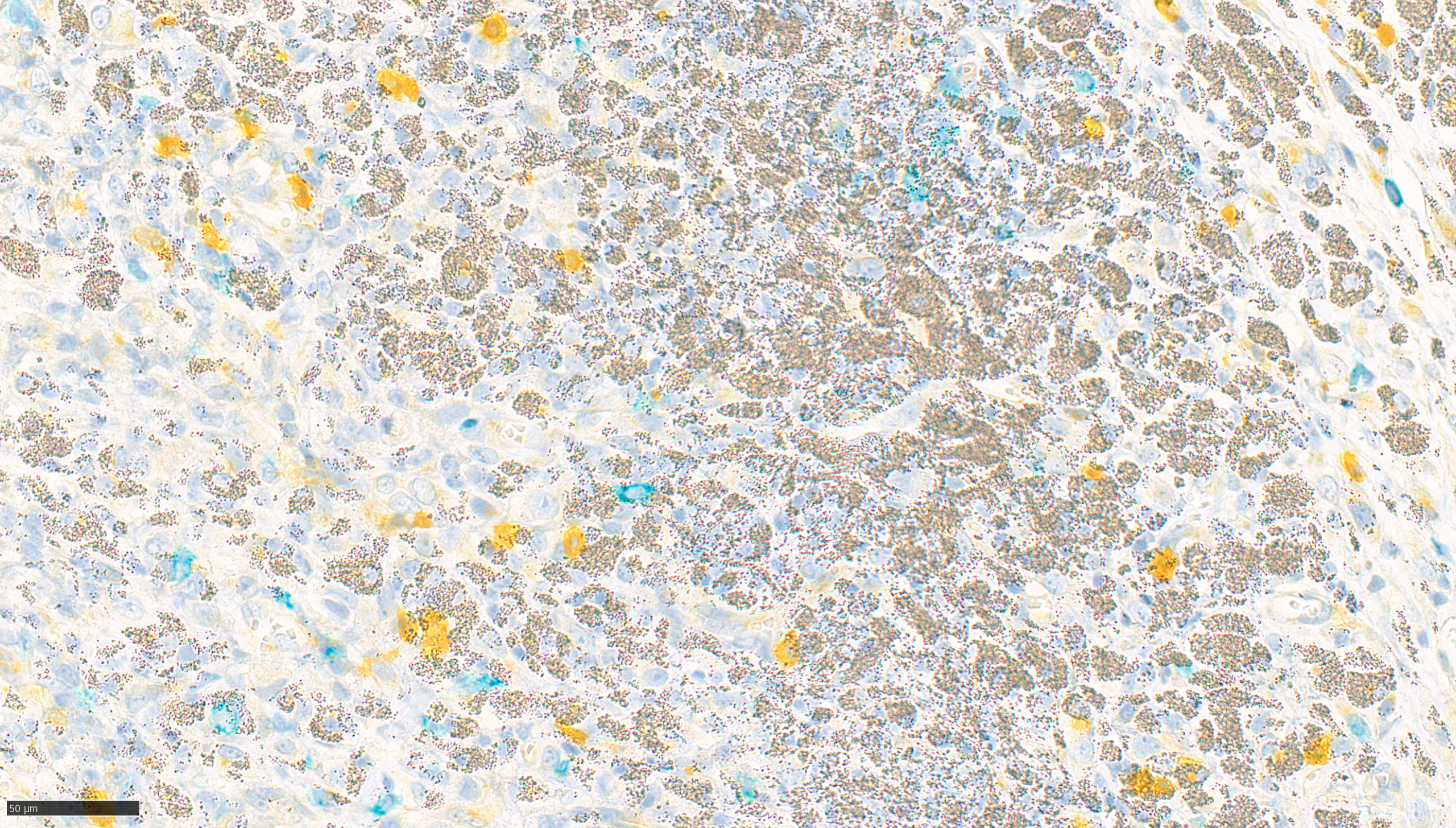

Supplement: Supplementary file 1 [file pharmaceutics-17-01273-s001.zip › IHC/CD4-CD8/LIFE BIOMATERIAL_CONV-5Gy/C5-L2/C5-L2-2.jpg]

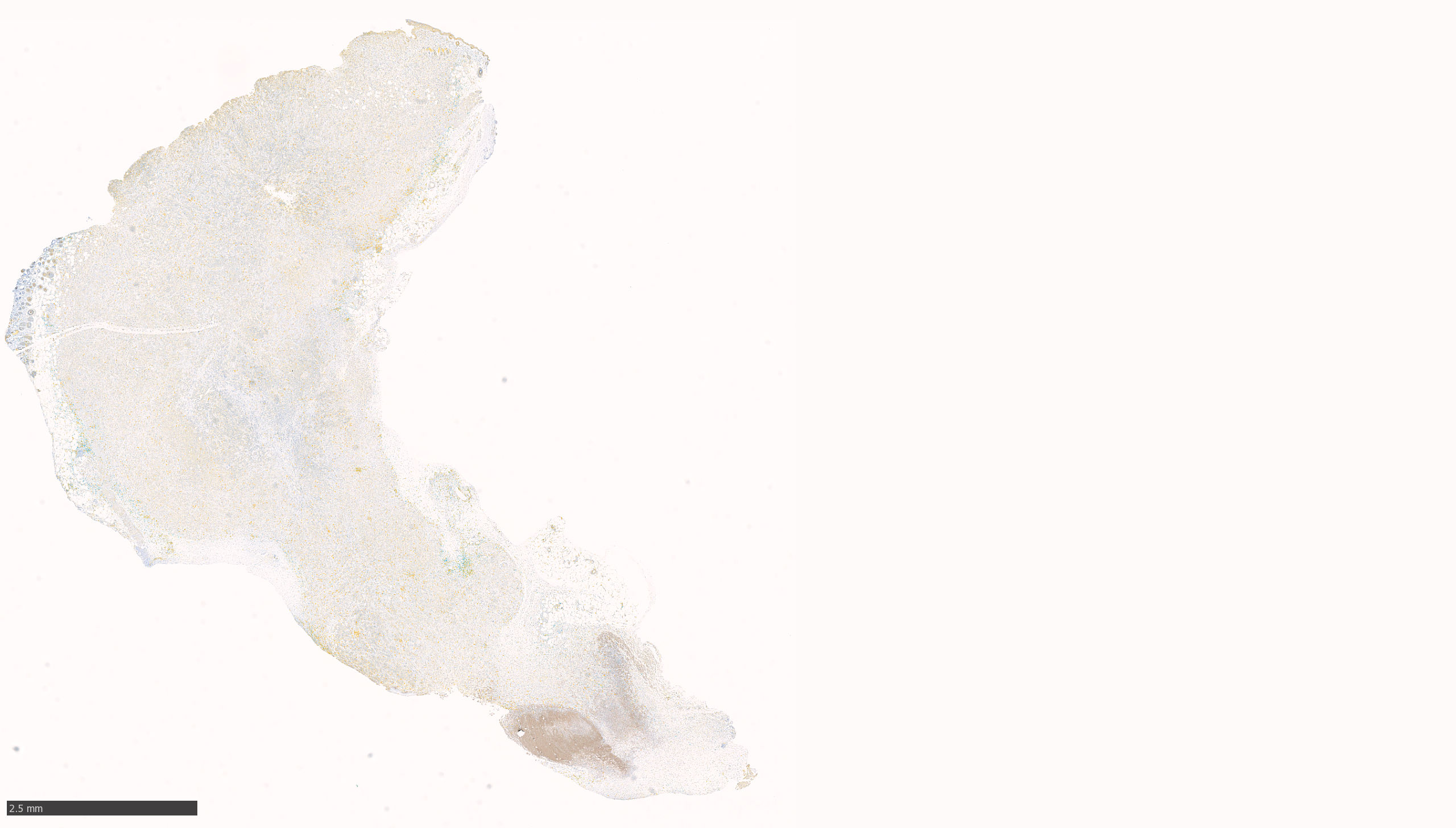

Supplement: Supplementary file 1 [file pharmaceutics-17-01273-s001.zip › IHC/CD4-CD8/LIFE BIOMATERIAL_CONV-5Gy/C5-L2/C5-L2.jpg]

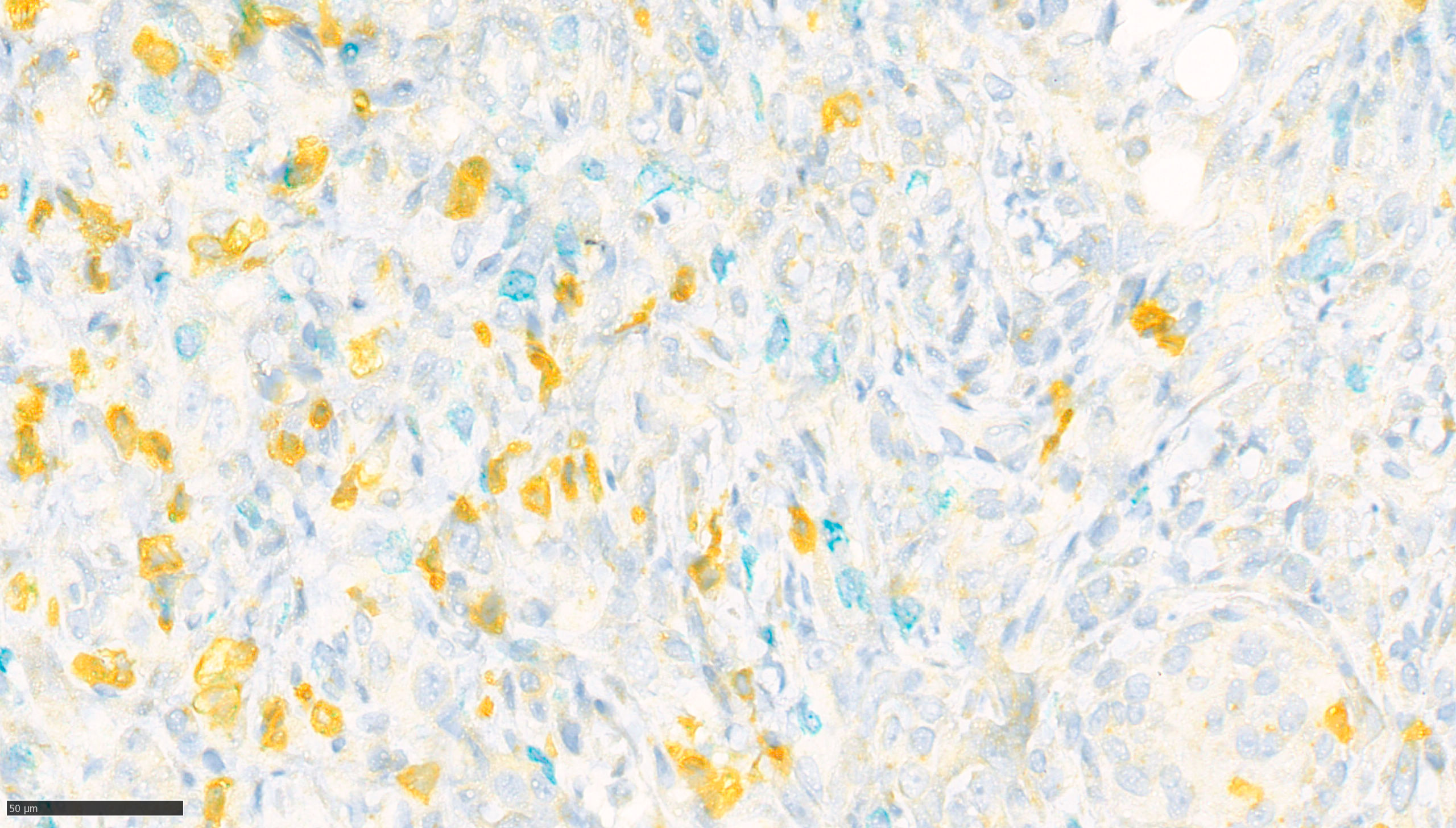

Supplement: Supplementary file 1 [file pharmaceutics-17-01273-s001.zip › IHC/CD4-CD8/LIFE BIOMATERIAL_CONV-8Gy/C8-L1/C8-L1-1.jpg]

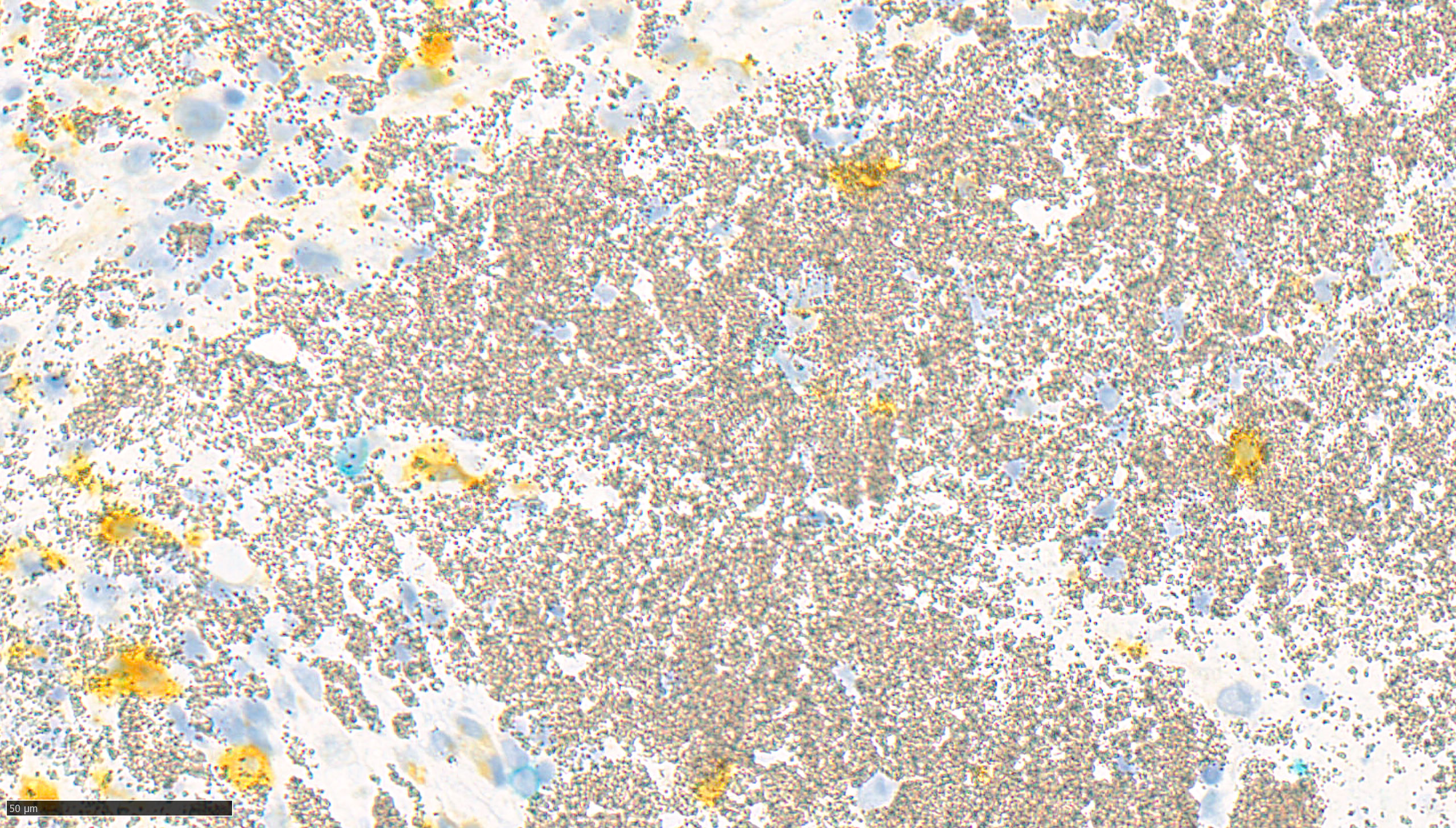

Supplement: Supplementary file 1 [file pharmaceutics-17-01273-s001.zip › IHC/CD4-CD8/LIFE BIOMATERIAL_CONV-8Gy/C8-L1/C8-L1-2.jpg]

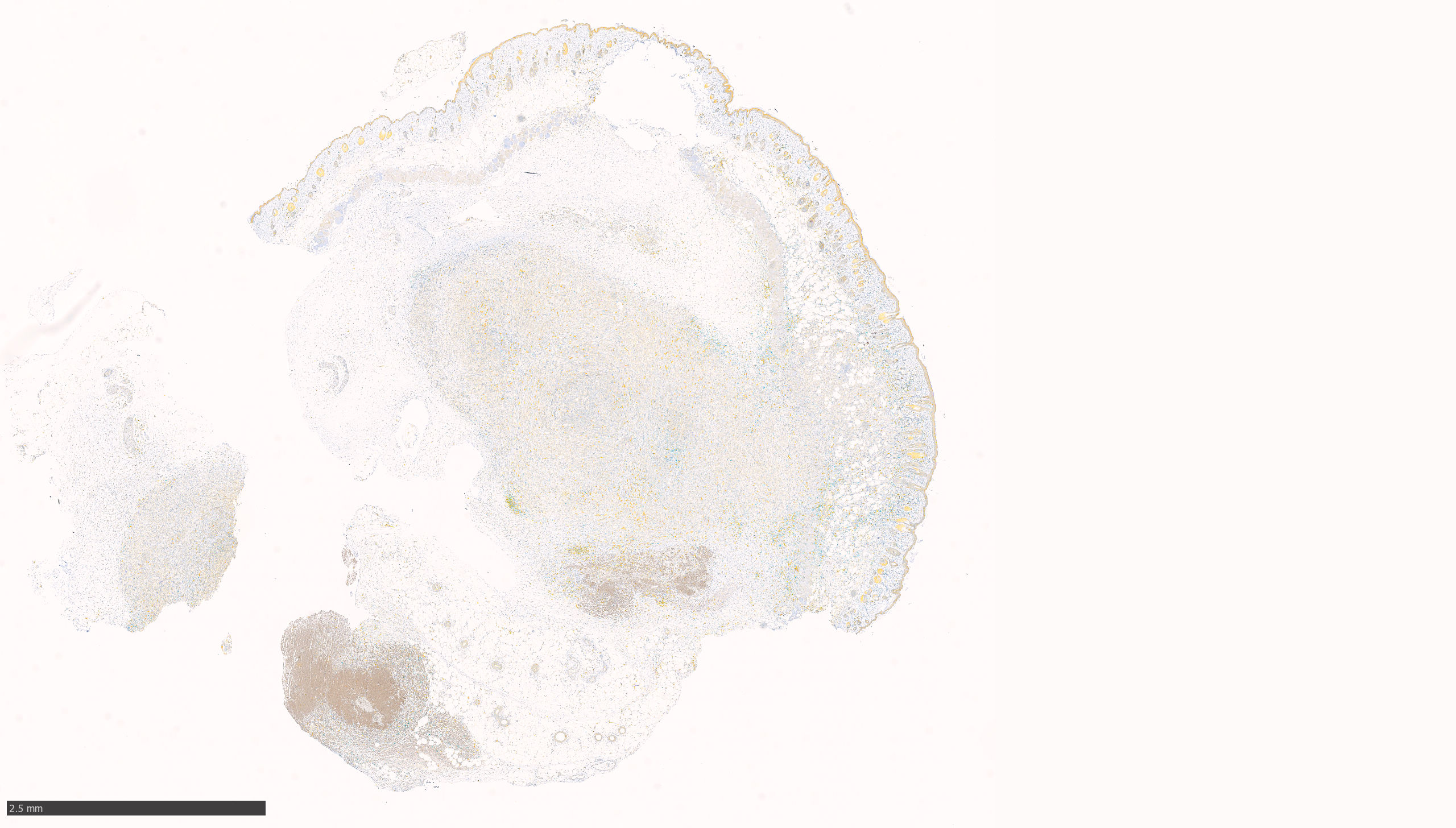

Supplement: Supplementary file 1 [file pharmaceutics-17-01273-s001.zip › IHC/CD4-CD8/LIFE BIOMATERIAL_CONV-8Gy/C8-L1/C8-L1.jpg]

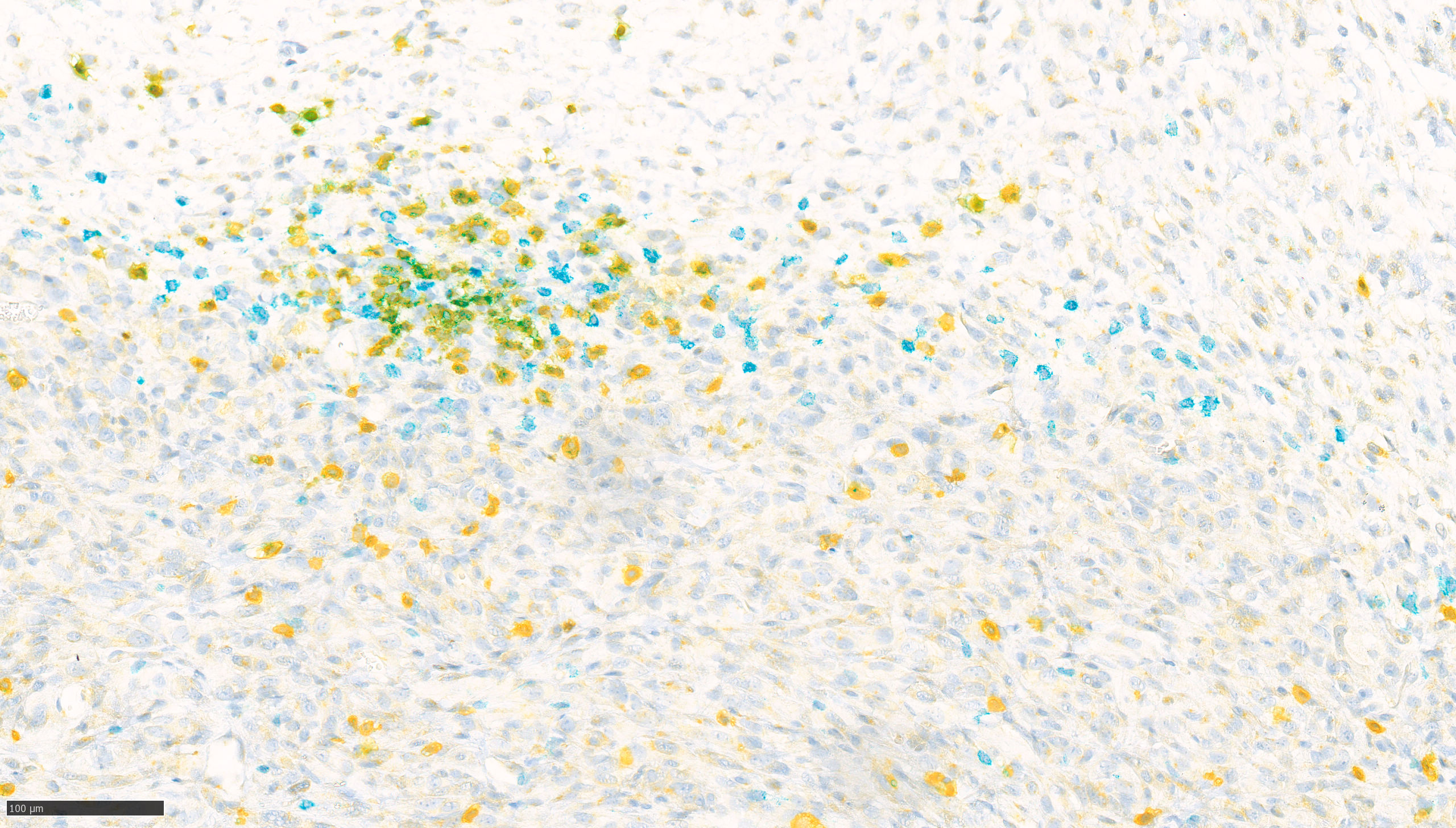

Supplement: Supplementary file 1 [file pharmaceutics-17-01273-s001.zip › IHC/CD4-CD8/LIFE BIOMATERIAL_CONV-8Gy/C8-L2/C8-L2-1.jpg]

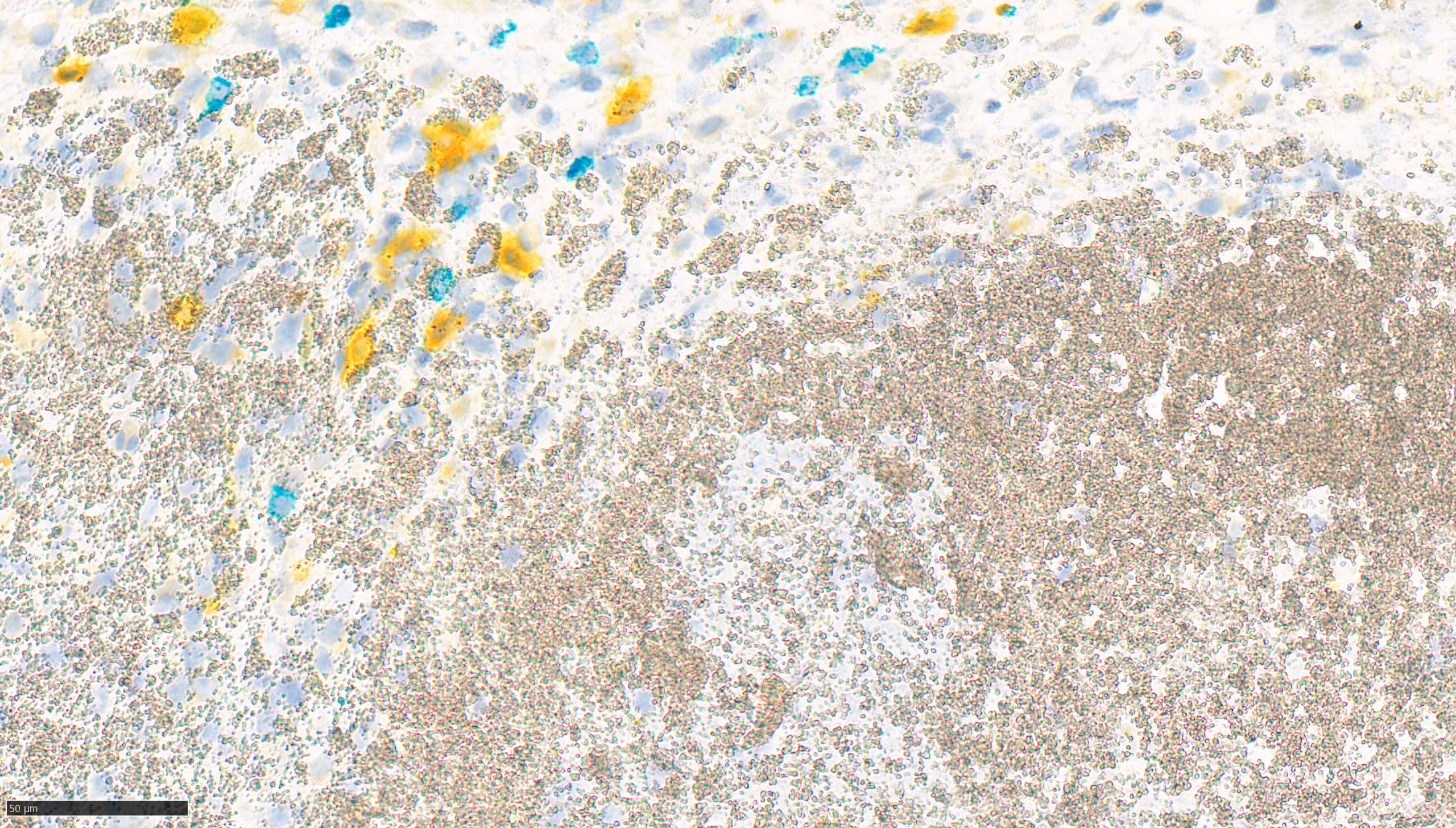

Supplement: Supplementary file 1 [file pharmaceutics-17-01273-s001.zip › IHC/CD4-CD8/LIFE BIOMATERIAL_CONV-8Gy/C8-L2/C8-L2-2.jpg]

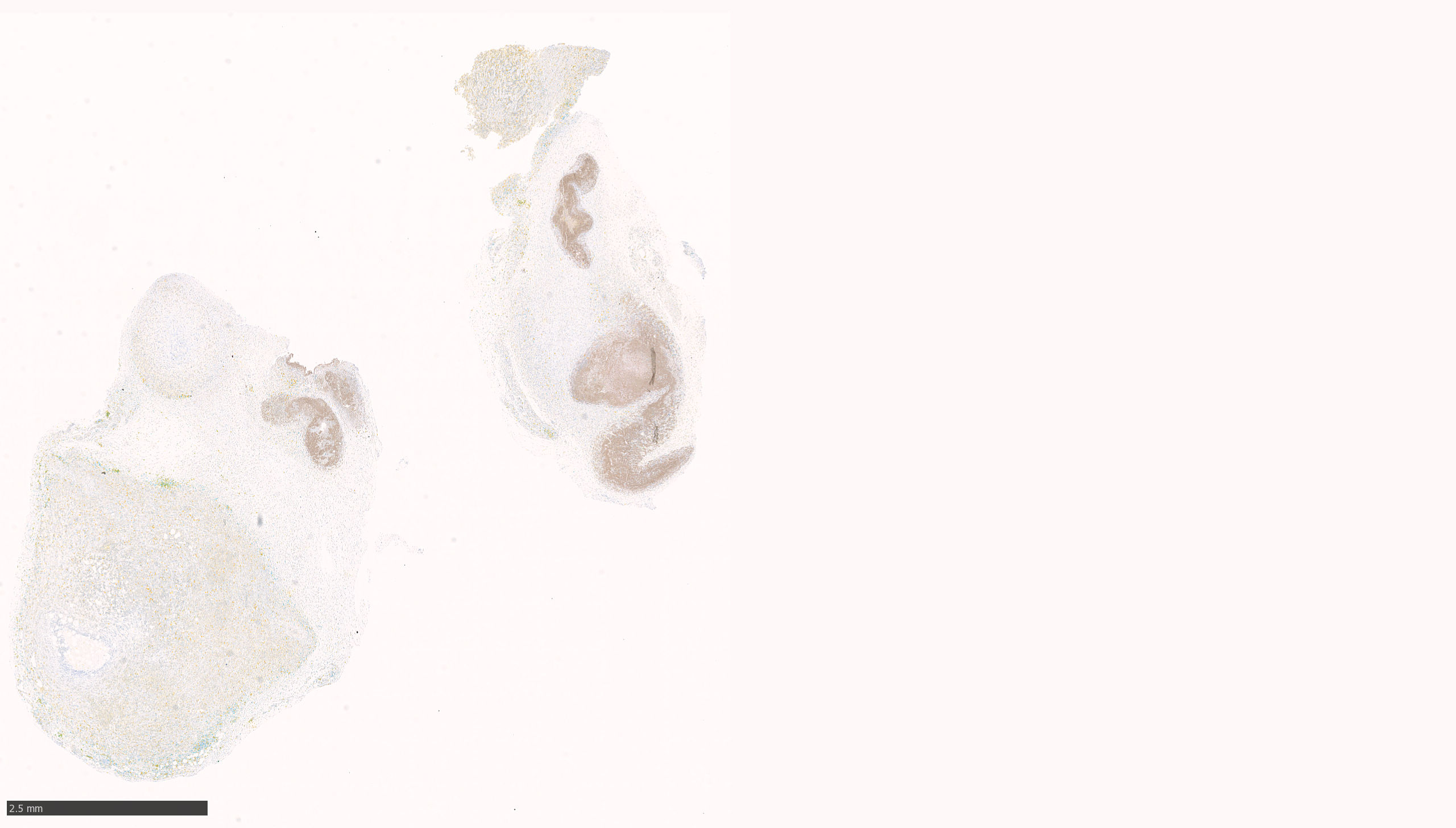

Supplement: Supplementary file 1 [file pharmaceutics-17-01273-s001.zip › IHC/CD4-CD8/LIFE BIOMATERIAL_CONV-8Gy/C8-L2/C8-L2.jpg]

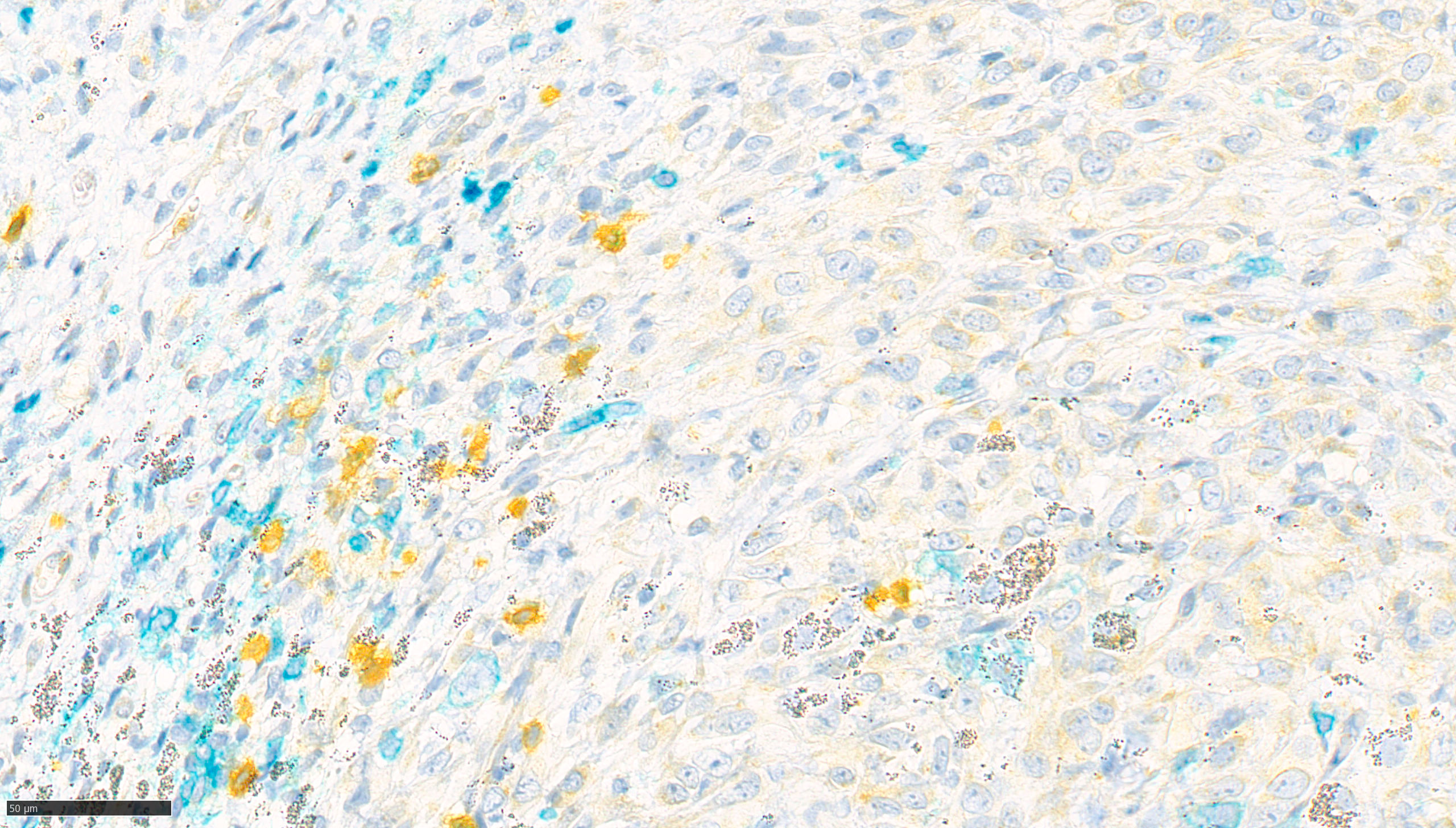

Supplement: Supplementary file 1 [file pharmaceutics-17-01273-s001.zip › IHC/CD4-CD8/LIFE BIOMATERIAL_FLASH 5Gy/F5-L1/F5-L1-1.jpg]
